# Multidirectionally Controlled Arrangement via Ion‐Pairing Assembly of Amphiphilic Charged π‐Electronic Systems

**DOI:** 10.1002/smll.202511729

**Published:** 2025-11-25

**Authors:** Yuto Maruyama, Biplab Manna, Koji Harano, Hayato Kanai, Yasuhiro Ishida, Hiromitsu Maeda

**Affiliations:** ^1^ Department of Applied Chemistry College of Life Sciences Kusatsu 525–8577 Japan; ^2^ Center for Basic Research on Materials National Institute for Materials Science Tsukuba 305–0044 Japan; ^3^ Research Center for Autonomous Systems Materialogy (ASMat) Institute of Integrated Research Institute of Science Tokyo Yokohama 226–8501 Japan; ^4^ Center for Emergent Matter Science (CEMS) RIKEN Wako 351–0198 Japan

**Keywords:** amphiphilic *^i^π*–*^i^π* interactions, charged π‐electronic systems, ion‐pairing assemblies, lateral hydrophobic effects, multi‐directional organization

## Abstract

Charged π‐electronic systems with hydrophilic substituents form lyotropic chromonic liquid crystals (LCLCs) through charge‐by‐charge assembly driven by *^i^π*–*^i^π* interactions and hydrophobic effects. In this study, the positions and numbers of triethylene glycol (TEG) chains in amphiphilic porphyrin Au^III^ complexes are tuned to control their assembly modes. In combination with the π‐electronic anion pentacyanocyclopentadienide (PCCp^–^), 5,15‐TEG‐aryl‐substituted porphyrin Au^III^ complex generates lamello‐columnar (Lam_col_) phases via amphiphilic *^i^π*–*^i^π* interactions and proximal interactions at the unsubstituted sites. In the presence of water, the Lam_col_ phases, with lateral hydrophobic effects, exhibit transitions to nematic sheet (N_sheet_) and isotropic sheet (Iso_sheet_) phases depending on water content and temperature. The Lam_col_ phases align macroscopically under a magnetic field, and scanning transmission electron microscopy (STEM) reveals monolayer sheet structures as key components of the LCLCs. These findings demonstrate a controllable charge‐by‐charge strategy for designing π‐electronic LCLCs with tunable structural and phase behaviors.

## Graphical abstract

Ion‐pairing lamello‐columnar lyotropic chromonic liquid crystals are formed from 2D sheet‐like structures via charge‐by‐charge assembly of partially hydrophilic‐aryl‐substituted porphyrin Au^III^ complex. The sheet‐like structures, arranged via amphiphilic *^i^π*–*^i^π* interactions and lateral hydrophobic effects, afford various phases, depending on water content and temperature.

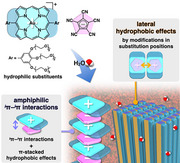

## 1 Introduction

**Figure 1:**
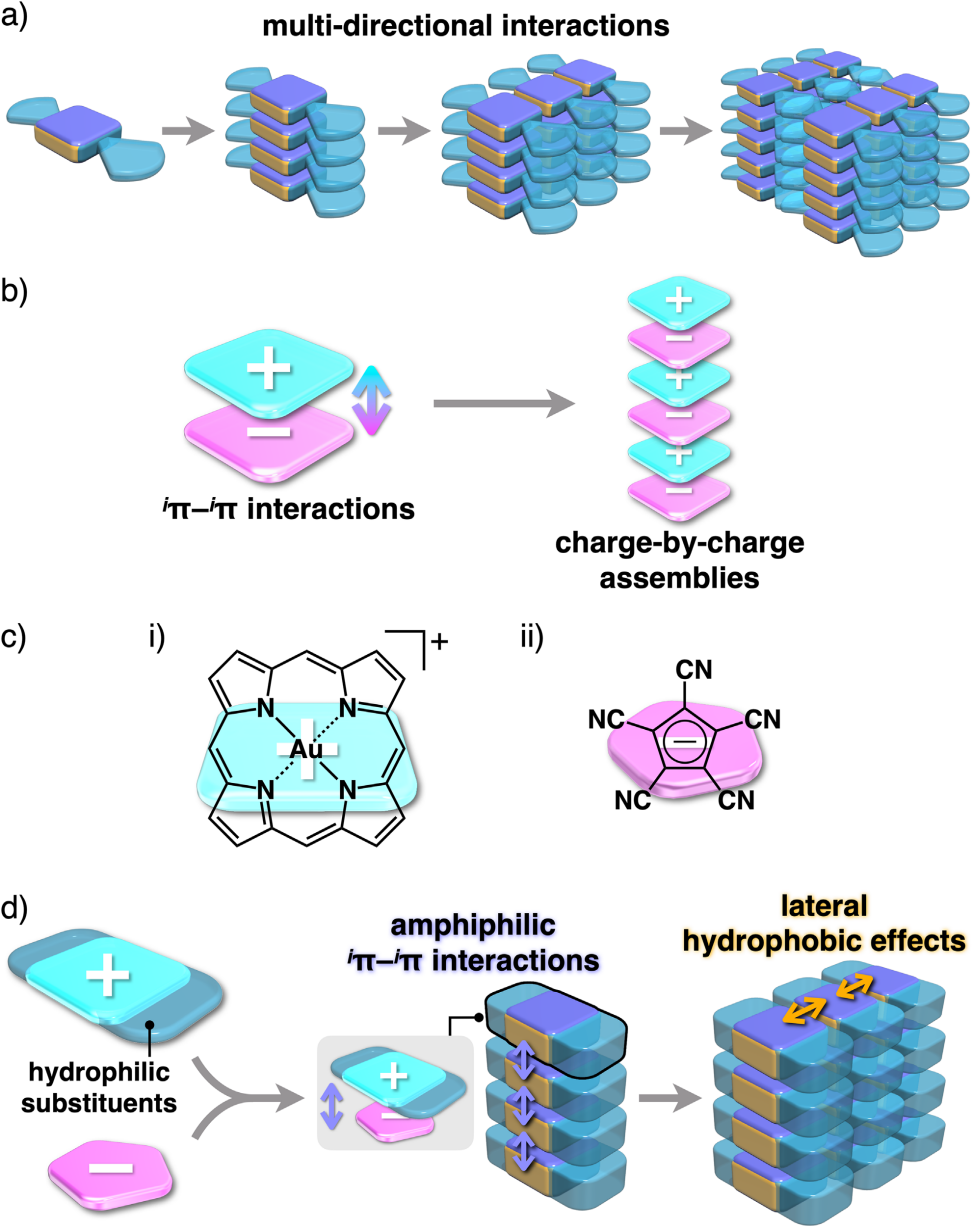
a) Self‐assembly, showing the formation of assembled structures depending on directional interaction sites, b) charge‐by‐charge assemblies via *^i^π*–*^i^π* interactions of π‐electronic ion pairs, c) i) porphyrin Au^III^ complexes, represented as a parent structure, and ii) PCCp^–^ as building units of ion‐pairing assemblies, and d) porphyrin Au^III^ complexes with hydrophilic substituents forming amphiphilic ion‐pairing assemblies depending on the peripheral substituents.

In supramolecular polymers,^[^ ^1^, ^2^, ^3^, ^4^, ^5^, ^6^, ^7^, ^8^, ^9^, ^10^, ^11^, ^12^, ^13^ ^]^ including fibers, ribbons, tubes, sheets, capsules, and 3D networks, building blocks are assembled via noncovalent interactions, such as hydrogen‐bonding, van der Waals, and *π*–*π* interactions, along with solvophobic effects. Rational molecular design enables precise control over the size and shape of nanostructures. In π‐electronic systems, *π*–*π* interactions among the core units induce the formation of columnar structures. Multi‐directional interactions among π‐electronic systems can control the arrangement of columns in nanostructures and their organized structures (**Figure** **1**a). In addition, structural features of assemblies are influenced by molecular geometries and the relative ratios of hydrophilic to hydrophobic parts.^[^ ^14^, ^15^, ^16^ ^]^ In amphiphilic π‐electronic systems, assembly in the lateral direction of the π‐planes occurs via hydrophobic effects at sites lacking hydrophilic chains, even in the absence of specific interaction sites.^[^ ^17^, ^18^ ^]^ Such hydrophobic effects are referred to as *lateral hydrophobic effects* in this study. Appropriate substituents in the sheet‐like structures assemble, inducing further ordered structures (Figure 1a). More dynamic assembled states, including solvents, such as lyotropic liquid crystals (LLCs), are formed through the aggregation of amphiphilic molecules driven by hydrophobic effects.^[^ ^19^ ^]^ Among LLCs, lyotropic chromonic liquid crystals (LCLCs), formed by π‐electronic systems, such as dyes and nucleic acids, exhibit molecular stacking not only through hydrophobic effects but also via *π*–*π* interactions,^[^ ^20^, ^21^, ^22^ ^]^ as seen in thermally responsive nanostructures of amphiphilic perylene bisimides.^[^ ^23^, ^24^, ^25^ ^]^ The ionic moieties of the mesogens and the corresponding counterions located outside the columnar structures enhance the affinity for water molecules.^[^ ^26^, ^27^, ^28^, ^29^, ^30^, ^31^, ^32^ ^]^

Introducing charges into π‐electronic core units generates charged π‐electronic systems and their corresponding ion‐pairing assemblies.^[^ ^33^, ^34^, ^35^, ^36^, ^37^, ^38^, ^39^ ^]^ In particular, alternately stacked π‐electronic cations and anions form charge‐by‐charge assemblies via *^i^π*–*^i^π* interactions, mainly comprising electrostatic and dispersion forces (Figure 1b).^[^ ^33^ ^]^ Charge‐by‐charge assemblies are observed in various forms, such as crystals and thermotropic liquid crystals, depending on the peripheral substituents.^[^ ^34^, ^35^, ^36^, ^37^, ^38^ ^]^ Among π‐electronic cations, porphyrin Au^III^ complexes have provided various ion‐pairing assemblies depending on the substituents and coexisting counteranions (Figure 1c (i)).^[^ ^34^, ^36^, ^38^ ^]^ An amphiphilic porphyrin Au^III^ complex that has hydrophilic aryl units at the four *meso*‐positions forms LCLCs in combination with pentacyanocylcopentadienide (PCCp^–^)^[^ ^40^, ^41^ ^]^ as a π‐electronic anion via the synergistic use of *^i^π*–*^i^π* interactions and hydrophobic effects between charged π‐electronic systems (Figure 1c (ii)).^[^ ^39^ ^]^ Throughout this report, such synergistic interactions will be termed *amphiphilic ^i^π–^i^π interactions*. Charge‐by‐charge columnar structures were unidirectionally oriented under a magnetic field, and single‐stranded charge‐by‐charge assemblies, as building units of LCLCs, were observed using transmission electron microscopy (TEM). The approach that uses amphiphilic *^i^π*–*^i^π* interactions is different from other strategies that include π–electronic systems bearing charged peripheral substituents.^[^ ^26^, ^27^, ^28^, ^29^, ^30^ ^]^ However, in our previous study, only LCLCs composed of 1D columnar assemblies were formed owing to nanoscale phase separation between charge‐by‐charge columns and peripheral hydrophilic aryl units. Multidirectionally controlled arrangement in LCLCs requires specific interaction sites that promote lateral packing between the columns. Modifying the substitution positions of hydrophilic aryl units in porphyrin Au^III^ complexes, together with π‐electronic anions, would facilitate the formation of LCLCs based on sheet‐like structures through proximal interactions at the unsubstituted sites (Figure 1d). This study shows the multi‐directional arrangement of π‐electronic ion pairs, providing, to the best of our knowledge, the first example of lamello‐columnar (Lam_col_)‐based LCLCs, via lateral hydrophobic effects between amphiphilic charge‐by‐charge columnar structures.

## 2 Results and Discussion

### 2.1 Synthesis and Characterization of Amphiphilic π‐Electronic Cations

**Figure 2:**
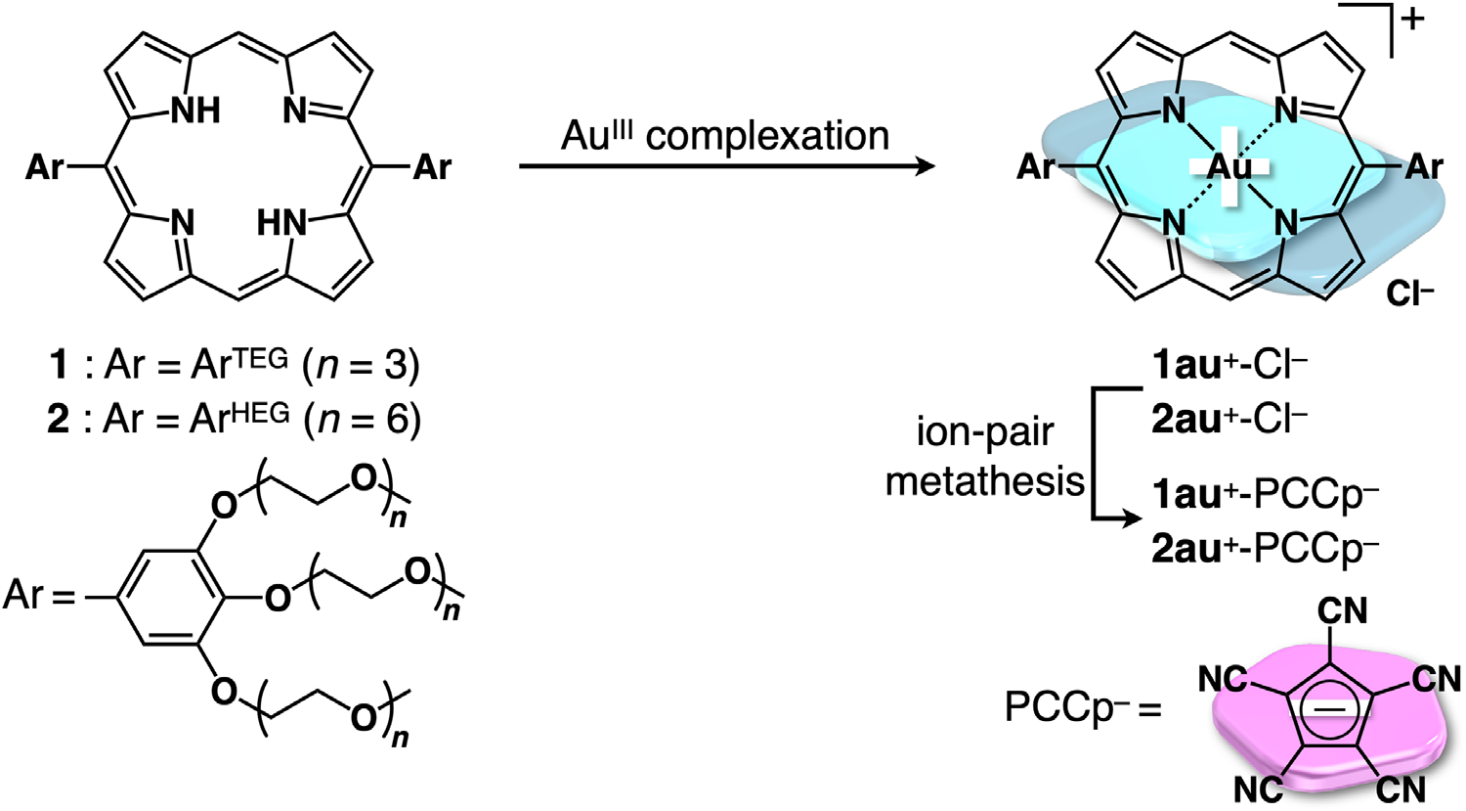
Synthesis of amphiphilic porphyrin Au^III^ complexes as ion pairs.

Hydrophilic substituents such as CH_3_(OCH_2_CH_2_)_3_O (triethylene glycol, TEG) moieties were introduced for hydration to form assemblies in aqueous media. Amphiphilic porphyrin Au^III^ complex **1au** ^+^ (**Figure** **2**) was synthesized as a Cl^–^ ion pair by Au^III^ complexation of 5,15‐bis(3,4,5‐trisTEG‐substituted aryl)porphyrin^[^ ^42^ ^]^ **1** by treatment with KAuCl_4_·*n*H_2_O and NaOAc·3H_2_O in AcOH. PCCp^–^ was introduced to provide π‐electronic ion pair **1au** ^+^‐PCCp^–^ by ion‐pair metathesis of **1au** ^+^‐Cl^–^ with NaPCCp (Figure 2). As a reference for the relative ratio between hydrophilic and hydrophobic parts, amphiphilic porphyrin Au^III^ complex **2au** ^+^ with CH_3_(OCH_2_CH_2_)_6_O (hexaethylene glycol, HEG) chains instead of TEG chains was also synthesized as a PCCp^–^ ion pair. The synthesized ion pairs were characterized using ^1^H and ^13^C NMR spectroscopy and ESI‐TOF‐MS. In CDCl_3_ (1 mm) at 20 °C, the ^1^H NMR signals of β‐CH proximal to Ar^TEG^ (9.67 ppm) and aryl‐CH (7.69 ppm) of **1au** ^+^ in **1au** ^+^‐PCCp^–^ showed downfield shifts compared to those of **1au** ^+^‐Cl^–^ (9.56 and 7.52 ppm, respectively) (Figures S4, S6, S8, and S9, Supporting Information). In contrast, the signals of β‐CH on the far side to Ar^TEG^ (9.86 ppm) and *meso*‐CH (11.07 ppm) of **1au** ^+^ in **1au** ^+^‐PCCp^–^ were shifted upfield compared to those of **1au** ^+^‐Cl^–^ (10.05 and 11.47 ppm, respectively). These shifts could be attributed to the location of *meso*‐CH on the π‐plane of PCCp^–^ in the π‐stacked ion pair (*π‐sip*).

In CH_2_Cl_2_ (≤0.10 mm), UV/vis absorption spectra of **1au** ^+^‐Cl^–^ and **1au** ^+^‐PCCp^–^ showed the maxima (λ_max_) of the Soret band at 399 nm, suggesting that the ion pairs exist in dispersed states without aggregation (Figure S16, Supporting Information).^[^ ^43^ ^]^ In contrast, in aqueous solutions (0.10 mm), **1au** ^+^‐Cl^–^ and **1au** ^+^‐PCCp^–^ exhibited blue‐shifted absorptions with the λ_max_ at 390 and 398 nm, respectively (Figure S16, Supporting Information). The difference in the blue shifts can be attributed to the anion‐dependent assembly modes. The separation with a distance of ≈0.7 nm in a charge‐by‐charge assembly of **1au** ^+^‐PCCp^–^ induced a smaller exciton coupling between **1au** ^+^ units and resulted in a slight blue shift (1 nm). Charge‐by‐charge assembly was formed via effective amphiphilic *^i^π*–*^i^π* interactions (**1au** ^+^ and PCCp^–^). In contrast, **1au** ^+^‐Cl^–^, with the hydration of Cl^–^, formed a stacking arrangement of **1au** ^+^ as suggested by the larger blue shift (9 nm). In aqueous solutions, these ion pairs constructed assembled structures with sizes of ≈500 nm, as indicated by DLS measurements (Figure S20, Supporting Information).

### 2.2 Thermotropic Liquid Crystals of Amphiphilic Ion Pairs

**Figure 3:**
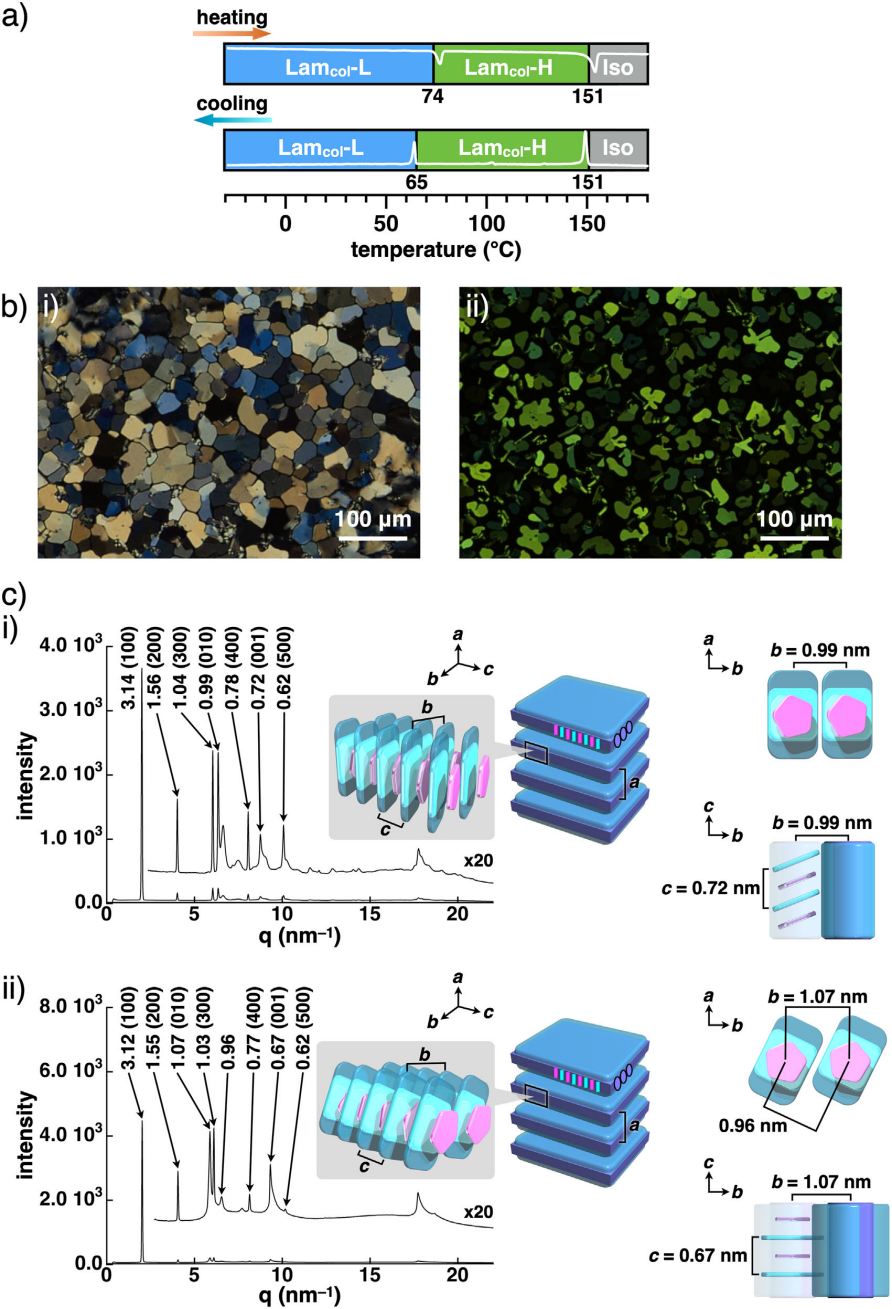
a) Phase transition behavior of **1au** ^+^‐PCCp^–^ with DSC profiles, b) POM images at i) 20 °C and ii) 150 °C upon cooling, and c) synchrotron XRD and possible assembled models at i) 20 °C and ii) 150 °C upon heating. The axis labels in c) correspond to the packing parameters.

According to solution behavior, bulk‐state ion‐pairing assembled structures were examined. In **1au** ^+^‐PCCp^–^, a paste‐state sample precipitated from CHCl_3_/*n*‐hexane,^[^ ^44^ ^]^ the transitions at 74 and 151 °C (heating) and 151 and 65 °C (cooling) were observed via differential scanning calorimetry (DSC) (**Figure** **3**a; Figure S26, Supporting Information). Polarized optical microscopy (POM) images showed mosaic textures at 20 and 150 °C upon cooling from the isotropic liquid (Iso) state (Figure 3b). Synchrotron X‐ray diffraction (XRD) at 20 °C upon heating showed the diffraction pattern derived from the Lam_col_ structure (Lam_col_‐L) with *a* = 3.14 nm, *b* = 0.99 nm, and *c* = 0.72 nm (Figure 3c (i)), and that at 150 °C upon heating revealed the Lam_col_ structure (Lam_col_‐H) with *a* = 3.12 nm, *b* = 1.07 nm, and *c* = 0.67 nm (Figure 3c (ii)). The sharp diffraction peaks in these Lam_col_ structures would be derived from the ordered arrangement of **1au** ^+^ and PCCp^–^. The observed *a* values indicated constituent sizes that were consistent with the model structure of **1au** ^+^ with folded TEG chains (Figure S69, Supporting Information), whereas the *b* values were attributed to the intercolumnar distance along the *b* axis resulting from the packing between the unsubstituted sites of **1au** ^+^. The *c* values reflect an alternately stacked arrangement of ion pairs, which is typical of charge‐by‐charge assemblies. The formation of Lam_col_ phases depended on the substitution position of the hydrophilic aryl units; an intercolumnar arrangement was observed along the *b* axis. In contrast, the paste‐state **1au** ^+^‐Cl^–^, precipitated from CHCl_3_/*n*‐hexane, showed a complex synchrotron XRD pattern derived from the highly crystalline mesophase (Figure S70, Supporting Information),^[^ ^45^ ^]^ suggesting that coexisting anions in the ion pairs of **1au** ^+^ are essential for assembly.

The shearing‐induced alignment of the sample between the polyimide films contributed to revealing the difference between the two Lam_col_ structures (Lam_col_‐L and ‐H) through synchrotron XRD analysis (Figures S77 and S78, Supporting Information). The diffractions at 25 °C derived from the lamellar pattern in Lam_col_‐L were augmented in the equatorial direction. In contrast, those derived from the ordered arrangement of columns in a sheet structure and charge‐by‐charge assembly mode increased in the shearing (meridional) direction. Upon heating at 150 °C, the Lam_col_‐H structure retained the shear‐induced alignment, exhibiting the same orientation as the Lam_col_‐L structure at 25 °C. The alignment tendency suggests that the sheet‐like structures were oriented perpendicularly to the polyimide films by the shearing process, with contributions from two types of domains showing either the *b* or *c* axis parallel to the shearing direction (Figure S78, Supporting Information). In the shearing direction, spacings of 0.71 and 0.67 nm for the Lam_col_‐L structure at 25 °C and the Lam_col_‐H structure at 150 °C, respectively, were observed, corresponding to the (001) faces of the alternately stacked ion‐pairing structures. The values of 0.99 and 1.07 nm, which were observed in the shearing direction of the Lam_col_‐L structure at 25 °C and the Lam_col_‐H structure at 150 °C, respectively, could be attributed to the (010) faces, the repeating intercolumnar distance along the *b* axis (Figure S77, Supporting Information).^[^ ^46^, ^47^ ^]^ As indicated by the *b* and *c* values in the Lam_col_‐L structures, the proposed sheet structures were formed by slipped stacked columns without the tilted **1au** ^+^ core units (Figure 3c (i)). In contrast, in the possible Lam_col_‐H structures, the sheet structures were formed with tilted **1au** ^+^ cores without slipped stacking (Figure 3c (ii)). This tilted arrangement could be suggested by the value of 0.96 nm observed 25° away from the shearing direction at 150 °C (Figure S77, Supporting Information).^[^ ^48^, ^49^ ^]^ Higher temperatures induced larger *a* values in the Lam_col_‐L structures because of the spreading of the TEG chains along the *a* axis (Figure S72, Supporting Information). In contrast, in the Lam_col_‐H structures, the spreading of the TEG chains within the *b*–*c* plane, attributed to the elongation of the lateral distance between the unsubstituted sites of **1au** ^+^ with the tilted cores, resulted in smaller *a* values.^[^ ^50^ ^]^ This behavior revealed that thermotropic liquid crystals were formed based on highly ordered assemblies, ascribed to charge‐by‐charge stacking of **1au** ^+^ and PCCp^–^ via amphiphilic *^i^π*–*^i^π* interactions and also to lateral hydrophobic effects at proximally located unsubstituted sites of **1au** ^+^.

### 2.3 Lyotropic Chromonic Liquid Crystals of Amphiphilic Ion Pairs

**Figure 4:**
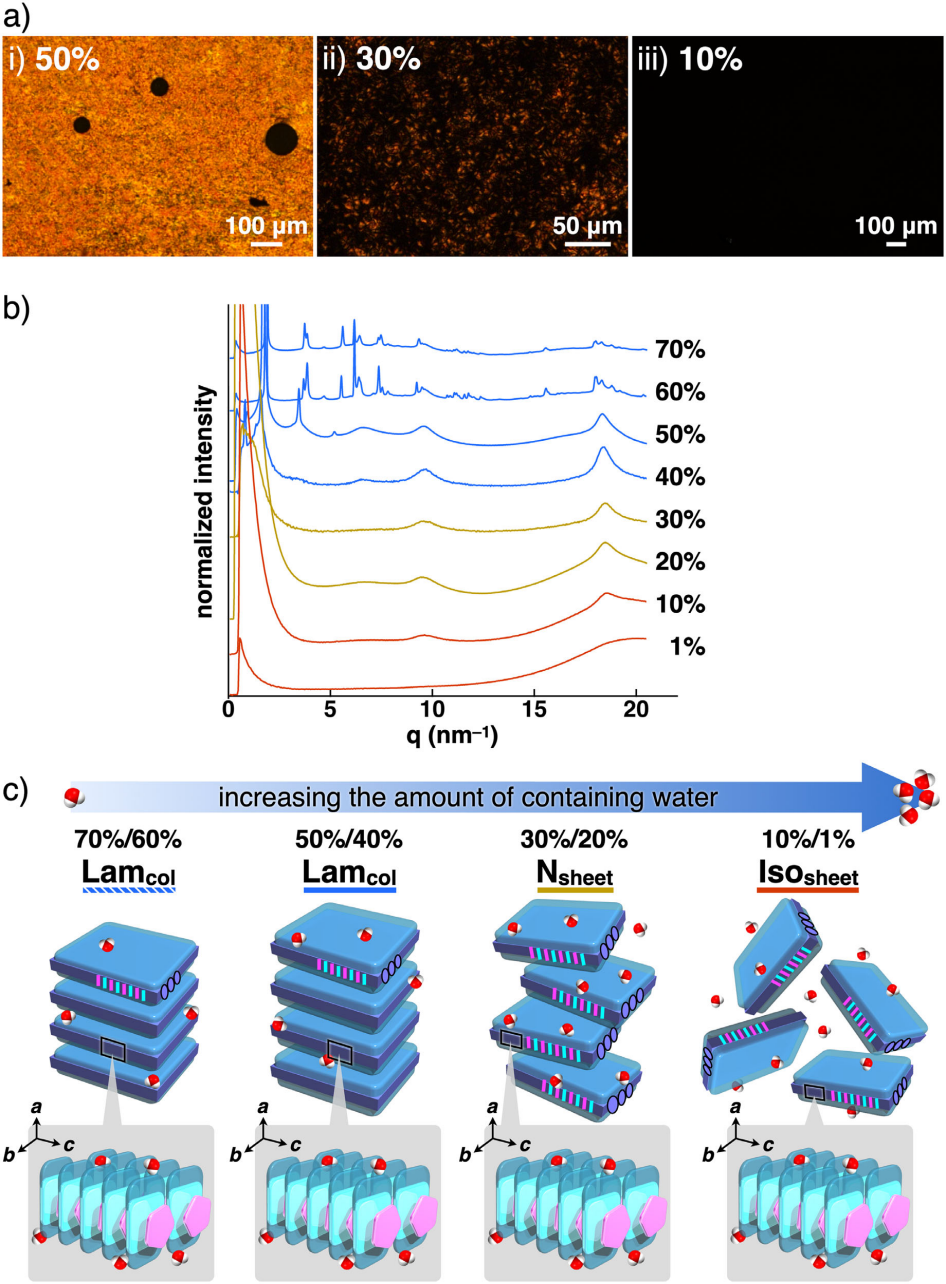
a) POM images of i) **1au** ^+^‐PCCp^–^_50%_, ii) **1au** ^+^‐PCCp^–^_30%_, and iii) **1au** ^+^‐PCCp^–^_10%_ at 20 °C, b) synchrotron XRD of **1au** ^+^‐PCCp^–^ and water‐containing **1au** ^+^‐PCCp^–^ at 25 °C, and c) phase changes according to the ion‐pair content. Shaded bars show the highly ordered mesophases.

The assembly behavior of the water‐containing states of **1au** ^+^‐PCCp^–^ was further examined. The water‐containing **1au** ^+^‐PCCp^–^, in the percentages (w/w) of ion pairs to the total amounts (ion‐pair content) of 70–1%, were prepared from **1au** ^+^‐PCCp^–^ (**1au** ^+^‐PCCp^–^_100%_) and the corresponding amounts of water. The POM textures at r.t. were dependent on the ion‐pair content (**Figure** **4**a). The water‐containing states with ion‐pair content of 70%, 60%, 50%, and 40%, **1au** ^+^‐PCCp^–^_70%/60%/50%/40%_ labeled with the corresponding content, formed Lam_col_ structures at r.t., as revealed by synchrotron XRD, whereas **1au** ^+^‐PCCp^–^_30%_ and the states with higher water amounts showed no diffraction peaks derived from Lam_col_‐based structures (Figure 4b,c). The *a* values of 3.39, 3.44, 3.67, and 3.96 nm for **1au** ^+^‐PCCp^–^_70%/60%/50%/40%_, respectively, were correlated with the extended inter‐sheet distances due to more effective hydration at the TEG chains. In particular, the diffraction patterns of **1au** ^+^‐PCCp^–^_50%/40%_ indicated lower crystallinity than those of **1au** ^+^‐PCCp^–^_100%/70%/60%_, suggesting phase transitions to Lam_col_ phases by hydration of the TEG chains (Figure 4b,c). The (001) diffractions at 0.66, 0.66, 0.66, and 0.65 nm for **1au** ^+^‐PCCp^–^_70%/60%/50%/40%_, respectively, suggested the formation of charge‐by‐charge assemblies without slipped stacking in the presence of water.^[^ ^51^ ^]^ Furthermore, in **1au** ^+^‐PCCp^–^_50%/40%_, the broad (010) diffractions at 0.95 and 0.98 nm, respectively, showed no tilted **1au** ^+^ core owing to the efficient hydration of a sheet structure. These observations indicated that **1au** ^+^‐PCCp^–^ in the Lam_col_ phases exhibited LCLC behaviors based on 2D organized structures via the amphiphilic *^i^π*–*^i^π* interactions between **1au** ^+^ and PCCp^–^ and the lateral hydrophobic effects with a notable contribution from the unsubstituted sites of **1au** ^+^. Increased amounts of water resulted in a less ordered arrangement of the lamellar structures, as seen in **1au** ^+^‐PCCp^–^_30%/20%_, which formed nematic sheet (labelled as N_sheet_ in this study) phases with only orientational ordering, as supported by the POM textures (Figure 4a,c; Figure S50, Supporting Information). The absence of diffraction or broadening peaks at ≈1.0 nm, corresponding to the intercolumnar distance (*b*), suggested a reduced number of repetitions and decreased ordering due to increased fluidity. **1au** ^+^‐PCCp^–^_10%/1%_ showed no POM textures at r.t. owing to the isotropically dispersed sheet structures, labeled as Iso_sheet_, with charge‐by‐charge assemblies in non‐LCLC states (Figure 4a,c; Figure S52, Supporting Information). The stacking diffractions (*c*) at 0.65 and 0.66 nm, respectively, were weak, suggesting the retention of charge‐by‐charge assemblies.

**Figure 5:**
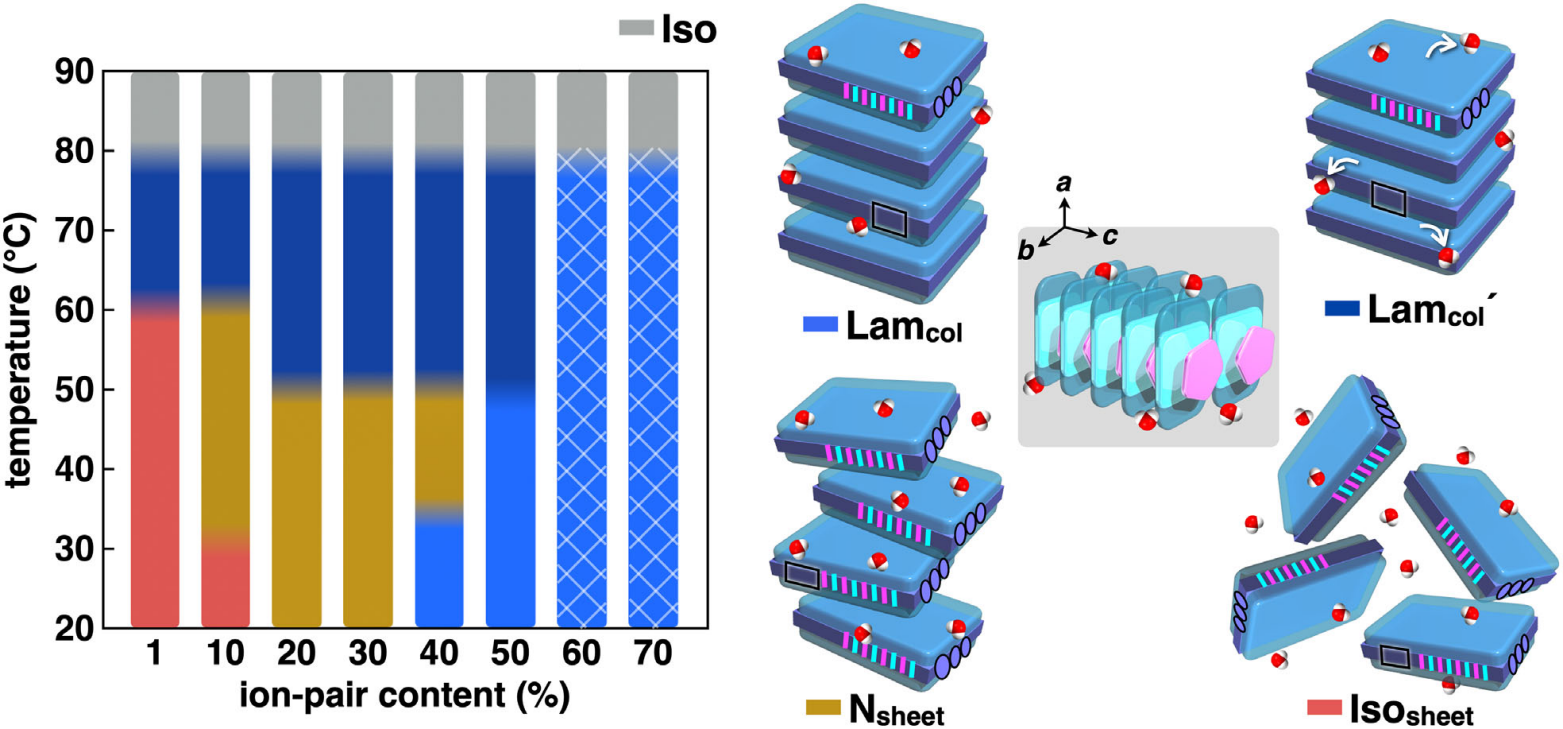
Diagram of phase changes of water‐containing **1au** ^+^‐PCCp^–^ by thermal transitions. Shaded bars show the highly ordered mesophases.

The thermal conditions and ion‐pair content influenced the assembly modes in the LCLCs of **1au** ^+^‐PCCp^–^ (**Figure** **5**).^[^ ^52^ ^]^ For example, **1au** ^+^‐PCCp^–^_40%_ in a Lam_col_ phase at r.t. showed thermal transitions at 45 and 77 °C upon heating, as revealed by DSC (Figure S29, Supporting Information). Synchrotron XRD at 25 and 60 °C upon heating showed the formation of two Lam_col_ phases (Lam_col_ and Lam_col_´ phases) with *a* values of 3.96 and 3.29 nm, *b* values of 0.98 and 1.09 nm, and *c* values of 0.65 and 0.67 nm, respectively, whereas, at 40 °C, diffraction peaks from the sheet structure without Lam_col_ pattern (*b* = 0.97 nm, *c* = 0.65 nm) were observed (Figure S96, Supporting Information). The POM texture at 40 °C suggests the formation of a N_sheet_ phase within a small temperature range (Figure S48, Supporting Information). At <45 °C, the inter‐sheet distances and ordering were mainly controlled by thermal motion. At 45 °C, the Lam_col_´ structure was formed owing to deswelling by partial dehydration upon heating. POM observation at 90 °C showed no textures, suggesting that partially hydrating TEG chains caused a phase transition to an Iso state at lower temperatures than **1au** ^+^‐PCCp^–^_100%_.

**1au** ^+^‐PCCp^–^_30%/20%_ in the N_sheet_ phases at r.t. showed thermal transitions like those of **1au** ^+^‐PCCp^–^_40%_, as seen in the transitions at 49 and 76 °C for **1au** ^+^‐PCCp^–^_20%_ (Figures S29 and S50, Supporting Information). At 60 °C, inducing dehydration, **1au** ^+^‐PCCp^–^_20%_ showed another Lam_col_ phase (Lam_col_´ phase) with *a*, *b*, and *c* values of 3.24, 0.99, and 0.66 nm, respectively, as revealed by synchrotron XRD (Figure S102, Supporting Information).^[^ ^53^ ^]^ Above 76 °C, the phase, being invisible under POM and showing no diffraction peaks, suggested a transition to an Iso state. The states with high water content, such as **1au** ^+^‐PCCp^–^_10%_ in an Iso_sheet_ phase at r.t., enhanced lateral hydrophobic effects, resulting in deswelling due to dehydration at <76 °C upon heating. **1au** ^+^‐PCCp^–^_10%_ exhibited thermal transitions at 31, 50, and 75 °C, the first of which was indicated by POM (Figures S29 and S51, Supporting Information). At 40 °C, synchrotron XRD revealed that **1au** ^+^‐PCCp^–^_10%_ exhibited a N_sheet_ phase upon dehydration, with a *c* value of 0.66 nm (Figure S104, Supporting Information). Furthermore, at 70 °C, **1au** ^+^‐PCCp^–^_10%_ showed a Lam_col_´ phase upon dehydration, with *a* and *c* values of 3.09 and 0.66 nm, respectively, as revealed by synchrotron XRD. **1au** ^+^‐PCCp^–^_1%_, showing no phase transition to a N_sheet_ phase, exhibited the phase transition from the Iso_sheet_ phase at r.t. to the Lam_col_´ phase with *a* and *c* values of 3.25 and 0.65 nm, respectively, at 60 °C (Figures S29, S52, and S106, Supporting Information). In the states with ≤50% ion‐pair content, dehydration induced enhanced ordering from 45 °C to the temperatures at which the phases were converted to Iso states. The observed thermal transitions to sheet‐based mesophases suggest a high organizing ability via lateral hydrophobic effects between amphiphilic charge‐by‐charge‐based columns. In contrast, swelling by hydration upon cooling induced phase transitions to less‐ordered phases. However, except for **1au** ^+^‐PCCp^–^_10%/1%_, which showed the difficulty in hydration upon cooling, sheet structures of different sizes and orders were formed in separated domains with different ion‐pair content.

Water‐containing **1** (**1**_70%/50%/20%_) exhibited no LCLCs, suggesting that ion pairing is necessary for the formation of ordered structures (Figures S27, S37–S39, and S79–S81, Supporting Information). Water‐containing **1au** ^+^‐Cl^–^ (**1au** ^+^‐Cl^–^_75%/70%/60%/50%/20%_) showed the LCLC behaviors (Figures S28, S40–S44, and S82–S89, Supporting Information). However, **1au** ^+^‐Cl^–^, which has no efficiently stackable anions, exhibited a high affinity for water molecules, resulting in difficulty in controlling the structures of LCLCs. **1au** ^+^‐Cl^–^_75%/70%_ formed rectangular columnar (Col_r_) phases with *a* value of 5.57 and 5.58 nm and *b* values of 1.92 and 1.94 nm, respectively, at 25 °C. At ≤60% ion‐pair content, no ordered arrangements were observed. Thus, the synergistic amphiphilic *^i^π*–*^i^π* interactions and lateral hydrophobic effects seen in **1au** ^+^‐PCCp^–^ with various ion‐pair content were found crucial for controlling the assembly modes of LCLCs. In particular, the unsubstituted parts of **1au** ^+^ aligned by charge‐by‐charge assembly cooperatively interact via lateral hydrophobic effects to form sheet‐like structures and resulting Lam_col_ phases. Synergistic amphiphilic *^i^π*–*^i^π* interactions and lateral hydrophobic effects are essential to induce ordered arrangements in multiple directions.

**2au** ^+^‐PCCp^–^, bearing HEG chains as longer hydrophilic side chains, provided different LCLC structures. In the absence of water, the elongation of the side chains led to reduced columnar alignment, resulting in charge‐by‐charge‐based columns (*c* = 0.68 nm) that were isotropically dispersed (Iso_col_) without forming organized structures (Figures S26, S36, S76, and S128, Supporting Information). On the other hand, water‐containing **2au** ^+^‐PCCp^–^_70%/60%/50%/40%_ formed hexagonal columnar (Col_h_) structures (*Z* = 1 for ρ = 0.88) with *a* and *c* values of 2.90 and 0.68 nm, respectively, for **2au** ^+^‐PCCp^–^_70%_, as an example, via amphiphilic *^i^π*–*^i^π* interactions (Figures S32, S60–S63, and S115–S122, Supporting Information). In these Col_h_ structures, HEG chains folded around the unsubstituted regions, thereby inhibiting their lateral hydrophobic effects to form sheet‐like structures. Furthermore, **2au** ^+^‐PCCp^–^_30%_ and the states with higher water content showed the formation of sheet structures and resulting Lam_col_ phases via lateral hydrophobic effects at the unsubstituted sites (Figures S32, S64–S67, and S123–S128, Supporting Information). At ≤70% ion‐pair content, thermal motion mainly controlled the inter‐sheet and intercolumnar distances and ordering below the partially dehydrated transition temperatures (61–82 °C). Above these temperatures, the partial dehydration induced deswelling, leading to Iso_col_´ states.^[^ ^54^ ^]^ For example, **2au** ^+^‐PCCp^–^_30%_ in a Lam_col_ phase (*a* = 4.07 nm, *c* = 0.67 nm) at r.t. showed thermal motions, forming the N_sheet_ phase at 50 °C upon heating (Figures S32, S64, and S123, Supporting Information). At 60 °C, the Iso_col_´ phase was formed due to deswelling by partial dehydration upon heating. Upon cooling to 50 °C, the sheet structures were hydrated, forming a Lam_col_ phase. Furthermore, at 30 °C, synchrotron XRD of **2au** ^+^‐PCCp^–^_30%_ suggested the formation of dimers proximally located at the unsubstituted sites via the partial dissociation of intercolumnar arrangements along the *b* axis driven by lateral hydrophobic effects, providing a Col_h_ phase. Tuning the relative ratio of hydrophilic to hydrophobic parts and the amount of water enabled control over the associations and dissociations in the hydrophobic regions, resulting in the formation of various assembled structures based on charge‐by‐charge stacking.

### 2.4 Macroscopically Oriented Structures in Magnetic Fields

**Figure 6:**
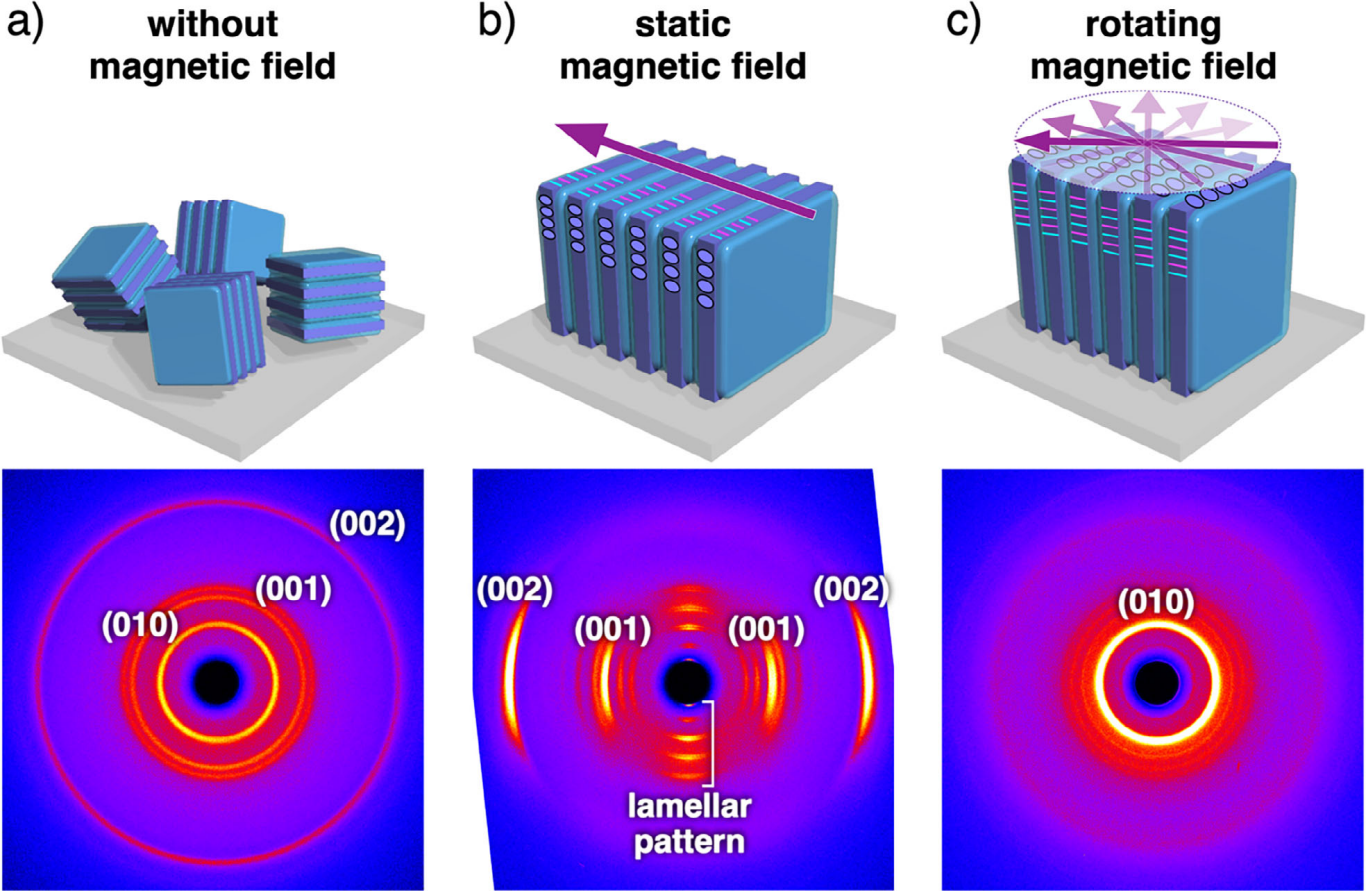
2D XRD images of the samples of **1au** ^+^‐PCCp^–^ cast on glass substrates, which were prepared by slow vaporization of MeOH from **1au** ^+^‐PCCp^–^_10%_: a) without a magnetic field, b) with a 10‐T static magnetic field applied along the glass substrate, and c) with a 10‐T magnetic field rotating in‐plane of the glass substrate.

The anisotropic magnetic susceptibility of **1au** ^+^‐PCCp^–^, along with the viscosity tunability via MeOH content control,^[^ ^55^ ^]^ would be used to induce macroscopically oriented assembled structures.^[^ ^39^, ^56^, ^57^, ^58^, ^59^ ^]^ The sample of the ion pair in MeOH exhibited superior alignment compared to that in aqueous solutions. The Lam_col_ structure fabricated under the slow condensation of **1au** ^+^‐PCCp^–^_10%_ in MeOH drop‐cast on a glass substrate by vaporizing MeOH for 14 h showed no orientation of Lam_col_ domains (**Figure** **6**a; Figure S130a, Supporting Information). In contrast, **1au** ^+^‐PCCp^–^_10%_ in MeOH in a 10‐T magnetic field applied along the glass substrate was initially less viscous and responsive to the magnetic field, followed by conversion to a viscous state that maintained the oriented structure even without the magnetic field after the drying procedure. The brightness of the POM for the dried sample changed homogeneously and drastically depending on the angular geometry, indicating that the charge‐by‐charge‐based sheet‐like structures were oriented in one direction along the glass substrate (Figure S130b, Supporting Information).^[^ ^56^ ^]^ 2D XRD revealed anisotropic (001) and (002) diffractions derived from charge‐by‐charge assemblies in the equatorial region (Figure 6b). In contrast, diffractions from a lamellar pattern with (100) and (200) diffractions were observed in the meridional region. These observations suggest that the sheet‐like structures comprising charge‐by‐charge columns were oriented with the layer normal that was parallel to the magnetic field.^[^ ^59^ ^]^ Furthermore, the 2D XRD showed no (010) diffraction, suggesting a lamellar structure perpendicular to the glass substrate and excluding the possibility of sheet‐like structures parallel to the substrate.

A magnetic field rotating in‐plane with respect to the glass substrate induces a vertical orientation of the columns in a sheet‐like structure.^[^ ^58^ ^]^ The same condensation process performed under a 10‐T magnetic field with the glass substrate rotated in‐plane at 20 rpm resulted in the orientation of the columns perpendicular to the rotation plane. The POM observations of the resulting sample exhibited slight angle‐independent birefringence, as seen in the vertically oriented columns and resulting Lam_col_ structures (Figure S130c, Supporting Information).^[^ ^57^ ^]^ In addition, the resulting sample showed almost no (001) and (002) diffractions derived from charge‐by‐charge assemblies, further supporting the vertical orientation of the columns (Figure 6c). In contrast, the (010) diffraction was the highest, suggesting strong lateral hydrophobic effects even under dynamic conditions with magnetic field‐induced alignment control. The weak (100) diffraction showed smaller domains caused by the disruption of the lamellar pattern under a magnetic field rotating within the plane of the glass substrate. The charge‐by‐charge‐based sheet‐like structure of **1au** ^+^‐PCCp^–^ can be macroscopically oriented in a single direction on demand.

### 2.5 STEM Observations of Charge‐by‐Charge‐Based Sheet‐Like Structures

**Figure 7:**
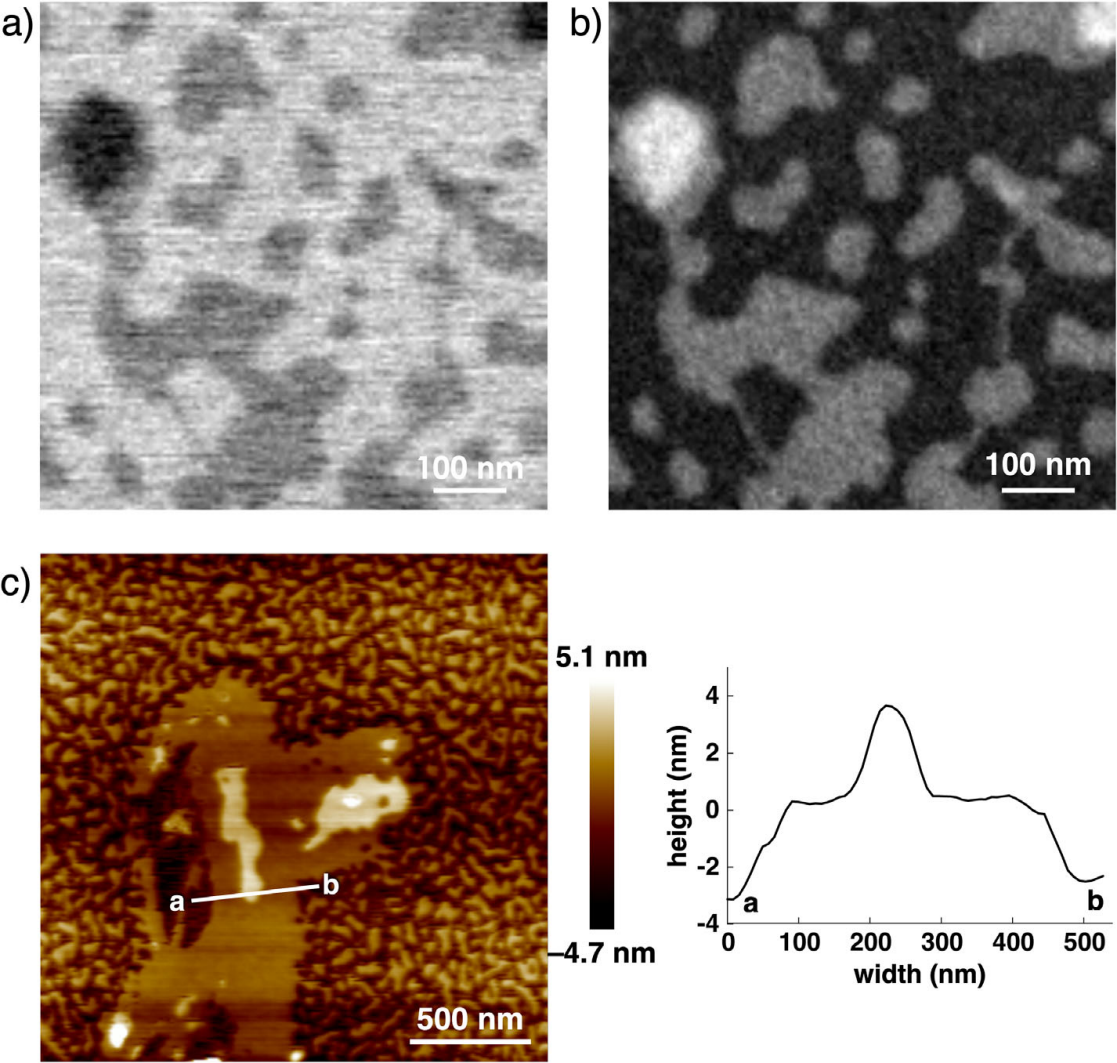
a) BF‐STEM, b) HAADF‐STEM, and c) AFM images of **1au** ^+^‐PCCp^–^ as monolayer sheet‐like structures formed in aqueous solutions (25 µm).

Amphiphilic *^i^π*–*^i^π* interactions and lateral hydrophobic effects between columns would effectively contribute to the organization even in a dilute aqueous solution. Charge‐by‐charge‐based monolayer sheet‐like structures were observed by bright‐field scanning transmission electron microscopy (BF‐STEM) in a non‐stained specimen prepared from a dilute aqueous solution of **1au** ^+^‐PCCp^–^ (25 µm) upon the deposition on a thin carbon film via freeze‐drying processes (**Figure** **7**a). This result suggests that the interactions between the sheet‐like structures at the hydrophilic substituent regions are weak compared to the amphiphilic *^i^π*–*^i^π* interactions and lateral hydrophobic effects, and charge‐by‐charge assemblies are crucial for ordered structures. The homogeneous contrast suggests that the sheet‐like structures have a uniform thickness. The high image contrast in the corresponding high‐angle annular dark‐field STEM (HAADF‐STEM) image suggests the presence of heavy Au atoms in the sheet‐like structures (Figure 7b). This observation was consistent with the STEM energy‐dispersive X‐ray spectroscopy (EDS) images obtained (Figure S131, Supporting Information). AFM observation of an aqueous solution of **1au** ^+^‐PCCp^–^ (25 µm) on a Si wafer via freeze‐drying processes showed the uniform thickness of 2.9–4.0 nm, indicating the monolayer sheet‐like structures (Figure 7c; Figure S132, Supporting Information). The direct observation of charge‐by‐charge‐based monolayer sheet‐like structures suggests that the synergistic use of amphiphilic *^i^π*–*^i^π* interactions and lateral hydrophobic effects is essential for producing multidirectionally organized structures.

## 3 Conclusion

Ion pairs of amphiphilic porphyrin Au^III^ complexes bearing hydrophilic aryl units at the 5,15‐positions were synthesized for dimension‐controlled assembly. In the PCCp^–^ ion pair of the TEG‐substituted cation, synchrotron XRD revealed the formation of thermotropic liquid crystals comprising sheet‐like structures arranged in a charge‐by‐charge assembly via amphiphilic *^i^π*–*^i^π* interactions. The proximal locations at the unsubstituted sites of the porphyrin Au^III^ complex contributed to the formation of Lam_col_ phases, which were characterized by highly ordered arrangements. In the water‐containing states, charge‐by‐charge‐based Lam_col_, N_sheet_, and Iso_sheet_ phases were constructed depending on the water amount and temperature, exhibiting LCLC behaviors for the first two phases. Unlike in crystalline solids, the ordering along the directions via amphiphilic *^i^π*–*^i^π* interactions, lateral hydrophobic effects, and hydrophilic interactions are not mutually correlated in the studied LCLCs. Furthermore, the charge‐by‐charge‐based sheet‐like structure in the Lam_col_ phase was unidirectionally oriented after drying under a magnetic field. Charge‐by‐charge‐based monolayer sheet‐like structures were observed as the components of LCLCs using STEM and AFM from a diluted aqueous solution via freeze‐drying. These observations suggest that amphiphilic charged π‐electronic systems were multidirectionally organized via amphiphilic *^i^π*–*^i^π* interactions and lateral hydrophobic effects between the charge‐by‐charge‐based columns. The relative ratio of hydrophilic and hydrophobic regions is essential for constructing 2D organized structures. Ion‐pairing‐based organization without substituents involved in directional interactions can facilitate the control of assembly modes and properties by introducing hydrophobic substituents and other counterions. Charge‐by‐charge columnar assemblies and their proximal locations would provide 2D anisotropic functional materials, some of which may exhibit stimuli‐responsive ferroelectric properties derived from *π‐sip*‐based supramolecular dipoles.

## Conflict of Interest

The authors declare no conflict of interest.

## Supporting information



Supporting Information

## Data Availability

The data that support the findings of this study are available in the supplementary material of this article.
