# Supplementary material for: Multidirectionally Controlled Arrangement via Ion‐Pairing Assembly of Amphiphilic Charged π‐Electronic Systems
Source: Small. 2025 Nov 25;22(3):e11729. doi: 10.1002/smll.202511729 (PMC12802529; doi:10.1002/smll.202511729)
Supplement: Supplementary file 1 — Supporting Information [file SMLL-22-e11729-s001.pdf]

NANO · MICRO
small

Supporting Information

for *Small*, DOI 10.1002/smll.202511729

Multidirectionally Controlled Arrangement via Ion-Pairing Assembly of Amphiphilic Charged π-Electronic Systems

*Yuto Maruyama, Biplab Manna, Koji Harano, Hayato Kanai, Yasuhiro Ishida and Hiromitsu Maeda**

Supporting Information

Multidirectionally Controlled Arrangement via Ion-Pairing Assembly of Amphiphilic Charged π-Electronic Systems

Yuto Maruyama, Biplab Manna, Koji Harano, Hayato Kanai, Yasuhiro Ishida, and Hiromitsu Maeda*

Department of Applied Chemistry, College of Life Sciences, Ritsumeikan University, Kusatsu 525–8577, Japan, E-mail: maedahir@ph.ritsumei.ac.jp, Center for Basic Research on Materials, National Institute for Materials Science, Tsukuba 305–0044, Japan, Research Center for Autonomous Systems Materialogy (ASMat), Institute of Integrated Research, Institute of Science Tokyo, Yokohama 226–8501, Japan, and Center for Emergent Matter Science (CEMS), RIKEN, Wako 351–0198, Japan

Table of Contents

1. Synthetic procedures and spectroscopic data S2
Figure S1 Synthesis of amphiphilic porphyrin Au^{III} complex ion pairs. S2
Figure S2–11 ^{1}H and ^{13}C NMR spectra. S5
2. Theoretical studies S13
Figure S12 Optimized structure. S13
Figure S13 Electrostatic potential (ESP) mapping. S13
Figure S14 Molecular orbitals. S13
Figure S15 TD-DFT calculations. S15
Cartesian coordination of optimized structures. S15
3. Examination of organized structures S19
Figure S16–19 UV/vis absorption spectra. S20
Figure S20–25 Dynamic light scattering. S22
Figure S26–32 DSC thermographs. S29
Figure S33–67 POM images. S36
Figure S68–128 XRD and packing diagrams. S51
Figure S129 UV/vis absorption spectra of thermotropic liquid crystals. S153
Figure S130 XRD and POM images of the aligned thin film under magnetic field. S154
Figure S131 STEM images. S156
Figure S132 AFM image. S156

1. Synthetic procedures and spectroscopic data

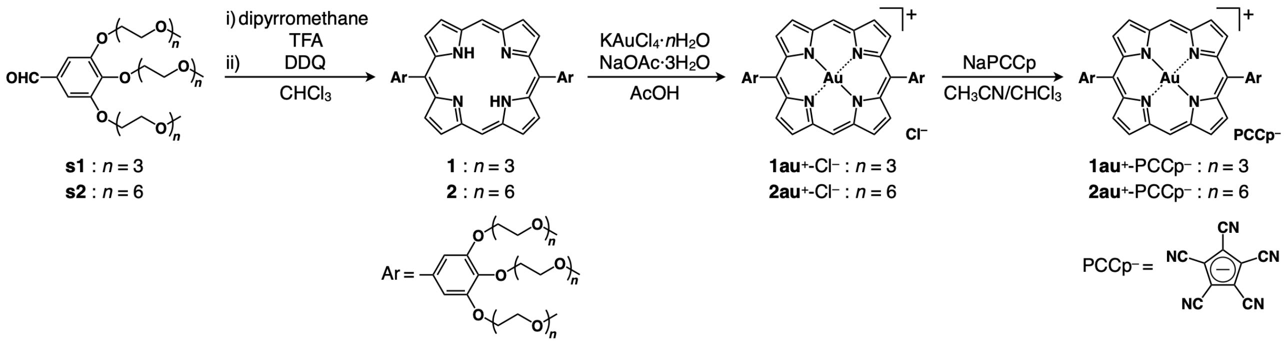

Figure S1 Synthesis of amphiphilic porphyrin Au^{III} complex ion pairs.

General procedures. Starting materials were purchased from FUJIFILM Wako Pure Chemical Corp., Nacalai Tesque Inc., Tokyo Chemical Industry Co., Ltd., and Sigma-Aldrich Co. and were used without further purification unless otherwise stated. 5,15-Bis(3,4,5-tri(2-(2-(2-methoxyethoxy)ethoxy)ethoxy)phenyl)-porphyrin **1** was synthesized according to the literature procedure.[S1] NMR spectra used in the characterization of products were recorded on a JEOL ECA-600 600 MHz spectrometer. ^{1}H and ^{13}C NMR spectra were referenced to solvent. UV-visible absorption spectra were recorded on a Hitachi U-4100 spectrometer. High-resolution (HR) electrospray ionization mass spectrometry (ESI-MS) was recorded on a BRUKER microTOF using ESI-TOF method. TLC analyses were carried out on aluminum sheets coated with silica gel 60 (Merck 5554). Column chromatography was performed on Wakogel C-300.

3,4,5-Tris(2-(2-(2-(2-(2-(2-methoxyethoxy)ethoxy)-ethoxy)ethoxy)ethoxy)ethoxy)benzaldehyde, s2. To a solution of 3,4,5-trihydroxybenzaldehyde (0.46 g, 3.0 mmol) and tosylated hexaethylene glycol monomethyl ether (4.5 g, 10 mmol) in DMF (20 mL) was added K_2CO_3 (2.7 g, 20 mmol). The reaction was left stirring at 100 °C for 3 days, cooled to r.t., and was poured into a 2 M aqueous solution of H_2SO_4 (77 mL). The mixture was extracted with CH_2Cl_2 and the combined organic phases were washed with brine. The organic phase was dried over Na_2SO_4 and the solvent removed in vacuo leaving the crude product, which was purified using as a silica gel column (Wakogel C-300, eluent: 5% MeOH/CH_2Cl_2) to yield **s2** (0.87 g, 0.880 mmol, 29%) as a light yellow oil. R_f = 0.25 (5% MeOH/CH_2Cl_2). ^{1}H NMR (600 MHz, $CDCl_3$, 20 °C): δ (ppm): 9.82 (s, 1H, CHO), 7.14 (s, 2H, Ar-H), 4.26 (t, J = 5.4 Hz, 2H, OCH_2), 4.22 (t, J = 4.8 Hz, 4H, OCH_2), 3.87 (t, J = 4.8 Hz, 4H, $OCH_2\underline{CH_2}$), 3.80 (m, 2H, $OCH_2\underline{CH_2}$), 3.72–3.69 (m, 6H, $O(CH_2)_2O\underline{CH_2}$), 3.66–3.62 (m, 48H, $O(CH_2)_2OCH_2\underline{CH_2}$ + $\{O(CH_2)_2\}_2O\underline{CH_2}$ + $\{O(CH_2)_2\}_2OCH_2\underline{CH_2}$ + $\{O(CH_2)_2\}_3O\underline{CH_2}$ + $\{O(CH_2)_2\}_3OCH_2\underline{CH_2}$ + $\{O(CH_2)_2\}_4O\underline{CH_2}$ + $\{O(CH_2)_2\}_4OCH_2\underline{CH_2}$ + $\{O(CH_2)_2\}_5O\underline{CH_2}$), 3.55–3.54 (m, 6H, $\{O(CH_2)_2\}_5OCH_2\underline{CH_2}$)), 3.38 (s, 9H, $\{O(CH_2)_2\}_6O\underline{CH_3}$). ^{13}C{^{1}H} NMR (151 MHz, $CDCl_3$, 20 °C): δ(ppm) 191.08, 153.11, 144.16, 131.70, 109.08, 72.78, 72.62, 72.02, 70.91, 70.74, 70.71, 70.68, 70.65, 70.58, 70.34, 69.70, 69.04, 61.78, 59.09 (several signals for HEG chains were overlapped). HRMS (ESI-TOF): m/z: calcd for $C_{46}H_{84}O_{22}$ ([M + H]$^+$): 989.5527; found 989.5527.

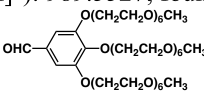

5,15-Bis(3,4,5-tris(2-(2-(2-(2-(2-(2-methoxyethoxy)-ethoxy)ethoxy)ethoxy)ethoxy)ethoxy)phenyl)porphyrin, 2. According to the modified literature method,[S1] to the solution of **s2** (868.8 g, 0.878 mmol) in $CHCl_3$ (175 mL), deaerated by bubbling with N_2 for 20 min, was added dipyrromethane[S2] (128.3 mg, 0.878 mmol). The mixture was stirred with TFA (0.89 mL, 7.55 mmol) at r.t. in the dark for 14 h. To the reaction mixture was added 2,3-dichloro-5,6-dicyano-1,4-benzoquinone (DDQ) (200.7 mg, 0.88 mmol) as the oxidant, followed by stirring for 3.5 h. The reaction mixture was washed successively with aqueous saturated $NaHCO_3$ solution and brine. The organic layer separated was dried over anhydrous Na_2SO_4. The residue was then chromatographed over a silica gel column (Wakogel C-300, eluent: 7% and 4% MeOH/CH_2Cl_2) to provide **2** (369 mg, 0.166 mmol, 38%) as a viscous purple substance. R_f = 0.32 (7% MeOH/$CHCl_3$). ^{1}H NMR (600 MHz, $CDCl_3$, 20 °C): δ (ppm): 10.31 (s, 2H, *meso*-H), 9.39 (d, J = 4.2 Hz, 4H, β-H), 9.14 (d, J = 4.8 Hz, 4H, β-H), 7.53 (s, 4H, Ar-H), 4.52 (t, J = 5.4 Hz, 4H, OCH_2), 4.34 (t, J = 4.8 Hz, 8H, OCH_2), 4.05 (t, J = 4.8 Hz, 4H, $OCH_2\underline{CH_2}$), 3.94 (t, J = 4.8 Hz, 8H, $OCH_2\underline{CH_2}$), 3.91–3.89 (m, 4H, $(CH_2)_2O\underline{CH_2}$), 3.80–3.78 (m, 4H, $O(CH_2)_2OCH_2\underline{CH_2}$), 3.76–3.72 (m, 16H, $O(CH_2)_2O\underline{CH_2}$ + $O(CH_2)_2OCH_2\underline{CH_2}$), 3.69–3.62 (m, 28H, $\{O(CH_2)_2\}_2O\underline{CH_2}$ + $\{O(CH_2)_2\}_2OCH_2\underline{CH_2}$ + $\{O(CH_2)_2\}_3O\underline{CH_2}$), 3.56–3.52 (m, 36H, $\{O(CH_2)_2\}_3O\underline{CH_2}$ + $\{O(CH_2)_2\}_3OCH_2\underline{CH_2}$ + $\{O(CH_2)_2\}_4O\underline{CH_2}$ + $\{O(CH_2)_2\}_4OCH_2\underline{CH_2}$), 3.49–3.44 (m, 32H, $\{O(CH_2)_2\}_4OCH_2\underline{CH_2}$ + $\{O(CH_2)_2\}_5O\underline{CH_3}$ + $\{O(CH_2)_2\}_5OCH_2\underline{CH_2}$), 3.38 (s, 6H, $\{O(CH_2)_2\}_6O\underline{CH_3}$), 3.30 (s, 12H, $\{O(CH_2)_2\}_6O\underline{CH_3}$), –3.16 (s, 2H, NH).

$^{13}C\{^1H\}$ NMR (151 MHz, $CDCl_3$, 20 °C): δ(ppm) 151.01, 147.01, 145.18, 138.31, 136.58, 131.60, 130.95, 118.66, 115.38, 105.27, 72.71, 71.87, 70.78, 70.74, 70.61, 70.55, 70.52, 70.45, 70.41, 70.36, 70.33, 69.81, 69.09, 58.95, 58.87 (several signals for HEG chains were overlapped). UV/vis (CH_2Cl_2, λ_{max}[nm] (ε, 10^5 $M^{-1}cm^{-1}$)): 410 (3.5), 503 (0.18). HRMS (ESI-TOF): *m/z*: calcd for $C_{110}H_{178}N_4O_{42}$ ([M + H]$^+$): 2228.1988; found 2228.1988.

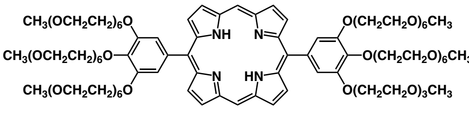

Au^{III} complex of 1 as a Cl$^-$ ion pair, 1au$^+$-Cl$^-$. According to the literature procedure,[S3] a solution of $KAuCl_4{\cdot}nH_2O$ (38.3 mg, 0.10 mmol) and $NaOAc{\cdot}3H_2O$ (68.4 mg, 0.50 mmol) in AcOH (4.0 mL) was heated at 80 °C for 15 min. A solution of **1** (117 mg, 0.082 mmol) in AcOH (4.5 mL) was added dropwise. The mixture was heated under reflux for 2 h. After the removal of solvent by vacuum, the residue was dissolved in CH_2Cl_2. The CH_2Cl_2 solution was washed with water and brine and was evaporated to dryness. The residue was then chromatographed over a silica gel column (Wakogel C-300, eluent: 10% and 5% MeOH/CH_2Cl_2) and was recrystallized from $CHCl_3$/*n*-hexane to give **1au**$^+$-Cl$^-$ (32.5 mg, 0.019 mmol, 24%) as a red solid. R_f = 0.34 (10% MeOH/CH_2Cl_2). ^1H NMR (600 MHz, $CDCl_3$, 20 °C): δ(ppm): 11.47 (s, 2H, *meso*-H), 10.05 (d, *J* = 5.4 Hz, 4H, β-H), 9.56 (d, *J* = 4.8 Hz, 4H, β-H), 7.52 (s, 4H, Ar-H), 4.55 (t, *J* = 5.4 Hz, 4H, OCH_2), 4.33 (t, *J* = 4.8 Hz, 8H, OCH_2), 4.07–4.05 (m, 4H, $OCH_2\underline{CH_2}$), 3.95 (t, *J* = 4.8 Hz, 8H, $OCH_2\underline{CH_2}$), 3.92–3.91 (m, 4H, $(CH_2)_2O\underline{CH_2}$), 3.81–3.80 (m, 4H, $O(CH_2)_2OCH_2\underline{CH_2}$), 3.77–3.75 (m, 12H, $O(CH_2)_2O\underline{CH_2}$ + $\{O(CH_2)_2\}_2O\underline{CH_2}$), 3.65–3.63 (m, 12H, $O(CH_2)_2OCH_2\underline{CH_2}$ + $\{O(CH_2)_2\}_2OCH_2\underline{CH_2}$), 3.56–3.55 (m, 8H, $\{O(CH_2)_2\}_2O\underline{CH_2}$), 3.44–3.43 (m, 14H, $\{O(CH_2)_2\}_2OCH_2\underline{CH_2}$ + $\{O(CH_2)_2\}_3O\underline{CH_3}$), 3.25 (s, 12H, $\{O(CH_2)_2\}_3O\underline{CH_3}$). $^{13}C\{^1H\}$ NMR (151 MHz, $CDCl_3$, 20 °C): δ(ppm) 151.85, 139.67, 136.63, 136.36, 133.41, 133.24, 132.75, 122.28, 115.20, 109.77, 73.01, 72.15, 71.93, 70.98, 70.96, 70.84, 70.77, 70.58, 69.90, 69.48, 59.24, 59.05 (several signals for TEG chains were overlapped). UV/vis (CH_2Cl_2, λ_{max}[nm] (ε, 10^5 $M^{-1}cm^{-1}$)): 398 (1.2), 513 (0.16). HRMS (ESI-TOF): *m/z*: calcd for $C_{74}H_{104}AuN_4O_{24}$ ([M – Cl]$^+$): 1629.6701; found 1629.6701.

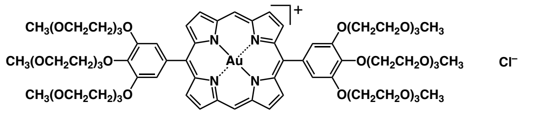

Au^{III} complex of 2 as a Cl$^-$ ion pair, 2au$^+$-Cl$^-$. According to the literature procedure,[S3] a solution of $KAuCl_4{\cdot}nH_2O$ (32.1 mg, 0.085 mmol) and $NaOAc{\cdot}3H_2O$ (53.4 mg, 0.392 mmol) in AcOH (3.4 mL) was heated at 80 °C for 15 min. A solution of **2** (147.9 mg, 0.066 mmol) in AcOH (4.4 mL) was added dropwise. The mixture was heated under reflux for 2 h. After the removal of solvent by vacuum, the residue was dissolved in $CHCl_3$. The $CHCl_3$ solution was washed with water and brine and was evaporated to dryness. The residue was then chromatographed over a silica gel column (Wakogel C-300, eluent: 10% and 13% MeOH/$CHCl_3$) and was recrystallized from $CHCl_3$/*n*-hexane to give **2au**$^+$-Cl$^-$ (43.1 mg, 0.0175 mmol, 26%) as a red solid. R_f = 0.26 (13% MeOH/$CHCl_3$). ^1H NMR (600 MHz, $CDCl_3$, 20 °C): δ(ppm): 11.46 (s, 2H, *meso*-H), 10.04 (d, *J* = 4.8 Hz, 4H, β-H), 9.54 (d, *J* = 4.8 Hz, 4H, β-H), 7.50 (s, 4H, Ar-H), 4.53 (t, *J* = 4.8 Hz, 4H, OCH_2), 4.32 (t, *J* = 4.8 Hz, 8H, OCH_2), 4.03 (t, *J* = 4.8 Hz, 4H, $OCH_2\underline{CH_2}$), 3.93 (t, *J* = 4.8 Hz, 8H, $OCH_2\underline{CH_2}$), 3.89–3.87 (m, 4H, $(CH_2)_2O\underline{CH_2}$), 3.78–3.77 (m, 4H, $O(CH_2)_2OCH_2\underline{CH_2}$), 3.74–3.70 (m, 16H, $O(CH_2)_2O\underline{CH_2}$ + $O(CH_2)_2OCH_2\underline{CH_2}$), 3.69–3.61 (m, 28H, $\{O(CH_2)_2\}_2O\underline{CH_2}$ + $\{O(CH_2)_2\}_2OCH_2\underline{CH_2}$ + $\{O(CH_2)_2\}_3O\underline{CH_2}$), 3.57–3.52 (m, 60H, $\{O(CH_2)_2\}_3O\underline{CH_2}$ + $\{O(CH_2)_2\}_3OCH_2\underline{CH_2}$ + $\{O(CH_2)_2\}_4O\underline{CH_2}$ + $\{O(CH_2)_2\}_4OCH_2\underline{CH_2}$ + $\{O(CH_2)_2\}_5O\underline{CH_2}$ + $\{O(CH_2)_2\}_5OCH_2\underline{CH_2}$), 3.48–3.46 (m, 8H, $\{O(CH_2)_2\}_5OCH_2\underline{CH_2}$), 3.36 (s, 6H, $\{O(CH_2)_2\}_6O\underline{CH_3}$), 3.30 (s, 12H, $\{O(CH_2)_2\}_6O\underline{CH_3}$). $^{13}C\{^1H\}$ NMR (151 MHz, $CDCl_3$, 20 °C): δ(ppm) 152.04, 139.88, 136.85, 136.58, 133.64, 133.44, 132.96, 122.48, 115.43, 109.95, 73.19, 72.25, 72.18, 71.14, 71.10, 71.02, 70.98, 70.95, 70.92, 70.87, 70.82, 70.78, 70.75, 70.09, 69.65, 59.34, 59.27 (several signals for HEG chains were overlapped). UV/vis (CH_2Cl_2, λ_{max}[nm] (ε, 10^5 $M^{-1}cm^{-1}$)): 399 (1.3), 513 (0.18). HRMS (ESI-TOF): *m/z*: calcd for $C_{110}H_{176}AuN_4O_{42}$ ([M – Cl]$^+$): 2422.1419; found 2422.1419.

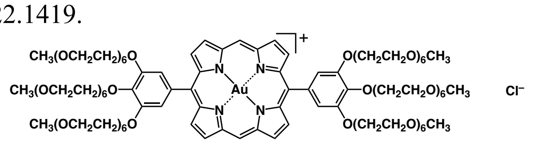

General preparation protocol for anion exchanges from Cl$^-$ to other anions. A solution of Na$^+$ salt of anions (3 equiv) in CH_3CN was added to a solution of Cl$^-$ salts of porphyrin–Au^{III} complexes in CH_3CN/$CHCl_3$ and the mixture was stirred for a few minutes. The resulting precipitates were collected and was washed with CH_2Cl_2 and water. After confirming that no precipitate formed, the products were filtered and the solvent was evaporated. The residue was then chromatographed over a silica gel column and was recrystallized from suitable solvents afforded ion pairs as solid materials. The obtained ion pairs were characterized by ^1H and ^{13}C NMR. The details for each ion pair are described as below.

Au^{III} complex 1au$^+$ as a PCCp$^-$ ion pair, 1au$^+$-PCCp$^-$. Sodium pentacyanocyclopentadienide (NaPCCp)[S4] was used for anion exchange. After the workup, the residue was purified by chromatography over a silica gel column (Wakogel C-300, eluent: 4% MeOH/$CHCl_3$) and was recrystallized from $CHCl_3$/*n*-hexane to give **1au**$^+$-PCCp$^-$ (39.5 mg, 0.022 mmol, 73%) as a red solid. R_f = 0.16 (4% MeOH/$CHCl_3$). ^1H NMR (600 MHz, $CDCl_3$, 20 °C): δ(ppm): 11.07 (s, 2H, *meso*-H), 9.86 (d, *J* = 4.8 Hz, 4H, β-H), 9.67 (d, *J* = 5.4 Hz, 4H, β-H), 7.69 (s, 4H, Ar-H), 4.56 (t, *J* = 5.4 Hz, 4H, OCH_2), 4.36–4.34 (m, 8H, OCH_2), 4.08–4.06 (m, 4H, $OCH_2\underline{CH_2}$), 3.95–3.92 (m, 12H, $OCH_2\underline{CH_2}$ + $O(CH_2)_2O\underline{CH_2}$), 3.82–3.80 (m, 4H,

$O(CH_2)_2OCH_2\underline{CH_2}$), 3.78–3.75 (m, 12H, $O(CH_2)_2O\underline{CH_2}$ + $\{O(CH_2)_2\}_2O\underline{CH_2}$), 3.67–3.63 (m, 12H, $O(CH_2)_2OCH_2\underline{CH_2}$ + $\{O(CH_2)_2\}_2OCH_2\underline{CH_2}$), 3.60–3.59 (m, 8H, $\{O(CH_2)_2\}_2O\underline{CH_2}$), 3.49–3.47 (m, 8H, $\{O(CH_2)_2\}_2OCH_2\underline{CH_2}$), 3.44 (s, 6H, $\{O(CH_2)_2\}_3O\underline{CH_3}$), 3.29 (s, 12H, $\{O(CH_2)_2\}_3O\underline{CH_3}$). $^{13}C\{^1H\}$ NMR (151 MHz, $CDCl_3$, 20 °C): δ (ppm) 151.72, 139.51, 136.84, 136.12, 133.44, 133.30, 132.45, 122.96, 115.38, 110.12, 108.80, 98.57, 72.98, 72.17, 72.01, 70.98, 70.93, 70.84, 70.81, 70.77, 70.62, 69.91, 69.38, 59.23, 59.08 (several signals for TEG chains were overlapped). UV/vis (CH_2Cl_2, λ_{max}[nm] (ε, 10^5 $M^{-1}cm^{-1}$)): 398 (1.1), 513 (0.15). HRMS (ESI-TOF): *m/z*: calcd for $C_{74}H_{104}AuN_4O_{24}$ ([M – $C_{10}N_5$]$^+$): 1629.6701; found 1629.6701. Calcd for $C_{10}N_5$ ([M – **1au**]$^-$): 190.0159; found 190.0159.

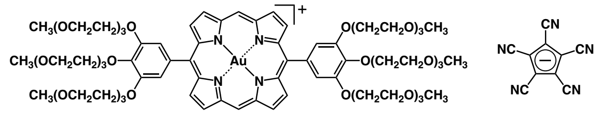

Au^{III} complex 2au$^+$ as a PCCp$^-$ ion pair, 2au$^+$-PCCp$^-$. NaPCCp[S4] was used for anion exchange. After the workup, the residue was purified by chromatography over a silica gel column (Wakogel C-300, eluent: 7% MeOH/$CHCl_3$) to give **2au**$^+$-PCCp$^-$ (42.7 mg, 0.016 mmol, 93%) as a red oil. R_f = 0.30 (7% MeOH/$CHCl_3$). ^1H NMR (600 MHz, $CDCl_3$, 20 °C): δ (ppm): 11.10 (s, 2H, *meso*-H), 9.87 (d, J = 5.4 Hz, 4H, β-H), 9.66 (d, J = 4.8 Hz, 4H, β-H), 7.69 (s, 4H, Ar-H), 4.55 (t, J = 4.8 Hz, 4H, OCH_2), 4.35 (t, J = 4.8 Hz, 8H, OCH_2), 4.06 (t, J = 4.8 Hz, 4H, $OCH_2\underline{CH_2}$), 3.94 (t, J = 4.8 Hz, 8H, $OCH_2\underline{CH_2}$), 3.91–3.90 (m, 4H, $(CH_2)_2O\underline{CH_2}$), 3.81–3.79 (m, 4H, $O(CH_2)_2OCH_2\underline{CH_2}$), 3.77–3.73 (m, 16H, $O(CH_2)_2O\underline{CH_2}$ + $O(CH_2)_2OCH_2\underline{CH_2}$), 3.71–3.64 (m, 28H, $\{O(CH_2)_2\}_2O\underline{CH_2}$ + $\{O(CH_2)_2\}_2OCH_2\underline{CH_2}$ + $\{O(CH_2)_2\}_3O\underline{CH_2}$), 3.61–3.56 (m, 60H, $\{O(CH_2)_2\}_3O\underline{CH_2}$ + $\{O(CH_2)_2\}_3OCH_2\underline{CH_2}$ + $\{O(CH_2)_2\}_4O\underline{CH_2}$ + $\{O(CH_2)_2\}_4OCH_2\underline{CH_2}$ + $\{O(CH_2)_2\}_5O\underline{CH_2}$ + $\{O(CH_2)_2\}_5OCH_2\underline{CH_2}$), 3.52–3.50 (m, 8H, $\{O(CH_2)_2\}_5OCH_2\underline{CH_2}$), 3.39 (s, 6H, $\{O(CH_2)_2\}_6O\underline{CH_3}$), 3.34 (s, 12H, $\{O(CH_2)_2\}_6O\underline{CH_3}$). $^{13}C\{^1H\}$ NMR (151 MHz, $CDCl_3$, 20 °C): δ(ppm) 151.86, 139.51, 137.04, 136.36, 133.61, 132.74, 130.32, 123.10, 115.44, 110.44, 109.09, 98.90, 73.12, 72.24, 72.18, 71.15, 71.06, 70.96, 70.91, 70.83, 70.88, 70.79, 70.75, 70.07, 69.47, 59.36, 59.30 (several signals for HEG chains were overlapped). UV/vis (CH_2Cl_2, λ_{max}[nm] (ε, 10^5 $M^{-1}cm^{-1}$)): 399 (1.2), 513 (0.16). HRMS (ESI-TOF): *m/z*: calcd for $C_{110}H_{176}AuN_4O_{42}$ ([M – $C_{10}N_5$]$^+$): 2422.1419; found 2422.1419. Calcd for $C_{10}N_5$ ([M – **2au**]$^-$): 190.0159; found 190.0159.

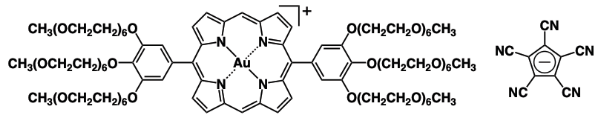

[S1] T. Morita, Y. Kuroda, M. Morisue, *Org. Biomol. Chem.* **2010**, *8*, 3457–3463.

[S2] K. Lu, C. He, W. Lin, *J. Am. Chem. Soc.* **2014**, *136*, 16712–16715.

[S3] Y. Maruyama, K. Harano, H. Kanai, Y. Ishida, H. Tanaka, S. Sugiura, H. Maeda, *Angew. Chem. Int. Ed.* **2025**, *64*, e202415135.

[S4] a) O. W. Webster, *J. Am. Chem. Soc.* **1965**, *87*, 1820–1821; b) T. Sakai, S. Seo, J. Matsuoka, Y. Mori, *J. Org. Chem.* **2013**, *78*, 10978–10985.

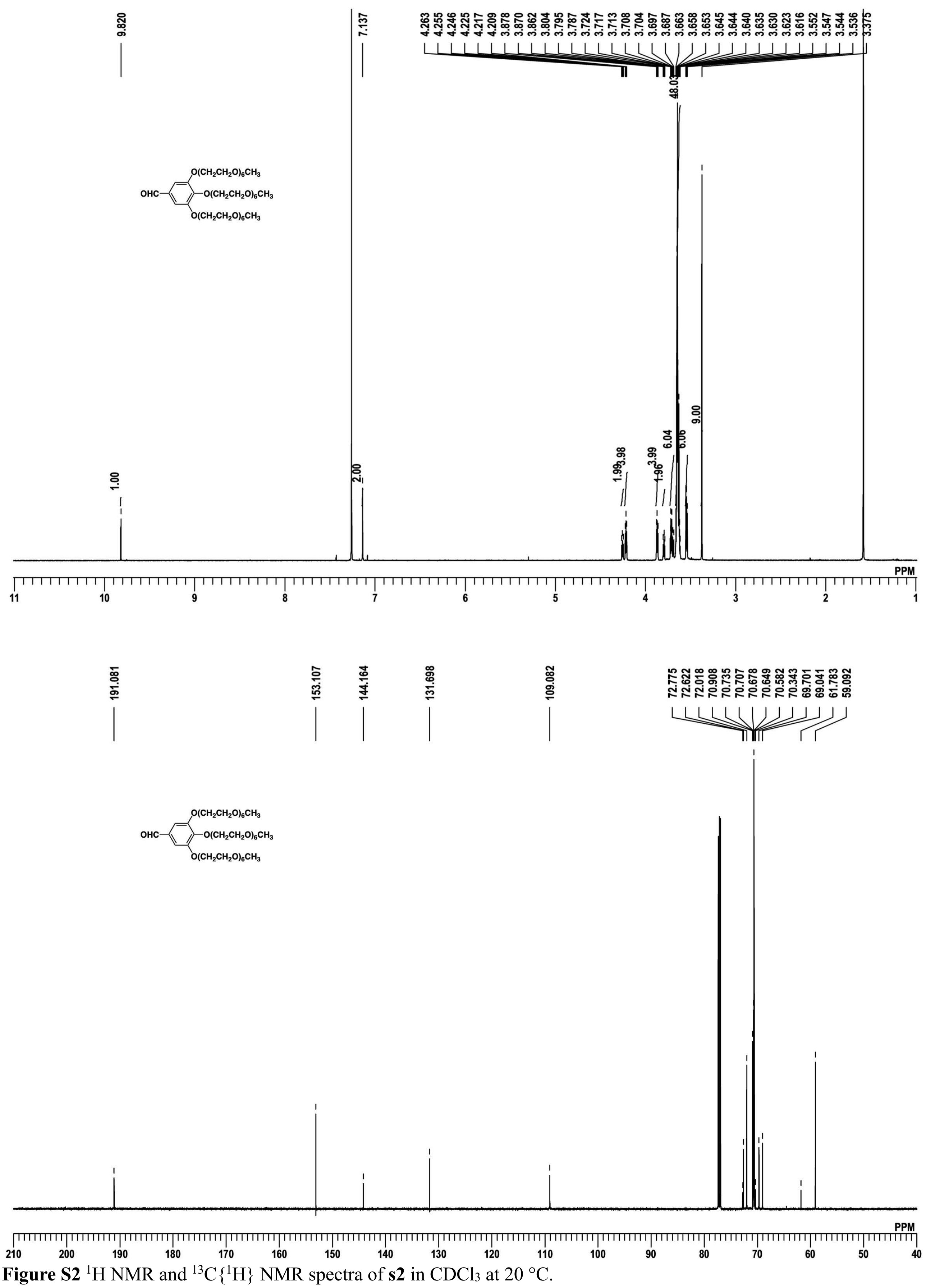

Figure S2 ^{1}H NMR and $^{13}C\{^{1}H\}$ NMR spectra of **s2** in $CDCl_3$ at 20 °C.

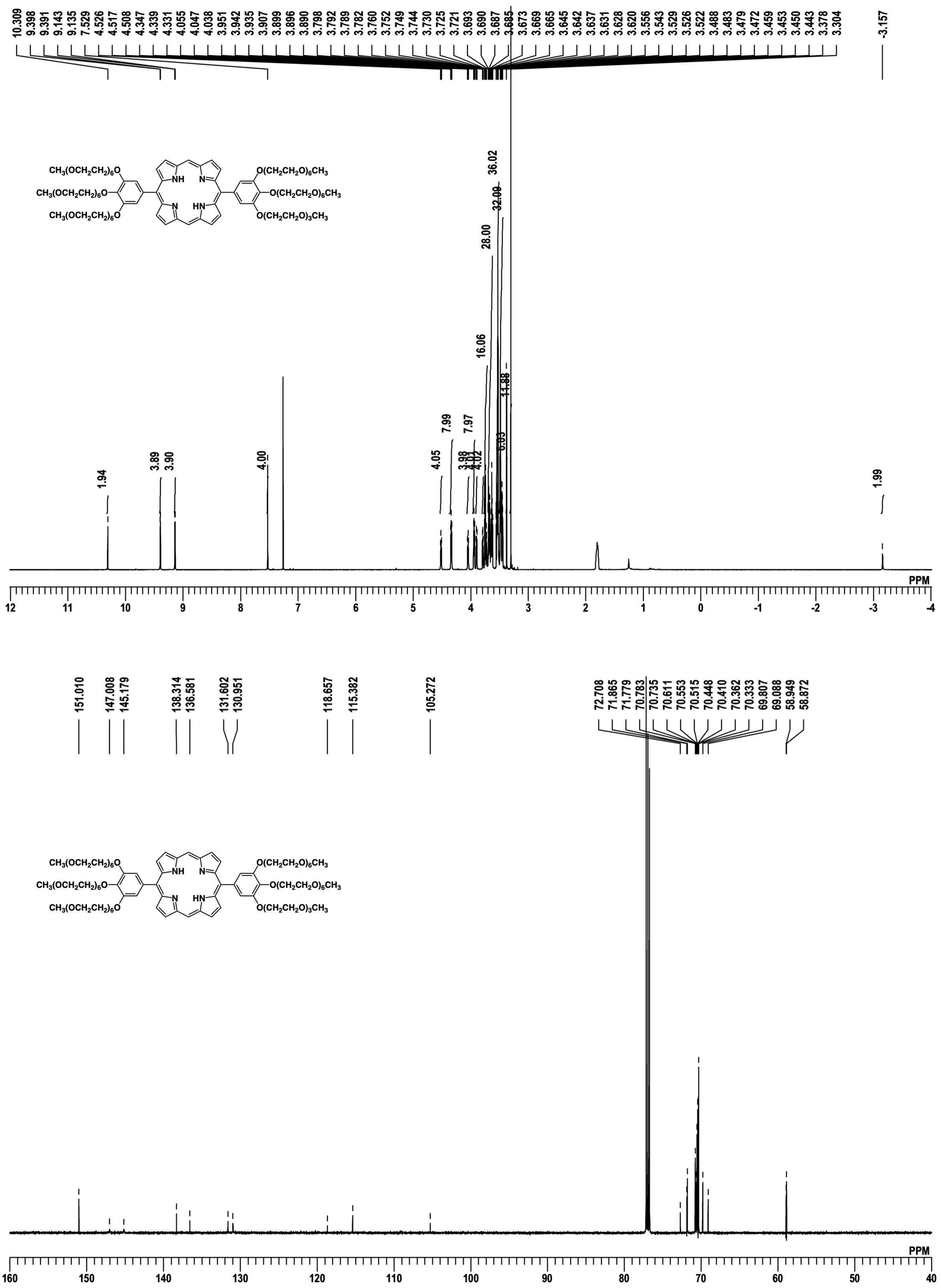

Figure S3 ^{1}H NMR and $^{13}C\{^{1}H\}$ NMR spectra of **2** in $CDCl_3$ at 20 °C.

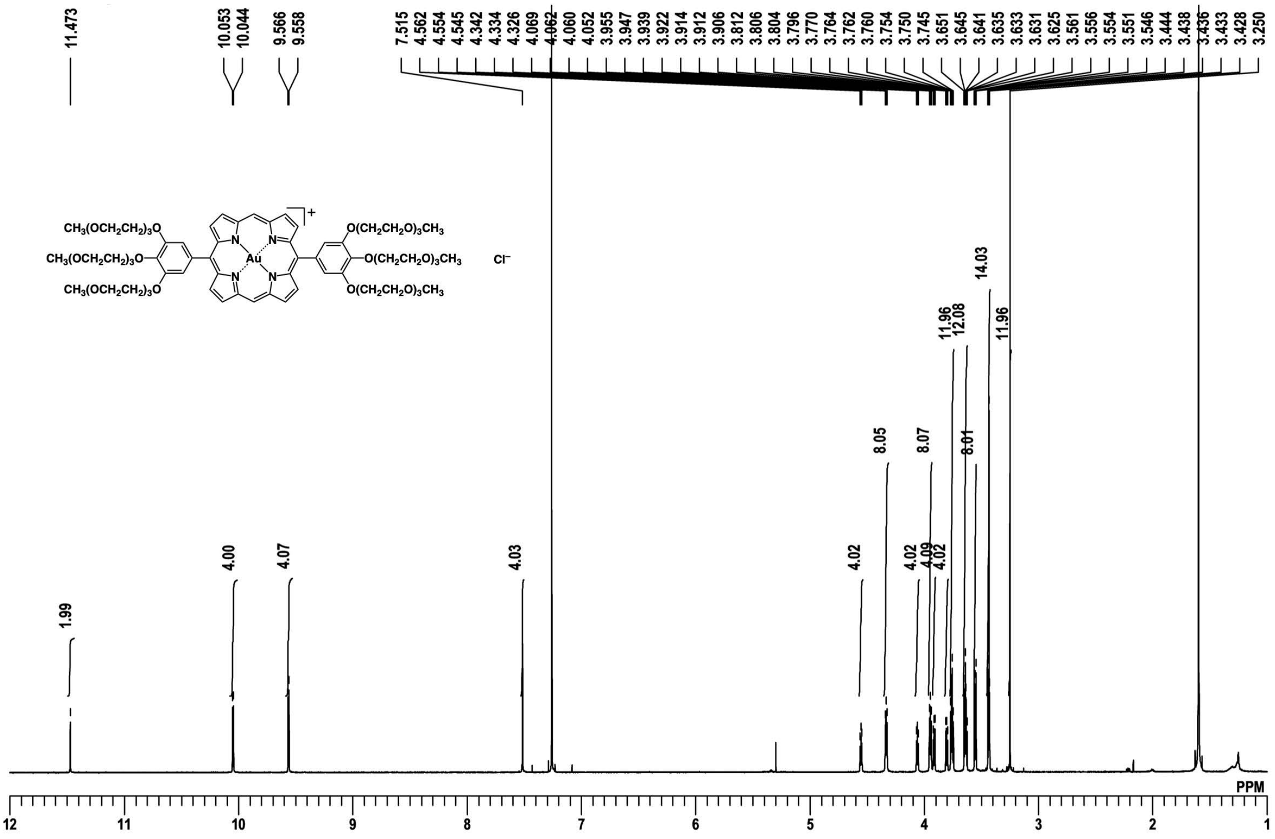

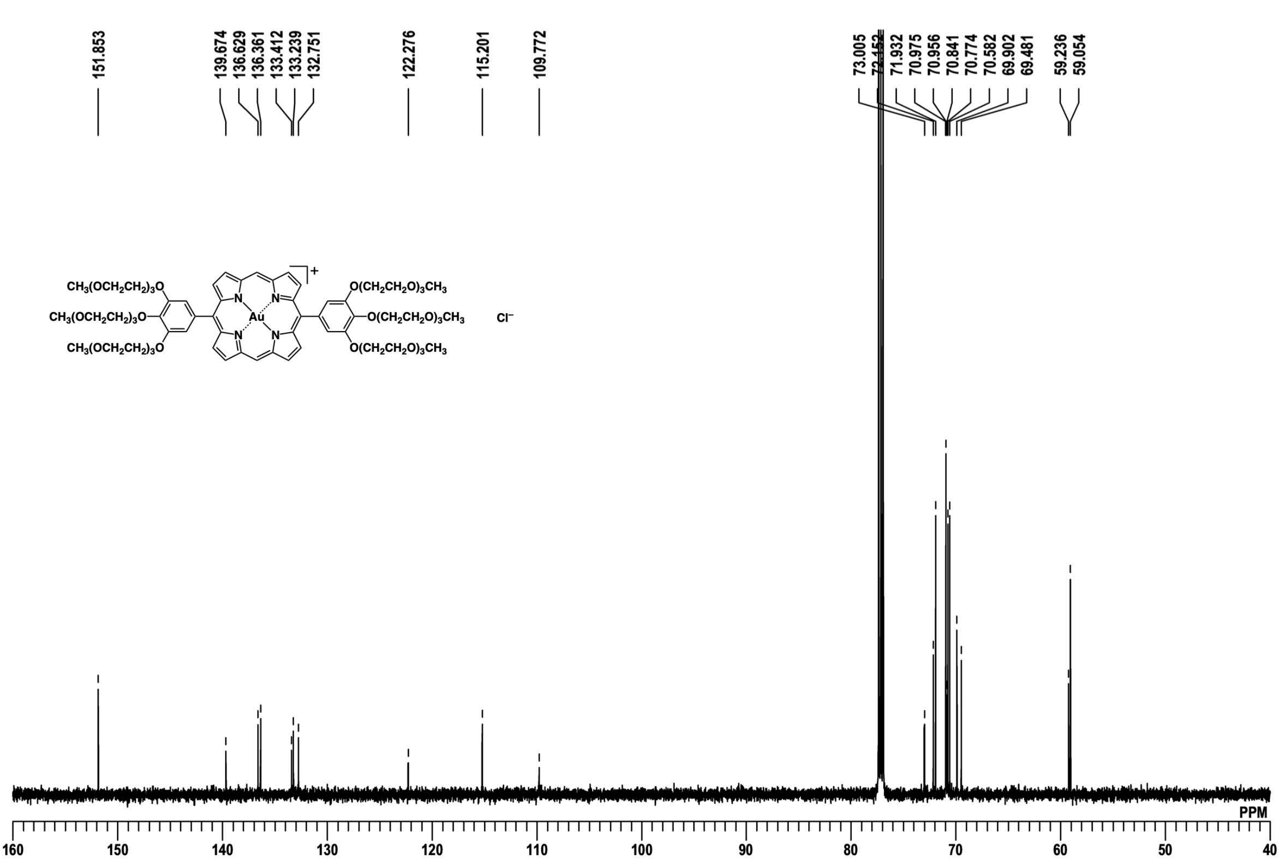

Figure S4 ^{1}H NMR (1 mM) and ^{13}C{^{1}H} NMR spectra of **1au**$^{+}$-Cl^{-} in $CDCl_3$ at 20 °C.

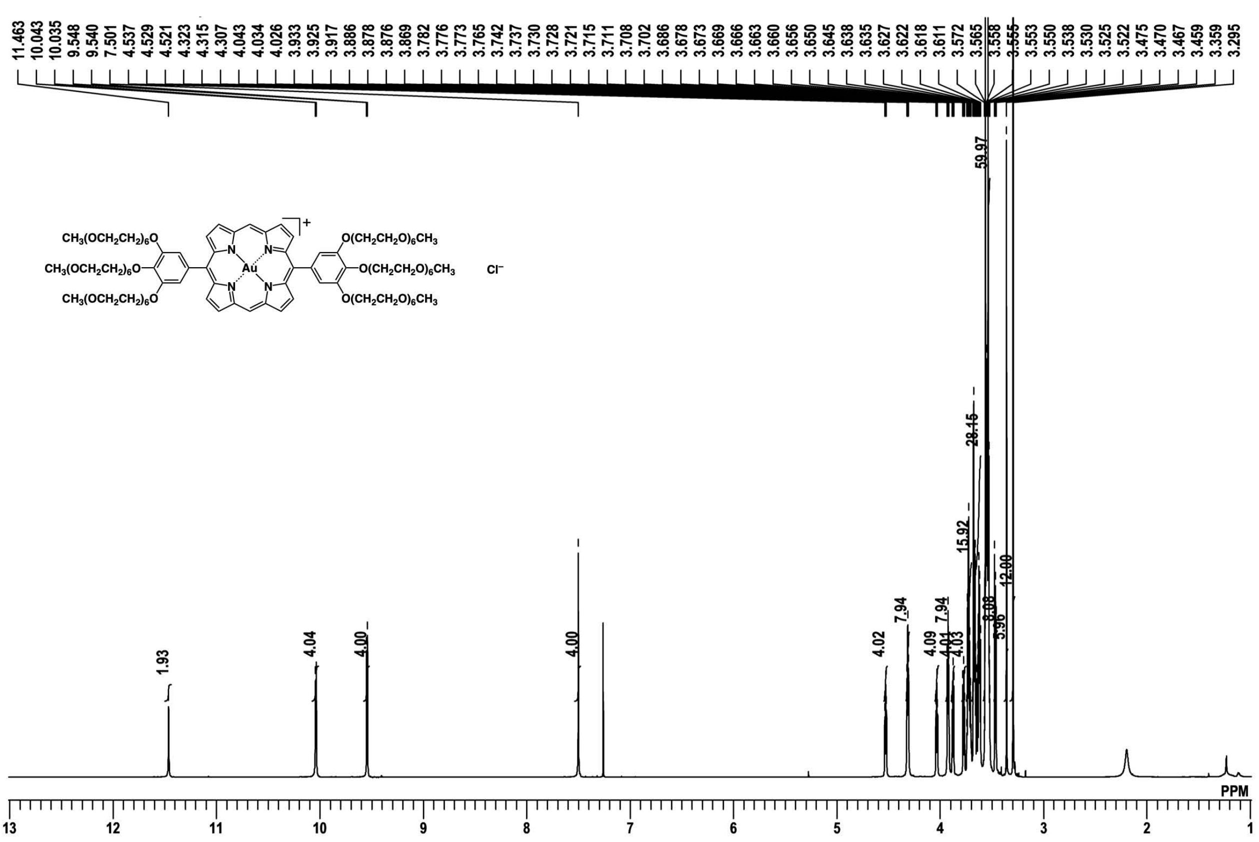

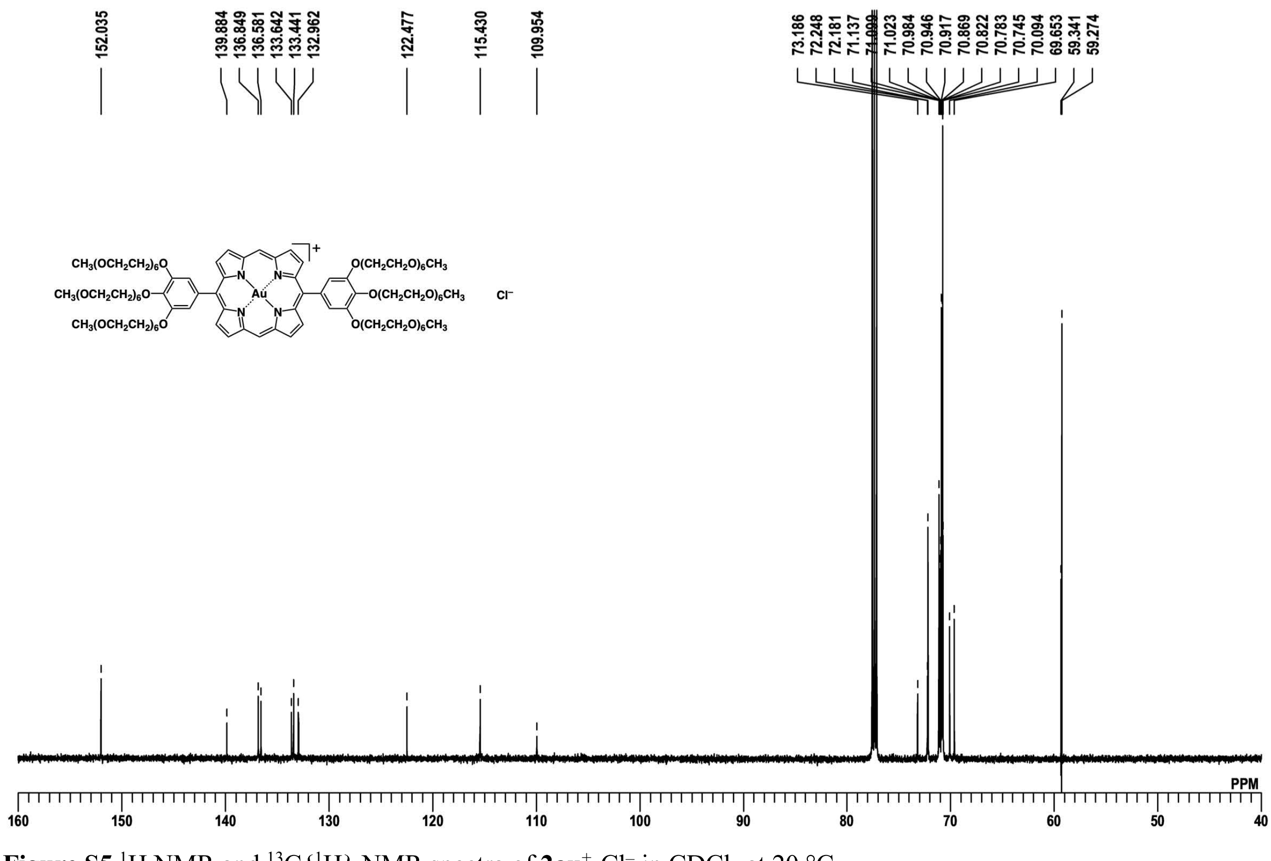

Figure S5 ^{1}H NMR and $^{13}C\{^{1}H\}$ NMR spectra of **2au**$^{+}$-Cl^{-} in $CDCl_3$ at 20 °C.

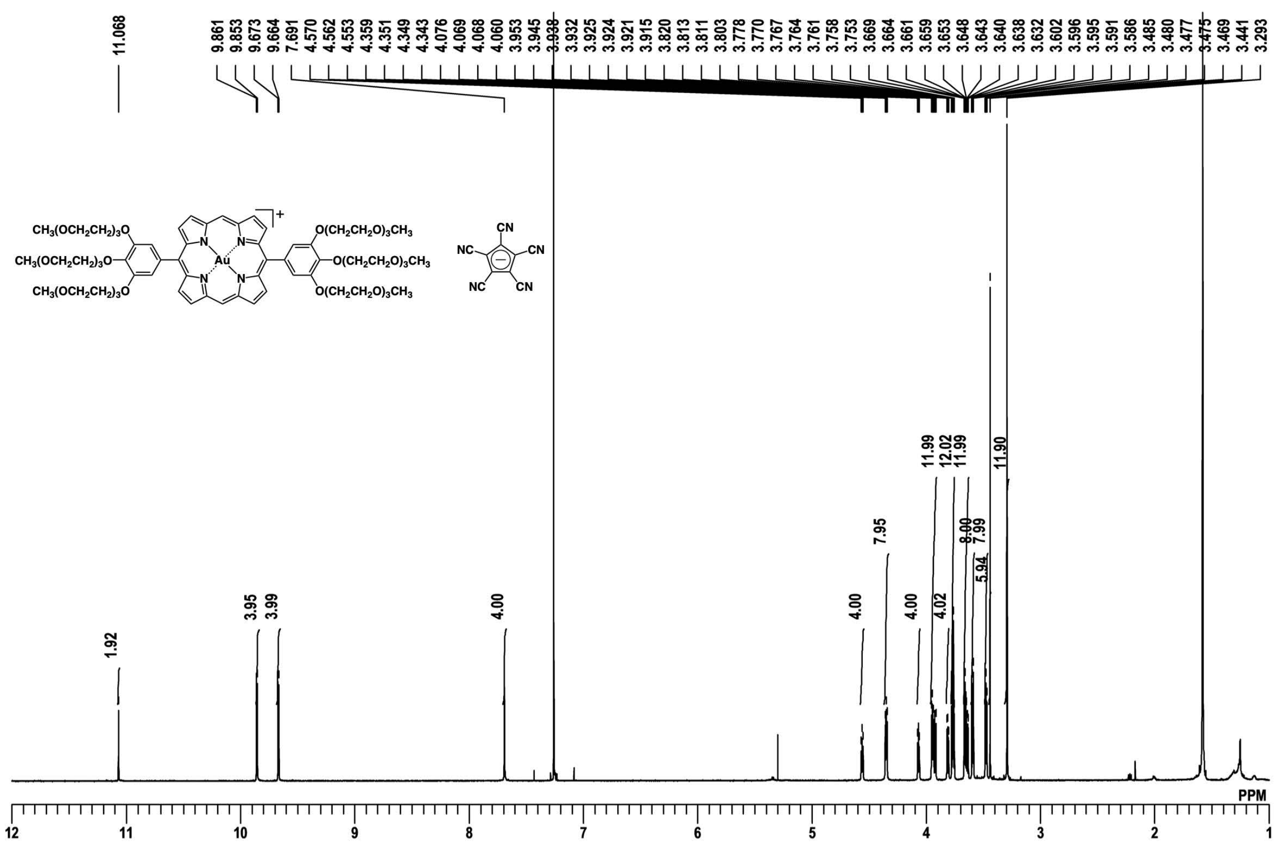

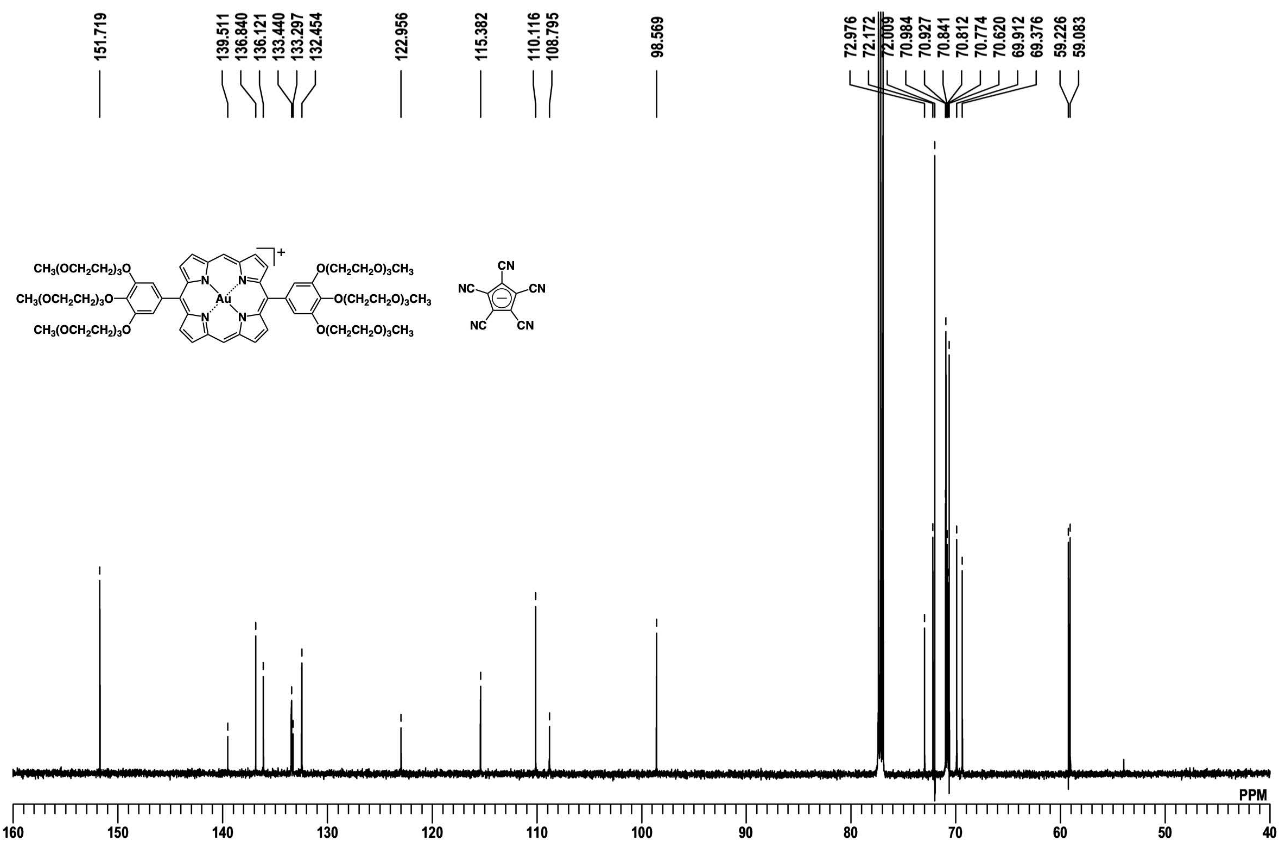

Figure S6 ^{1}H NMR (1 mM) and ^{13}C{^{1}H} NMR spectra of **1au**$^{+}$-PCCp^{-} in $CDCl_3$ at 20 °C.

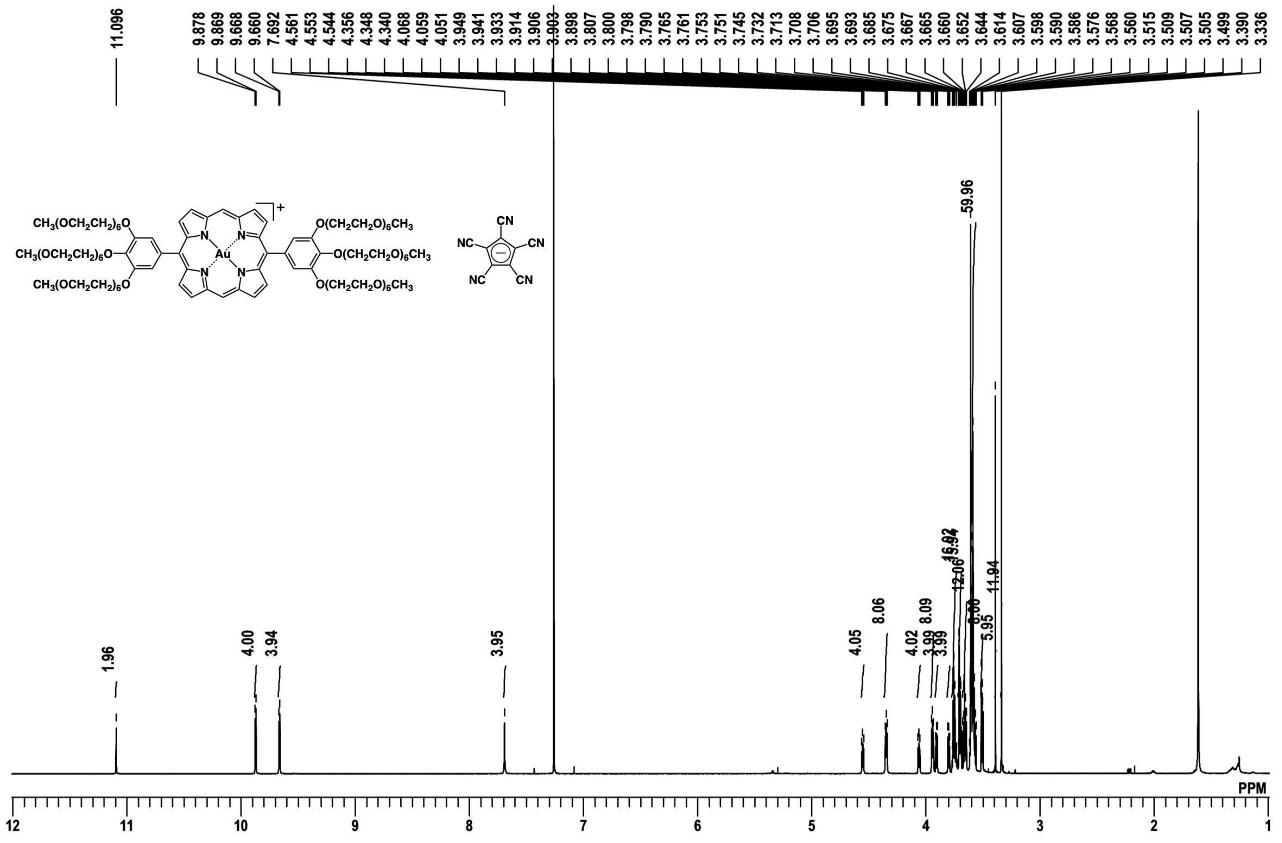

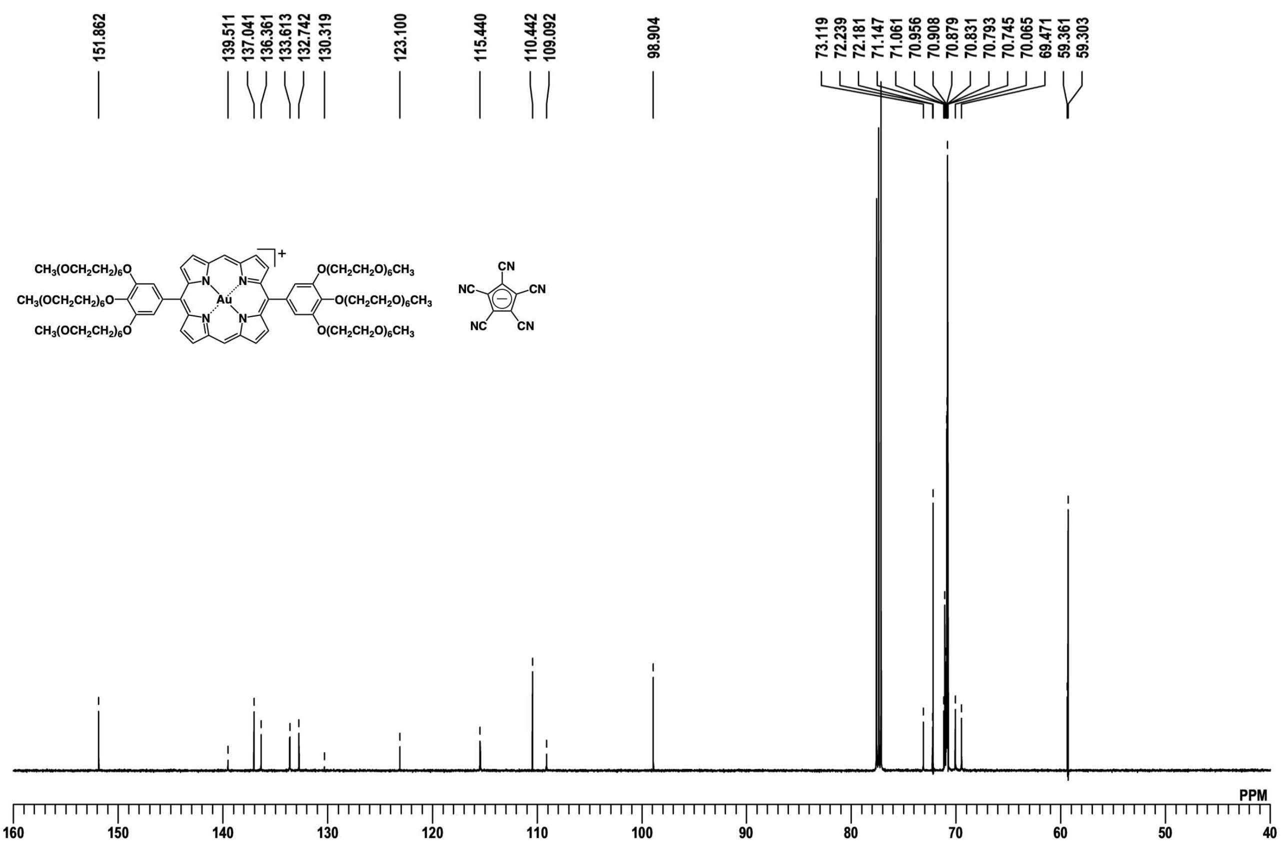

Figure S7 ^{1}H NMR (1 mM) and $^{13}C\{^{1}H\}$ NMR spectra of **2au**$^{+}$-PCCp^{-} in $CDCl_3$ at 20 °C.

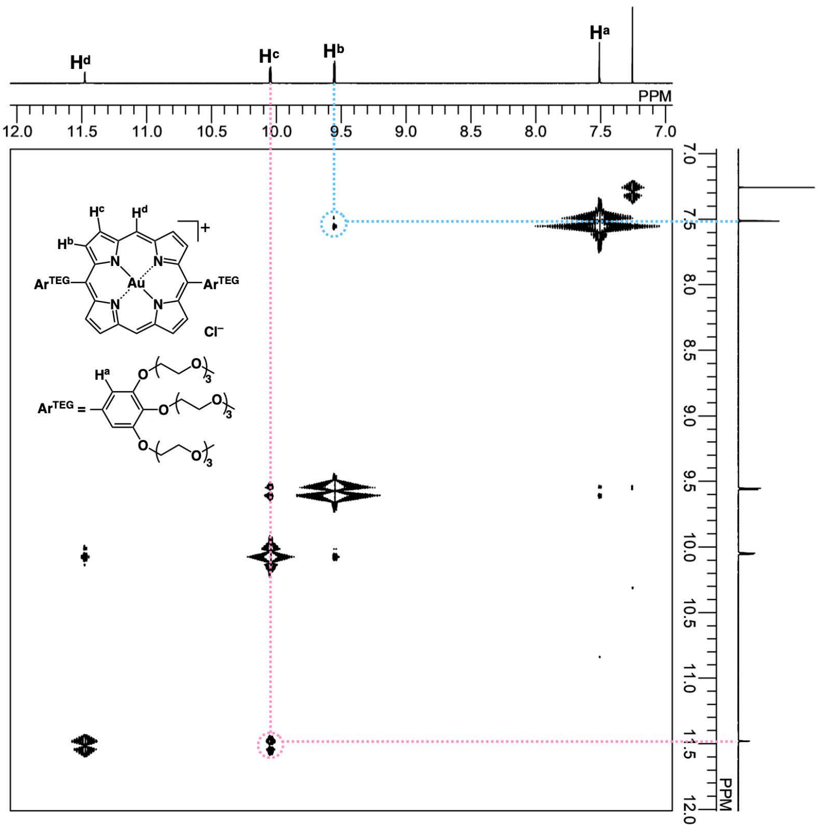

Figure S8 ^{1}H–^{1}H NOESY of **1au**$^+$-Cl$^-$ in CDCl$_3$ at 20 °C.

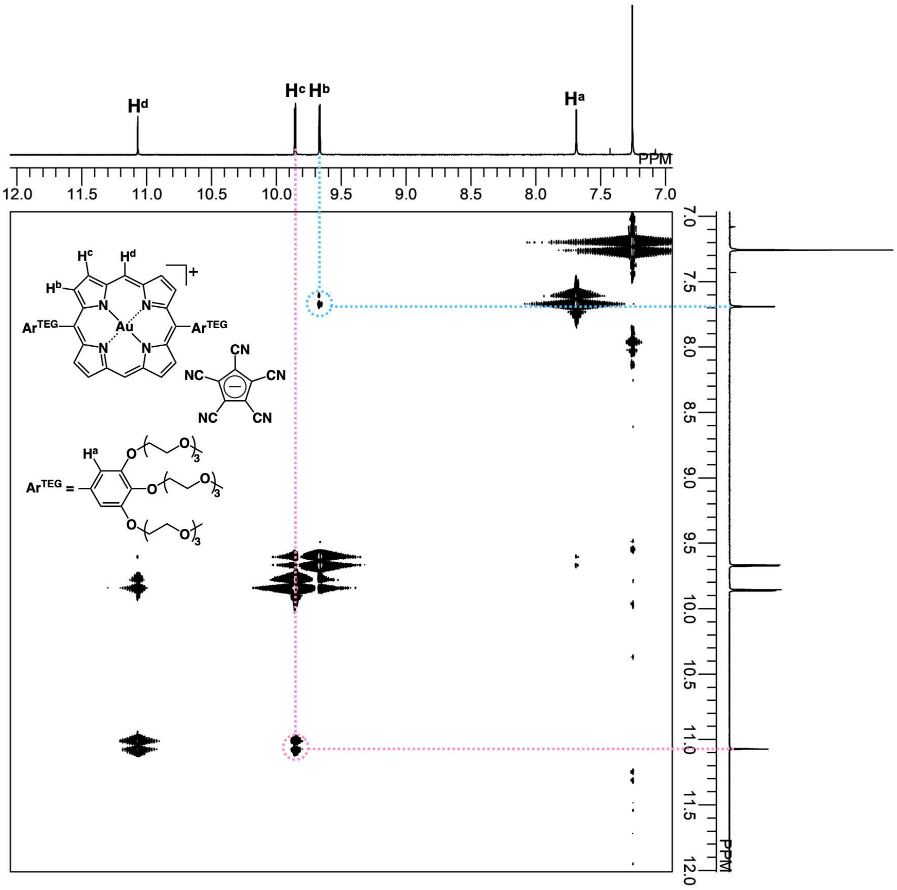

Figure S9 ^{1}H–^{1}H NOESY of **1au**$^+$-PCCp$^-$ in CDCl$_3$ at 20 °C.

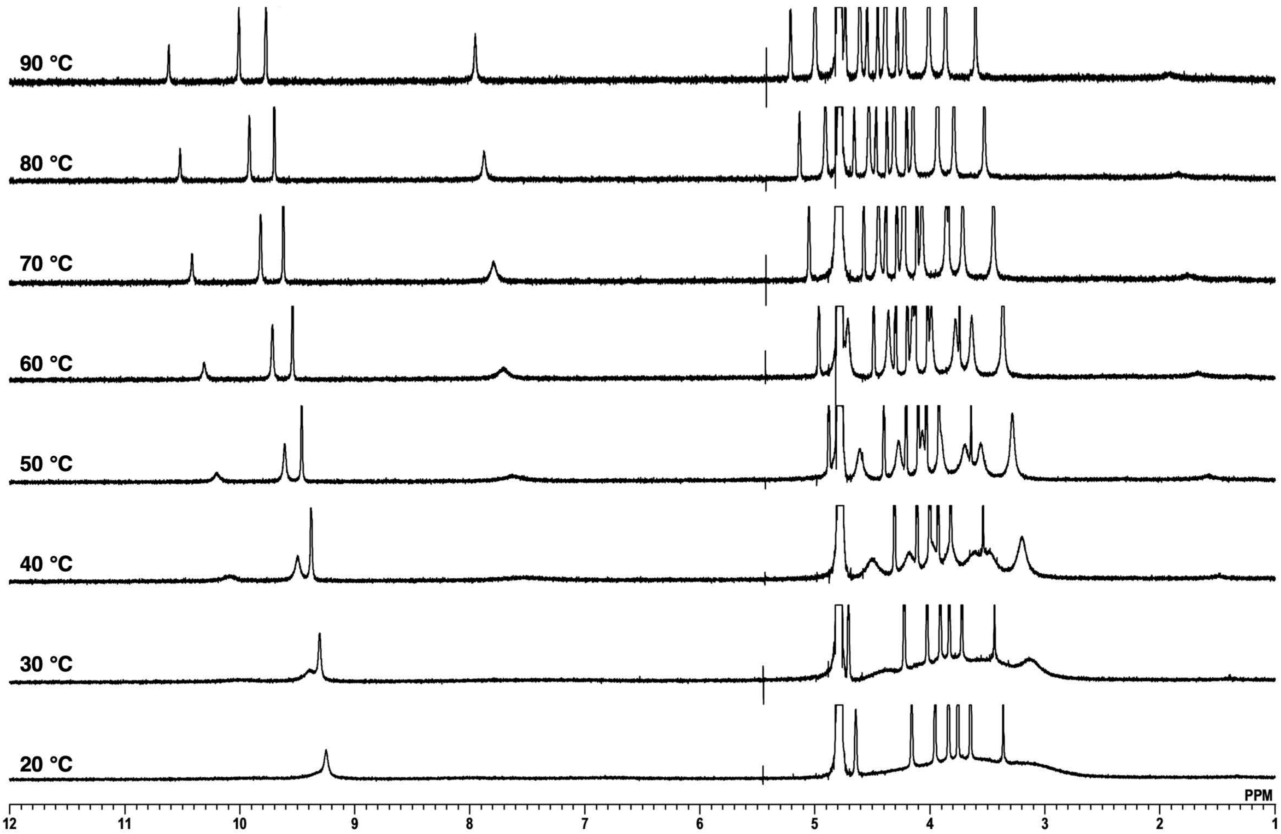

Figure S10 VT ^{1}H NMR spectra of **1au**$^+$-Cl$^-$ in D$_2$O (1 mM) at 20 to 90 °C.

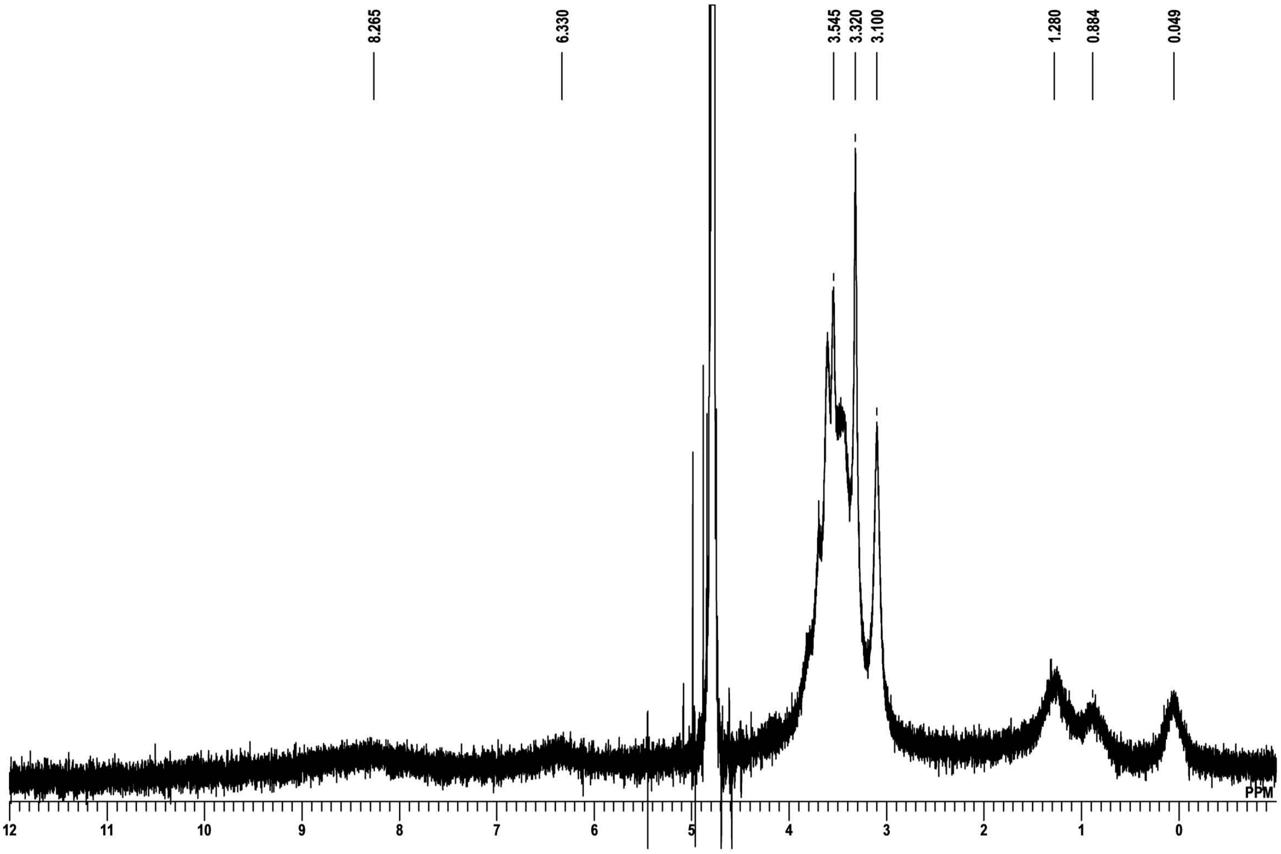

Figure S11 ^{1}H NMR spectrum of **1au**$^+$-PCCp$^-$ in D$_2$O (saturated solution (<1 mM)) at 20 °C.

2. Theoretical studies

DFT calculations. DFT calculations for the geometrical optimizations were carried out by using the *Gaussian 16* program.[S5]

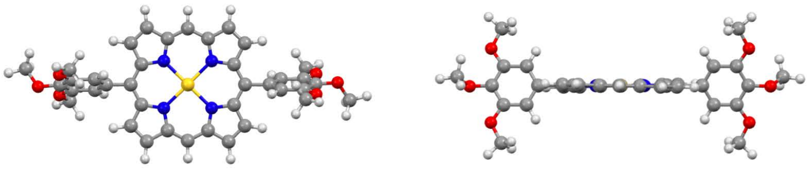

Figure S12 Optimized structure (top and side views) of **1au**$'^{+}$, in which TEG chains in **1au**$^{+}$ are replaced with methoxy units, at B3LYP/6-31+G(d,p) with LanL2DZ for Au.

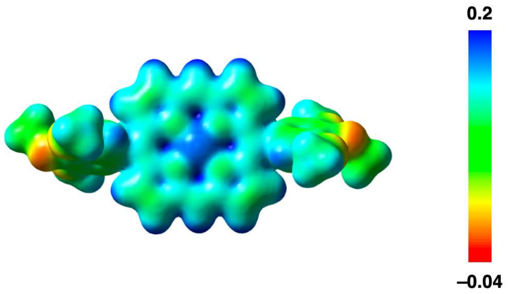

Figure S13 Electrostatic potential (ESP) mapping (δ = 0.01) of **1au**$'^{+}$ at B3LYP/6-31+G(d,p) with LanL2DZ for Au.

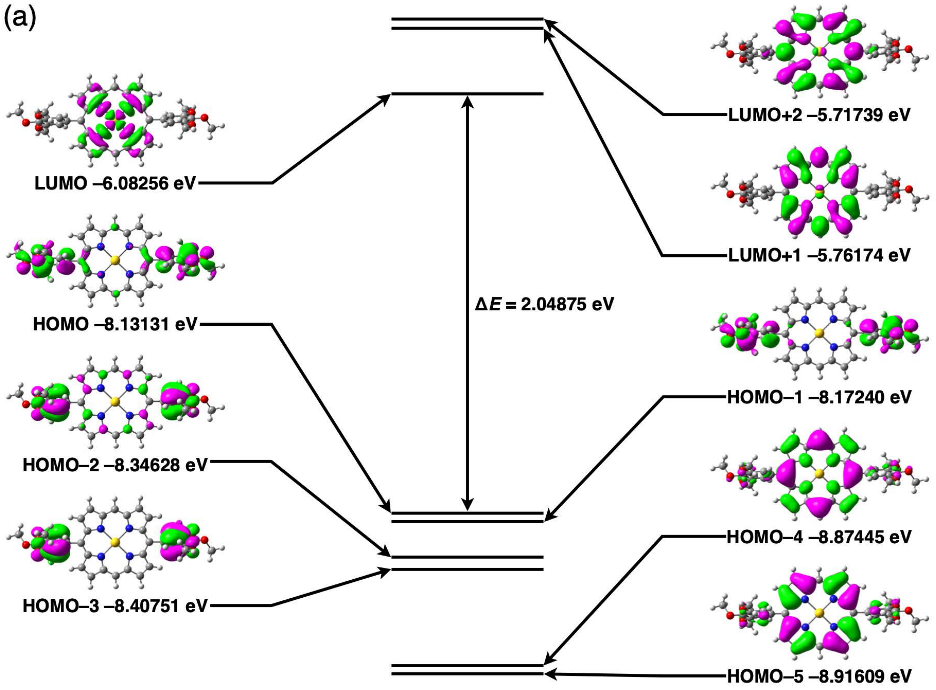

Figure S14 Molecular orbitals (HOMO/LUMO) of (a) **1au**$'^{+}$, (b) **1au**$'^{+}$ in CH_2Cl_2, and (c) **1au**$'^{+}$ in an aqueous solution estimated at (PCM-)B3LYP/6-31+G(d,p) with LanL2DZ for Au.

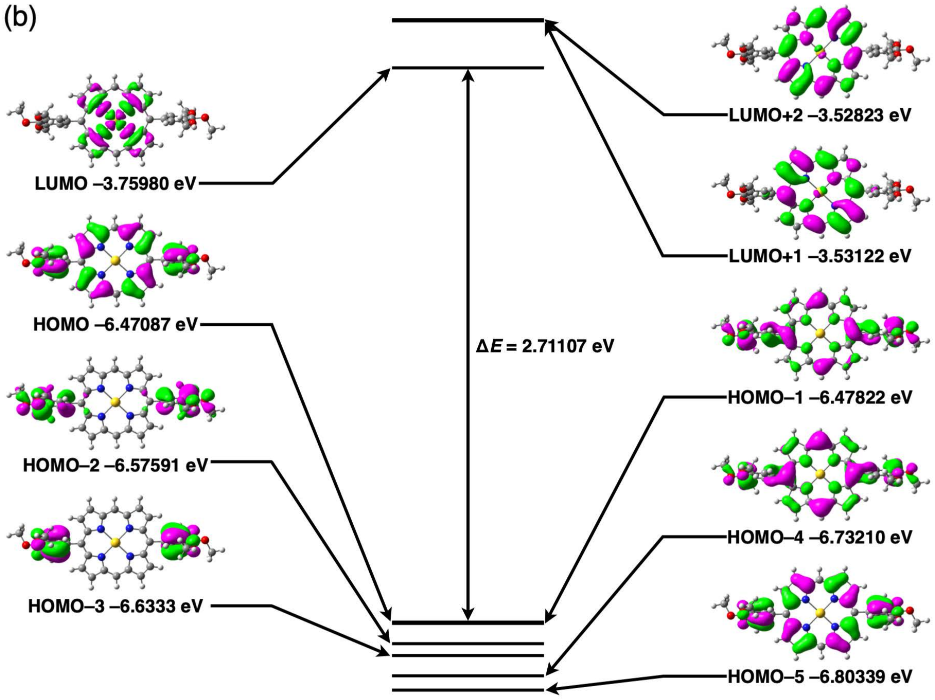

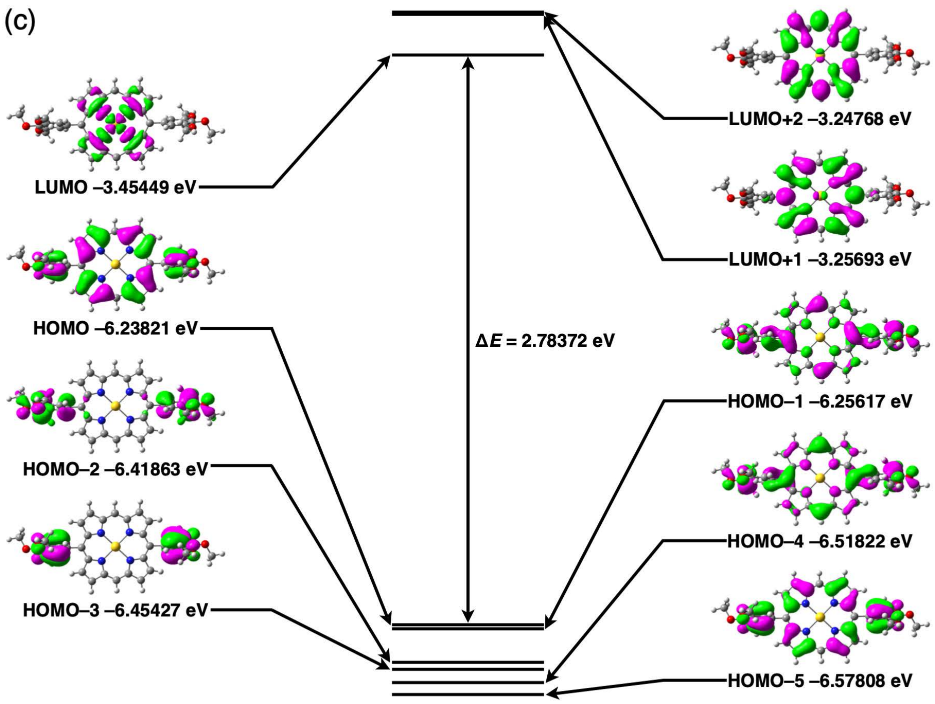

Figure S14 (Continued)

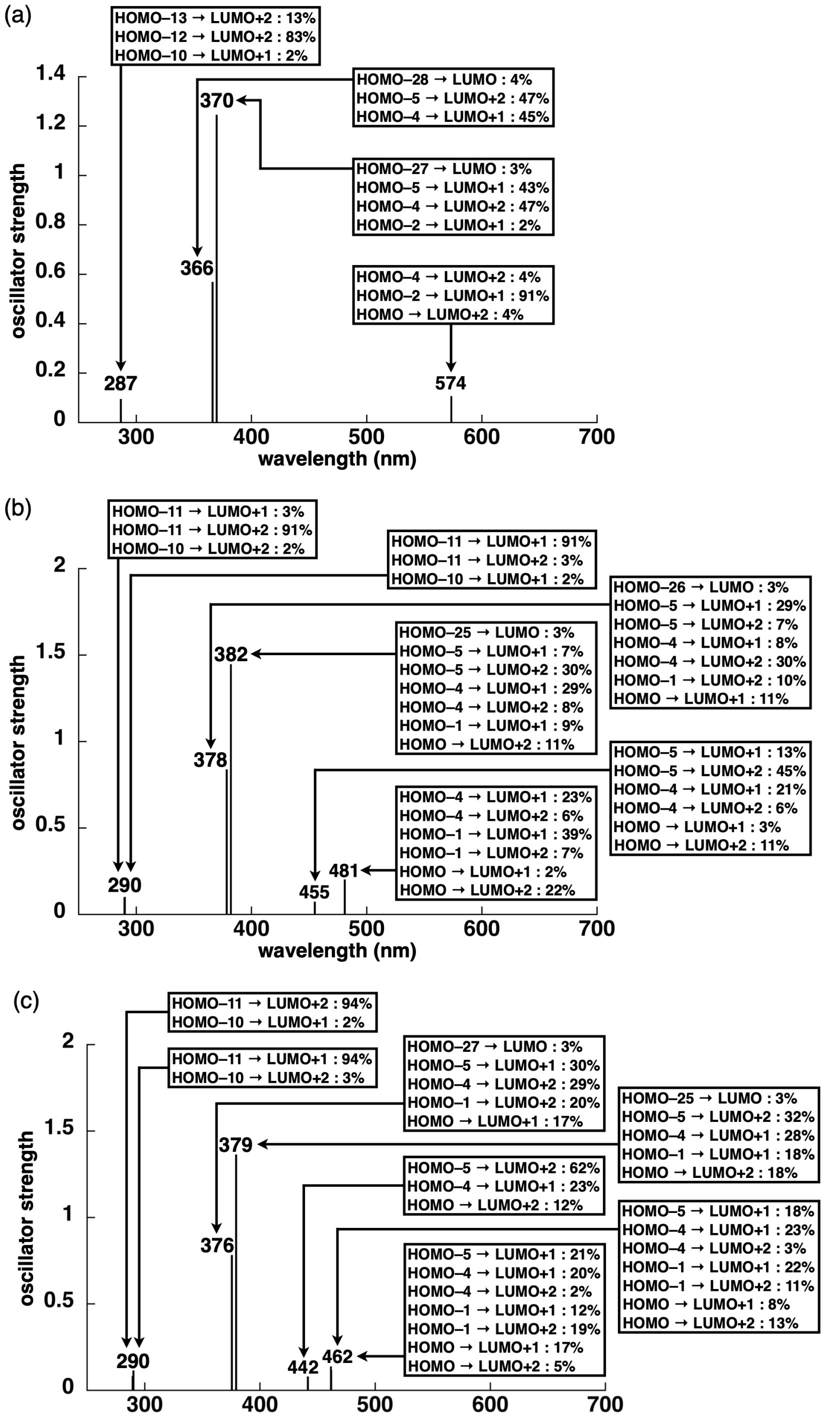

Figure S15 TD-DFT-based UV/vis absorption stick spectra of (a) **1au′**$^{+}$, (b) **1au′**$^{+}$ in CH_2Cl_2, and (c) **1au′**$^{+}$ in an aqueous solution with the transitions correlated with molecular orbitals estimated at (PCM-)B3LYP/6-31+G(d,p) with LanL2DZ for Au. These theoretical results were consistent with the observed spectra (Figure S16).

Cartesian Coordination of 1au′$^{+}$

–2272.9221967 hartree

C,10.4984210254,2.9254896075,6.4401857263
C,9.7253512871,3.062773233,5.274984904
C,10.3587719413,3.2489386586,4.0400546405
C,11.7559819487,3.2913991673,3.951703365
C,12.5294357743,3.1474584435,5.1148304983
C,11.9060244551,2.9692027709,6.3667994666
H,8.6436503859,3.0397471058,5.3241790762
H,12.2285957913,3.4281628043,2.9868221674
O,12.6374723795,2.9306273384,7.5148498195
C,13.2869565829,1.6849156615,7.8164610774
H,12.5459408657,0.8864907555,7.9403780344
H,14.0050628825,1.4187153908,7.0342756466
O,13.8869880663,3.1720389455,5.1390153629
C,14.5976152097,3.3888854416,3.9227641225
H,14.4043252775,2.5874582068,3.1990773498
H,14.3410128653,4.358968021,3.4800806864

O,9.9839621948,2.7492550265,7.6820409875
C,8.5692913316,2.7471898786,7.8505295107
H,8.1288805256,3.6988448291,7.5288634987
H,8.1018559075,1.9184570138,7.3040112674
C,9.532952009,3.409308381,2.8001877189
C,9.4060394467,4.6890081906,2.2471410199
C,8.9245156546,2.274320826,2.2517467224
N,8.7160804714,5.0310850488,1.0981386345
C,9.9843494472,5.8871410684,2.7991878186
N,8.1079237055,2.2284422858,1.1362253345
C,9.053105236,0.9372020443,2.771172334
C,8.8494815626,6.3914246926,0.9137152086
C,9.6483955934,6.9249605176,1.9805025874
H,10.569379071,5.9138275363,3.7062348521
C,7.7213093222,0.9179469434,0.9482955006
C,8.3127971676,0.1109292841,1.9777267952
H,9.6445347377,0.6796293871,3.636880363
C,8.3099332723,7.1356164311,-0.1256211772
H,9.9074962221,7.9699394614,2.0849965537
C,6.8971927038,0.4419003494,-0.0614225462
H,8.1774172978,-0.9584562127,2.0678278904
C,7.5139907282,6.6534531204,-1.1548221309
C,6.3341210298,1.1912015414,-1.0844594401
N,7.0897823698,5.3511277622,-1.3167064659
C,6.993852719,7.4449159426,-2.2336877072
N,6.5134949674,2.5415299342,-1.3006779877
C,5.4621742642,0.6735966099,-2.1007354064
C,6.3182022018,5.2954342134,-2.4633735639
C,6.2682092325,6.6154437473,-3.0372579876
H,7.1750843996,8.504326198,-2.3554913363
C,5.7830215888,2.8924546669,-2.4216260143
C,5.1199194517,1.7127959263,-2.9150239947
H,5.149682647,-0.3597557704,-2.1682810932
C,5.6858353293,4.1656580508,-2.9952248113
H,5.7398531753,6.8592004649,-3.9466578672
H,4.4711465049,1.7000260403,-3.7779528976
C,4.8632506355,4.3253017567,-4.2373684711
C,3.5996977511,4.9238179662,-4.15927599
C,5.3648915286,3.8700461366,-5.4632794274
C,2.8219407005,5.0621709995,-5.321242038
H,3.2259638364,5.263254607,-3.2010844717
C,4.590992151,4.0140248279,-6.6261192611
H,6.3476734988,3.4168417901,-5.5057655328
C,3.3128910726,4.6057845322,-6.5619405863
O,1.5862042323,5.6188892085,-5.3516605633
O,4.9832935975,3.608835344,-7.8614685295
O,2.5095358978,4.6601384836,-7.6602210271
C,0.9949937771,6.0570032651,-4.1317412841
C,6.2450588993,2.9630354575,-8.0104063655
C,2.793437938,5.7020843729,-8.6080339688
H,0.878965929,5.2263003258,-3.4249528003
H,1.5816836259,6.85979673,-3.6677425825
H,7.0697906946,3.6271804195,-7.7238821649
H,6.2905158197,2.0389446464,-7.4214566292
H,2.6803870827,6.6870688432,-8.1400868578
H,3.8010574032,5.5911796702,-9.0212339398
Au,7.6070085425,3.7880038947,-0.0958882738
H,6.3268709931,2.7200364385,-9.0699430306
H,2.055547448,5.5899696596,-9.4035216712
H,8.4022044626,2.6145423975,8.9195704331
H,13.8123142017,1.8437249209,8.7591323456
H,15.6531704398,3.3844137052,4.1950564101
H,0.0113660334,6.439829707,-4.4042900555
H,6.6715043566,-0.618759298,-0.0500443662
H,8.5321423649,8.197034838,-0.1345309445

Cartesian Coordination of 1au$^{\cdot+}$ (CH_2Cl_2)
–2272.9812287 hartree
C,-7.0982876259,0.0882077502,1.1704720212
C,-5.6950849702,0.1158625694,1.178745011
C,-4.9914289912,-0.1293398835,-0.0063728012
C,-5.6701090544,-0.3996075648,-1.2004910836
C,-7.0734753017,-0.4210951839,-1.2108576544
C,-7.7929802138,-0.1798213258,-0.0247025125
H,-5.1501700458,0.3185086347,2.0921014129
H,-5.1060055633,-0.5806694324,-2.1067673645
O,-9.1623310574,-0.2714558606,-0.0194920959
C,-9.8532220857,0.9623616856,-0.2853447814
H,-9.6125352093,1.7119753126,0.4763708645
H,-9.5955488073,1.3429001582,-1.2797417187
O,-7.8275049019,-0.6655371015,-2.3168280081
C,-7.1621028789,-0.9197905969,-3.556745951
H,-6.5566163695,-0.0597438348,-3.8643415972
H,-6.531634863,-1.8135477986,-3.4914530958
O,-7.8752340799,0.3070643975,2.265966196
C,-7.2360678781,0.5812029044,3.5155359292
H,-6.602643612,-0.2565038953,3.8278387118
H,-6.6391600809,1.4987368557,3.4623132635
C,-3.4915559974,-0.0932363643,0.0013895216
C,-2.7965923784,-1.3071000791,0.0200713384
C,-2.8613810149,1.1556096102,-0.009973035
N,-1.4252255343,-1.4730777835,0.0062467591
C,-3.4099955076,-2.6108685707,0.0607097806
N,-1.5013128988,1.3938918548,0.0155402832
C,-3.542666939,2.4252215975,-0.0502956393
C,-1.1630472478,-2.8253631731,0.0323064615
C,-2.4100112486,-3.5383192752,0.0653313411
H,-4.4760195449,-2.7806336214,0.0847571933
C,-1.3108763806,2.7586598834,-0.0011849276
C,-2.593523584,3.4044312337,-0.0407379232
H,-4.6157966981,2.5386230969,-0.0831106461
C,0.0908565964,-3.4194788699,0.0213744889
H,-2.4950555075,-4.6162131859,0.0926965507
C,-0.0908593925,3.4195829379,0.0182198485
H,-2.7358219123,4.4762026718,-0.0647826645
C,1.3108726562,-2.7585739305,0.001304434
C,1.1630449827,2.8254775165,0.029643181
N,1.5013099086,-1.3937909123,0.0167589617
C,2.5935182474,-3.4043816434,-0.037702985
N,1.425222267,1.4731685143,0.004820377
C,2.4100102596,3.5384639661,0.0619562465
C,2.8613771302,-1.1555325208,-0.009025383
C,3.5426612627,-2.4251812971,-0.0482034253
H,2.7358155655,-4.4761749022,-0.0607630707
C,2.7965899035,1.3072039057,0.0187442276
C,3.4099945258,2.6110094555,0.0581501089
H,2.4950556693,4.6163827676,0.0883207799
C,3.4915527205,0.0933234364,0.0011606926
H,4.6157896679,-2.538613372,-0.080956428
H,4.4760195588,2.7807968568,0.0819959113

C,4.9914255349,0.1294179278,-0.0066794996
C,5.6951128204,-0.1146834383,1.178647781
C,5.6700742256,0.3985724165,-1.2010664322
C,7.0983136469,-0.087031914,1.170309893
H,5.1502228368,-0.3164723136,2.0922088974
C,7.0734417766,0.4200363997,-1.2114960409
H,5.1059456849,0.5787878483,-2.1074957364
C,7.7929763267,0.1798717511,-0.0251344886
O,7.8752947368,-0.3048512074,2.2659874684
O,7.8274367405,0.6634284803,-2.3177208382
O,9.1623276982,0.2715083071,-0.0200773567
C,7.2361691504,-0.5778226126,3.5158340462
C,7.1619941376,0.9165308684,-3.5578519638
C,9.8532077791,-0.9627042088,-0.2841201479
H,6.6027472138,0.2601716314,3.8273689529
H,6.6392679631,-1.4954111815,3.4634895394
H,6.5564841006,0.0562057068,-3.8646222154
H,6.5315412288,1.8103580816,-3.493374269
H,9.6125217235,-1.7112007522,0.4786909014
H,9.5955231722,-1.3446941864,-1.2779593661
Au,-0.0000015896,0.0000480711,0.0113192776
H,7.954833639,1.0825890093,-4.2871734648
H,10.918785898,-0.7302319494,-0.2459064037
H,-8.0441951421,0.714000879,4.2348384626
H,-10.9187981425,0.7299380384,-0.2467800647
H,-7.9549657353,-1.086542831,-4.2858835724
H,8.0443194524,-0.7099383252,4.2352362249
H,-0.1198742195,4.5032988517,0.0229866861
H,0.1198717974,-4.5031899417,0.0271406242

Cartesian Coordination of 1au$^{\cdot+}$ (an aqueous solution)
–2272.9904313 hartree
C,-7.0976196333,0.0965517834,1.1827710933
C,-5.6944772141,0.125070655,1.1911882965
C,-4.9899293766,-0.1229829966,0.0074223185
C,-5.6676984993,-0.402203829,-1.1851337673
C,-7.0703873742,-0.43046038,-1.1948934747
C,-7.7908765882,-0.1805394389,-0.0111004024
H,-5.1498313001,0.3338442997,2.1031633649
H,-5.1029550072,-0.5887718428,-2.0896872087
O,-9.1620112118,-0.2680717692,-0.0029047792
C,-9.8474166882,0.9402325604,-0.381272715
H,-9.6045981301,1.7537394378,0.3110629254
H,-9.5847444762,1.2299101734,-1.4040326521
O,-7.8237572919,-0.6925268887,-2.2982912441
C,-7.1564204707,-0.9385400029,-3.540227782
H,-6.5594615299,-0.0724209625,-3.846078566
H,-6.5173823761,-1.826072635,-3.4765540374
O,-7.8740428932,0.3210690074,2.2782617514
C,-7.2323585548,0.6138662371,3.523430924
H,-6.5986160667,-0.2190962193,3.8470289506
H,-6.6352074526,1.5298073013,3.4550765676
C,-3.4897816201,-0.0866945042,0.0152672891
C,-2.7973990641,-1.3023123294,0.0238770439
C,-2.8587616968,1.1618887901,0.0128885269
N,-1.4271102591,-1.4708145205,0.0118426896
C,-3.4135329232,-2.6056218423,0.0514159686
N,-1.4987994289,1.3985623726,0.0326677925
C,-3.5391149735,2.4328730173,-0.0126378644
C,-1.1671518016,-2.8233629894,0.0257820722
C,-2.4153587876,-3.5348969961,0.049384398
H,-4.4797371408,-2.7743905484,0.0714081669
C,-1.3066842029,2.7625713025,0.0262963945
C,-2.5887817115,3.410879252,-0.0001066451
H,-4.6122560376,2.5483263802,-0.0378222578
C,0.0856910686,-3.4200665026,0.0149604915
H,-2.5020934183,-4.6127978891,0.0662560965
C,-0.0853284515,3.4213299041,0.0420988213
H,-2.7294945656,4.4830579936,-0.0125945115
C,1.3069976732,-2.761189751,0.0033413434
C,1.1674994552,2.8245757404,0.0475565777
N,1.4989879896,-1.3972484047,0.0196664098
C,2.5891811015,-3.409196955,-0.0277270745
N,1.4273550096,1.4721614429,0.0234118125
C,2.4158203038,3.5358680761,0.0747054097
C,2.858912235,-1.1603770114,0.0018197332
C,3.5394278605,-2.4310461759,-0.0328375514
H,2.7299777377,-4.4812492547,-0.047851901
C,2.797630655,1.3035504775,0.0323089848
C,3.4139372212,2.6065499226,0.0684754291
H,2.5026289848,4.6136201879,0.0991121943
C,3.4899035221,0.0880825414,0.0137054814
H,4.6126166409,-2.546098035,-0.0584388764
H,4.4802229835,2.7749811043,0.0879288088
C,4.9901350793,0.1241314665,0.0058124924
C,5.6942781295,-0.1182782424,1.1909435314
C,5.6681491546,0.3969622408,-1.1880460555
C,7.0974313693,-0.090612573,1.1825556943
H,5.1493576678,-0.322065176,2.1038872227
C,7.070894926,0.423901268,-1.1979152663
H,5.1036297149,0.5793624159,-2.0935833388
C,7.7910435872,0.179275581,-0.0127628772
O,7.8736010409,-0.3094442579,2.2794037815
O,7.824376511,0.6798597938,-2.3027074661
O,9.1622953724,0.2650697642,-0.0047193843
C,7.2315322571,-0.5947840075,3.5261006133
C,7.1569497161,0.9209199281,-3.5455639318
C,9.8461814964,-0.9468077505,-0.3742013837
H,6.5975497977,0.2400127476,3.8444122562
H,6.6344950388,-1.5111684055,3.4630770893
H,6.558558889,0.0542880402,-3.8470857108
H,6.519240374,1.8096759053,-3.48578488
H,9.6027478208,-1.7547991531,0.3243544233
H,9.582812832,-1.2440177367,-1.3946127886
Au,0.000089108,0.0006700918,0.0209772444
H,7.9486554073,1.0892028889,-4.2755983875
H,10.913317845,-0.7243661558,-0.3201460708
H,-8.0391904972,0.7578370772,4.2420544103
H,-10.9142777194,0.7169964954,-0.3251344824
H,-7.9481496014,-1.1114679872,-4.2691476404
H,8.0381328572,-0.7343033199,4.2458635475
H,-0.1123983079,4.5050883674,0.0494245331
H,0.1127579098,-4.5038515931,0.0144360072

[S5] M. J. Frisch, G. W. Trucks, H. B. Schlegel, G. E. Scuseria, M. A. Robb, J. R. Cheeseman, G. Scalmani, V. Barone, G. A. Petersson, H. Nakatsuji, X. Li, M. Caricato, A. V. Marenich, J. Bloino, B. G. Janesko, R. Gomperts, B. Mennucci, H. P. Hratchian, J. V. Ortiz, A. F. Izmaylov, J. L. Sonnenberg, D. Williams-Young, F. Ding, F. Lipparini, F. Egidi, J.

Goings, B. Peng, A. Petrone, T. Henderson, D. Ranasinghe, V. G. Zakrzewski, J. Gao, N. Rega, G. Zheng, W. Liang, M. Hada, M. Ehara, K. Toyota, R. Fukuda, J. Hasegawa, M. Ishida, T. Nakajima, Y. Honda, O. Kitao, H. Nakai, T. Vreven, K. Throssell, J. A. Montgomery, Jr., J. E. Peralta, F. Ogliaro, M. J. Bearpark, J. J. Heyd, E. N. Brothers, K. N. Kudin, V. N. Staroverov, T. A. Keith, R. Kobayashi, J. Normand, K. Raghavachari, A. P. Rendell, J. C. Burant, S. S. Iyengar, J. Tomasi, M. Cossi, J. M. Millam, M. Klene, C. Adamo, R. Cammi, J. W. Ochterski, R. L. Martin, K. Morokuma, O. Farkas, J. B. Foresman, D. J. Fox, *Gaussian 16*, Revision C.01, Gaussian, Inc., Wallingford CT, 2016.

3. Examination of organized structures

Dynamic light scattering (DLS). DLS measurements were obtained with a Malvern Zetasizer Nano-ZS dynamic light scattering instrument.

Differential scanning calorimetry (DSC). The phase transitions were measured on a differential scanning calorimetry (Shimadzu DSC-60). The water-containing samples were encapsulated in crimped aluminum pans to prevent the evaporation of water upon heating.

Polarizing optical microscopy (POM). POM observations were carried out with a Nikon ECLIPSE LV100N-POL polarizing optical microscope equipped with a Mettler-Toledo HS82 hot stage system. The assembled cells filled with the water-containing samples were sealed promptly by an epoxy glue to prevent the evaporation of water upon heating.

Synchrotron X-ray diffraction analysis (XRD). High-resolution XRD analyses were carried out using a synchrotron radiation X-ray beam with wavelengths of 1.00 Å on BL40B2 (**1au**$^+$-Cl$^-$, **1au**$^+$-PCCp$^-$, **2**, **2au**$^+$-Cl$^-$, **1**$_{70\%}$, **1**$_{50\%}$, **1**$_{20\%}$, **1au**$^+$-Cl$^-_{75\%}$, **1au**$^+$-Cl$^-_{50\%}$, **1au**$^+$-PCCp$^-_{70\%}$, **1au**$^+$-PCCp$^-_{60\%}$, **1au**$^+$-PCCp$^-_{50\%}$, **1au**$^+$-PCCp$^-_{20\%}$, **2au**$^+$-PCCp$^-_{50\%}$, and **2au**$^+$-PCCp$^-_{20\%}$), 0.496 Å on BL19B2 (**1**, **2au**$^+$-PCCp$^-$, **1au**$^+$-Cl$^-_{70\%}$, **1au**$^+$-Cl$^-_{60\%}$, **1au**$^+$-Cl$^-_{20\%}$, **1au**$^+$-PCCp$^-_{40\%}$, **1au**$^+$-PCCp$^-_{30\%}$, **1au**$^+$-PCCp$^-_{10\%}$, **1au**$^+$-PCCp$^-_{1\%}$, **2**$_{70\%}$, **2**$_{50\%}$, **2**$_{20\%}$, **2au**$^+$-Cl$^-_{70\%}$, **2au**$^+$-Cl$^-_{50\%}$, **2au**$^+$-Cl$^-_{20\%}$, **2au**$^+$-PCCp$^-_{1\%}$, and sheared samples (**1au**$^+$-PCCp$^-$, **1au**$^+$-PCCp$^-_{50\%}$, **1au**$^+$-PCCp$^-_{40\%}$)), and 0.689 Å on BL19B2 (**2au**$^+$-PCCp$^-_{80\%}$, **2au**$^+$-PCCp$^-_{70\%}$, **2au**$^+$-PCCp$^-_{60\%}$, **2au**$^+$-PCCp$^-_{40\%}$, **2au**$^+$-PCCp$^-_{30\%}$, and **2au**$^+$-PCCp$^-_{10\%}$) at SPring-8 (Hyogo, Japan). The diffractions were detected by a large Debye-Scherrer camera with a Pilatus3S 2M (Dectris Ltd., Switzerland) as a detector. The camera lengths were set at 427.5 mm for **1au**$^+$-Cl$^-$, **1au**$^+$-PCCp$^-$, **1au**$^+$-Cl$^-_{75\%}$, **1au**$^+$-Cl$^-_{50\%}$, and **1au**$^+$-PCCp$^-_{50\%}$, 435.3 mm for **2**, **2au**$^+$-Cl$^-$, **2au**$^+$-PCCp$^-_{50\%}$, and **2au**$^+$-PCCp$^-_{20\%}$, 435.3 mm for **1**$_{70\%}$, **1**$_{50\%}$, **1**$_{20\%}$, **1au**$^+$-PCCp$^-_{70\%}$, **1au**$^+$-PCCp$^-_{60\%}$, and **1au**$^+$-PCCp$^-_{20\%}$, 756.0 nm for **1au**$^+$-Cl$^-_{70\%}$, **1au**$^+$-Cl$^-_{60\%}$, and **1au**$^+$-Cl$^-_{20\%}$, 755.0 mm for **2au**$^+$-PCCp$^-$, **1au**$^+$-PCCp$^-_{1\%}$, **2**$_{70\%}$, **2**$_{50\%}$, **2**$_{20\%}$, **2au**$^+$-Cl$^-_{70\%}$, **2au**$^+$-Cl$^-_{50\%}$, **2au**$^+$-Cl$^-_{20\%}$, and **2au**$^+$-PCCp$^-_{1\%}$, 761.0 mm for **1**, **1au**$^+$-PCCp$^-_{40\%}$, **1au**$^+$-PCCp$^-_{30\%}$, **1au**$^+$-PCCp$^-_{10\%}$, and sheared samples (**1au**$^+$-PCCp$^-$, **1au**$^+$-PCCp$^-_{50\%}$, **1au**$^+$-PCCp$^-_{40\%}$), and 763.0 mm for **2au**$^+$-PCCp$^-_{80\%}$, **2au**$^+$-PCCp$^-_{70\%}$, **2au**$^+$-PCCp$^-_{60\%}$, **2au**$^+$-PCCp$^-_{40\%}$, **2au**$^+$-PCCp$^-_{30\%}$, and **2au**$^+$-PCCp$^-_{10\%}$. The diffraction patterns were obtained with a 0.01° step in 2θ on BL40B2 and a 0.02° step in 2θ on BL19B2. An exposure time of the X-ray beam was 10 sec. The water-containing samples were sealed to prevent the evaporation of water upon heating.

Magnetic orientation. A 10% solution of **1au**$^+$-PCCp$^-$ in MeOH (20 μL) was drop-cast on the center of a sapphire glass substrate (0.1 mm in thickness, 20 mm in diameter). The glass substrate was placed inside of a hand-made cylindrical container (26 mm in inner diameter, 30 mm in outer diameter, 5 mm in height), and the top of the container was loosely covered with a glass plate. This setup was placed in the horizontally directed bore of a 10-T superconducting magnet with a field (JASTEC model JMTD-10T100) in such a way that the glass substrate become horizontal. Then the setup was left in the magnetic field for 14 hours without or with in-plane rotation of the glass substrate, where the MeOH solution was condensed to dryness. For preparing a reference sample without the magnetic treatment, the same drying procedure was performed without the magnetic field and rotation.

XRD measurements of the magnetically oriented samples. XRD measurement of the magnetically oriented samples of **1au**$^+$-PCCp$^-$ was carried out by a Rigaku NANOPIX 3.5 m system equipped with a Rigaku HyPix-6000 detector. The scattering vector q and the position of an incident X-ray beam on the detector were calibrated using several orders of layer reflections from silver behenate. The sample-to-detector distance was set at 66.59 mm. An X-ray beam (CuKα, wavelength = 1.5418 Å) was irradiated in the direction vertical to the glass substrate.

Scanning transmission electron microscopy (STEM). Bright-field (BF) and high-angle annular dark-field (HAADF) STEM imaging and energy-dispersive spectroscopy (EDS) analysis were carried out on a Thermo Fisher Scientific Talos F200X G2 equipped with an EDS detector (Super-X G2) at an acceleration voltage of 80 kV. The specimens for STEM were prepared by drop-casting 10 μL of an 25 μM aqueous solution of **1au**$^+$-PCCp$^-$ onto a thin carbon-coated copper grid (SHR-C075, Okenshoji Co., Ltd.), blotting the excess solution with filter paper, and lyophilizing the grid at 5 Pa. A probe current for EDS mapping was set to ca 1.8 nA. Analysis of EDS spectrum imaging was carried out on Velox software (Thermo Fisher Scientific) using a single three-parameter Bethe-Heitler function as a background correction parameter.

Atomic force microscopy (AFM). AFM measurements were performed by a Bruker AXS Multimode 8 AFM system in tapping mode in air at r.t. on a Si wafer and an OLYMPUS silicon cantilever OMCL-AC160TS-C3 was used. The specimens for AFM were prepared by drop-casting a 0.1 mM aqueous solution of **1au**$^+$-PCCp$^-$ onto a Si wafer, blotting the excess solution with filter paper, and lyophilizing the wafer under reduced pressure.

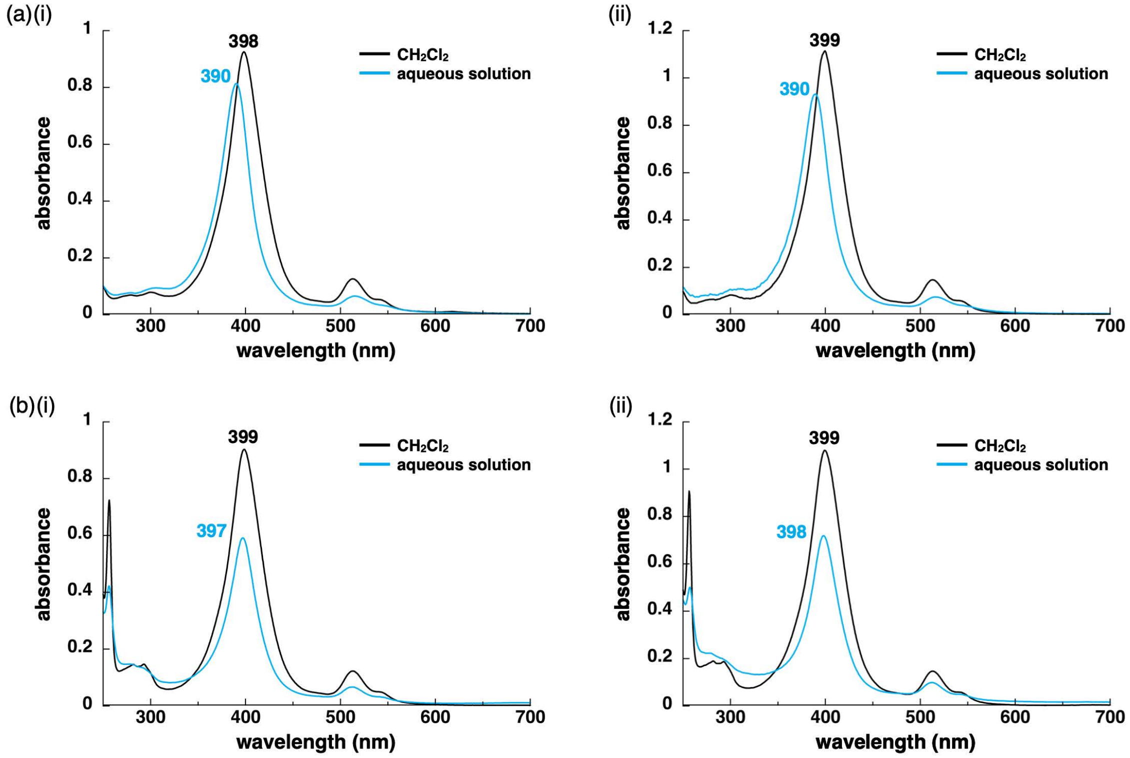

Figure S16 UV/vis absorption spectra of (a) **1au**$^{+}$-Cl^{-} and (b) **1au**$^{+}$-PCCp^{-} in CH_2Cl_2 and aqueous solutions ((i) 8 µM and (ii) 1.0×10^{-4} M).

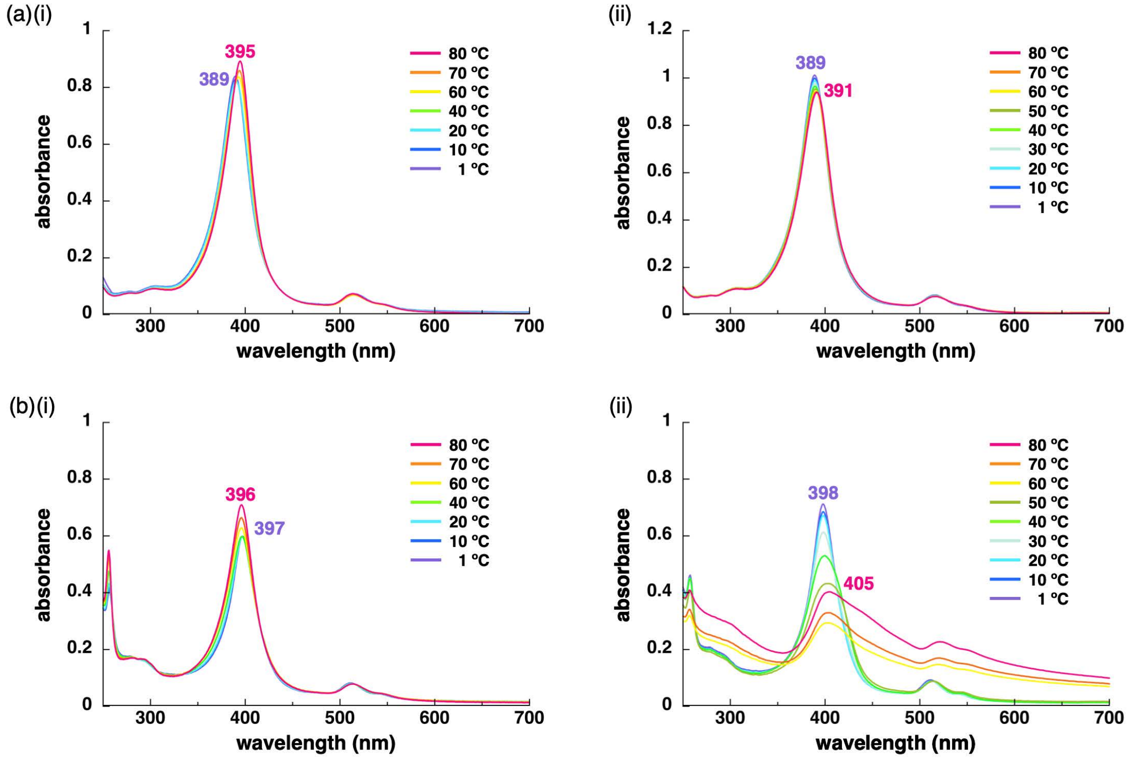

Figure S17 VT UV/vis absorption spectral changes of (a) **1au**$^{+}$-Cl^{-} and (b) **1au**$^{+}$-PCCp^{-} in aqueous solutions ((i) 8 µM and (ii) 1.0×10^{-4} M) from 80 to 1 °C.

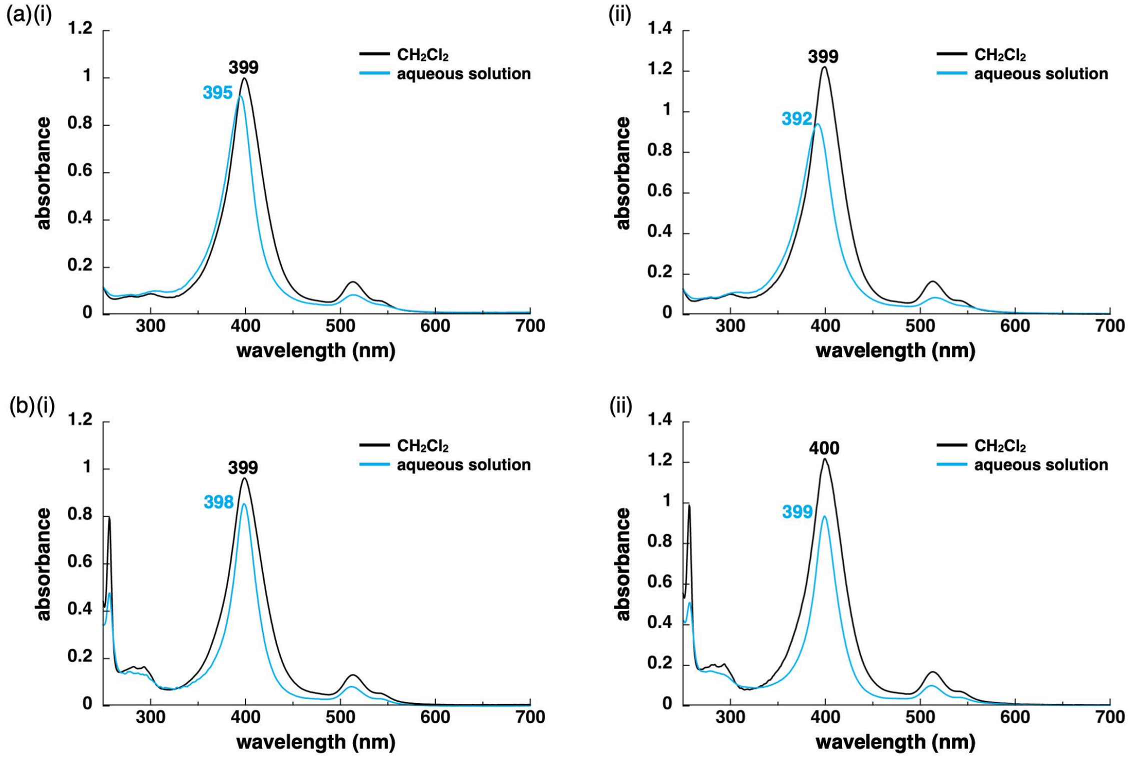

Figure S18 UV/vis absorption spectra of (a) **2au**$^{+}$-Cl^{-} and (b) **2au**$^{+}$-PCCp^{-} in CH_2Cl_2 and aqueous solutions ((i) 8 μM and (ii) 1.0 × 10^{-4} M).

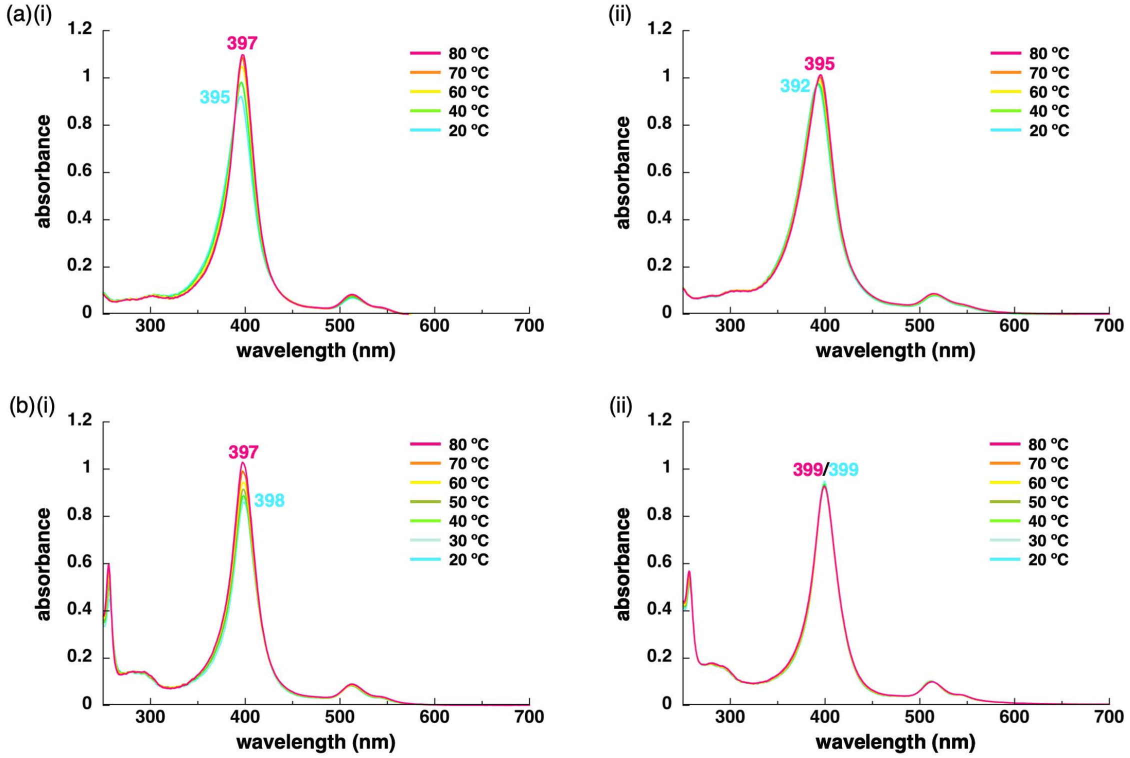

Figure S19 VT UV/vis absorption spectral changes of (a) **2au**$^{+}$-Cl^{-} and (b) **2au**$^{+}$-PCCp^{-} in aqueous solutions ((i) 8 μM and (ii) 1.0 × 10^{-4} M) from 80 to 20 °C.

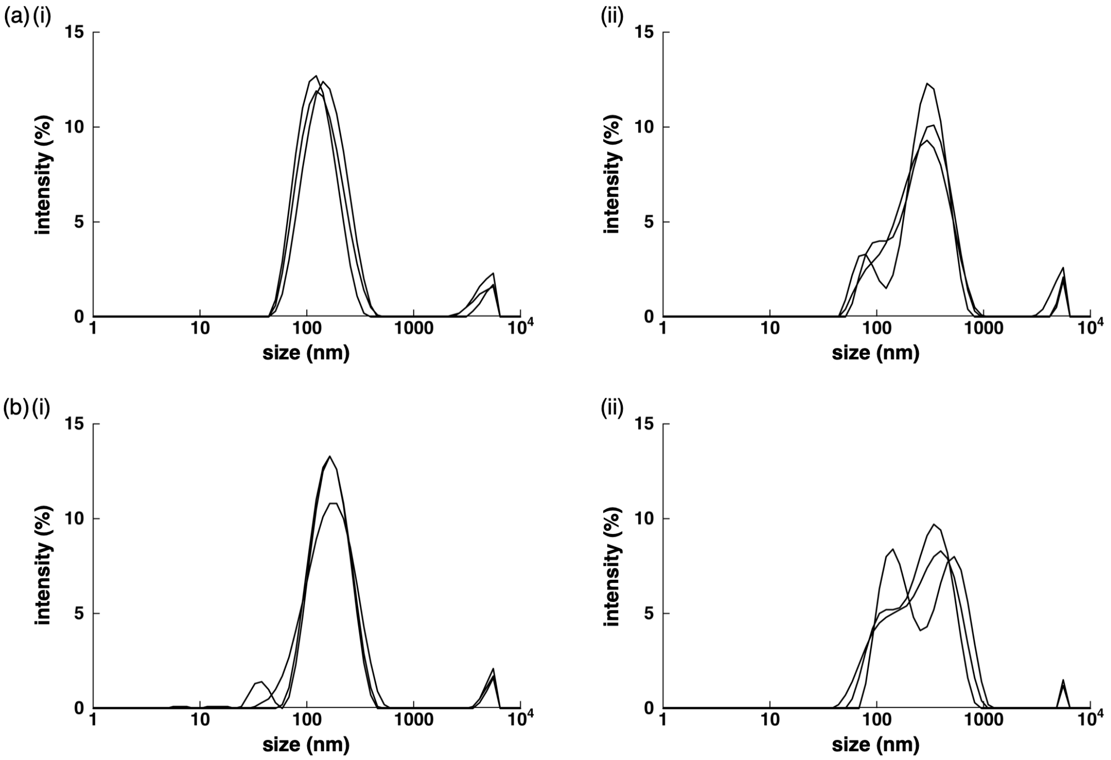

Figure S20 Dynamic light scattering (DLS) of (a) **1au**$^{+}$-Cl^{-} and (b) **1au**$^{+}$-PCCp^{-} in aqueous solutions ((i) 8 μM and (ii) 1.0 × 10^{-4} M) at 20 °C.

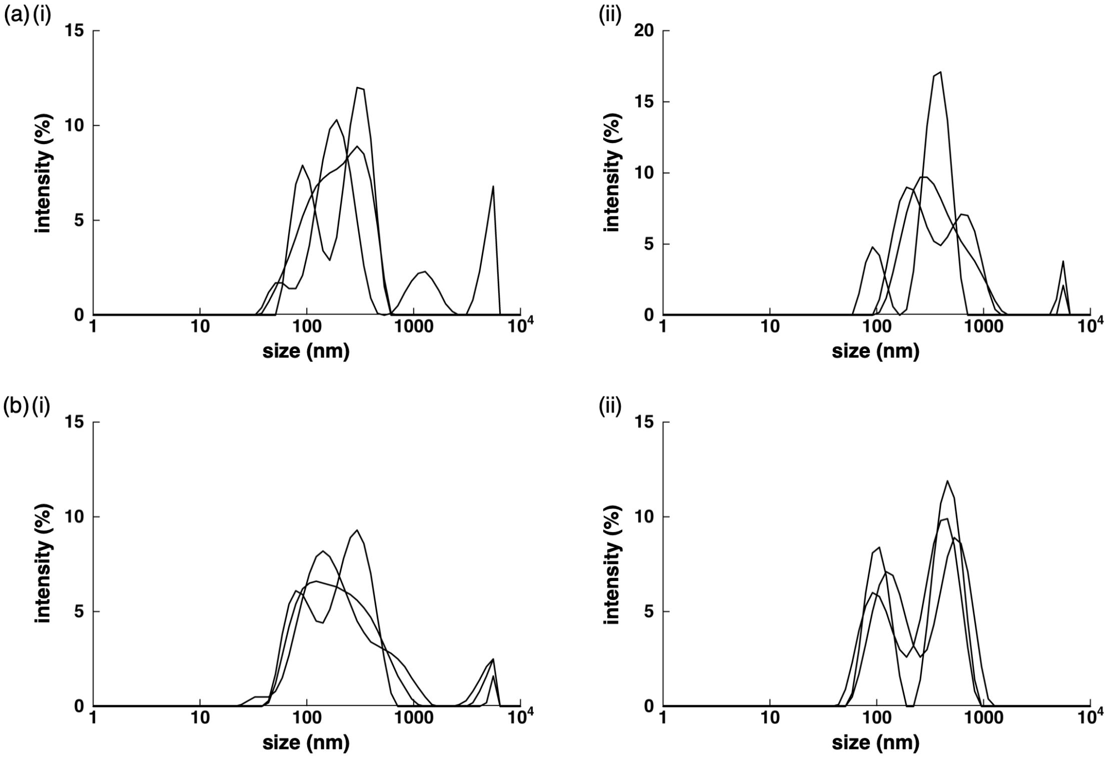

Figure S21 DLS of (a) **2au**$^{+}$-Cl^{-} and (b) **2au**$^{+}$-PCCp^{-} in aqueous solutions ((i) 8 μM and (ii) 1.0 × 10^{-4} M) at 20 °C.

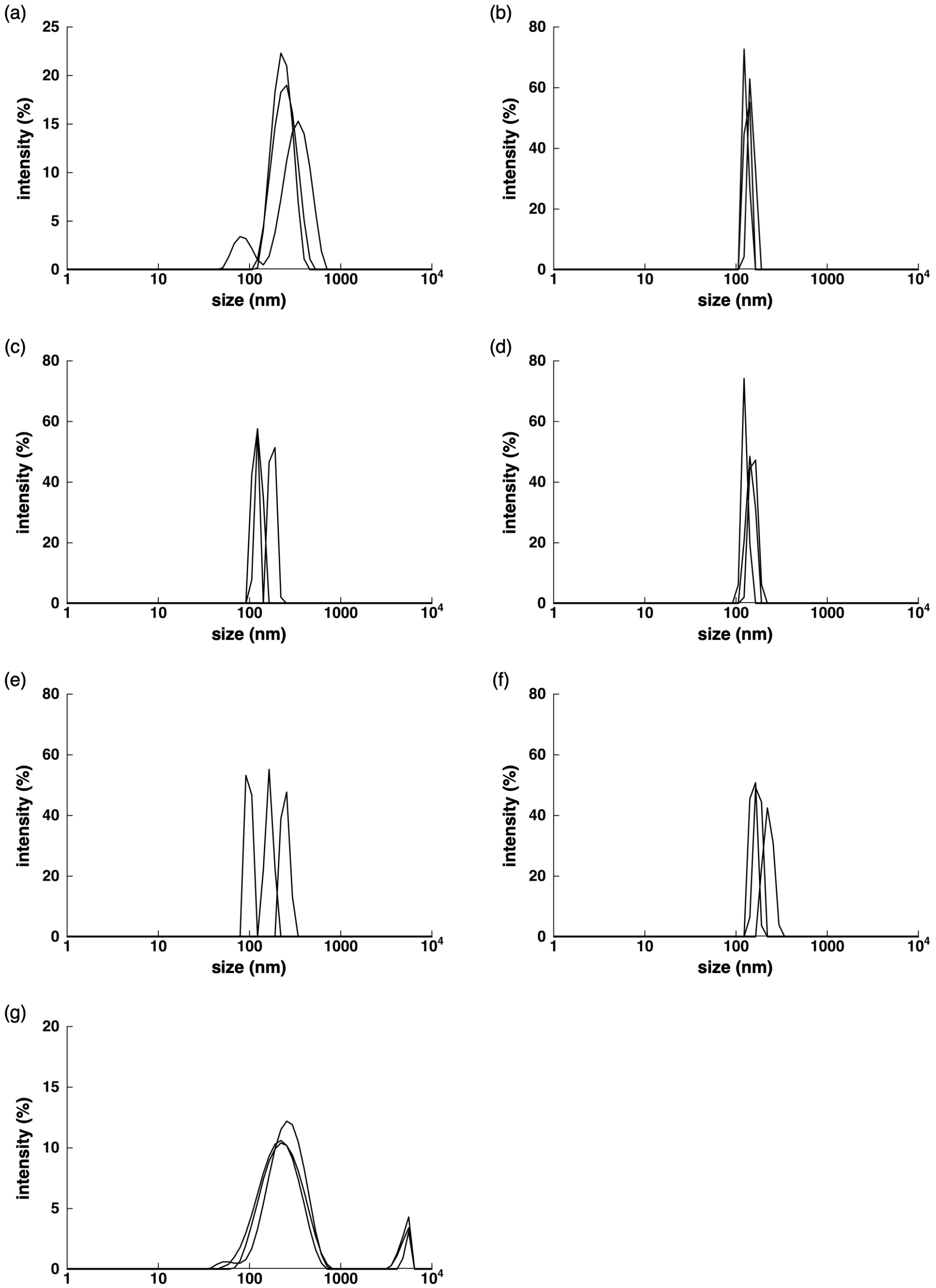

Figure S22 VT DLS changes of **1au**$^{+}$-Cl^{-} in aqueous solutions (8 μM) at (a) 20 °C, (b) 40 °C, (c) 60 °C, (d) 80 °C, (e) 60 °C, (f) 40 °C, and (g) 20 °C upon (a–d) heating and (e–g) cooling.

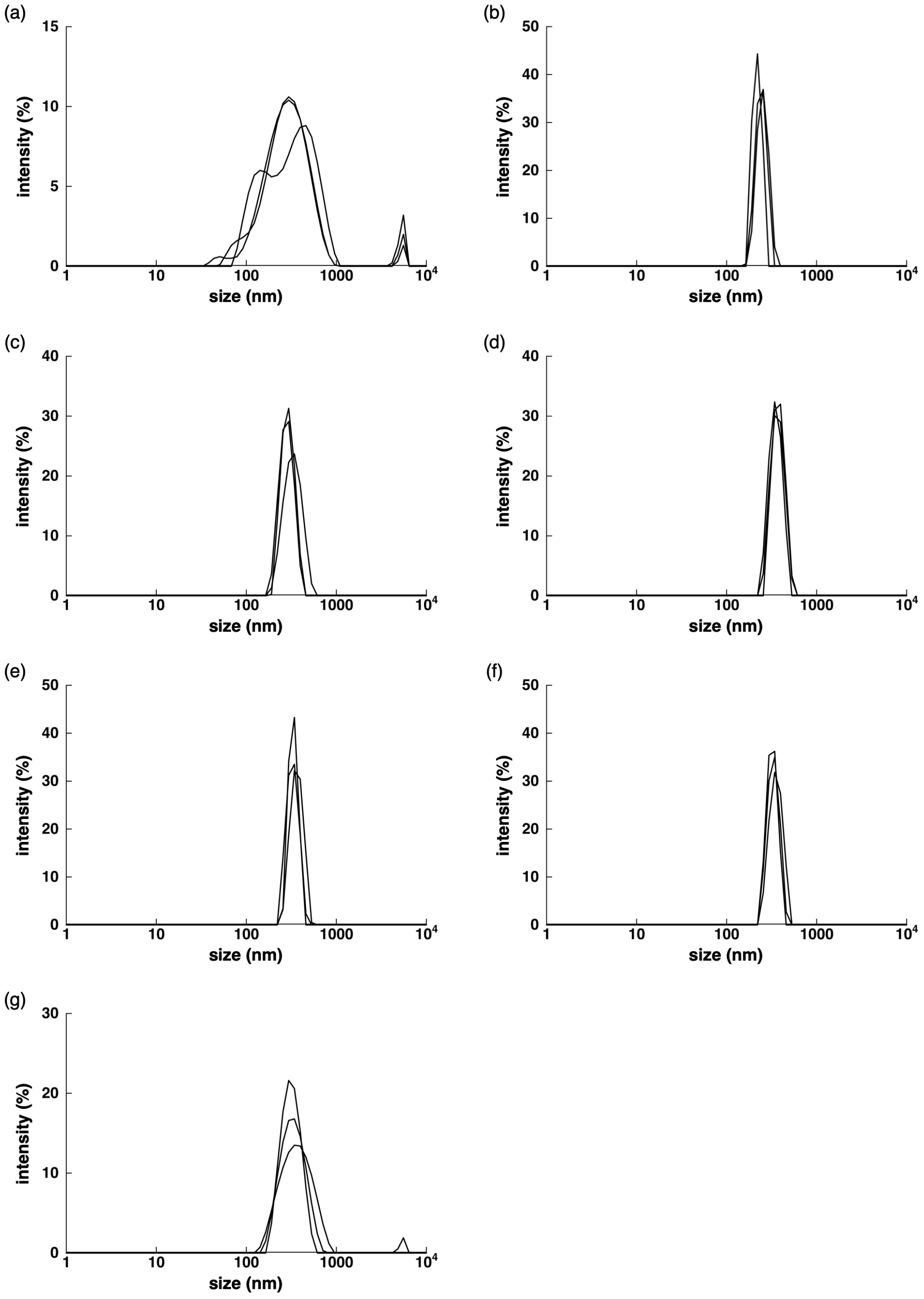

Figure S23 VT DLS changes of **1au**$^+$-Cl$^-$ in aqueous solutions (1.0×10^{-4} M) at (a) 20 °C, (b) 40 °C, (c) 60 °C, (d) 80 °C, (e) 60 °C, (f) 40 °C, and (g) 20 °C upon (a–d) heating and (e–g) cooling.

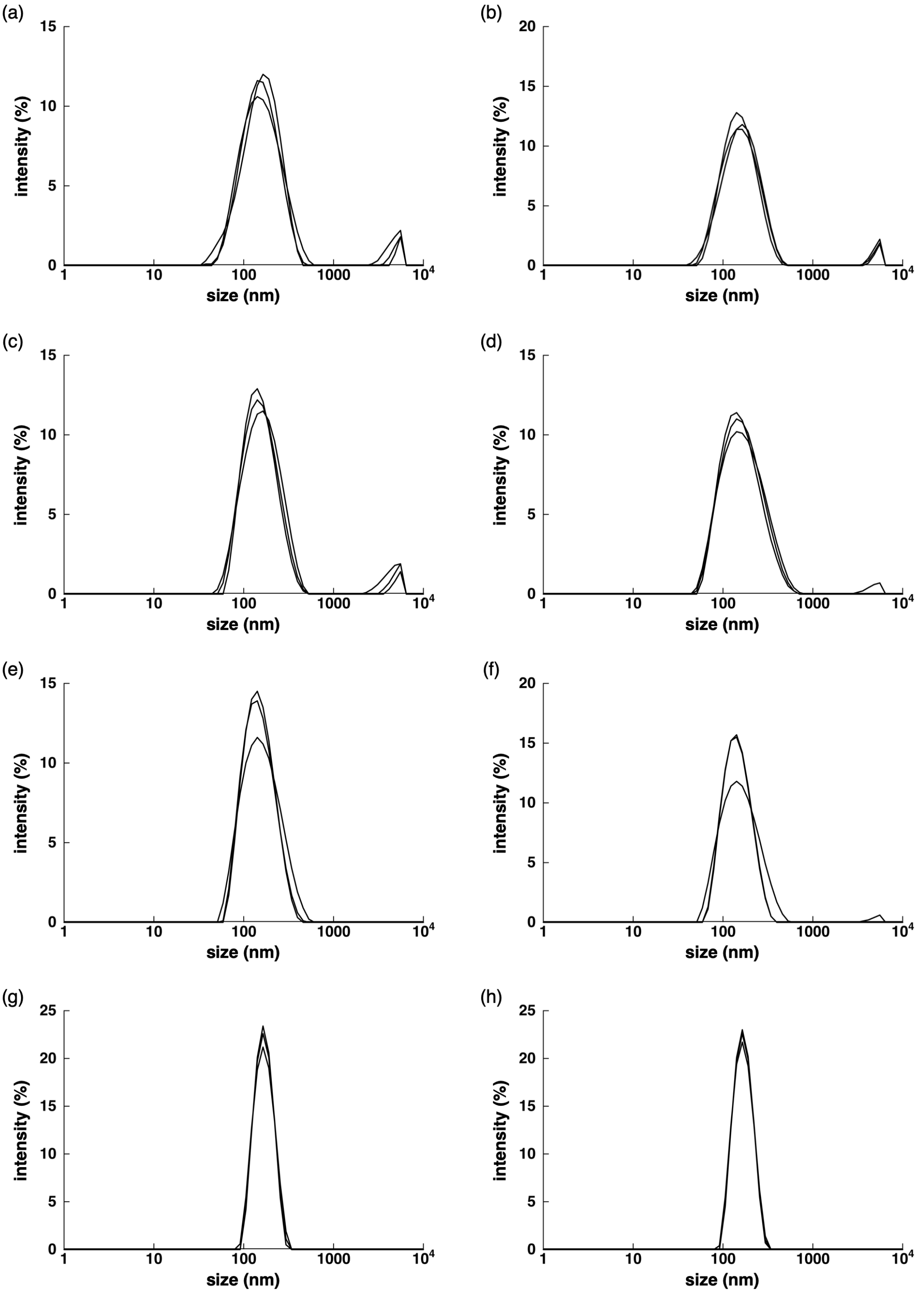

Figure S24 VT DLS changes of **1au**$^+$-PCCp$^-$ in aqueous solutions (8 μM) at (a) 20 °C, (b) 30 °C, (c) 40 °C, (d) 50 °C, (e) 60 °C, (f) 70 °C, (g) 80 °C, (h) 70 °C, (i) 60 °C, (j) 50 °C, (k) 40 °C, (l) 30 °C, and (m) 20 °C upon (a–g) heating and (h–m) cooling.

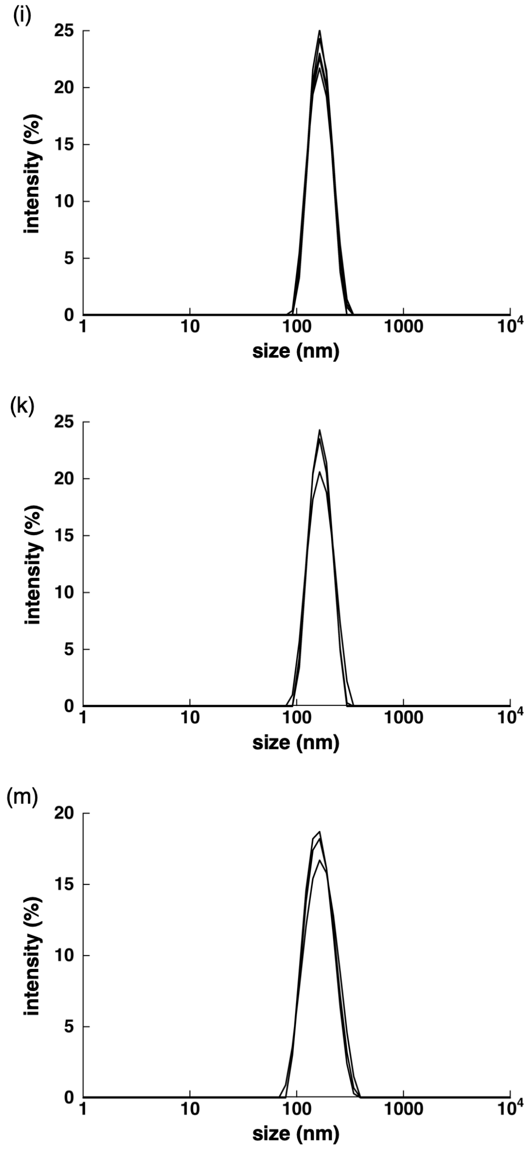

Figure S24 (Continued)

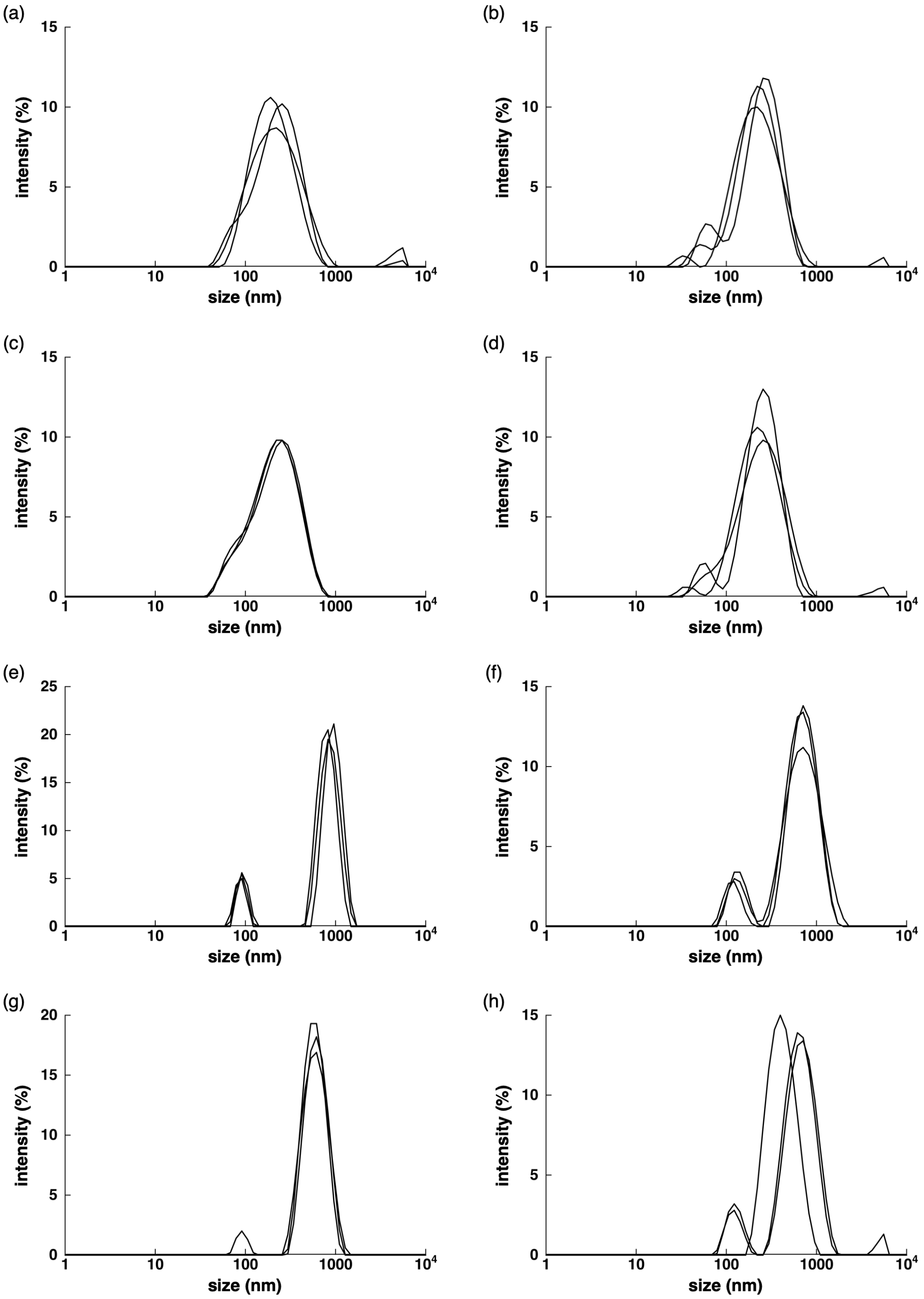

Figure S25 VT DLS changes of **1au**$^+$-PCCp$^-$ in aqueous solutions (1.0 × 10^{-4} M) at (a) 20 °C, (b) 30 °C, (c) 40 °C, (d) 50 °C, (e) 60 °C, (f) 70 °C, (g) 80 °C, (h) 70 °C, (i) 60 °C, (j) 50 °C, (k) 40 °C, (l) 30 °C, and (m) 20 °C upon (a–g) heating and (h–m) cooling.

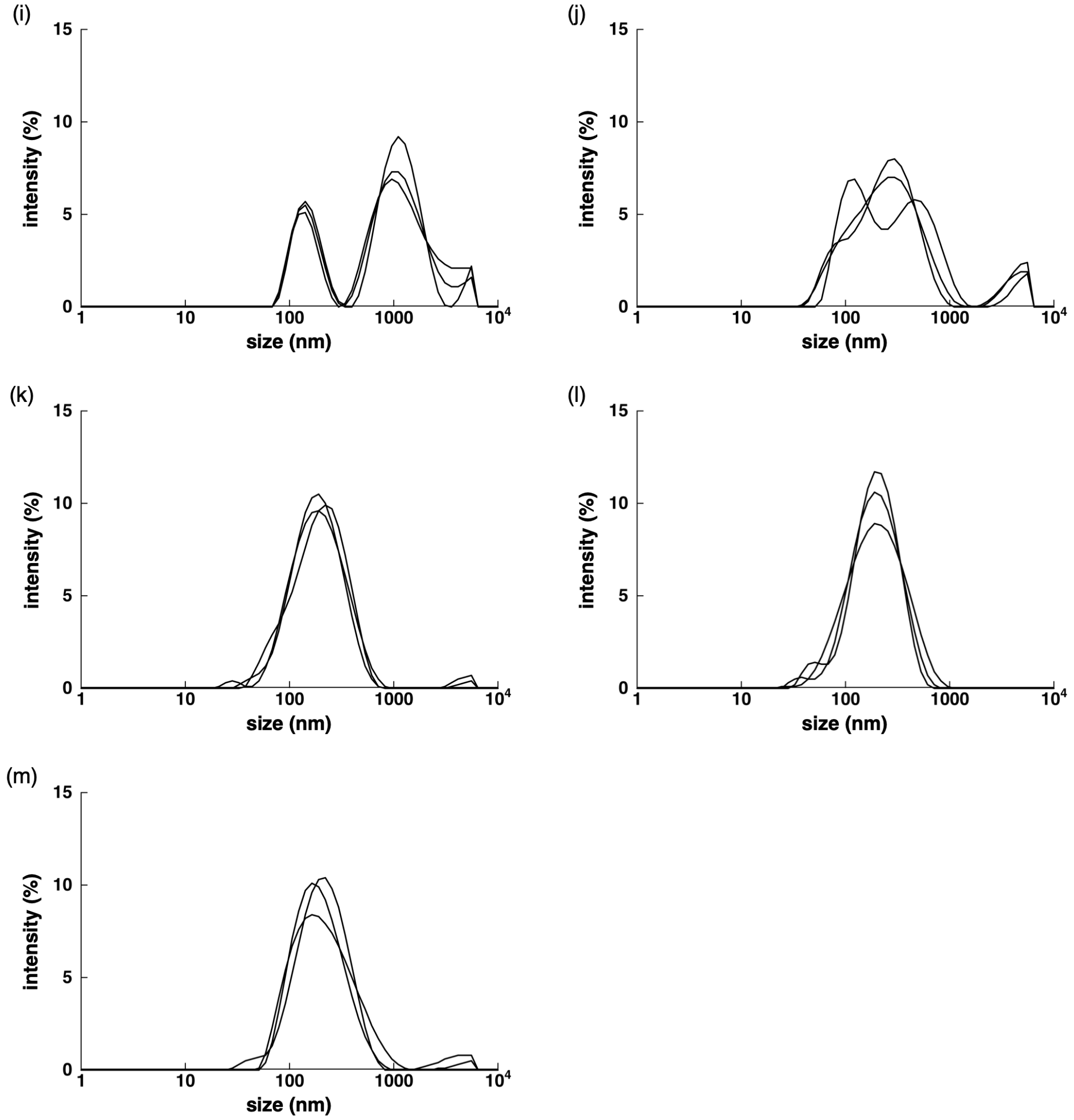

Figure S25 (Continued)

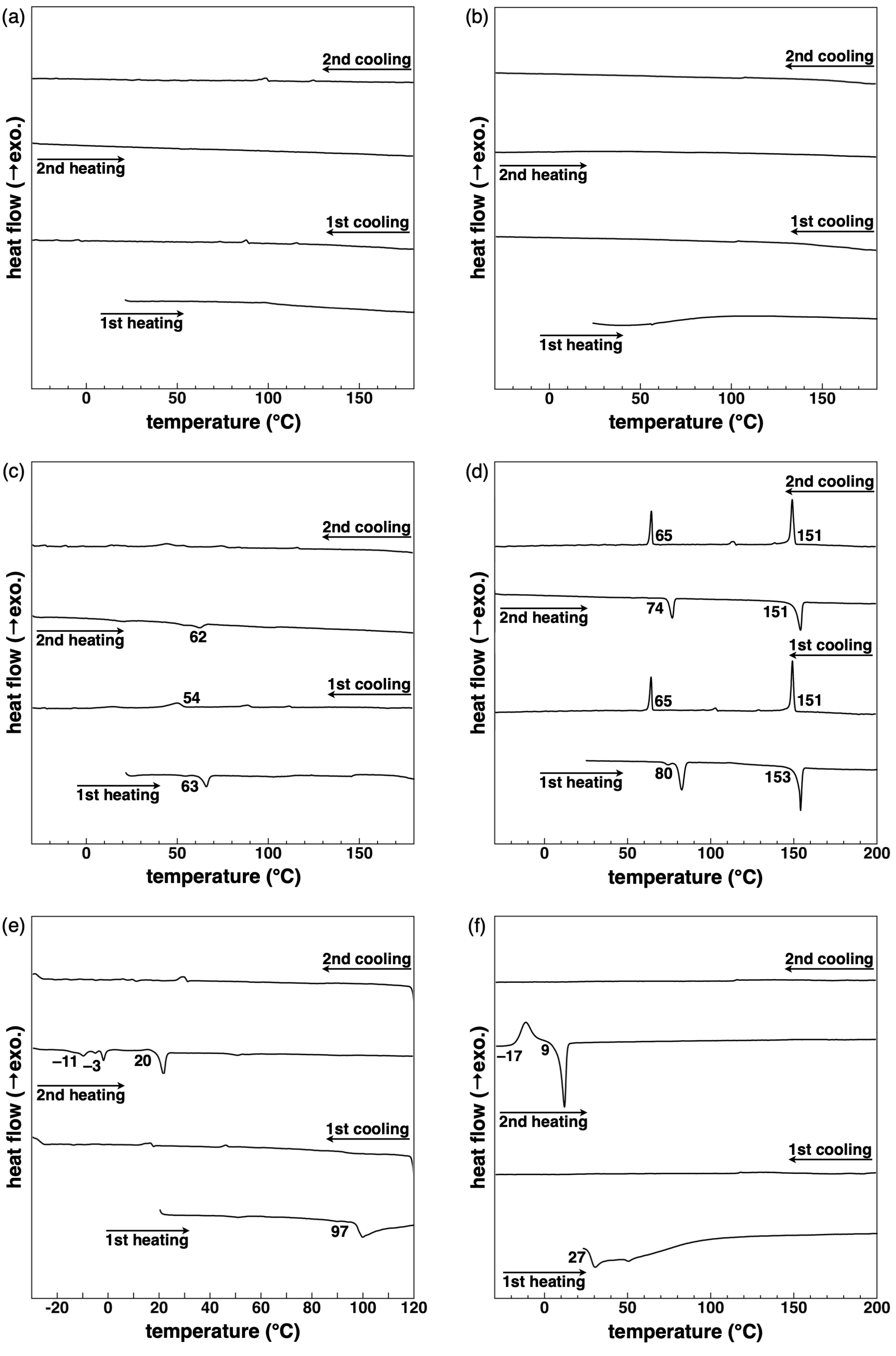

Figure S26 DSC thermograms of (a) **1**, (b) **2**, (c) **1au**$^{+}$-Cl^{-}, (d) **1au**$^{+}$-PCCp^{-}, (e) **2au**$^{+}$-Cl^{-}, and (f) **2au**$^{+}$-PCCp^{-}. Onset temperatures (°C) of phase transitions are labeled although some peaks are weak. In this figure (the cooling processes) and following figures, unidentified peaks derived from machine operation were observed.

Table S1 Summarized DSC data of transition temperatures and enthalpies (Δ*H* in kJ/mol) for **1au**$^+$-PCCp$^-$ and **2au**$^+$-PCCp$^-$.

	temperature (°C)	Δ*H* (kJ/mol)
**1au**$^+$-PCCp$^-$	80 (1st heating)	–14.2
	153 (1st heating)	–15.4
	151 (1st cooling)	15.6
	65 (1st cooling)	7.9
	74 (2nd heating)	–8.4
	151 (2nd heating)	–13.7
	151 (2nd cooling)	14.2
	65 (2nd cooling)	7.3
**2au**$^+$-PCCp$^-$	27 (1st heating)	–62.0
	–17 (2nd heating)	15.4
	9 (2nd heating)	–22.7

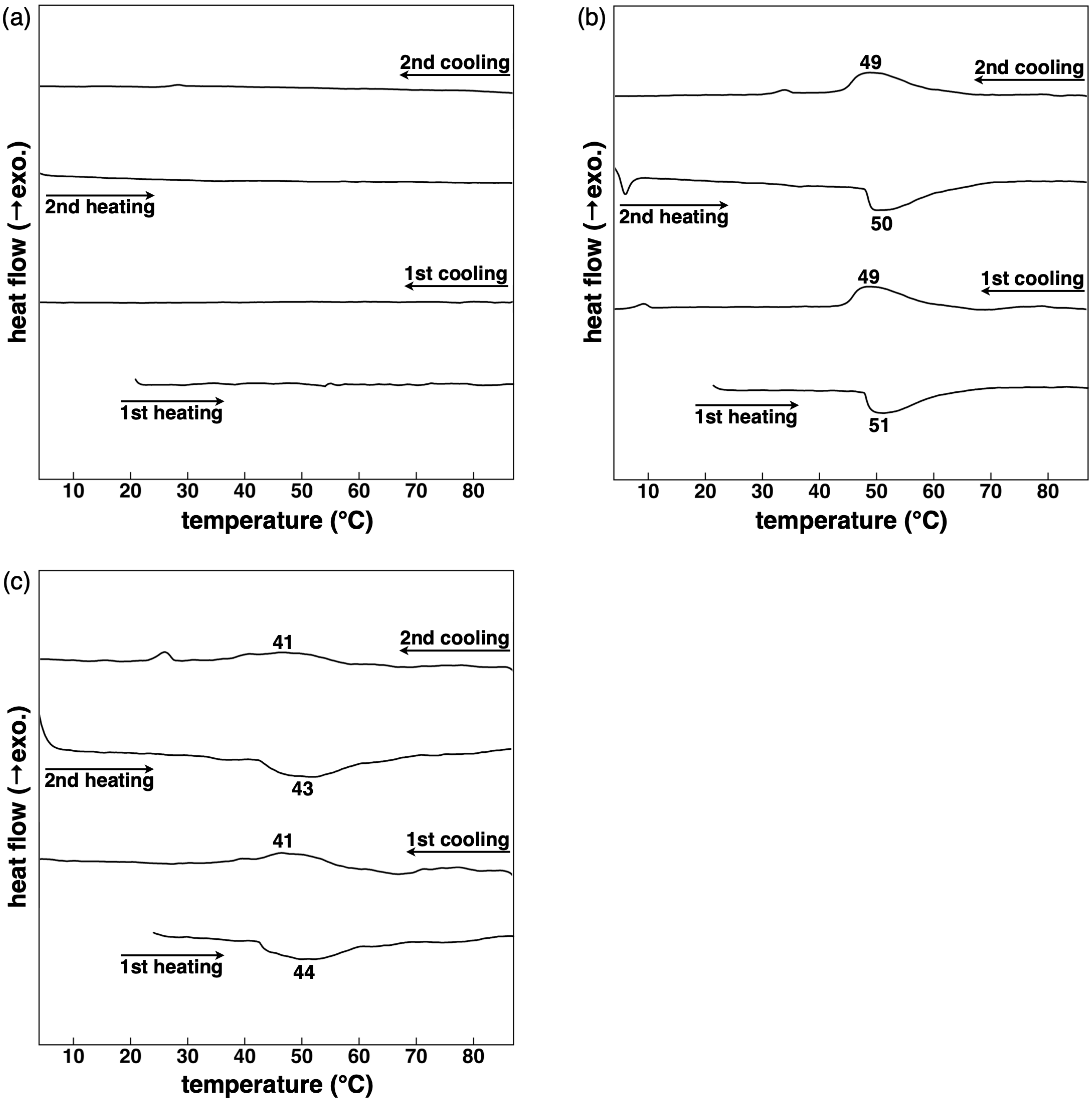

Figure S27 DSC thermograms of (a) $\mathbf{1}_{70\%}$ (the percentage (w/w) of **1** to the total amount containing **1** and water: 70%), (b) $\mathbf{1}_{50\%}$ (the percentage (w/w): 50%), and (c) $\mathbf{1}_{20\%}$ (the percentage (w/w): 20%).

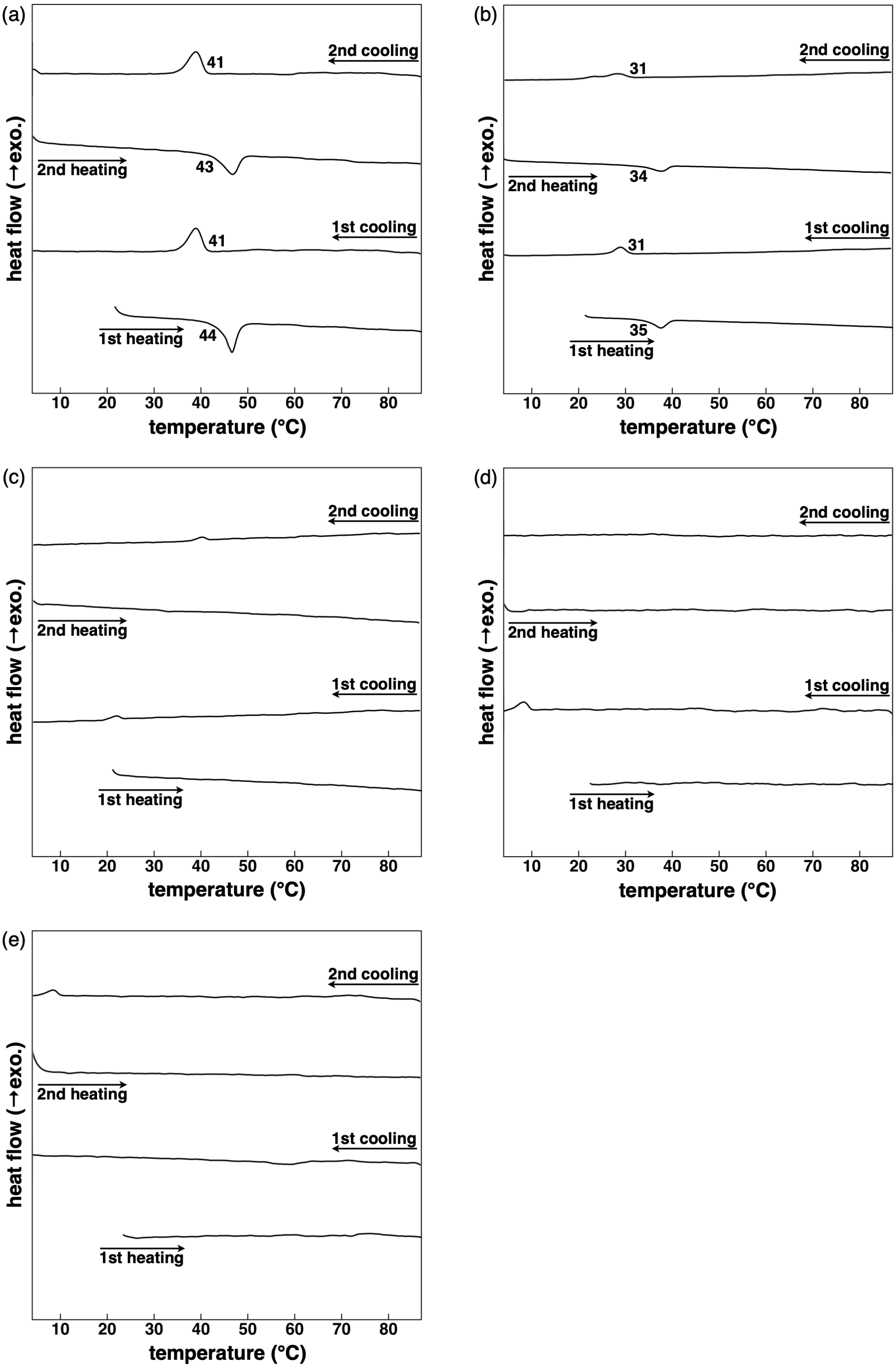

Figure S28 DSC thermograms of (a) **1au**$^+$-Cl$^-_{75\%}$ (the percentage (w/w) of **1au**$^+$-Cl$^-$ to the total amount containing **1au**$^+$-Cl$^-$ and water: 75%), (b) **1au**$^+$-Cl$^-_{70\%}$ (the percentage (w/w): 70%), (c) **1au**$^+$-Cl$^-_{60\%}$ (the percentage (w/w): 60%), (d) **1au**$^+$-Cl$^-_{50\%}$ (the percentage (w/w): 50%), and (e) **1au**$^+$-Cl$^-_{20\%}$ (the percentage (w/w): 20%).

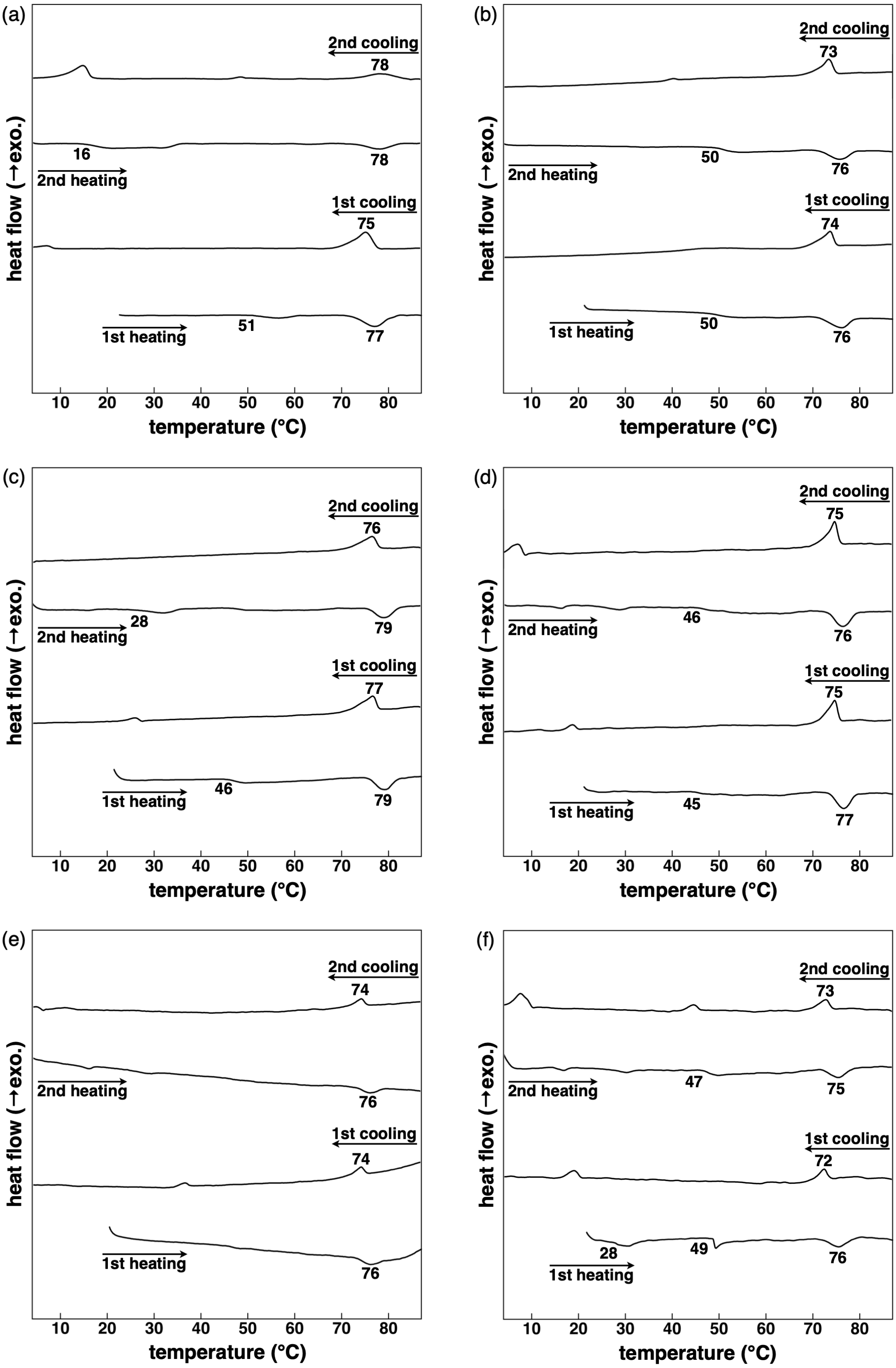

Figure S29 DSC thermograms of (a) **1au**$^{+}$-PCCp^{-}$_{70\%}$ (the percentage (w/w) of **1au**$^{+}$-PCCp^{-} to the total amount containing **1au**$^{+}$-PCCp^{-} and water: 70%), (b) **1au**$^{+}$-PCCp^{-}$_{60\%}$ (the percentage (w/w): 60%), (c) **1au**$^{+}$-PCCp^{-}$_{50\%}$ (the percentage (w/w): 50%), (d) **1au**$^{+}$-PCCp^{-}$_{40\%}$ (the percentage (w/w): 40%), (e) **1au**$^{+}$-PCCp^{-}$_{30\%}$ (the percentage (w/w): 30%), (f) **1au**$^{+}$-PCCp^{-}$_{20\%}$ (the percentage (w/w): 20%), (g) **1au**$^{+}$-PCCp^{-}$_{10\%}$ (the percentage (w/w): 10%), and (h) **1au**$^{+}$-PCCp^{-}$_{1\%}$ (the percentage (w/w): 1%).

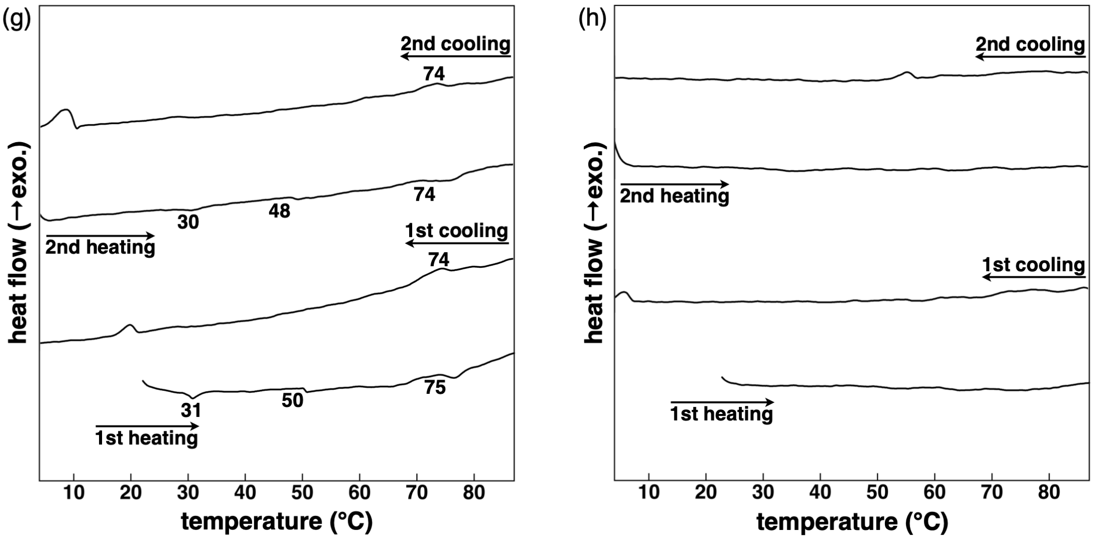

Figure S29 (Continued)

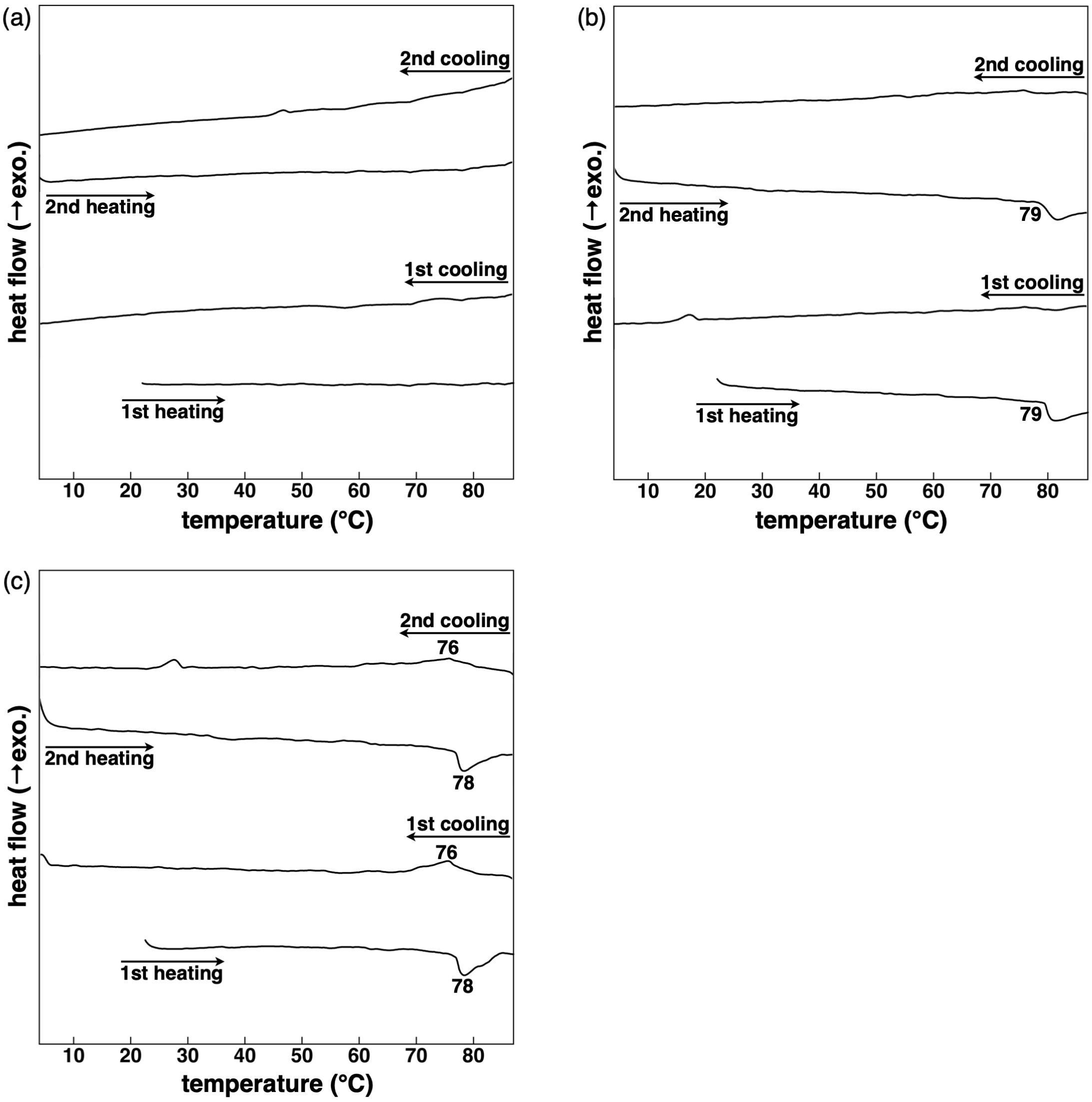

Figure S30 DSC thermograms of (a) **2**$_{70\%}$ (the percentage (w/w) of **2** to the total amount containing **2** and water: 70%), (b) **2**$_{50\%}$ (the percentage (w/w): 50%), and (c) **2**$_{20\%}$ (the percentage (w/w): 20%).

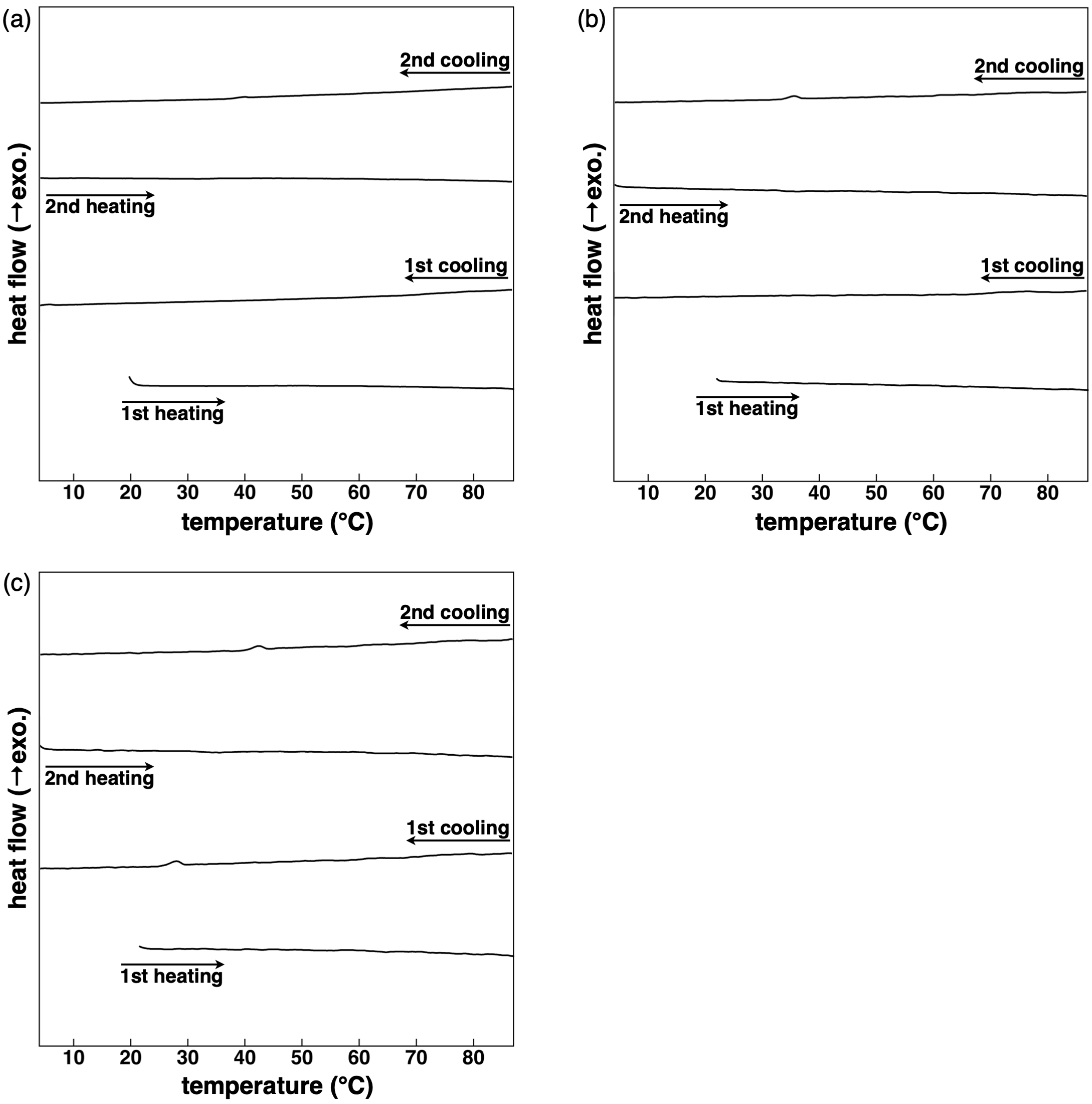

Figure S31 DSC thermograms of (a) **2au**$^{+}$-Cl$^{-}_{70\%}$ (the percentage (w/w) of **2au**$^{+}$-Cl^{-} to the total amount containing **2au**$^{+}$-Cl^{-} and water: 70%), (b) **2au**$^{+}$-Cl$^{-}_{50\%}$ (the percentage (w/w): 50%), and (c) **2au**$^{+}$-Cl$^{-}_{20\%}$ (the percentage (w/w): 20%).

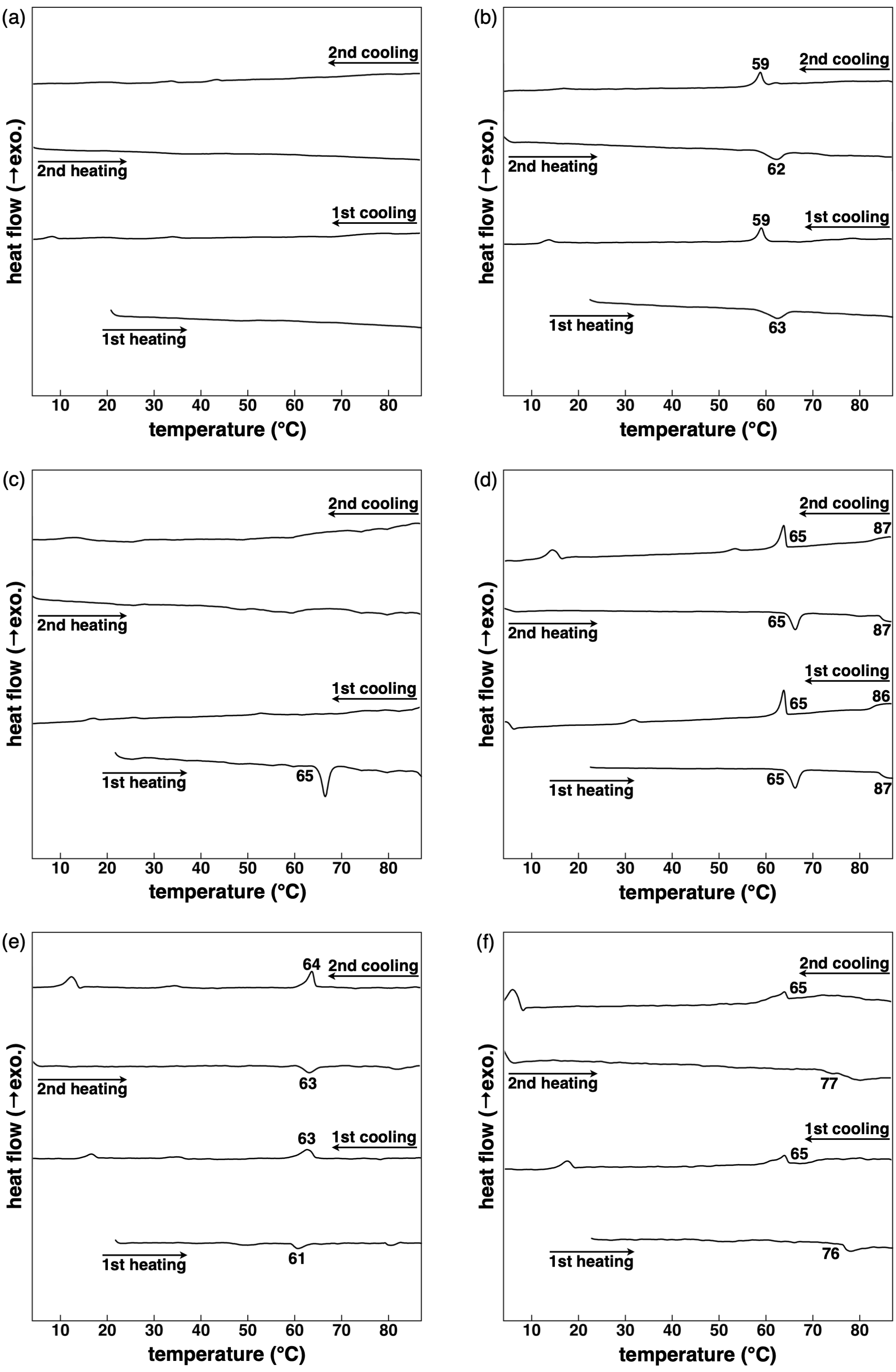

Figure S32 DSC thermograms of (a) **2au**$^+$-PCCp$^-_{80\%}$ (the percentage (w/w) of **2au**$^+$-PCCp$^-$ to the total amount containing **2au**$^+$-PCCp$^-$ and water: 80%), (b) **2au**$^+$-PCCp$^-_{70\%}$ (the percentage (w/w): 70%), (c) **2au**$^+$-PCCp$^-_{60\%}$ (the percentage (w/w): 60%), (d) **2au**$^+$-PCCp$^-_{50\%}$ (the percentage (w/w): 50%), (e) **2au**$^+$-PCCp$^-_{40\%}$ (the percentage (w/w): 40%), (f) **2au**$^+$-PCCp$^-_{30\%}$ (the percentage (w/w): 30%), (g) **2au**$^+$-PCCp$^-_{20\%}$ (the percentage (w/w): 20%), (h) **2au**$^+$-PCCp$^-_{10\%}$ (the percentage (w/w): 10%), and (i) **2au**$^+$-PCCp$^-_{1\%}$ (the percentage (w/w): 1%).

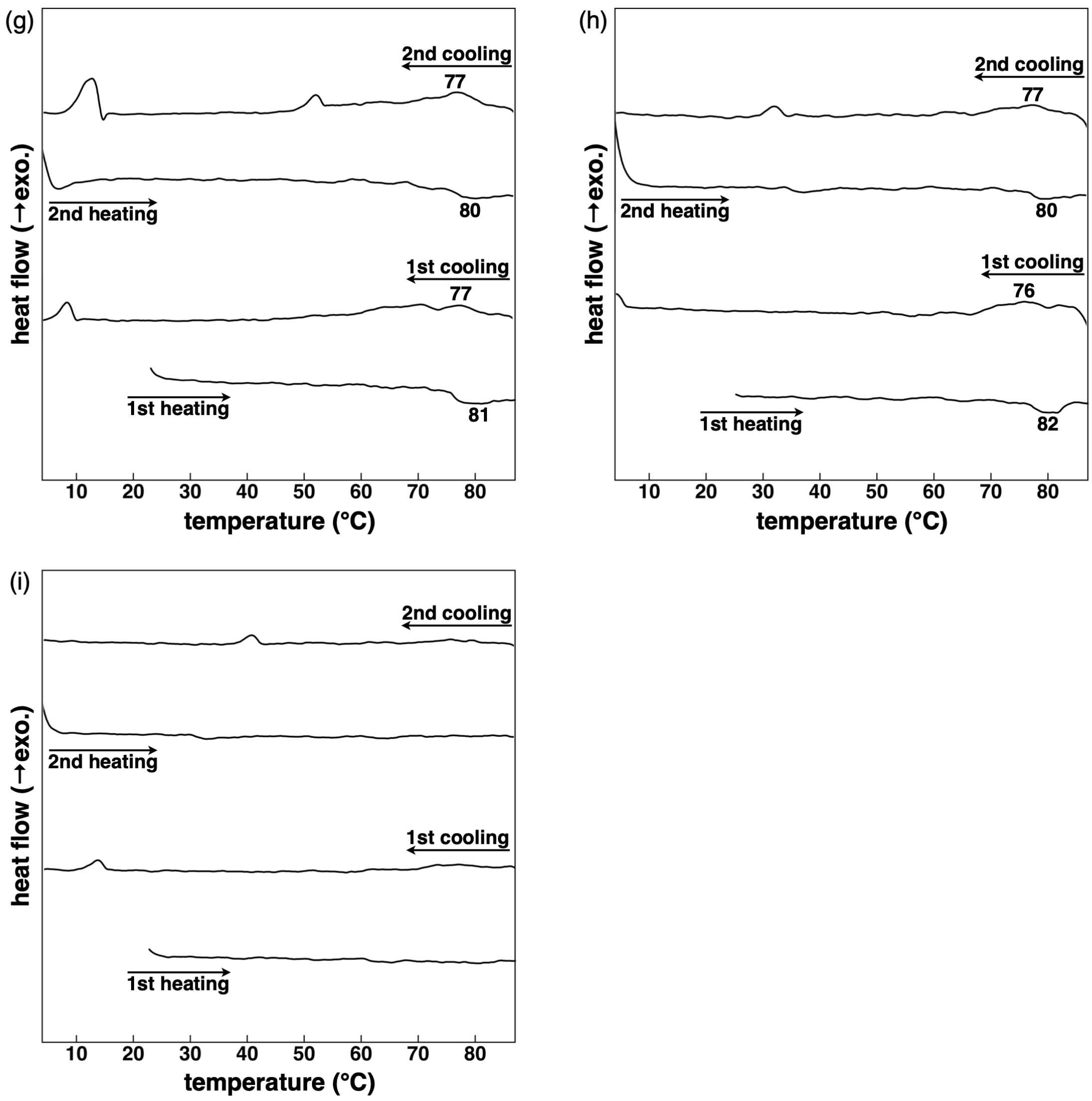

Figure S32 (Continued)

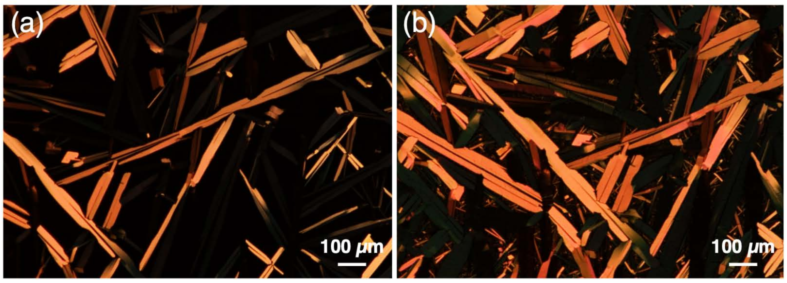

Figure S33 POM textures of **1au**$^{+}$-Cl^{-} at (a) 120 °C and (b) 20 °C upon cooling.

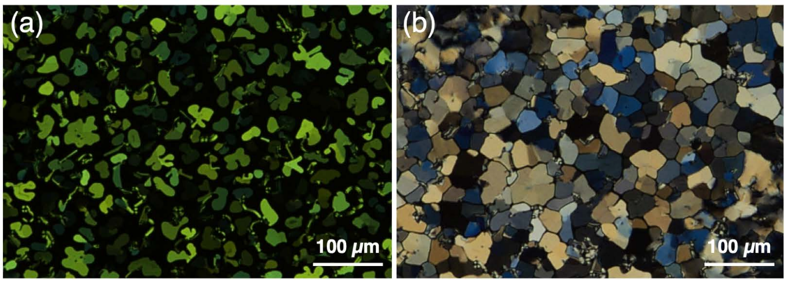

Figure S34 POM textures of **1au**$^{+}$-PCCp^{-} at (a) 150 °C and (b) 20 °C upon cooling.

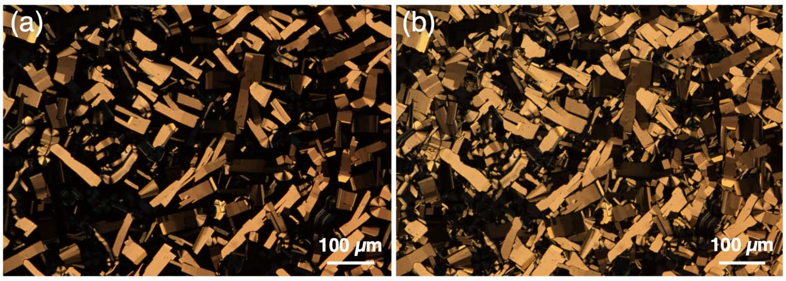

Figure S35 POM textures of **2au**$^+$-Cl$^-$ at (a) 80 °C and (b) 20 °C upon cooling.

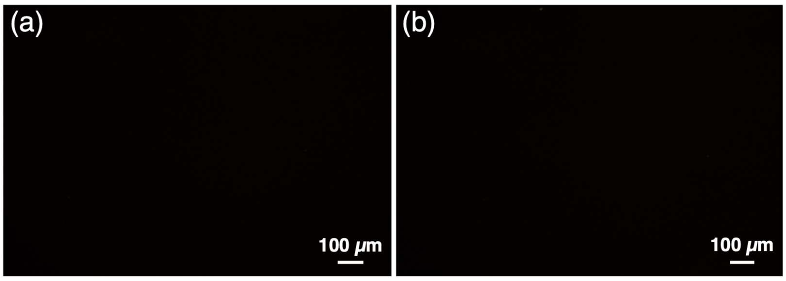

Figure S36 POM textures of **2au**$^+$-PCCp$^-$ at (a) 250 °C and (b) 20 °C upon cooling.

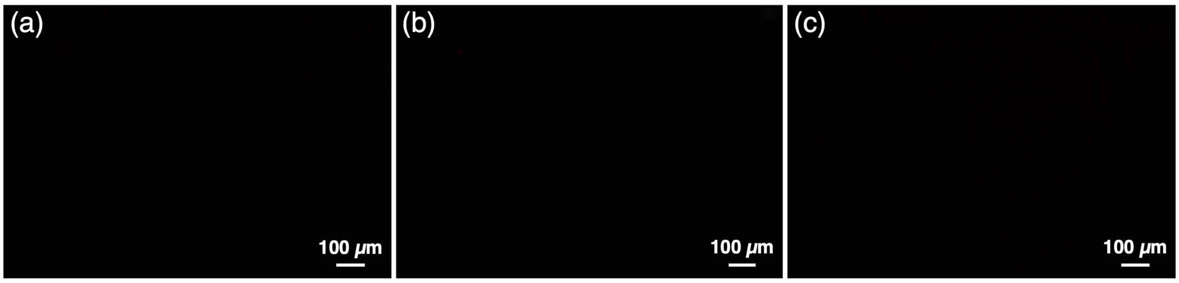

Figure S37 POM textures of $\mathbf{1}_{70\%}$ at (a) 20 °C and (b) 80 °C upon heating and (c) 20 °C upon cooling.

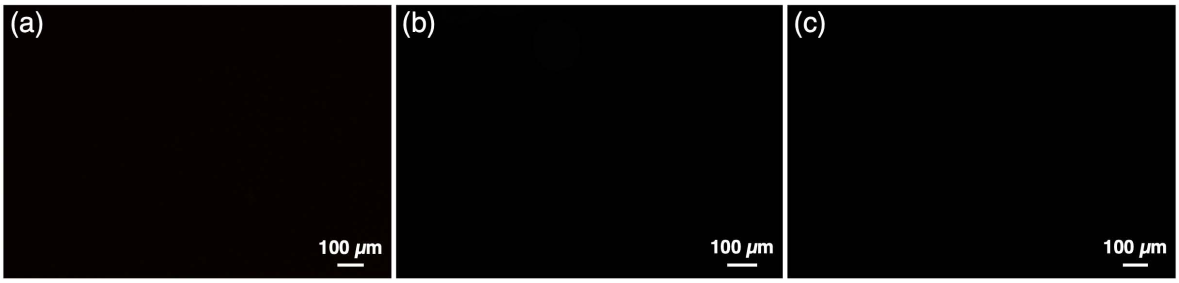

Figure S38 POM textures of $\mathbf{1}_{50\%}$ at (a) 20 °C and (b) 80 °C upon heating and (c) 20 °C upon cooling.

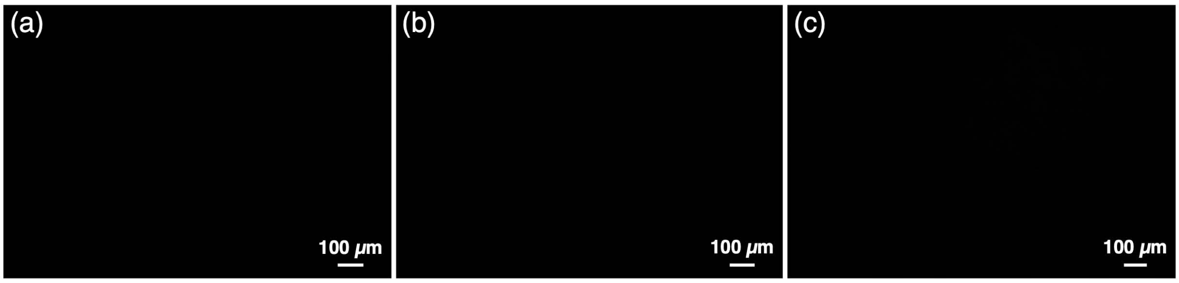

Figure S39 POM textures of $\mathbf{1}_{20\%}$ at (a) 20 °C and (b) 80 °C upon heating and (c) 20 °C upon cooling.

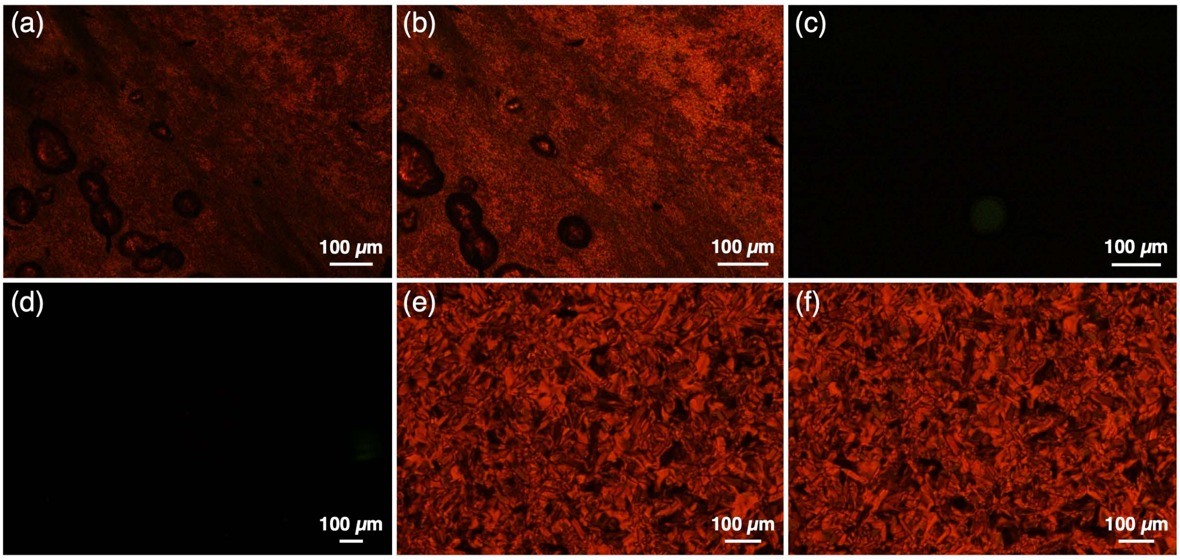

Figure S40 POM textures of **1au**$^+$-$Cl^-_{75\%}$ at (a) 20 °C, (b) 40 °C, (c) 60 °C, and (d) 80 °C upon heating and (e) 50 °C and (f) 20 °C upon cooling.

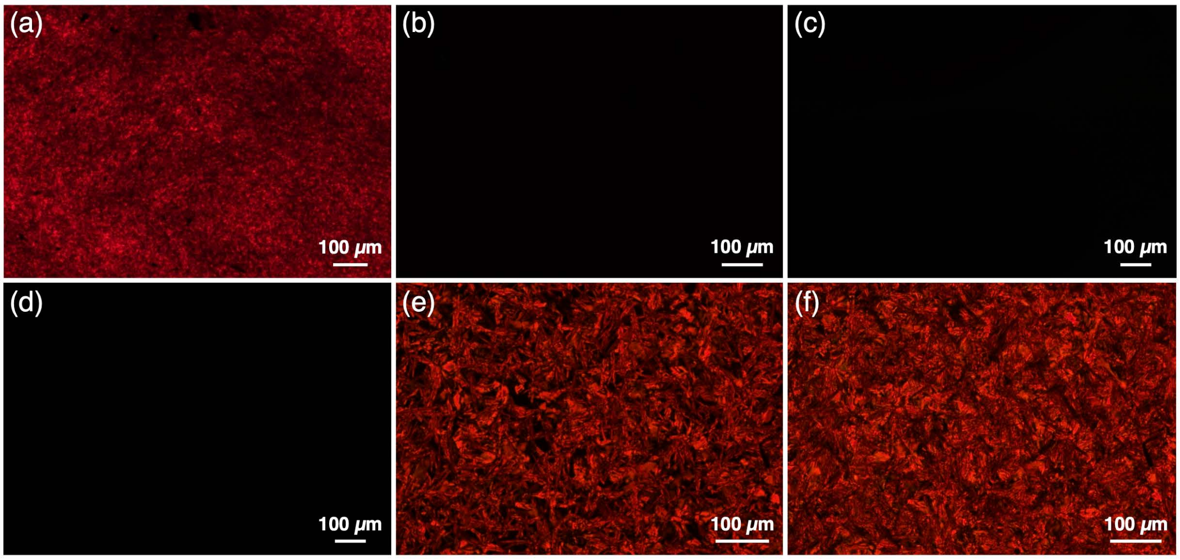

Figure S41 POM textures of **1au**$^+$-$Cl^-_{70\%}$ at (a) 20 °C, (b) 40 °C, and (c) 80 °C upon heating and (d) 40 °C, (e) 30 °C, and (f) 20 °C upon cooling.

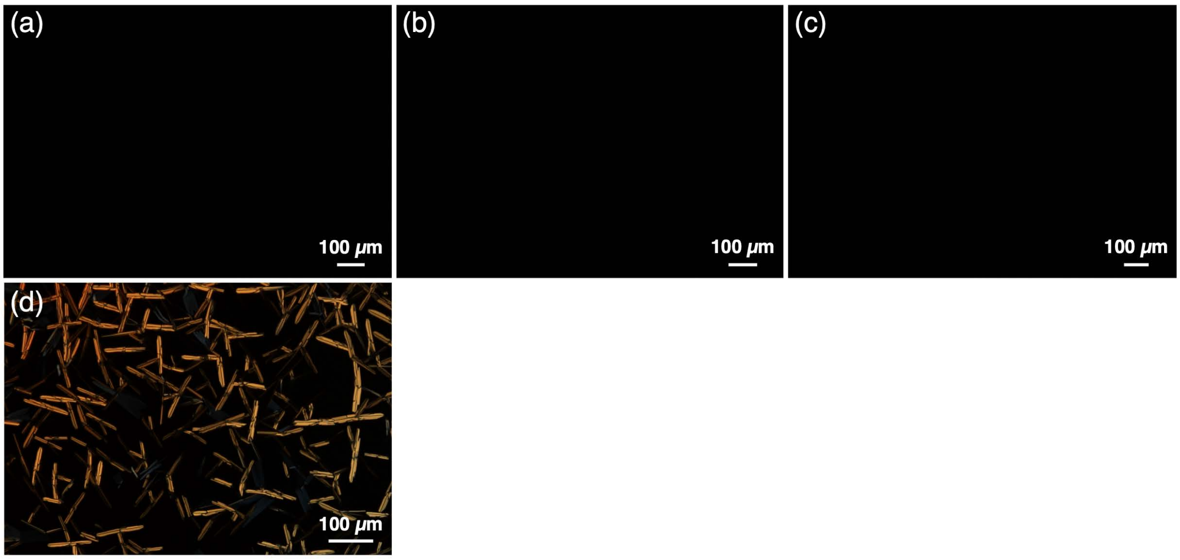

Figure S42 POM textures of **1au**$^{+}$-$Cl^{-}_{60\%}$ at (a) 20 °C and (b) 80 °C upon heating and (c) 40 °C and (d) 20 °C upon cooling. Upon cooling, the texture was observed due to inhomogeneous concentration distribution at 20 °C.

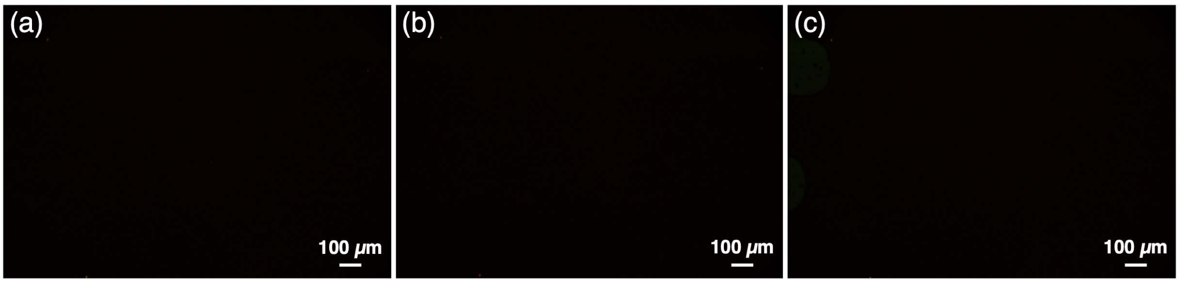

Figure S43 POM textures of **1au**$^{+}$-$Cl^{-}_{50\%}$ at (a) 20 °C and (b) 80 °C upon heating and (c) 20 °C upon cooling.

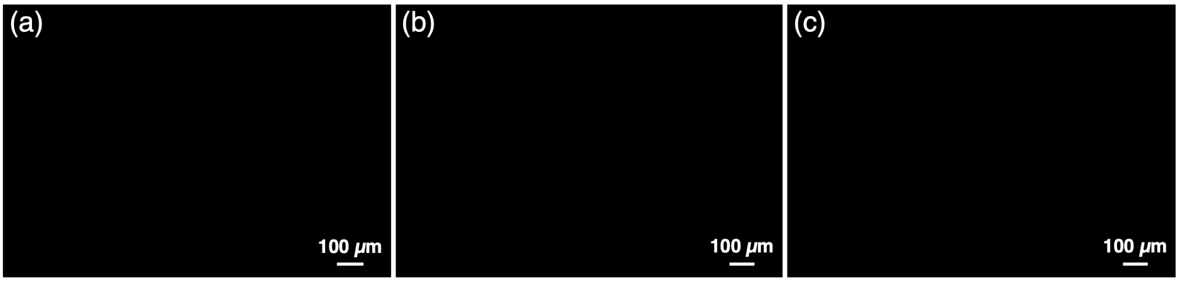

Figure S44 POM textures of **1au**$^{+}$-$Cl^{-}_{20\%}$ at (a) 20 °C and (b) 80 °C upon heating and (c) 20 °C upon cooling.

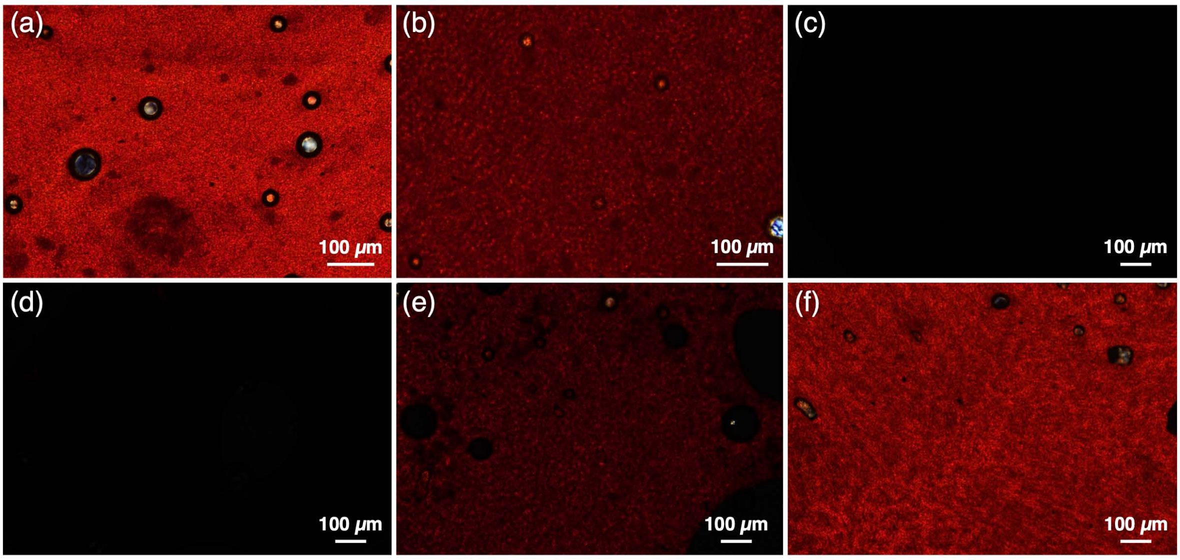

Figure S45 POM textures of **1au**$^+$-PCCp$^-_{70\%}$ at (a) 20 °C, (b) 60 °C, (c) 80 °C, and (d) 90 °C upon heating and (e) 70 °C and (f) 20 °C upon cooling.

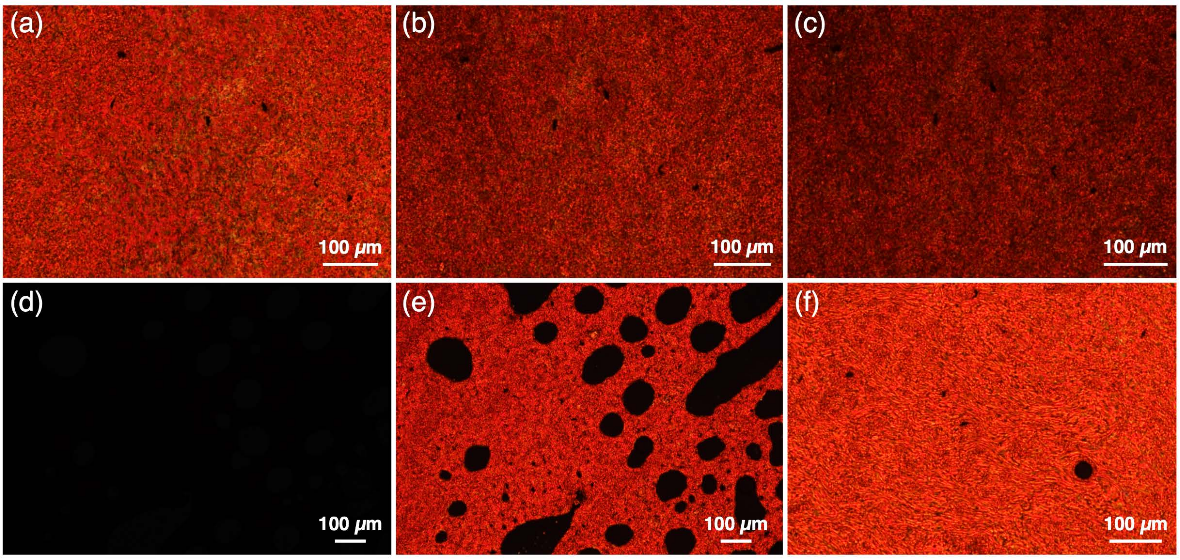

Figure S46 POM textures of **1au**$^+$-PCCp$^-_{60\%}$ at (a) 20 °C, (b) 60 °C, (c) 70 °C, and (d) 90 °C upon heating and (e) 70 °C and (f) 20 °C upon cooling.

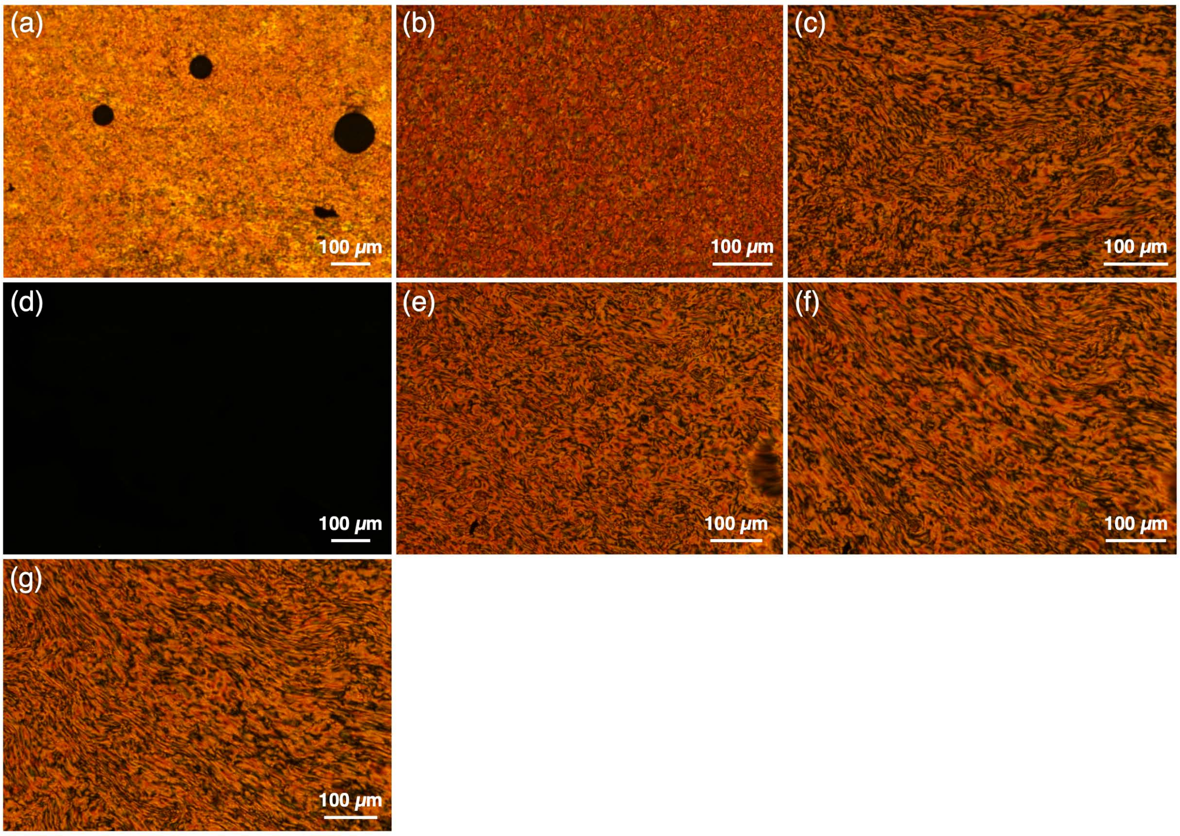

Figure S47 POM textures of **1au**$^{+}$-PCCp$^{-}_{50\%}$ at (a) 20 °C, (b) 40 °C, (c) 70 °C, and (d) 90 °C upon heating and (e) 70 °C , (f) 30 °C, and (g) 20 °C upon cooling.

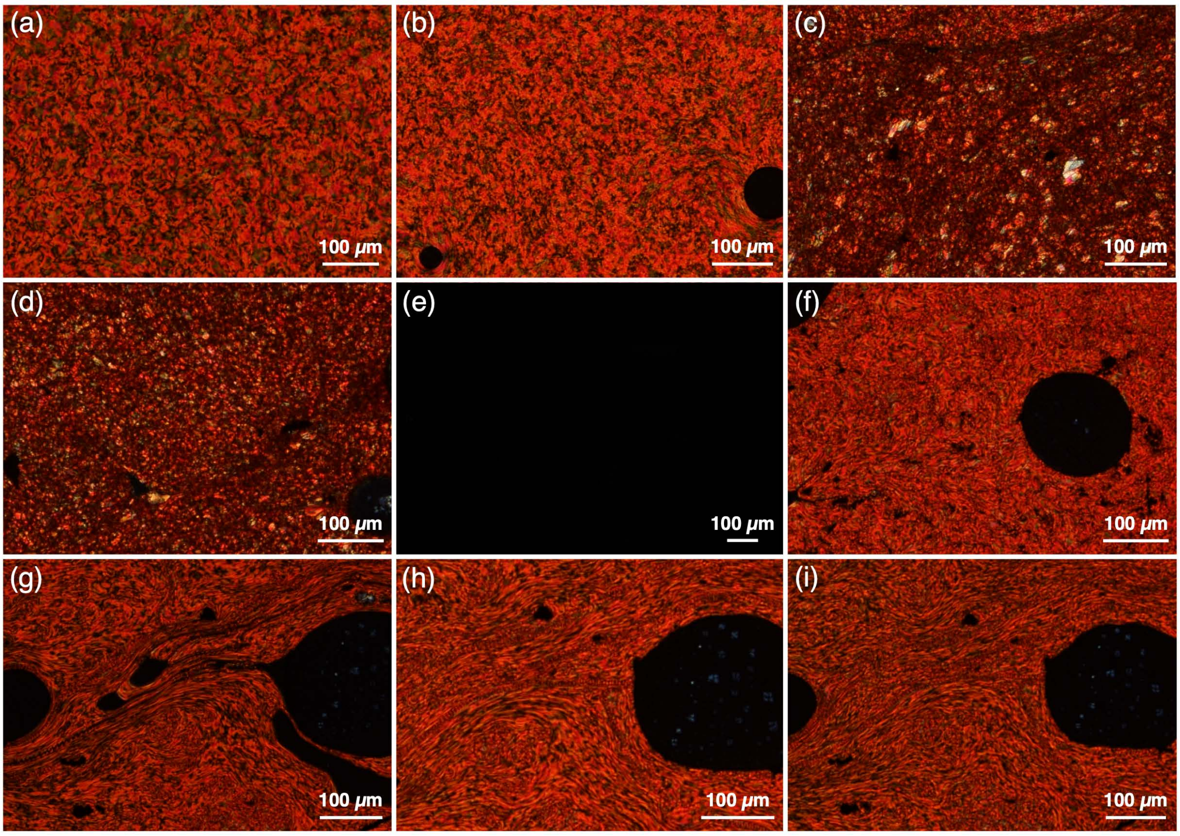

Figure S48 POM textures of **1au**$^{+}$-PCCp$^{-}_{40\%}$ at (a) 20 °C, (b) 40 °C, (c) 60 °C, (d) 70 °C, and (e) 90 °C upon heating and (f) 70 °C, (g) 60 °C, (h) 40 °C, and (i) 20 °C upon cooling.

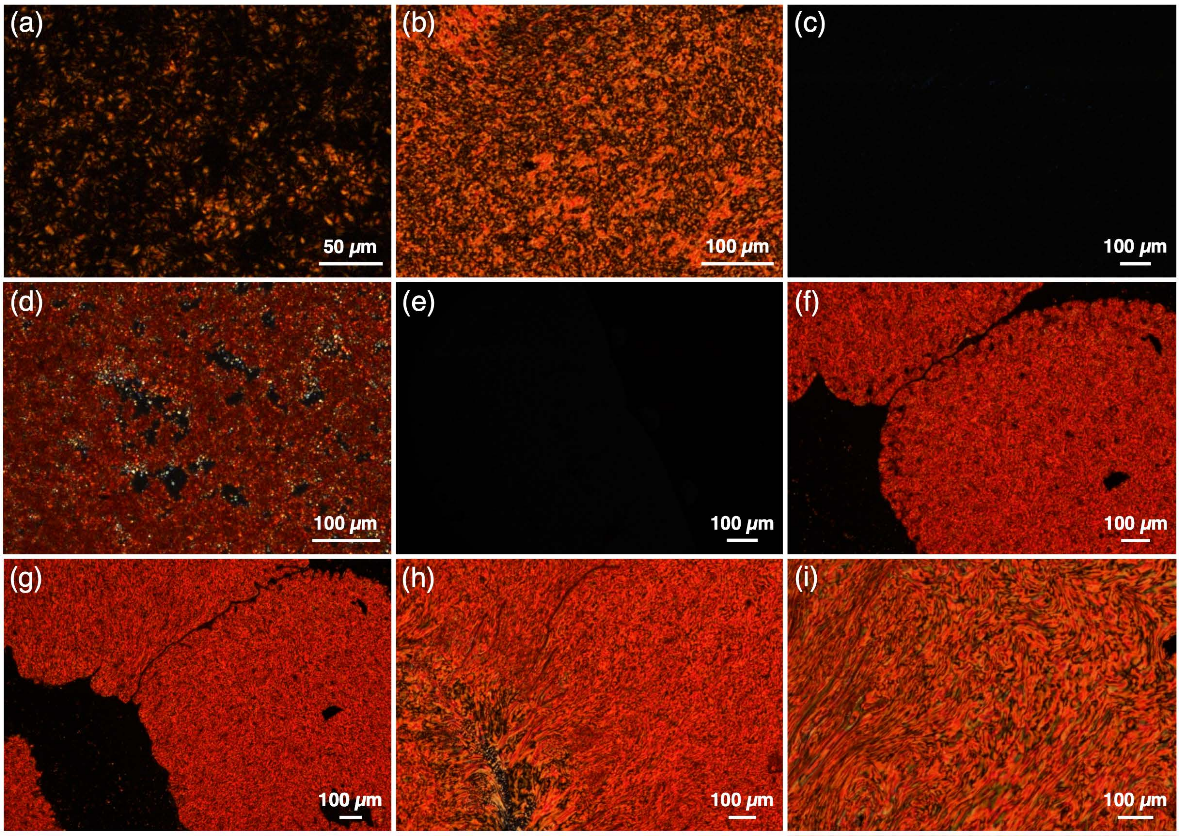

Figure S49 POM textures of **1au**$^{+}$-PCCp$^{-}_{30\%}$ at (a) 20 °C, (b) 40 °C, (c) 60 °C, (d) 70 °C, and (e) 90 °C upon heating and (f) 70 °C, (g) 60 °C, (h) 40 °C, and (i) 20 °C upon cooling.

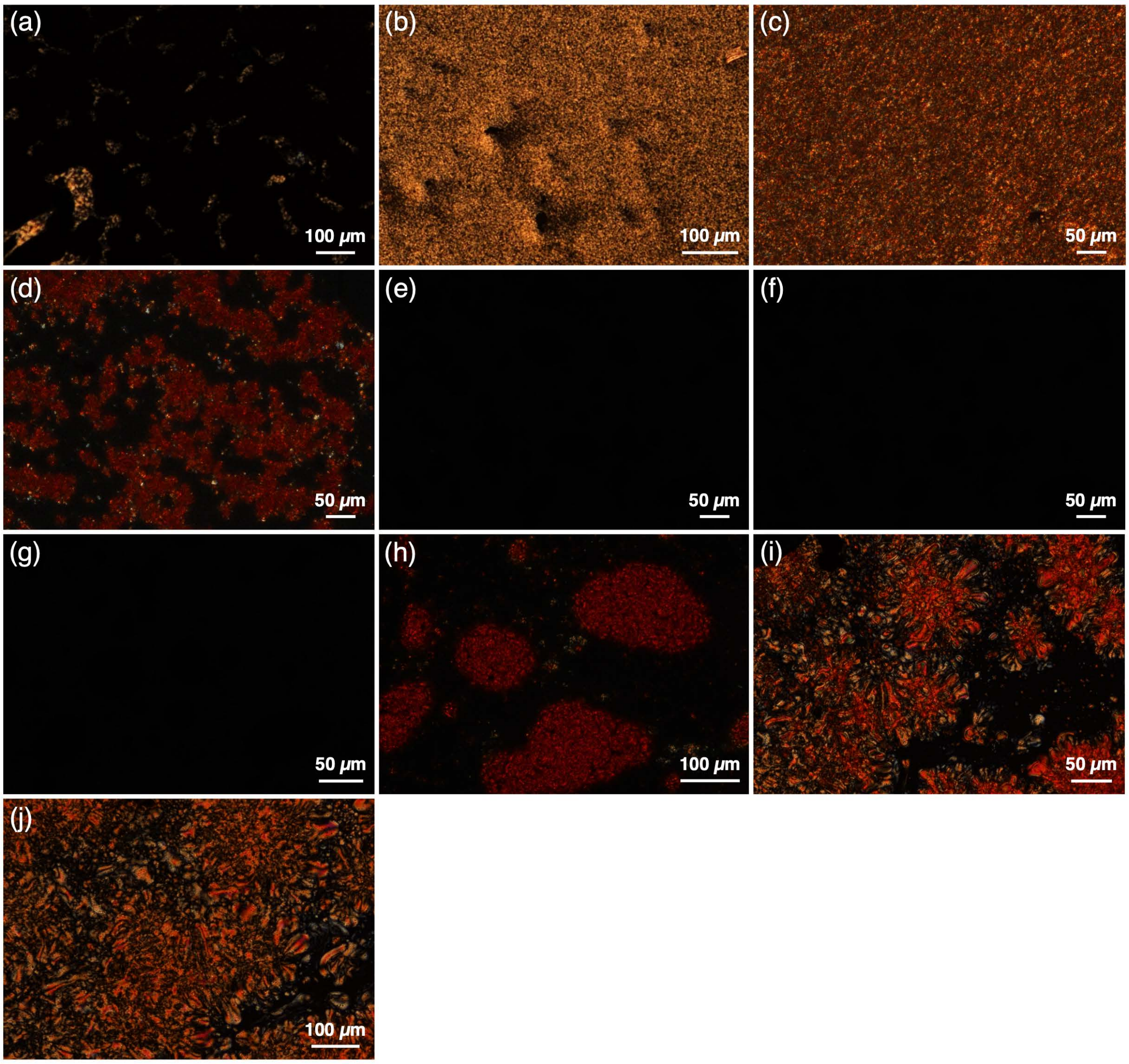

Figure S50 POM textures of **1au**$^{+}$-PCCp$^{-}_{20\%}$ at (a) 20 °C, (b) 30 °C, (c) 50 °C, (d) 70 °C, (e) 80 °C, and (f) 90 °C upon heating and (g) 80 °C, (h) 70 °C, (i) 50 °C, and (j) 40 °C upon cooling.

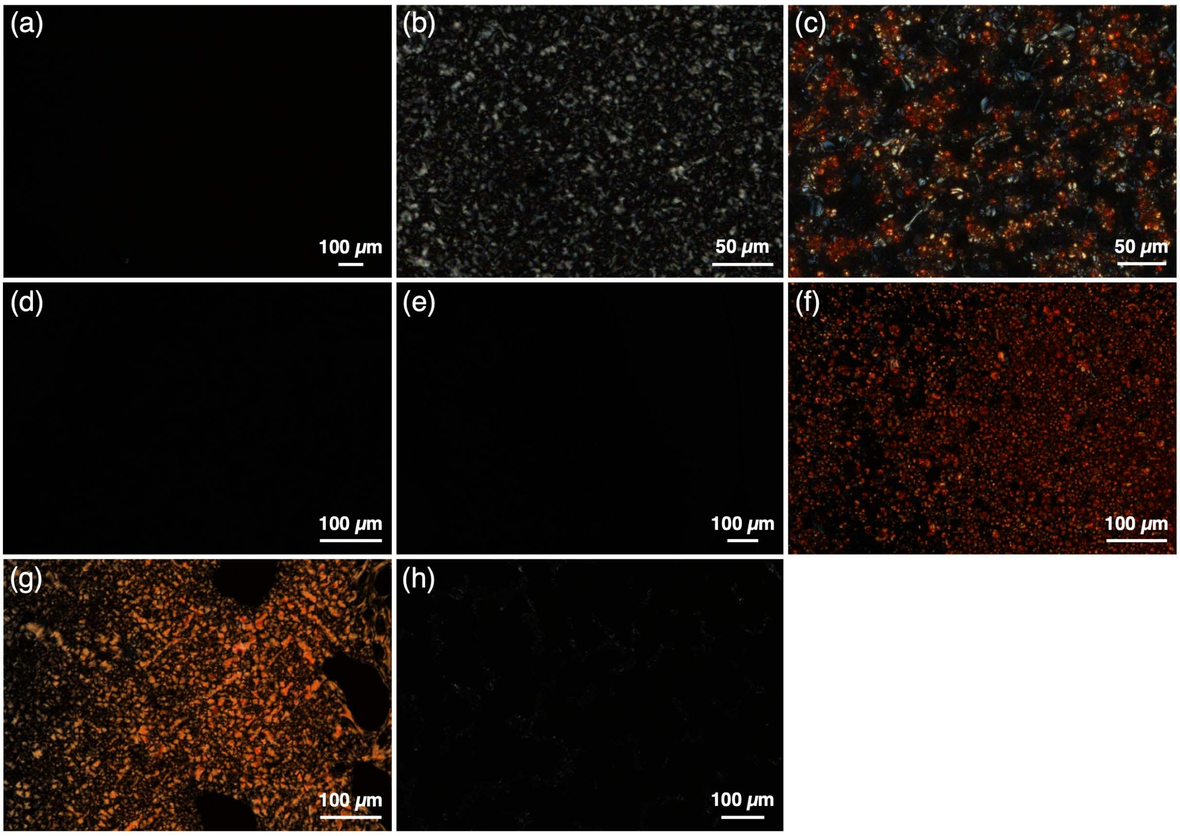

Figure S51 POM textures of **1au**$^{+}$-PCCp$^{-}_{10\%}$ at (a) 20 °C, (b) 40 °C, (c) 70 °C, and (d) 90 °C upon heating and (e) 80 °C, (f) 60 °C, (g) 30 °C, and (h) 20 °C upon cooling.

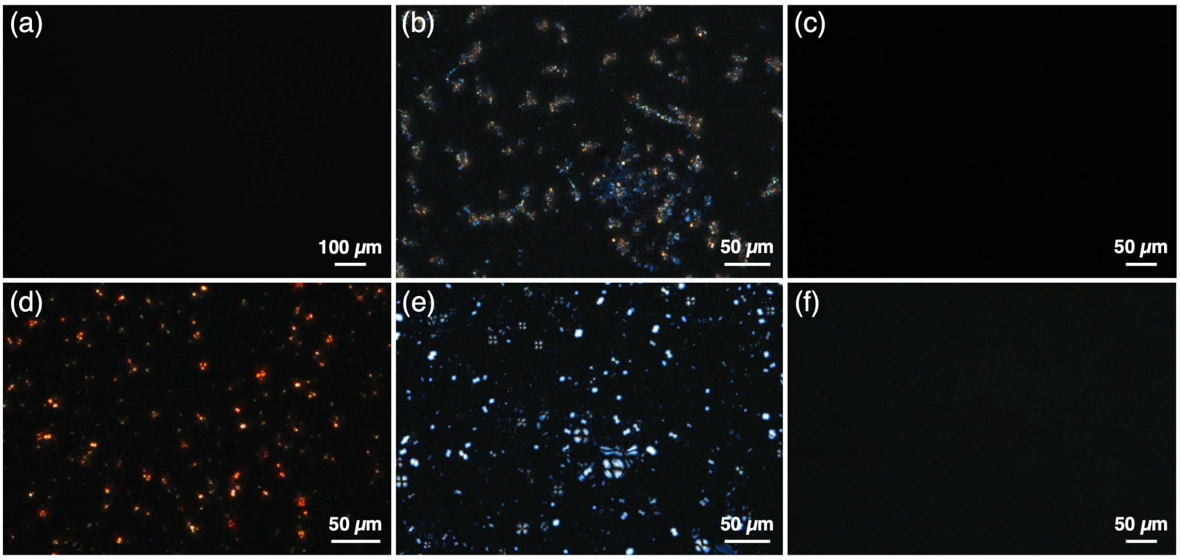

Figure S52 POM textures of **1au**$^{+}$-PCCp$^{-}_{1\%}$ at (a) 20 °C, (b) 60 °C, and (c) 80 °C upon heating and (d) 70 °C, (e) 50 °C, and (f) 20 °C upon cooling.

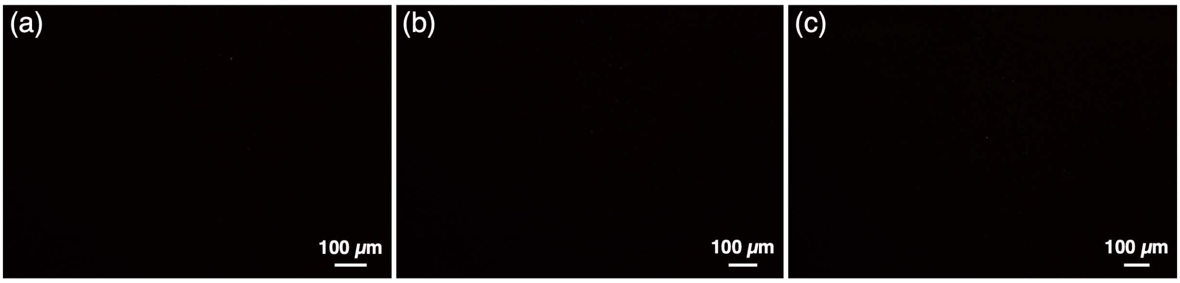

Figure S53 POM textures of **2**$_{70\%}$ at (a) 20 °C and (b) 80 °C upon heating and (c) 20 °C upon cooling.

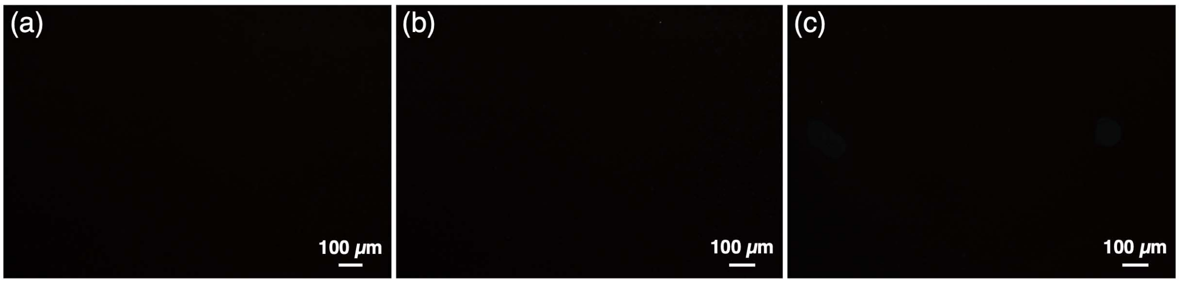

Figure S54 POM textures of **2**$_{50\%}$ at (a) 20 °C and (b) 80 °C upon heating and (c) 20 °C upon cooling.

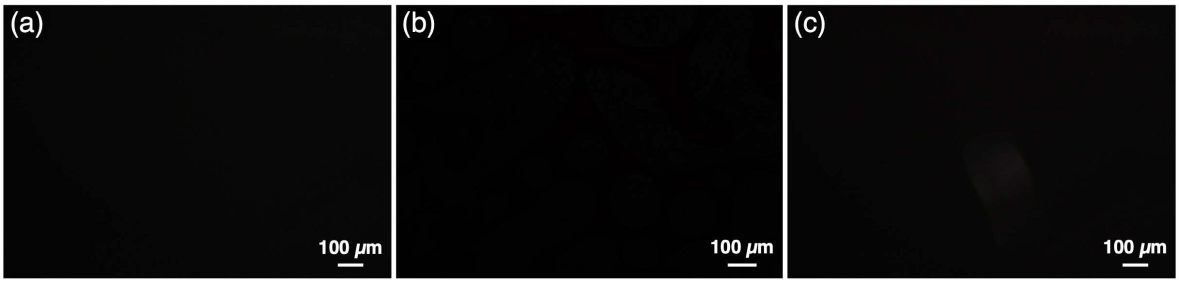

Figure S55 POM textures of **2**$_{20\%}$ at (a) 20 °C and (b) 80 °C upon heating and (c) 20 °C upon cooling.

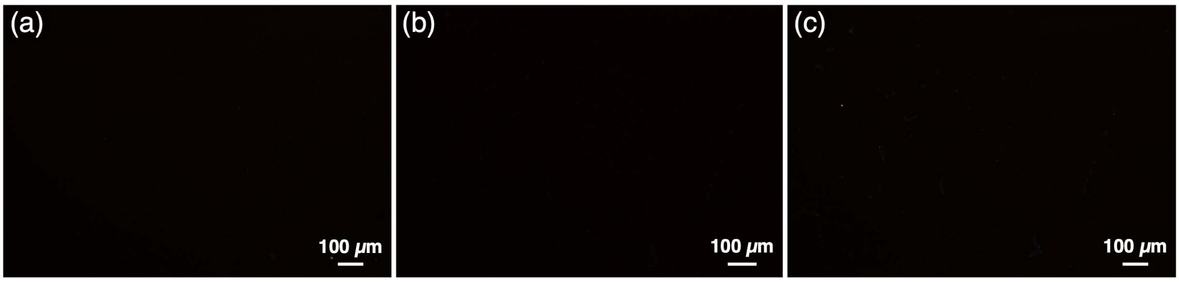

Figure S56 POM textures of **2au**$^{+}$-Cl$^{-}_{70\%}$ at (a) 20 °C and (b) 80 °C upon heating and (c) 20 °C upon cooling.

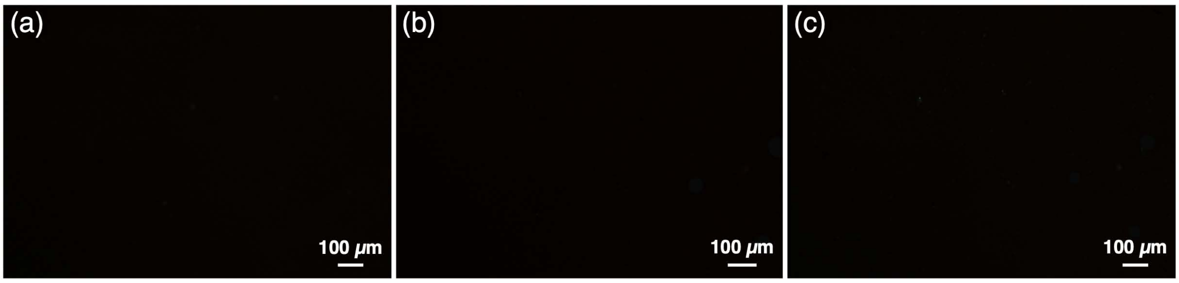

Figure S57 POM textures of **2au**$^{+}$-Cl$^{-}_{50\%}$ at (a) 20 °C and (b) 80 °C upon heating and (c) 20 °C upon cooling.

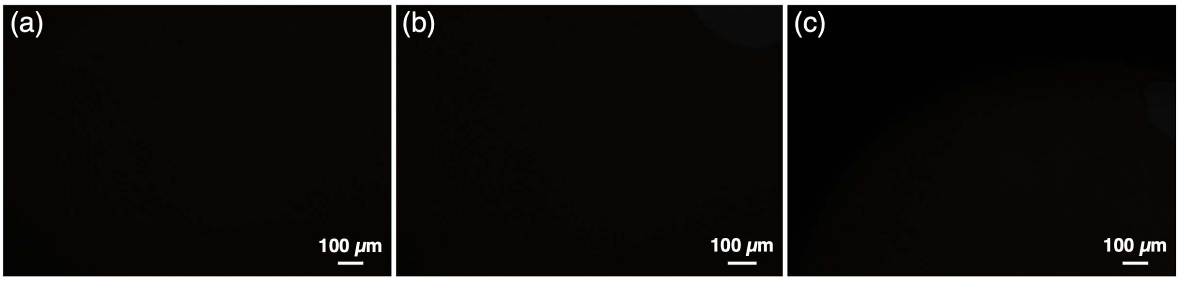

Figure S58 POM textures of **2au**$^{+}$-Cl^{-}$_{20\%}$ at (a) 20 °C and (b) 80 °C upon heating and (c) 20 °C upon cooling.

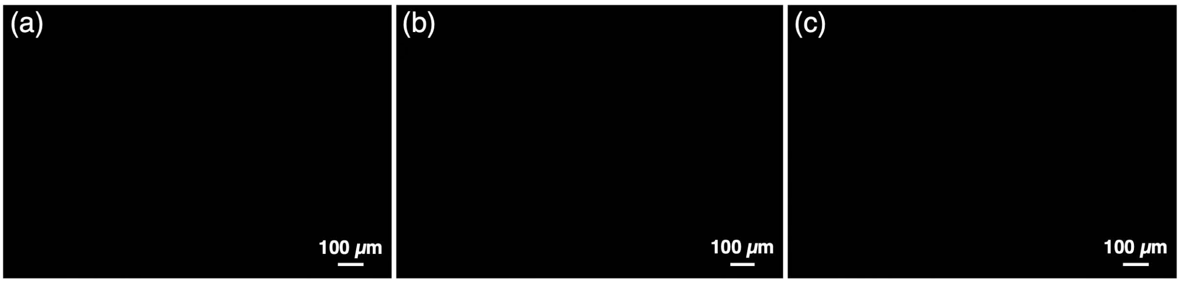

Figure S59 POM textures of **2au**$^{+}$-PCCp^{-}$_{80\%}$ at (a) 20 °C and (b) 80 °C upon heating and (c) 20 °C upon cooling.

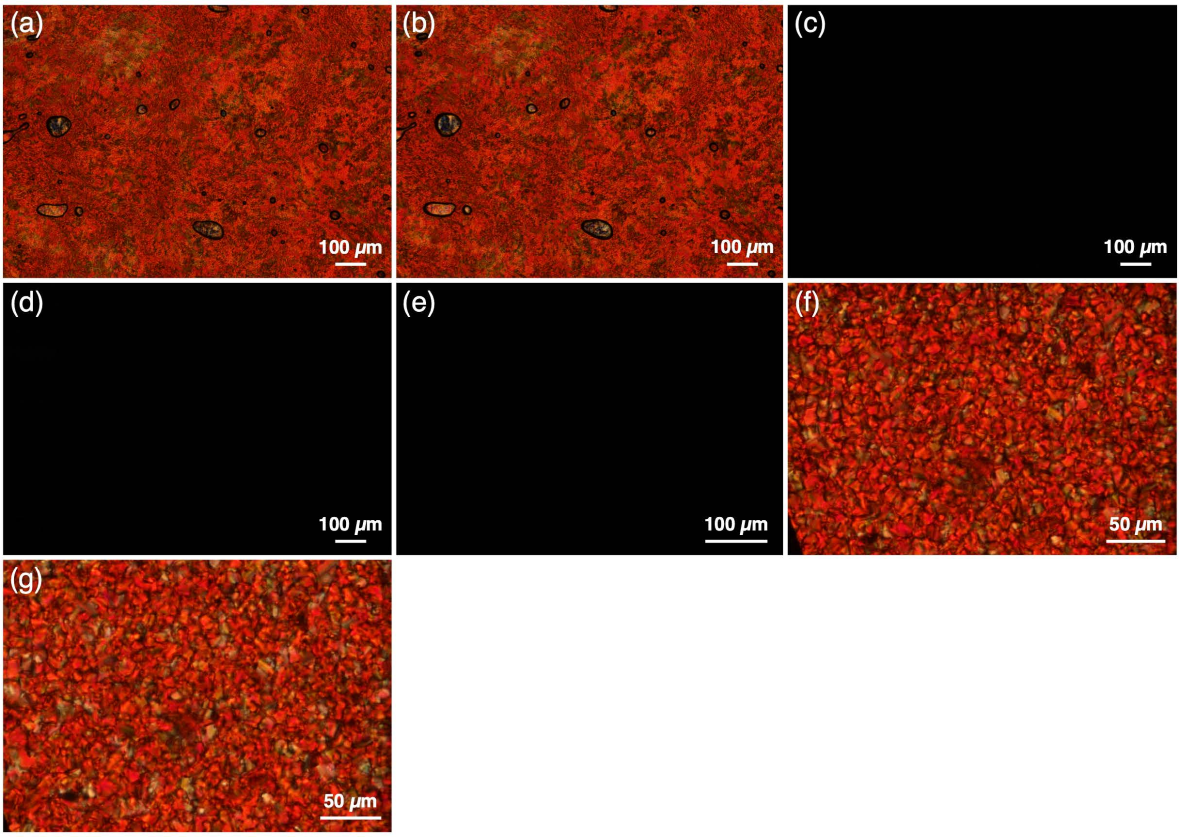

Figure S60 POM textures of **2au**$^{+}$-PCCp^{-}$_{70\%}$ at (a) 20 °C, (b) 50 °C, (c) 70 °C, and (d) 80 °C upon heating and (e) 65 °C , (f) 50 °C, and (g) 20 °C upon cooling.

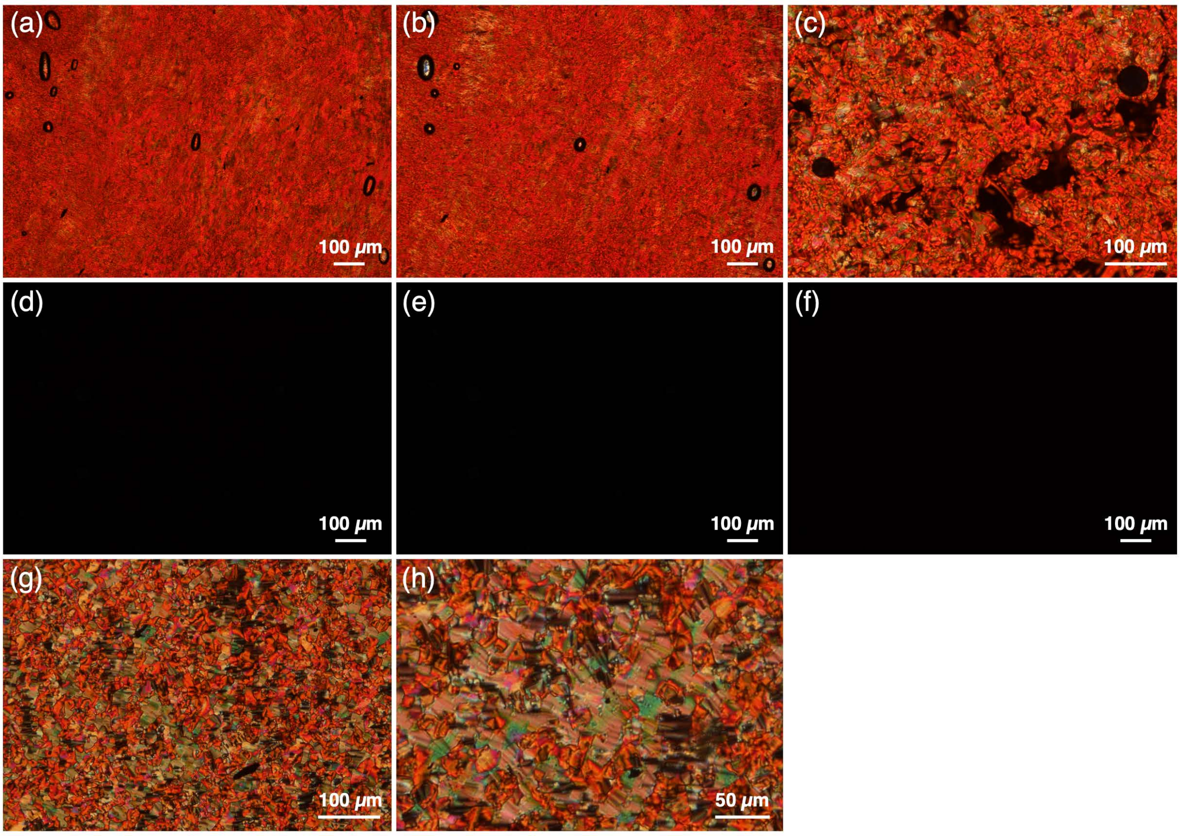

Figure S61 POM textures of **2au**$^{+}$-PCCp$^{-}_{60\%}$ at (a) 20 °C, (b) 60 °C, (c) 65 °C, (d) 70 °C and (e) 80 °C upon heating and (f) 70 °C, (g) 60 °C, and (h) 20 °C upon cooling.

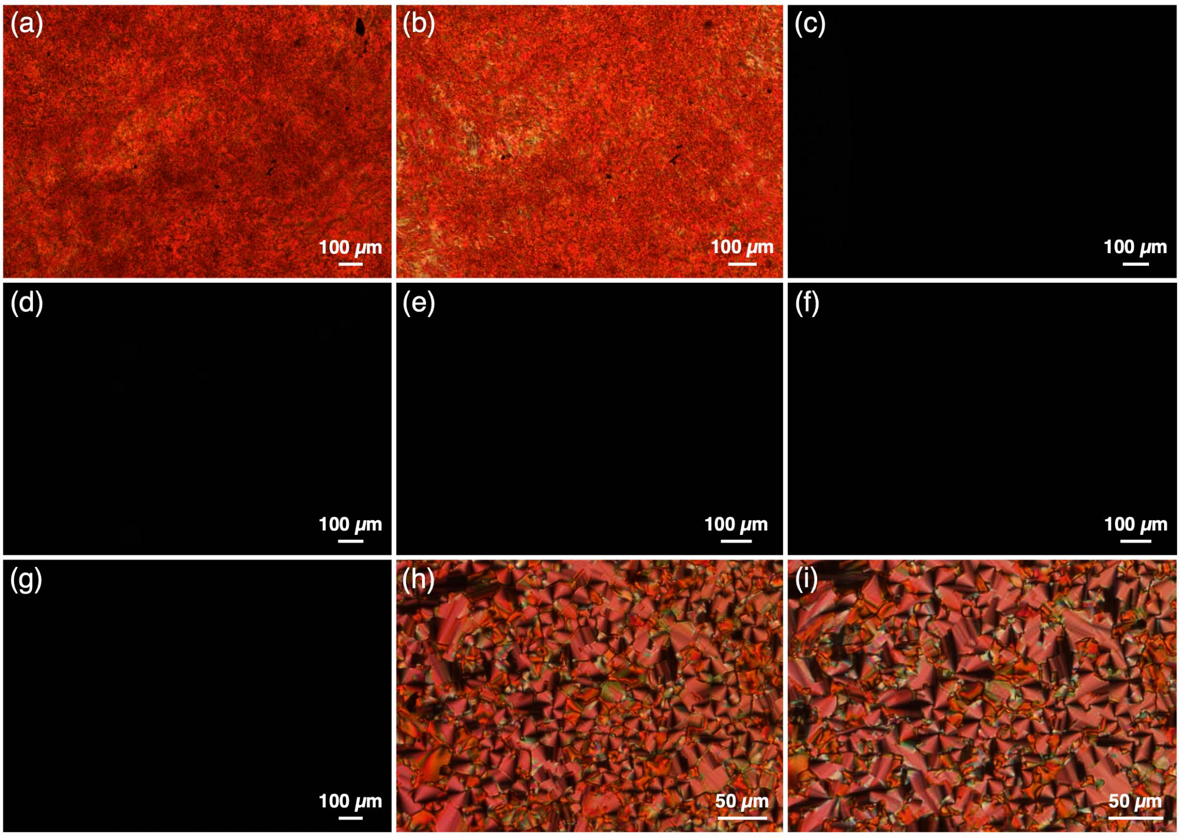

Figure S62 POM textures of **2au**$^{+}$-PCCp$^{-}_{50\%}$ at (a) 20 °C, (b) 60 °C, (c) 70 °C, (d) 80 °C, and (e) 90 °C upon heating and (f) 80 °C, (g) 70 °C, (h) 60 °C, and (i) 20 °C upon cooling.

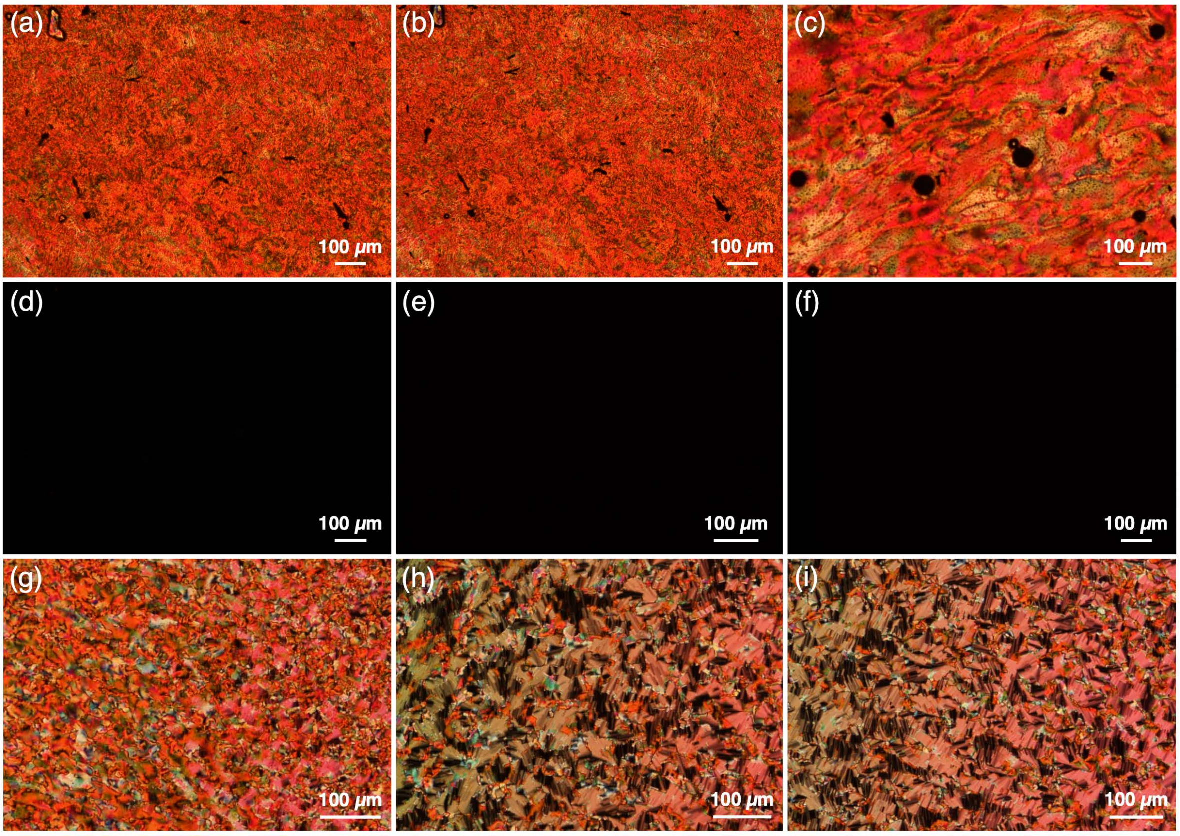

Figure S63 POM textures of **2au**$^{+}$-PCCp$^{-}_{40\%}$ at (a) 20 °C, (b) 50 °C, (c) 60 °C, (d) 70 °C, and (e) 80 °C upon heating and (f) 70 °C, (g) 60 °C, (h) 50 °C, and (i) 20 °C upon cooling.

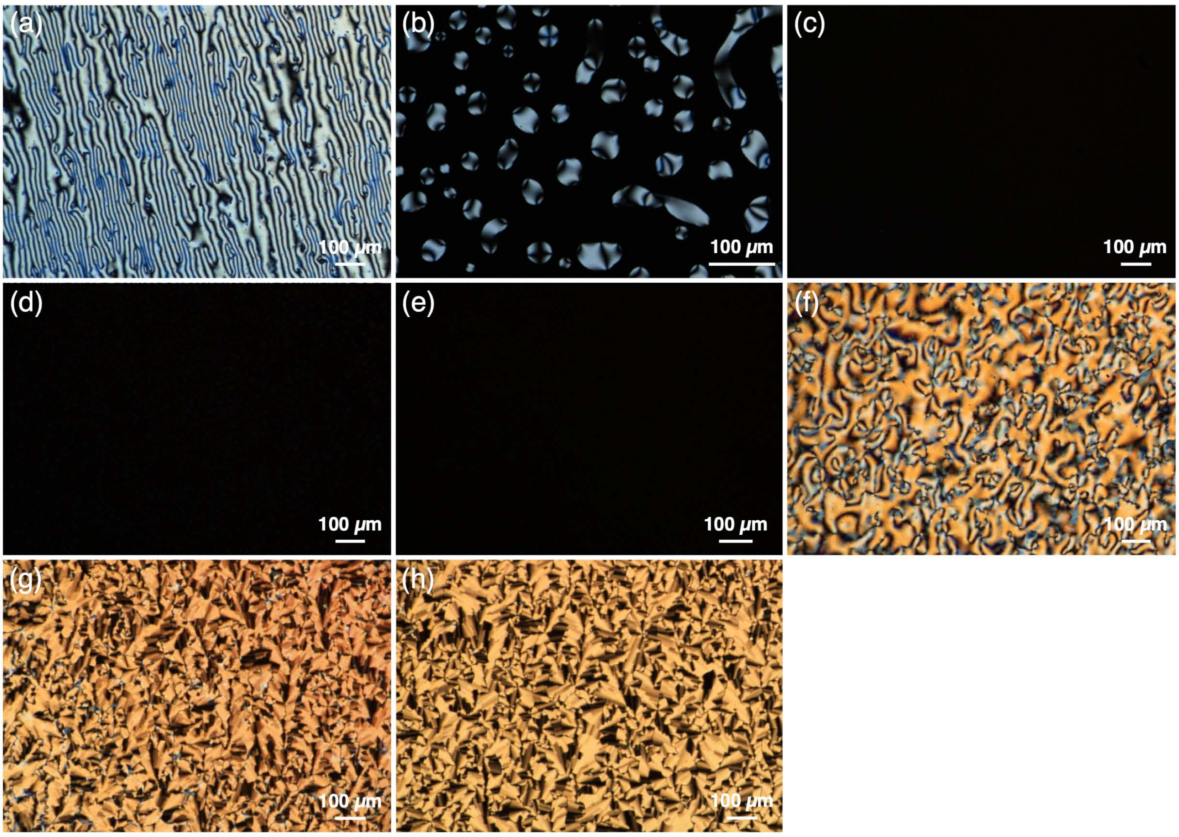

Figure S64 POM textures of **2au**$^{+}$-PCCp$^{-}_{30\%}$ at (a) 20 °C, (b) 50 °C, (c) 60 °C, and (d) 80 °C upon heating and (e) 60 °C, (f) 50 °C, (g) 30 °C, and (h) 20 °C upon cooling.

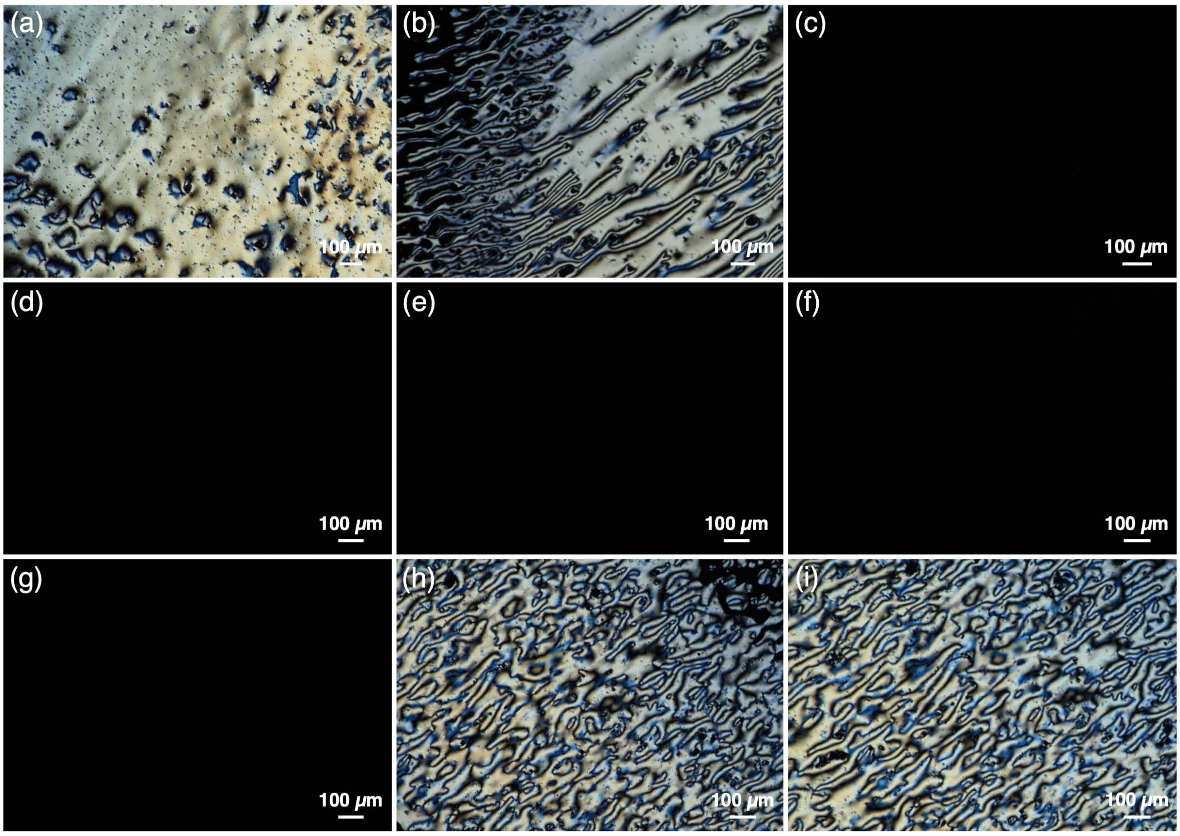

Figure S65 POM textures of **2au**$^+$-PCCp$^-_{20\%}$ at (a) 20 °C, (b) 40 °C, (c) 50 °C, (d) 60 °C, (e) 80 °C, and (f) 90 °C upon heating and (g) 60 °C, (h) 40 °C, and (i) 20 °C upon cooling.

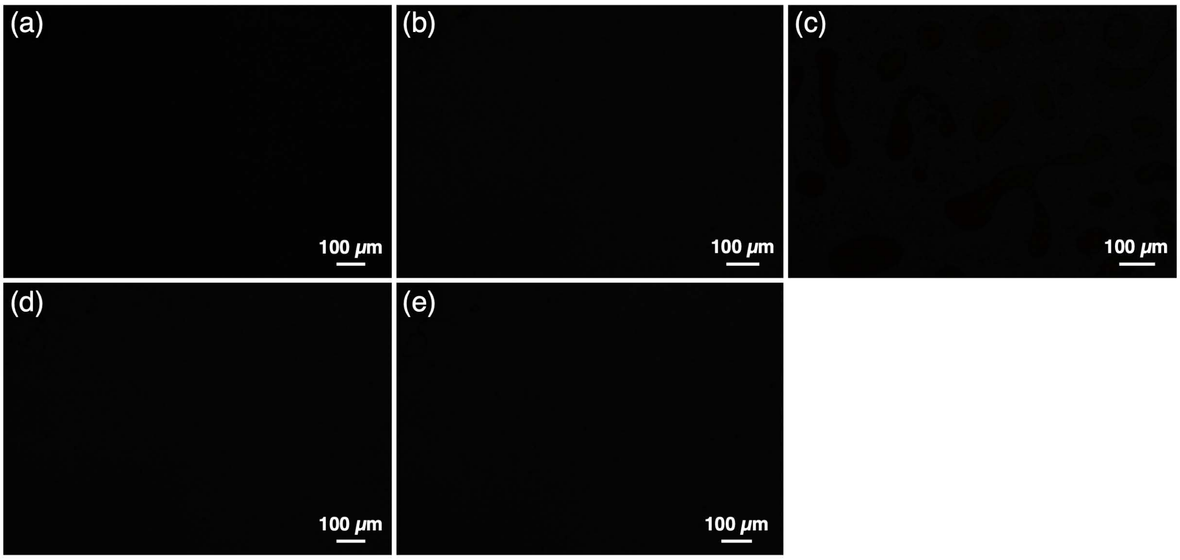

Figure S66 POM textures of **2au**$^+$-PCCp$^-_{10\%}$ at (a) 20 °C, (b) 60 °C, and (c) 80 °C upon heating and (d) 60 °C and (e) 20 °C upon cooling.

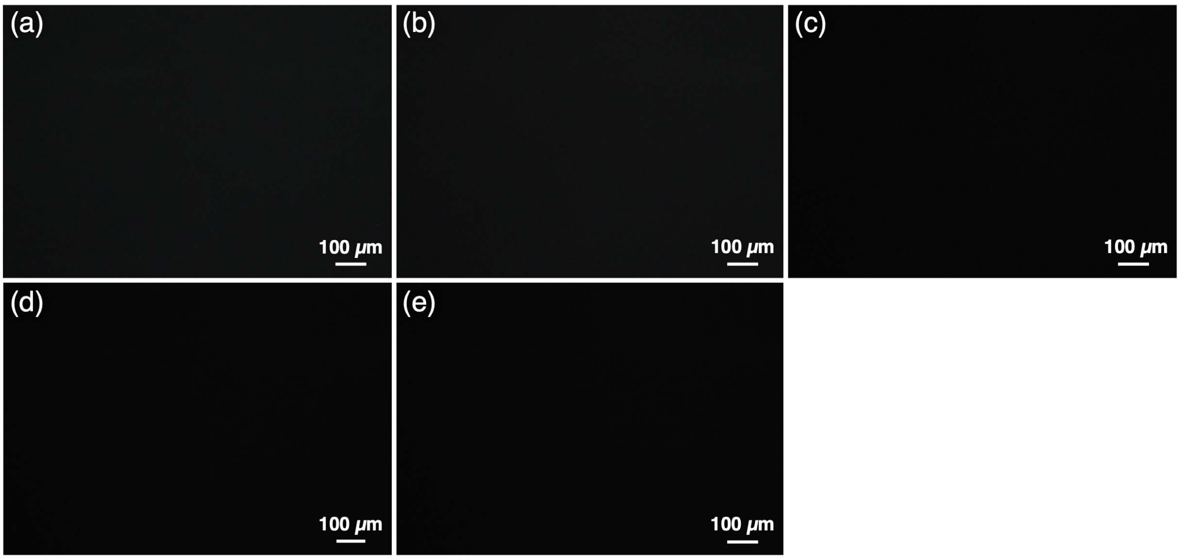

Figure S67 POM textures of **2au**$^{+}$-PCCp$^{-}_{1\%}$ at (a) 20 °C, (b) 70 °C, and (c) 80 °C upon heating and (d) 70 °C and (e) 20 °C upon cooling.

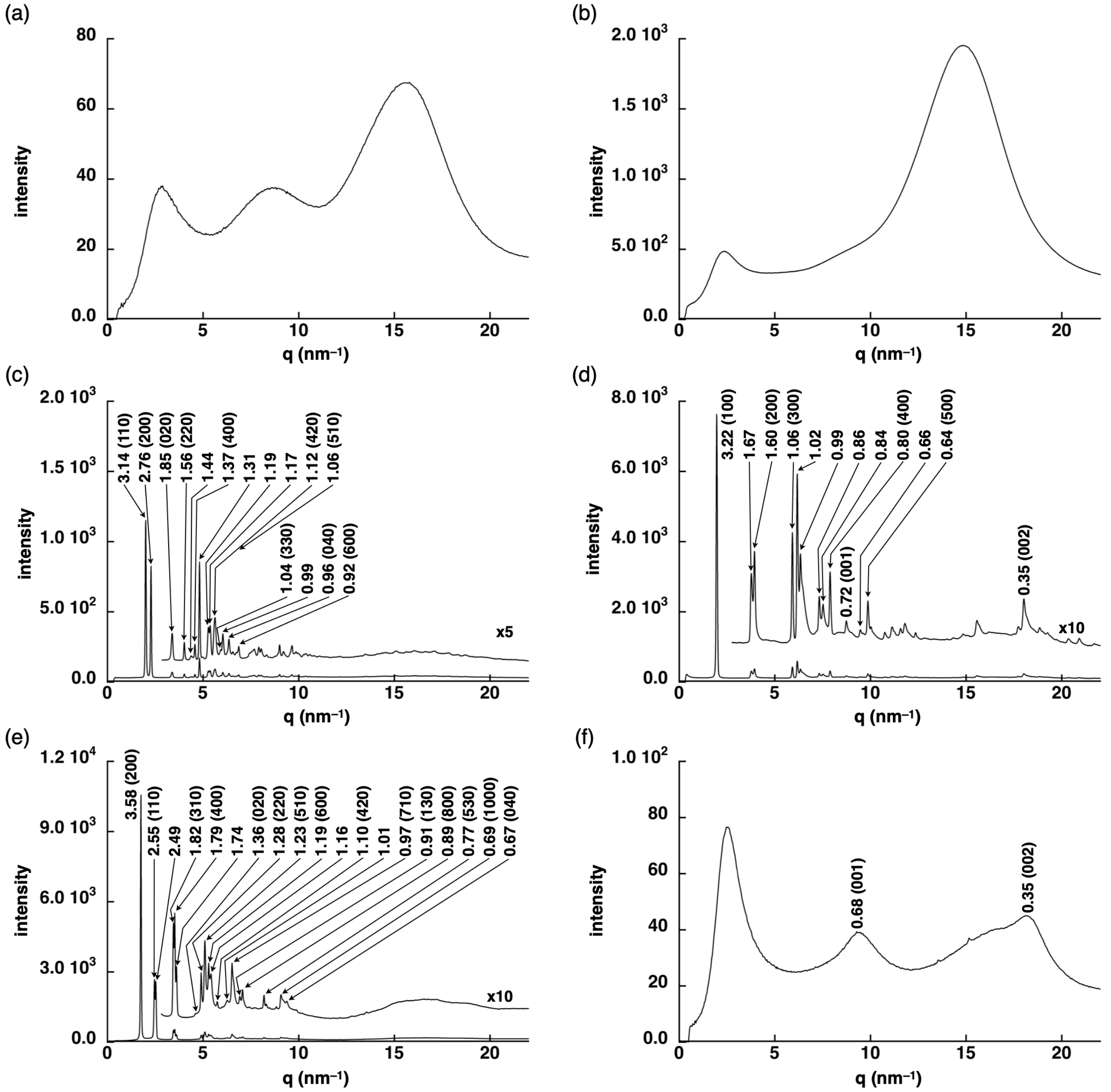

Figure S68 XRD patterns of (a) **1**, (b) **2**, (c) **1au**$^+$-Cl$^-$, (d) **1au**$^+$-PCCp$^-$, (e) **2au**$^+$-Cl$^-$, and (f) **2au**$^+$-PCCp$^-$ at 25 °C. The samples of **1au**$^+$-Cl$^-$, **1au**$^+$-PCCp$^-$, and **2au**$^+$-Cl$^-$ were prepared by precipitation from $CHCl_3$/*n*-hexane, whereas those of **1**, **2**, and **2au**$^+$-PCCp$^-$ were prepared by evaporation of $CHCl_3$ solutions at 80 °C.

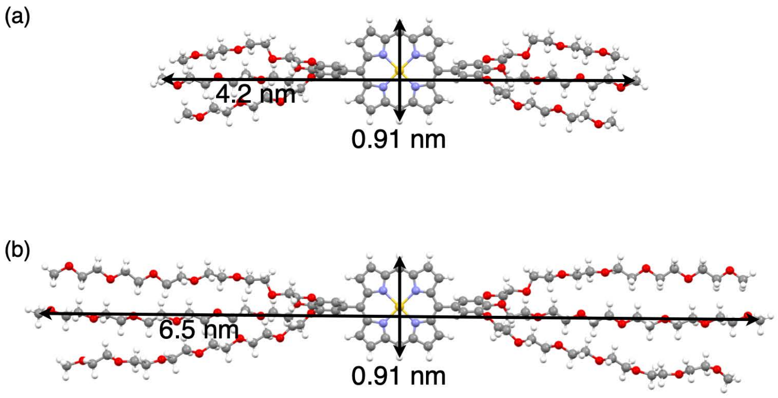

Figure S69 Molecular structures of (a) **1au**$^+$ and (b) **2au**$^+$.

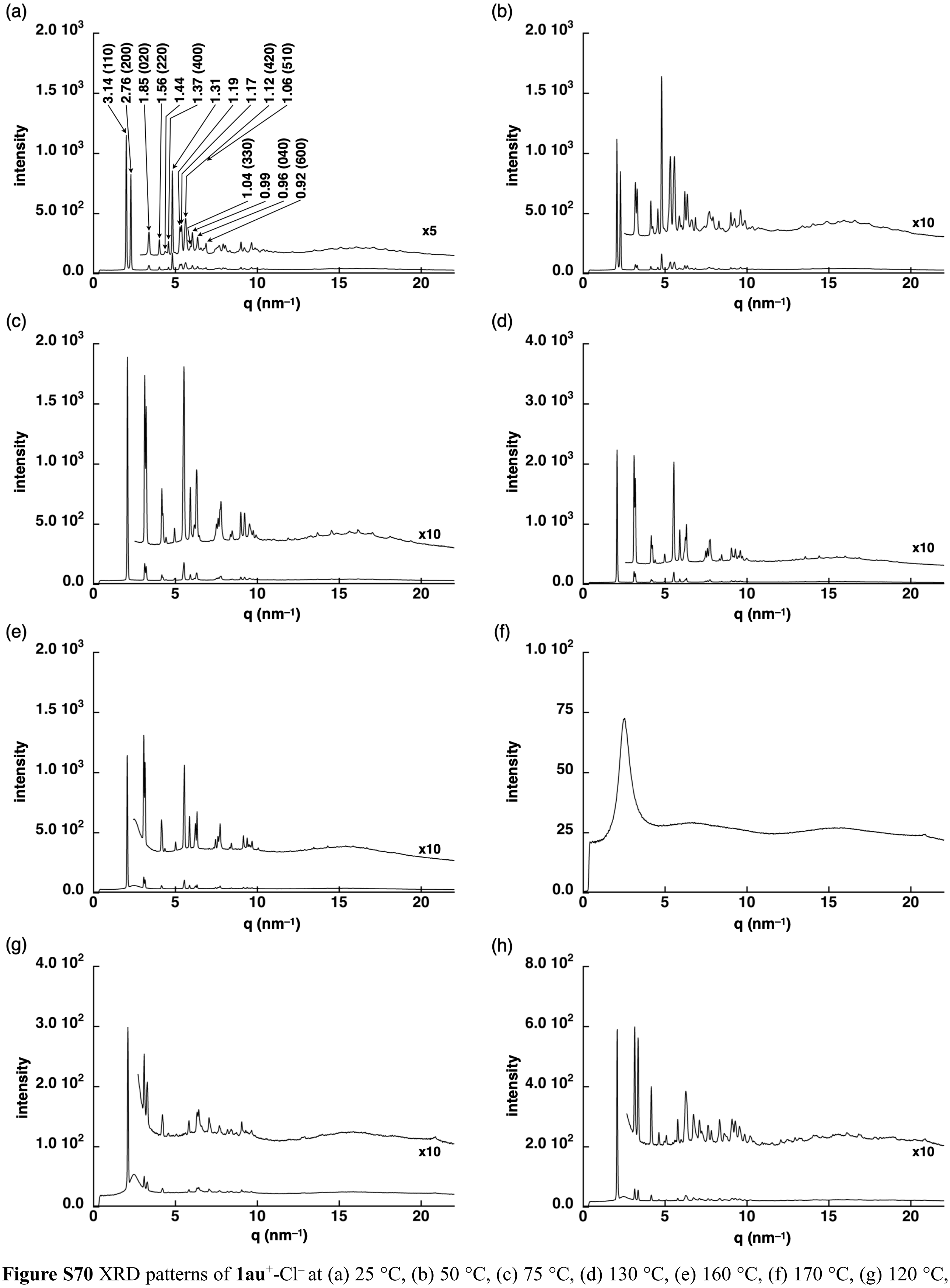

Figure S70 XRD patterns of **1au**$^{+}$-Cl^{-} at (a) 25 °C, (b) 50 °C, (c) 75 °C, (d) 130 °C, (e) 160 °C, (f) 170 °C, (g) 120 °C, (h) 100 °C, (i) 75 °C, (j) 60 °C, (k) 35 °C, (l) 20 °C, (m) 0 °C, (n) 20 °C, (o) 50 °C, (p) 75 °C, (q) 130 °C, and (r) 170 °C upon (a–f) 1st heating, (g–m) 1st cooling, and (n–r) 2nd heating.

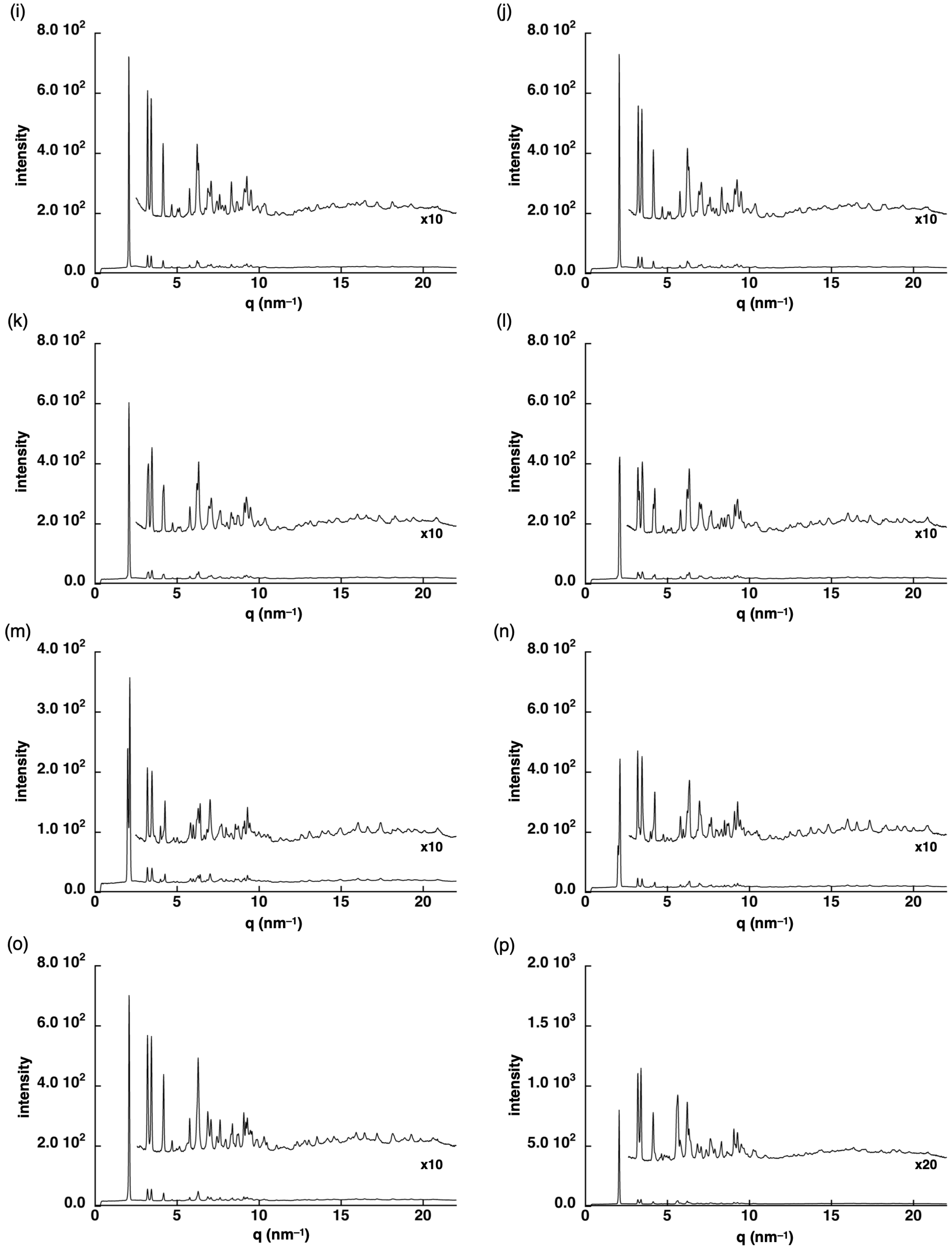

Figure S70 (Continued)

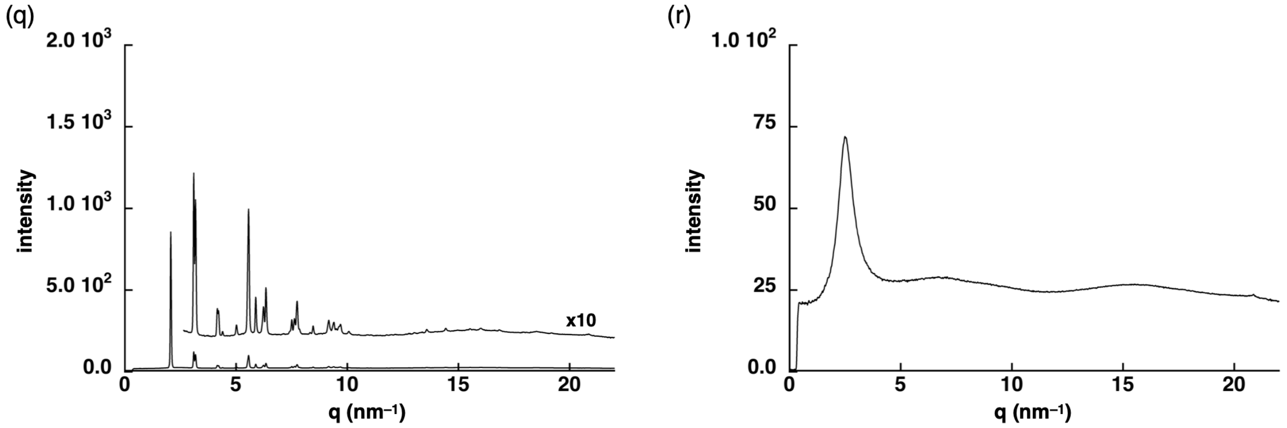

Figure S70 (Continued)

Table S2 Summary of XRD data of **1au**$^{+}$-Cl^{-}. The peaks which can be indexed are represented.

	q (nm^{-1})	*d*-spacing (nm)	ratio	ratio (calc.)	*hkl*
(a) 25 °C (1st heating)<br>Col$_r$<br>*a* = 5.51 nm, *b* = 3.82 nm	2.00	3.14	1.000	1.0000	110
	2.28	2.76	0.878	0.8782	200
	3.39	1.85	0.591	0.6082	020
	4.02	1.56	0.498	0.5000	220
	4.57	1.37	0.438	0.4391	400
	5.63	1.12	0.356	0.3560	420
	5.93	1.06	0.338	0.3375	510
	6.03	1.04	0.332	0.3333	330
	6.55	0.96	0.306	0.3041	040
	6.86	0.92	0.292	0.2927	600

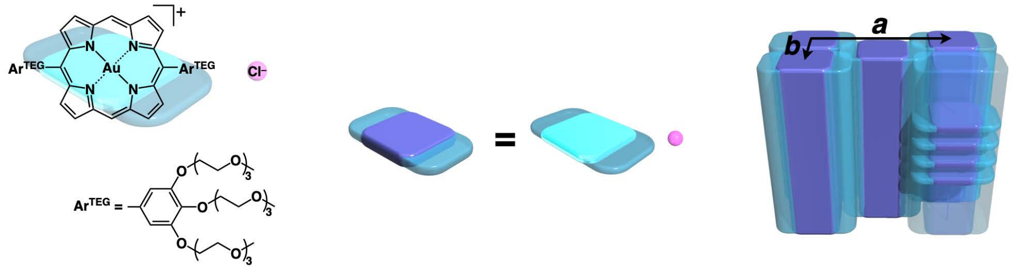

Figure S71 Possible packing model of **1au**$^{+}$-Cl^{-} as a Col$_r$ structure.

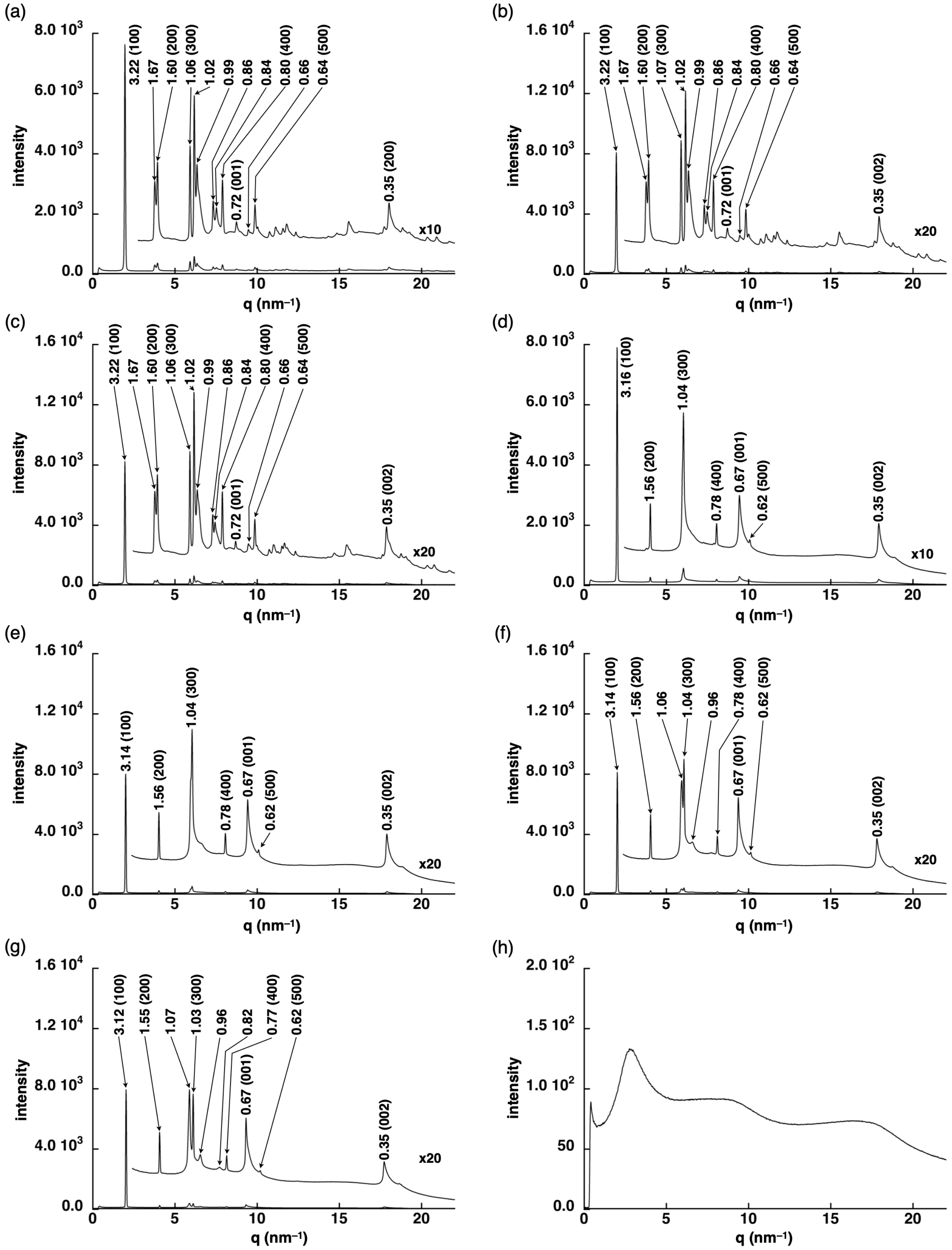

Figure S72 XRD patterns of **1au**$^{+}$-PCCp^{-} at (a) 25 °C, (b) 50 °C, (c) 70 °C, (d) 90 °C, (e) 100 °C, (f) 125 °C, (g) 150 °C, (h) 180 °C, (i) 150 °C, (j) 110 °C, (k) 80 °C, (l) 50 °C, (m) 20 °C, (n) 50 °C, (o) 80 °C, (p) 100 °C, (q) 125 °C, (r) 150 °C, and (s) 180 °C upon (a–h) 1st heating, (i–m) 1st cooling, and (n–s) 2nd heating.

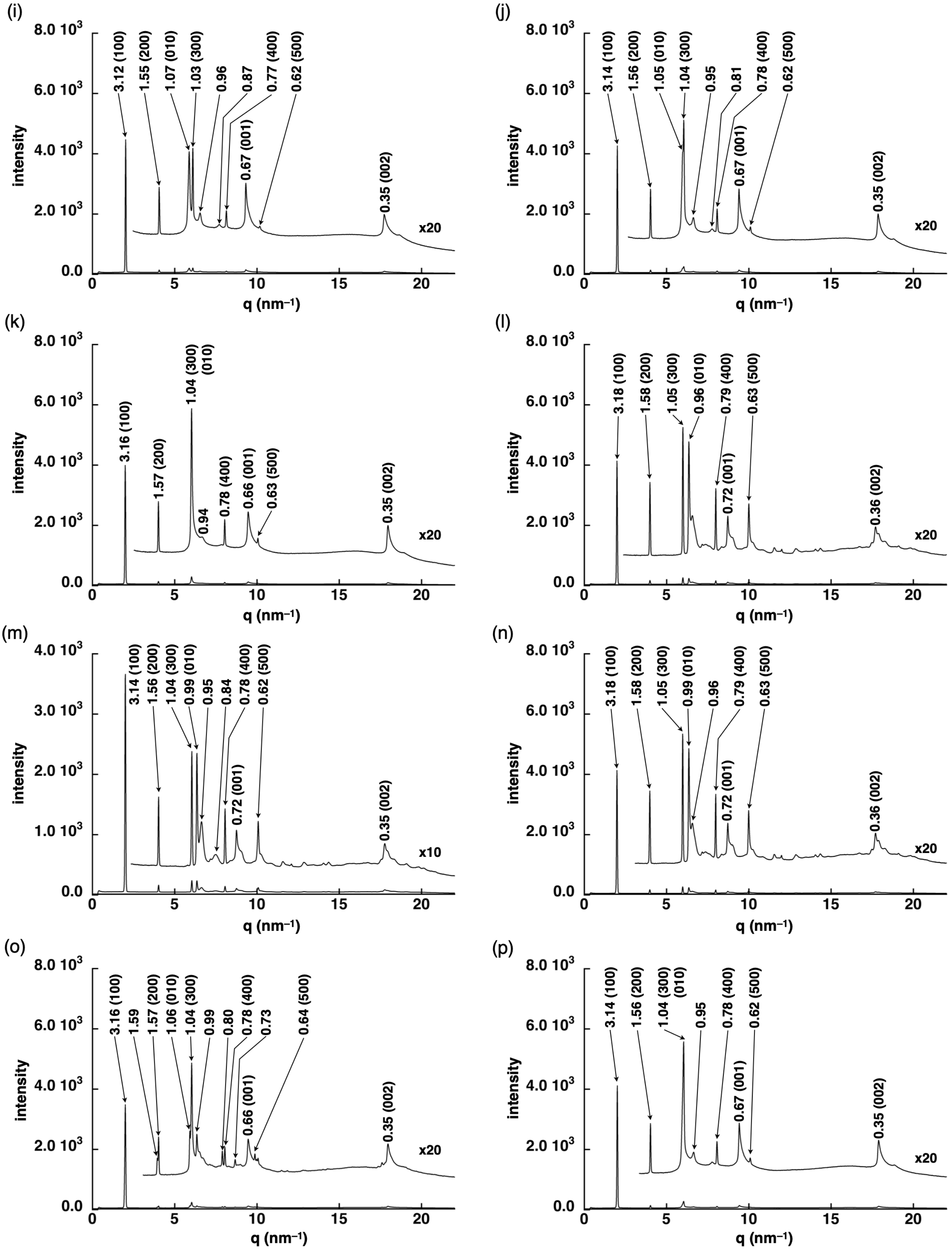

Figure S72 (Continued)

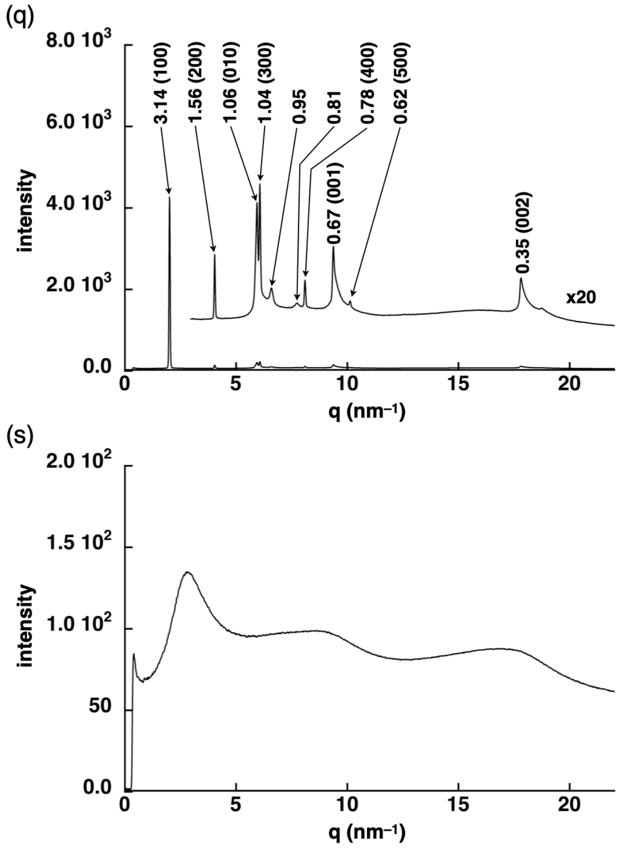

Figure S72 (Continued)

Table S3 Summary of XRD data of **1au**$^+$-PCCp$^-$. The peaks which can be indexed are represented.

	q (nm^{-1})	*d*-spacing (nm)	ratio	ratio (calc.)	*hkl*
	1.95	3.22	1.000	1.0000	100
	3.93	1.60	0.497	0.5000	200
(a) 25 °C (1st heating)	5.91	1.06	0.330	0.3333	300
Lam$_{col}$	7.88	0.80	0.248	0.2500	400
a = 3.22 nm, c = 0.72 nm	8.74	0.72	–	–	001
	9.86	0.64	0.198	0.2000	500
	18.0	0.35	–	–	002
	1.95	3.22	1.000	1.0000	100
	3.92	1.60	0.498	0.5000	200
(b) 50 °C (1st heating)	5.89	1.07	0.331	0.3333	300
Lam$_{col}$	7.86	0.80	0.248	0.2500	400
a = 3.22 nm, c = 0.72 nm	8.70	0.72	–	–	001
	9.83	0.64	0.199	0.2000	500
	17.9	0.35	–	–	002
	1.95	3.22	1.000	1.0000	100
	3.93	1.60	0.497	0.5000	200
(c) 70 °C (1st heating)	5.90	1.06	0.331	0.3333	300
Lam$_{col}$	7.88	0.80	0.248	0.2500	400
a = 3.22 nm, c = 0.72 nm	8.69	0.72	–	–	001
	9.85	0.64	0.198	0.2000	500
	17.9	0.35	–	–	002
	1.99	3.16	1.000	1.0000	100
	4.02	1.56	0.495	0.5000	200
(d) 90 °C (1st heating)	6.03	1.04	0.330	0.3333	300
Lam$_{col}$	8.06	0.78	0.247	0.2500	400
a = 3.16 nm, c = 0.67 nm	9.44	0.67	–	–	001
	10.1	0.62	0.198	0.2000	500
	17.9	0.35	–	–	002
	2.00	3.14	1.000	1.0000	100
	4.02	1.56	0.498	0.5000	200
(e) 100 °C (1st heating)	6.04	1.04	0.332	0.3333	300
Lam$_{col}$	8.06	0.78	0.249	0.2500	400
a = 3.14 nm, c = 0.67 nm	9.42	0.67	–	–	001
	10.1	0.62	0.199	0.2000	500
	17.9	0.35	–	–	002
	2.00	3.14	1.000	1.0000	100
	4.03	1.56	0.497	0.5000	200
(f) 125 °C (1st heating)	6.06	1.04	0.330	0.3333	300
Lam$_{col}$	8.10	0.78	0.247	0.2500	400
a = 3.14 nm, c = 0.67 nm	9.37	0.67	–	–	001
	10.1	0.62	0.198	0.2000	500
	17.8	0.35	–	–	002
	2.02	3.12	1.000	1.0000	100
	4.06	1.55	0.497	0.5000	200
(g) 150 °C (1st heating)	6.10	1.03	0.330	0.3333	300
Lam$_{col}$	8.15	0.77	0.247	0.2500	400
a = 3.12 nm, c = 0.67 nm	9.32	0.67	–	–	001
	10.2	0.62	0.198	0.2000	500
	17.7	0.35	–	–	002

Table S3 (Continued)

	q (nm^{-1})	*d*-spacing (nm)	ratio	ratio (calc.)	*hkl*
(i) 150 °C (1st cooling)<br>Lam_{col}-H<br>*a* = 3.12 nm, *b* = 1.07 nm, *c* = 0.67 nm	2.02	3.12	1.000	1.0000	100
	4.06	1.55	0.497	0.5000	200
	5.88	1.07	–	–	010
	6.10	1.03	0.330	0.3333	300
	8.13	0.77	0.248	0.2500	400
	9.32	0.67	–	–	001
	10.2	0.62	0.198	0.2000	500
	17.7	0.35	–	–	002
(j) 110 °C (1st cooling)<br>Lam_{col}-H<br>*a* = 3.14 nm, *b* = 1.05 nm, *c* = 0.67 nm	2.00	3.14	1.000	1.0000	100
	4.02	1.56	0.498	0.5000	200
	5.99	1.05	–	–	010
	6.04	1.04	0.332	0.3333	300
	8.07	0.78	0.248	0.2500	400
	9.39	0.67	–	–	001
	10.1	0.62	0.198	0.2000	500
	17.8	0.35	–	–	002
(k) 80 °C (1st cooling)<br>Lam_{col}-H<br>*a* = 3.16 nm, *b* = 1.04 nm, *c* = 0.66 nm	1.99	3.16	1.000	1.0000	100
	4.01	1.57	0.497	0.5000	200
	6.03	1.04	0.330	0.3333	300, 010
	8.03	0.78	0.248	0.2500	400
	9.47	0.66	–	–	001
	10.0	0.63	0.198	0.2000	500
	18.0	0.35	–	–	002
(l) 50 °C (1st cooling)<br>Lam_{col}-L<br>*a* = 3.18 nm, *b* = 0.96 nm, *c* = 0.72 nm	1.98	3.18	1.000	1.0000	100
	3.98	1.58	0.497	0.5000	200
	5.99	1.05	0.330	0.3333	300
	6.56	0.96	–	–	010
	7.98	0.79	0.248	0.2500	400
	8.72	0.72	–	–	001
	10.0	0.63	0.198	0.2000	500
	17.7	0.36	–	–	002
(m) 20 °C (1st cooling)<br>Lam_{col}-L<br>*a* = 3.14 nm, *b* = 0.99 nm, *c* = 0.72 nm	2.00	3.14	1.000	1.0000	100
	4.02	1.56	0.498	0.5000	200
	6.04	1.04	0.332	0.3333	300
	6.35	0.99	–	–	010
	8.06	0.78	0.249	0.2500	400
	8.75	0.72	–	–	001
	10.1	0.62	0.199	0.2000	500
	17.8	0.35	–	–	002
(n) 50 °C (2nd heating)<br>Lam_{col}-L<br>*a* = 3.18 nm, *b* = 0.99 nm, *c* = 0.72 nm	1.98	3.18	1.000	1.0000	100
	3.98	1.58	0.497	0.5000	200
	5.98	1.05	0.331	0.3333	300
	6.35	0.99	–	–	010
	7.98	0.79	0.248	0.2500	400
	8.72	0.72	–	–	001
	9.98	0.63	0.198	0.2000	500
	17.7	0.36	–	–	002
(o) 80 °C (2nd heating)<br>Lam_{col}-H<br>*a* = 3.16 nm, *b* = 1.06 nm, *c* = 0.66 nm	1.99	3.16	1.000	1.0000	100
	4.01	1.57	0.497	0.5000	200
	5.91	1.06	–	–	010
	6.03	1.04	0.330	0.3333	300
	8.05	0.78	0.247	0.2500	400
	9.46	0.66	–	–	001
	9.86	0.64	0.202	0.2000	500
	18.0	0.35	–	–	002

Table S3 (Continued)

	q (nm^{-1})	*d*-spacing (nm)	ratio	ratio (calc.)	*hkl*
(p) 100 °C (2nd heating) Lam_{col}-H *a* = 3.14 nm, *b* = 1.04 nm, *c* = 0.67 nm	2.00	3.14	1.000	1.0000	100
	4.02	1.56	0.498	0.5000	200
	6.04	1.04	0.332	0.3333	300, 010
	8.06	0.78	0.249	0.2500	400
	9.42	0.67	–	–	001
	10.1	0.62	0.199	0.2000	500
	17.9	0.35	–	–	002
(q) 125 °C (2nd heating) Lam_{col}-H *a* = 3.14 nm, *b* = 1.06 nm, *c* = 0.67 nm	2.00	3.14	1.000	1.0000	100
	4.03	1.56	0.497	0.5000	200
	5.94	1.06	–	–	010
	6.06	1.04	0.330	0.3333	300
	8.10	0.78	0.247	0.2500	400
	9.37	0.67	–	–	001
	10.1	0.62	0.198	0.2000	500
	17.8	0.35	–	–	002
(r) 150 °C (2nd heating) Lam_{col}-H *a* = 3.12 nm, *b* = 1.07 nm, *c* = 0.67 nm	2.02	3.12	1.000	1.0000	100
	4.06	1.55	0.497	0.5000	200
	5.88	1.07	–	–	010
	6.10	1.03	0.330	0.3333	300
	8.13	0.77	0.248	0.2500	400
	9.32	0.67	–	–	001
	10.2	0.62	0.198	0.2000	500
	17.7	0.35	–	–	002

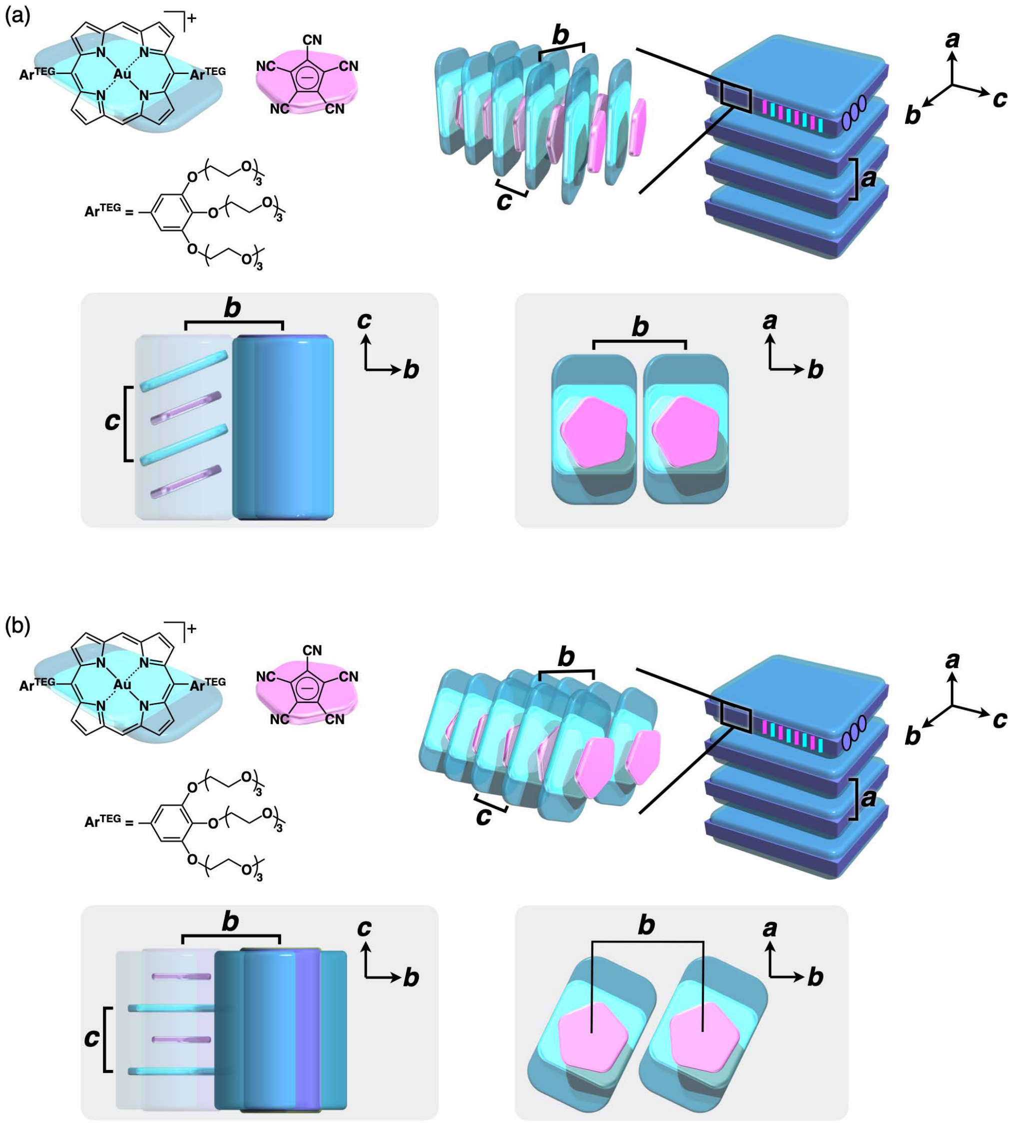

Figure S73 Possible packing models of **1au**$^+$-PCCp$^-$ as (a) a Lam$_{col}$-L structure (20–50 °C) and (b) a Lam$_{col}$-H structure (80–150 °C).

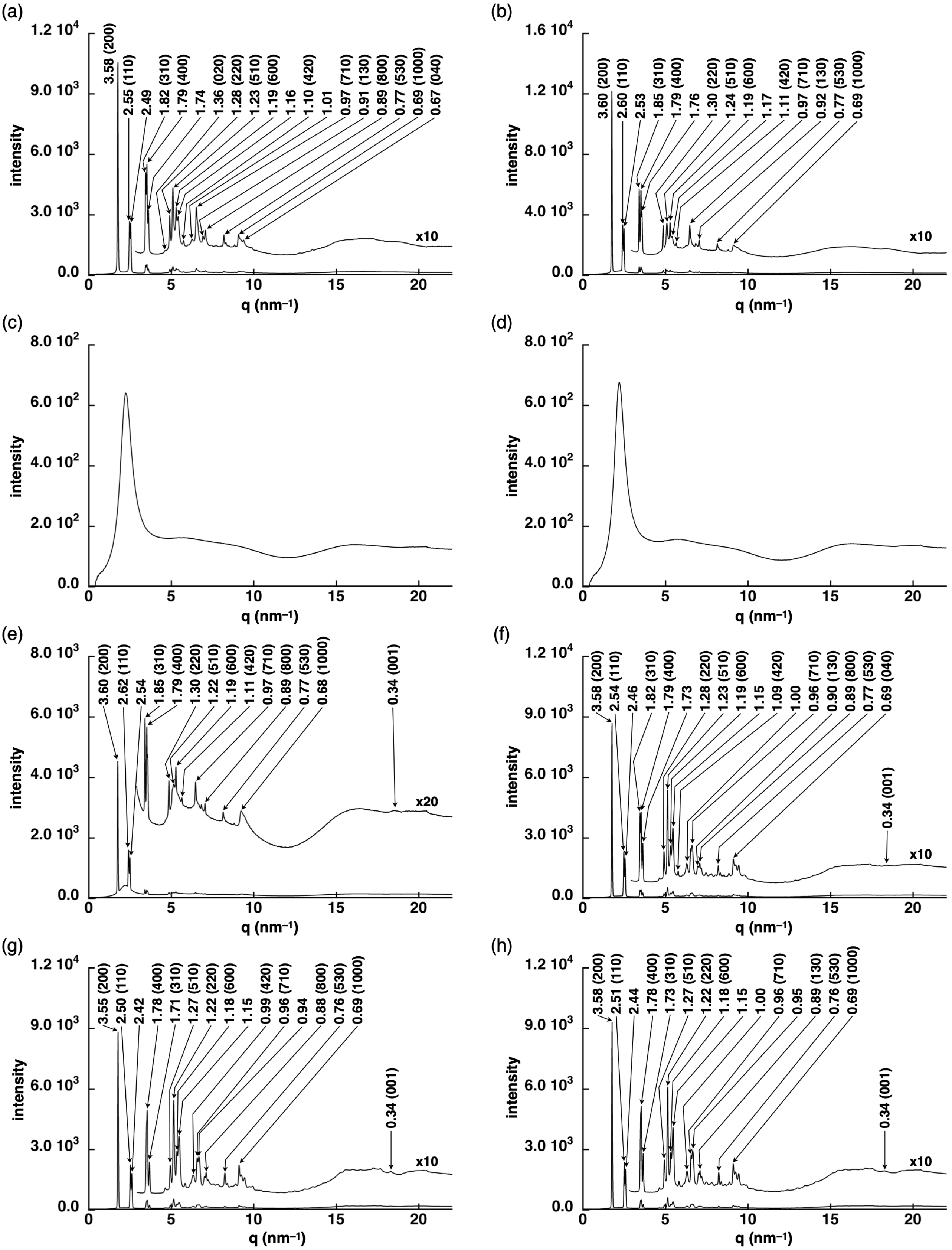

Figure S74 XRD patterns of **2au**$^+$-Cl$^-$ at (a) 25 °C, (b) 80 °C, (c) 120 °C, (d) 90 °C, (e) 80 °C, (f) 20 °C, (g) –20 °C, (h) 5 °C, (i) 90 °C, and (j) 120 °C upon (a–c) 1st heating, (d–g) 1st cooling, and (h–j) 2nd heating.

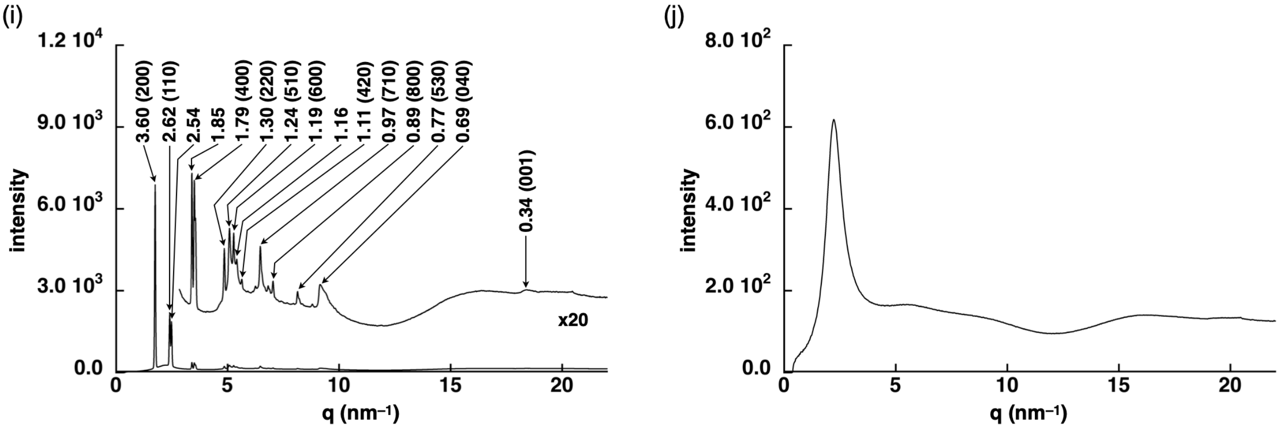

Figure S74 (Continued)

Table S4 Summary of XRD data of **2au**$^{+}$-Cl^{-}. The peaks which can be indexed are represented.

	q (nm^{-1})	*d*-spacing (nm)	ratio	ratio (calc.)	*hkl*
	1.76	3.58	1.000	1.0000	200
	2.46	2.55	0.713	0.7130	110
	3.45	1.82	0.508	0.5021	310
	3.52	1.79	0.500	0.5000	400
	4.61	1.36	0.381	0.3816	020
	4.90	1.28	0.358	0.3565	220
(a) 25 °C (1st heating)	5.10	1.23	0.345	0.3543	510
Col$_r$	5.29	1.19	0.332	0.3333	600
a = 7.16 nm, *b* = 2.73 nm	5.74	1.10	0.306	0.3033	420
	6.51	0.97	0.270	0.2676	710
	6.91	0.91	0.254	0.2524	130
	7.06	0.89	0.249	0.2500	800
	8.18	0.77	0.215	0.2147	530
	9.07	0.69	0.194	0.2000	1000
	9.37	0.67	0.187	0.1908	040
	1.74	3.60	1.000	1.0000	200
	2.41	2.60	0.723	0.7225	110
	3.40	1.85	0.512	0.5054	310
	3.50	1.79	0.498	0.5000	400
(b) 80 °C (1st heating)	4.85	1.30	0.360	0.3613	220
Col$_r$	5.07	1.24	0.344	0.3554	510
a = 7.21 nm, *b* = 2.79 nm	5.27	1.19	0.331	0.3333	600
	5.65	1.11	0.309	0.3063	420
	6.47	0.97	0.269	0.2681	710
	6.83	0.92	0.255	0.2562	130
	8.14	0.77	0.214	0.2170	530
	9.11	0.69	0.191	0.2000	1000
	1.74	3.60	1.000	1.0000	200
	2.40	2.62	0.726	0.7263	110
	3.40	1.85	0.512	0.5066	310
	3.52	1.79	0.496	0.5000	400
	4.85	1.30	0.360	0.3631	220
(e) 80 °C (1st cooling)	5.13	1.22	0.340	0.3559	510
Col$_r$	5.27	1.19	0.331	0.3333	600
a = 7.21 nm, *b* = 2.81 nm, *c* = 0.34 nm	5.64	1.11	0.309	0.3074	420
Z = 2 (ρ = 1.19)	6.46	0.97	0.270	0.2683	710
	7.04	0.89	0.248	0.2500	800
	8.14	0.77	0.214	0.2179	530
	9.22	0.68	0.189	0.2000	1000
	18.5	0.34	–	–	001

Table S4 (Continued)

	q (nm^{-1})	*d*-spacing (nm)	ratio	ratio (calc.)	*hkl*
	1.76	3.58	1.000	1.0000	200
	2.48	2.54	0.709	0.7095	110
	3.45	1.82	0.508	0.5008	310
	3.52	1.79	0.500	0.5000	400
	4.90	1.28	0.358	0.3547	220
(f) 20 °C (1st cooling)	5.12	1.23	0.343	0.3539	510
Col$_r$	5.29	1.19	0.332	0.3333	600
a = 7.16 nm, b = 2.72 nm, c = 0.34 nm	5.76	1.09	0.305	0.3022	420
Z = 2 (ρ = 1.23)	6.52	0.96	0.269	0.2674	710
	6.95	0.90	0.253	0.2509	130
	7.06	0.89	0.249	0.2500	800
	8.18	0.77	0.215	0.2138	530
	9.11	0.69	0.193	0.1897	040
	18.3	0.34	–	–	001
	1.77	3.55	1.000	1.0000	200
	2.51	2.50	0.704	0.7039	110
	3.54	1.78	0.500	0.5000	400
	3.68	1.71	0.481	0.4989	310
	4.95	1.27	0.357	0.3532	510
(g) –20 °C (1st cooling)	5.14	1.22	0.344	0.3520	220
Col$_r$	5.34	1.18	0.331	0.3333	600
a = 7.11 nm, b = 2.67 nm, c = 0.34 nm	6.33	0.99	0.280	0.3005	420
Z = 2 (ρ = 1.25)	6.56	0.96	0.270	0.2671	710
	7.11	0.88	0.249	0.2500	800
	8.24	0.76	0.214	0.2124	530
	9.10	0.69	0.194	0.2000	1000
	18.3	0.34	–	–	001
	1.76	3.58	1.000	1.0000	200
	2.50	2.51	0.702	0.7024	110
	3.53	1.78	0.498	0.5000	400
	3.64	1.73	0.483	0.4983	310
(h) 5 °C (2nd heating)	4.94	1.27	0.356	0.3530	510
Col$_r$	5.13	1.22	0.342	0.3512	220
a = 7.16 nm, b = 2.68 nm, c = 0.34 nm	5.32	1.18	0.330	0.3333	600
Z = 2 (ρ = 1.24)	6.55	0.96	0.268	0.2670	710
	7.09	0.89	0.248	0.2481	130
	8.22	0.76	0.214	0.2120	530
	9.10	0.69	0.193	0.2000	1000
	18.3	0.34	–	–	001
	1.74	3.60	1.000	1.0000	200
	2.40	2.62	0.726	0.7263	110
	3.50	1.79	0.498	0.5000	400
	4.85	1.30	0.360	0.3631	220
(i) 90 °C (2nd heating)	5.08	1.24	0.343	0.3559	510
Col$_r$	5.27	1.19	0.331	0.3333	600
a = 7.21 nm, b = 2.81 nm, c = 0.34 nm	5.64	1.11	0.309	0.3074	420
Z = 2 (ρ = 1.18)	6.46	0.97	0.270	0.2683	710
	7.04	0.89	0.248	0.2500	800
	8.14	0.77	0.214	0.2179	530
	9.14	0.69	0.191	0.1949	040
	18.4	0.34	–	–	001

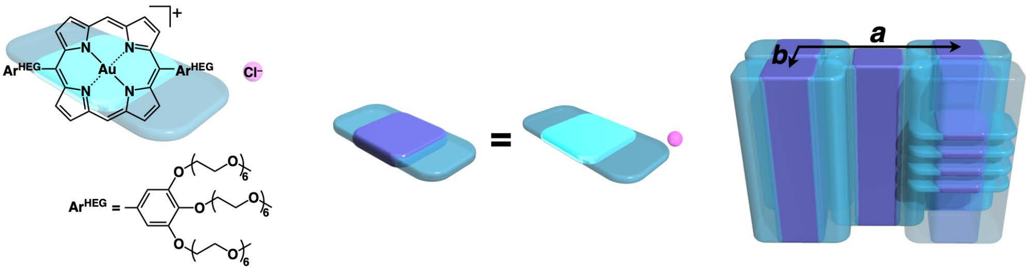

Figure S75 Possible packing model of **2au**$^{+}$-Cl$^{−}$ as a Col$_{r}$ structure.

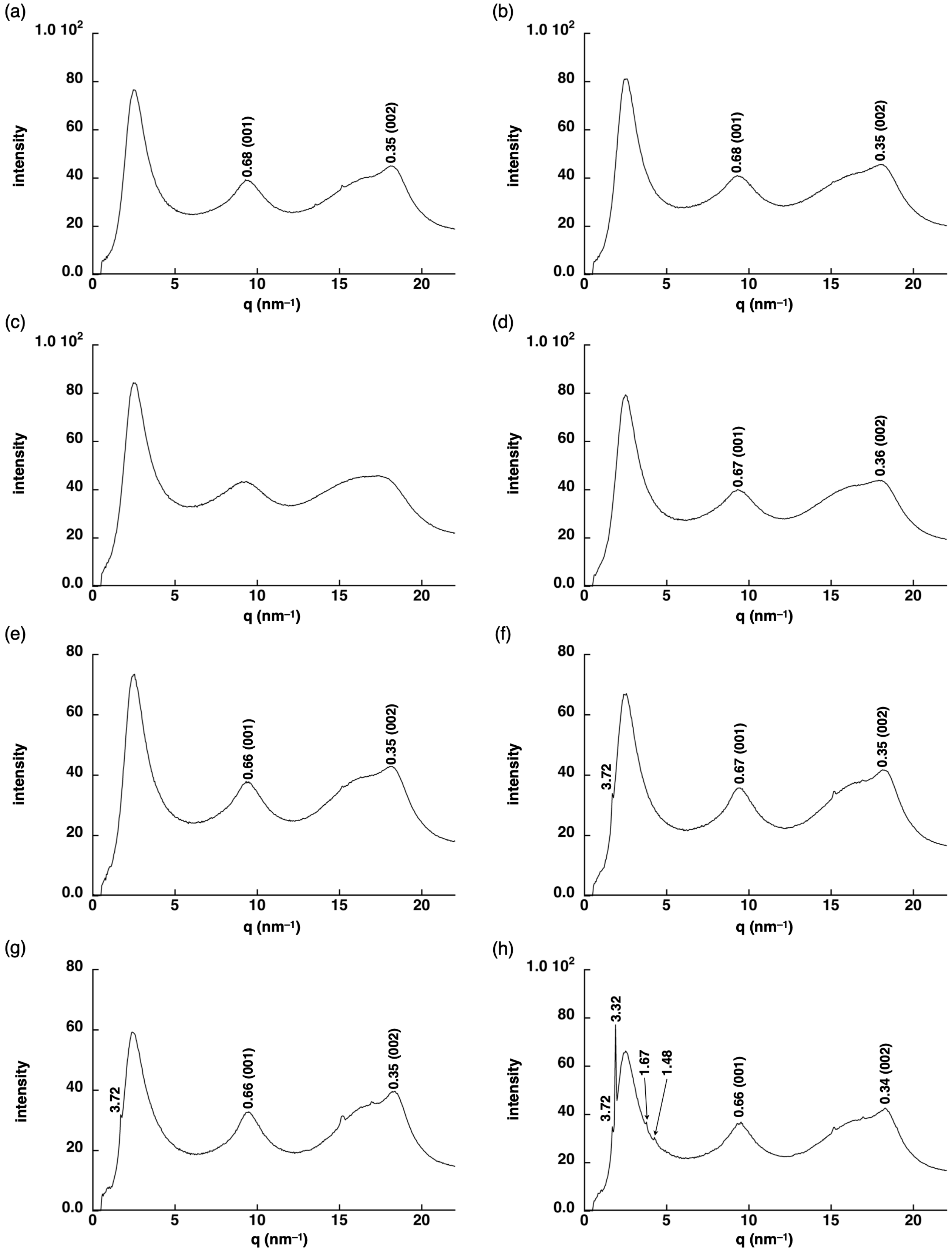

Figure S76 XRD patterns of **2au**$^{+}$-PCCp^{-} at (a) 25 °C, (b) 50 °C, (c) 100 °C, (d) 50 °C, (e) 20 °C, (f) 0 °C, (g) –30 °C, (h) 0 °C, (i) 20 °C, (j) 50 °C, and (k) 100 °C upon (a–c) 1st heating, (d–g) 1st cooling, and (h–k) 2nd heating.

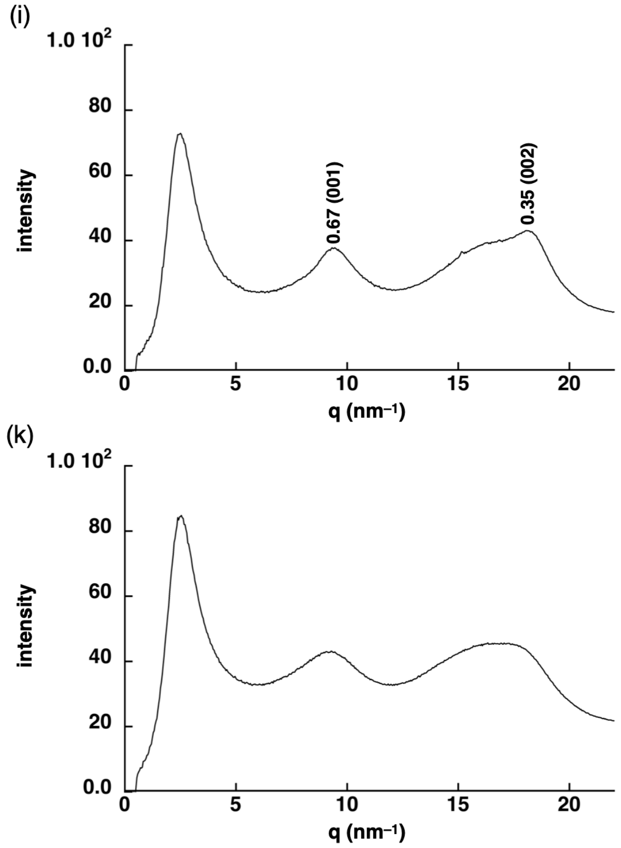

Figure S76 (Continued)

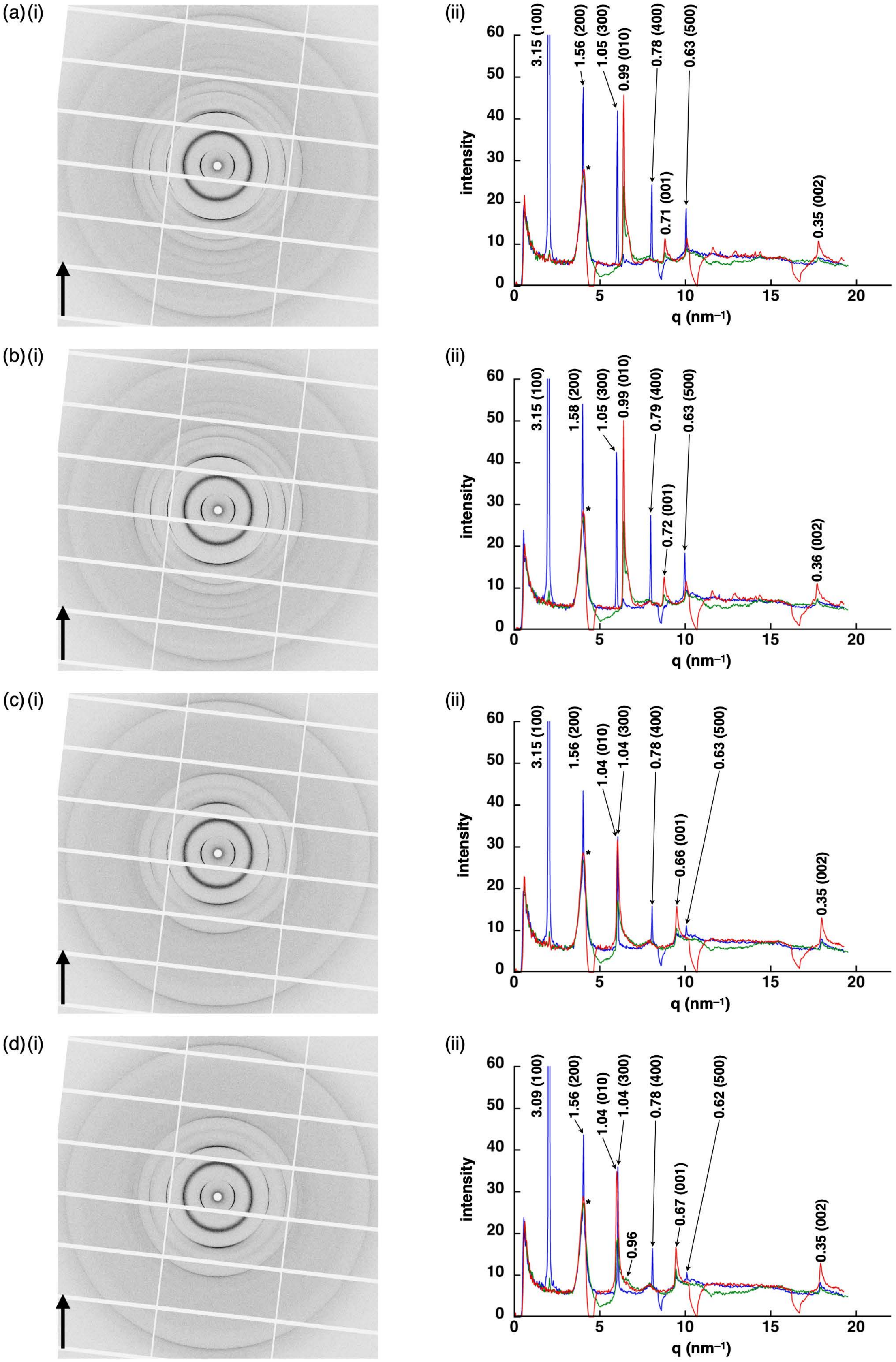

Figure S77 XRD patterns of **1au**$^{+}$-PCCp^{-} sheared between Kapton (polyimide) film at ca. 90 °C, cooled to (a) r.t., and subsequently heated to (b) 50 °C, (c) 80 °C, (d) 100 °C, (e) 125 °C, and (f) 150 °C: (i) 2D XRD diffraction patterns with an arrow indicating shearing (meridional) direction and (ii) corresponding 1D patterns of shearing direction (90° ± 10°, red), 25° away from the shearing direction (115° ± 10°, green), and equatorial direction (0° ± 10°, blue). Diffractions of asterisk indicate those from the polyimide film. The grid-like artifacts from the detector provided no severe problems in the examinations of shearing-induced alignment. The tilted arrangement of the Lam_{col}-H structures could be suggested by the value of 0.96, 0.95, and 0.96 nm observed 25° away from the shearing direction at 100, 125, and 150 °C, respectively.

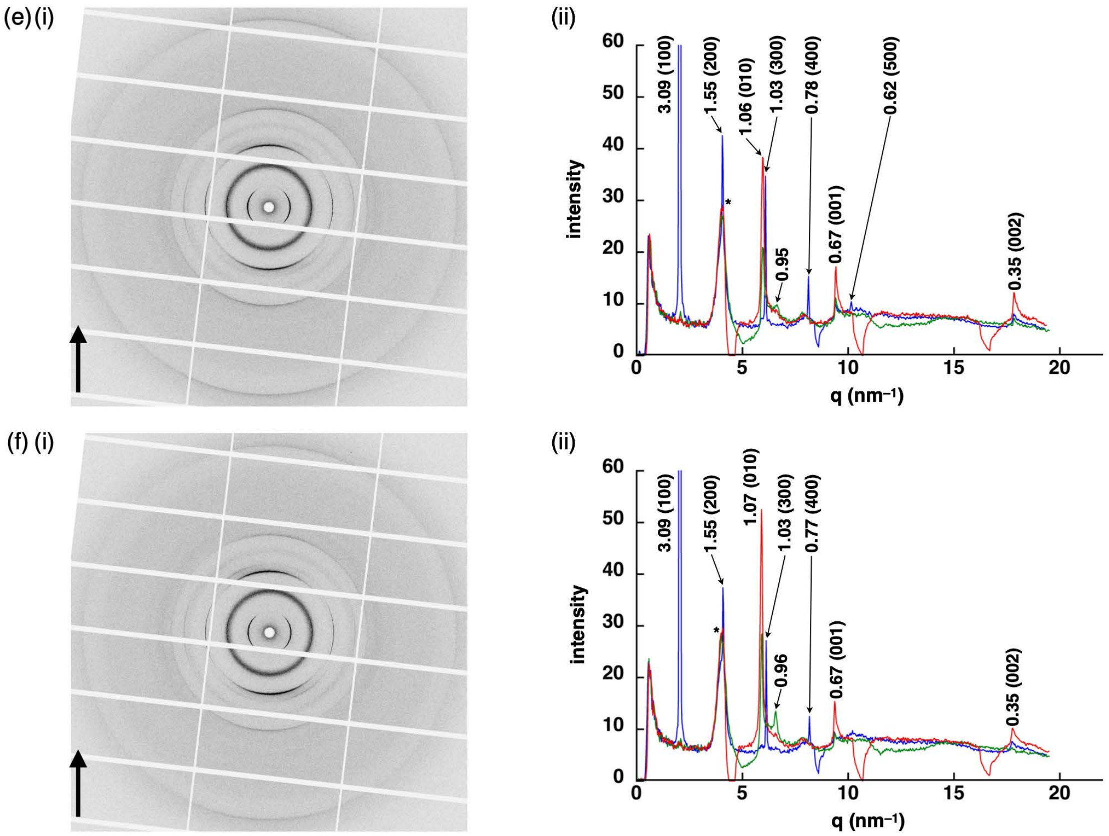

Figure S77 (Continued)

Table S5 Summary of XRD data of **1au**$^+$-PCCp$^-$. The peaks which can be indexed are represented.

	q (nm^{-1})	*d*-spacing (nm)	ratio	ratio (calc.)	*hkl*
(a) 25 °C (heating)<br>Lam$_{col}$-L<br>*a* = 3.15 nm, *b* = 0.99 nm, *c* = 0.71 nm	1.99	3.15	1.000	1.0000	100
	4.02	1.56	0.496	0.5000	200
	6.00	1.05	0.332	0.3333	300
	6.36	0.99	–	–	010
	8.02	0.78	0.248	0.2500	400
	8.79	0.71	–	–	001
	10.0	0.63	0.198	0.2000	500
	17.8	0.35	–	–	002
(b) 50 °C (heating)<br>Lam$_{col}$-L<br>*a* = 3.15 nm, *b* = 0.99 nm, *c* = 0.72 nm	1.99	3.15	1.000	1.0000	100
	3.98	1.58	0.501	0.5000	200
	5.96	1.05	0.334	0.3333	300
	6.36	0.99	–	–	010
	7.98	0.79	0.250	0.2500	400
	8.75	0.72	–	–	001
	9.97	0.63	0.200	0.2000	500
	17.7	0.36	–	–	002
(c) 80 °C (heating)<br>Lam$_{col}$-H<br>*a* = 3.15 nm, *b* = 1.04 nm, *c* = 0.66 nm	1.99	3.15	1.000	1.0000	100
	4.02	1.56	0.496	0.5000	200
	6.04	1.04	0.330	0.3333	300, 010
	8.06	0.78	0.247	0.2500	400
	9.48	0.66	–	–	001
	10.0	0.63	0.198	0.2000	500
	18.0	0.35	–	–	002

Table S5 (Continued)

	q (nm^{-1})	*d*-spacing (nm)	ratio	ratio (calc.)	*hkl*
(d) 100 °C (heating) Lam$_{col}$-H a = 3.09 nm, b = 1.04 nm, c = 0.67 nm	2.03	3.09	1.000	1.0000	100
	4.02	1.56	0.506	0.5000	200
	6.04	1.04	0.337	0.3333	300, 010
	6.53	0.96	–	–	–
	8.06	0.78	0.252	0.2500	400
	9.44	0.67	–	–	001
	10.1	0.62	0.202	0.2000	500
	17.9	0.35	–	–	002
(e) 125 °C (heating) Lam$_{col}$-H a = 3.09 nm, b = 1.06 nm, c = 0.67 nm	2.03	3.09	1.000	1.0000	100
	4.06	1.55	0.501	0.5000	200
	5.92	1.06	–	–	010
	6.08	1.03	0.334	0.3333	300
	6.61	0.95	–	–	–
	8.10	0.78	0.251	0.2500	400
	9.40	0.67	–	–	001
	10.1	0.62	0.201	0.2000	500
	17.8	0.35	–	–	002
(f) 150 °C (heating) Lam$_{col}$-H a = 3.09 nm, b = 1.07 nm, c = 0.67 nm	2.03	3.09	1.000	1.0000	100
	4.06	1.55	0.501	0.5000	200
	5.88	1.07	–	–	010
	6.12	1.03	0.332	0.3333	300
	6.53	0.96	–	–	–
	8.14	0.77	0.250	0.2500	400
	9.32	0.67	–	–	001
	17.7	0.35	–	–	002

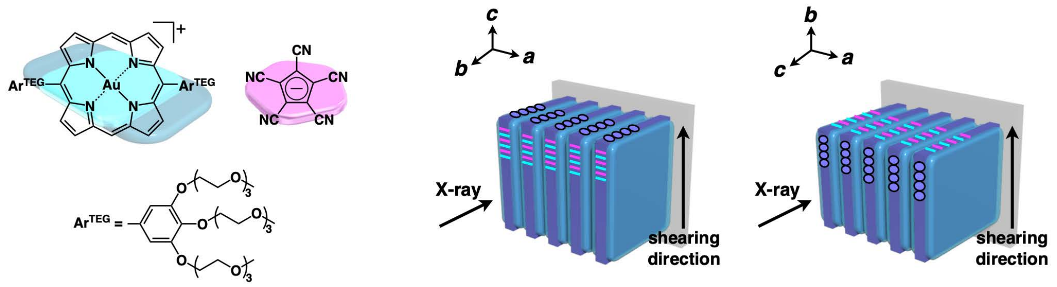

Figure S78 Possible shearing-induced alignment models of **1au**$^+$-PCCp$^-$ as a Lam$_{col}$ structure.

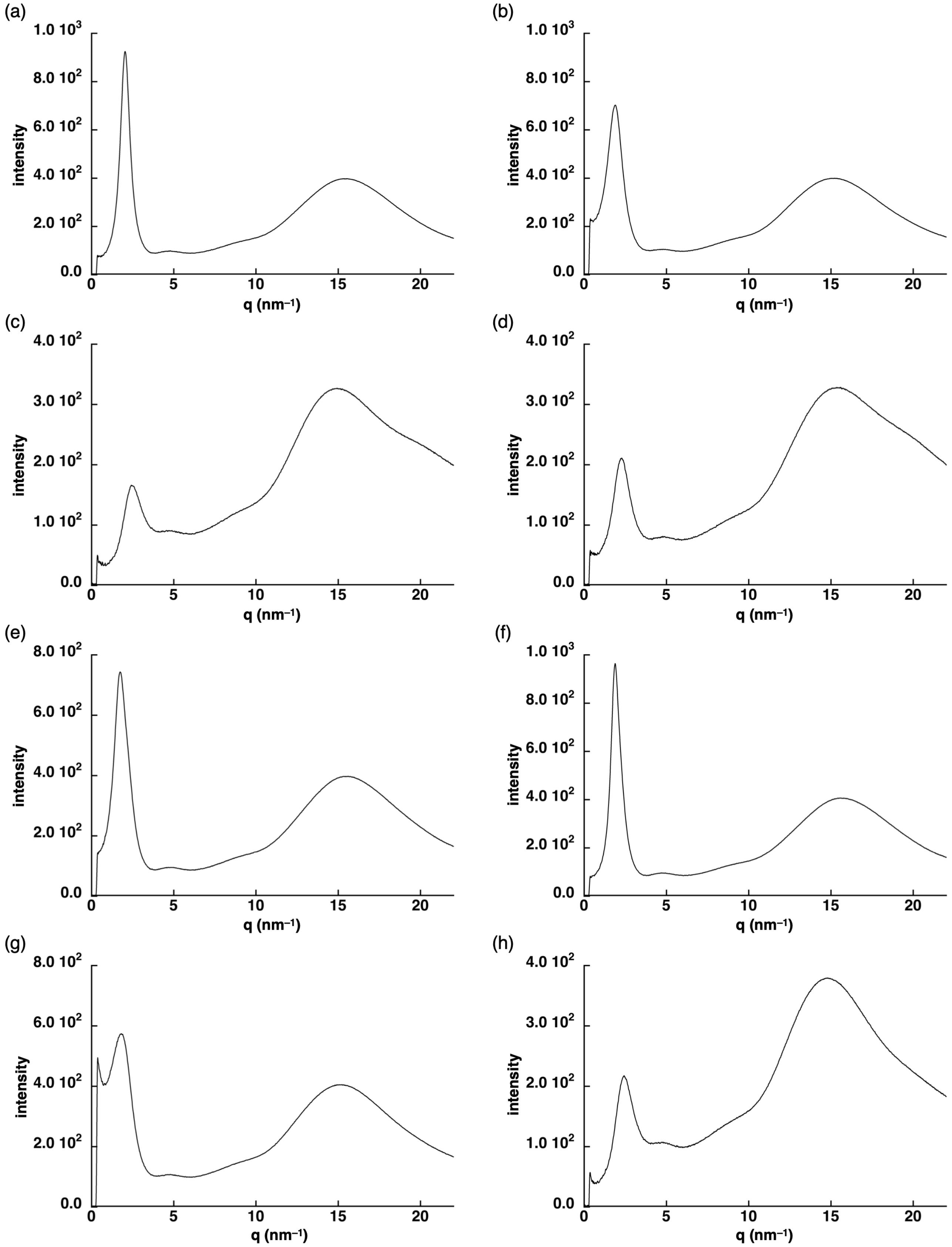

Figure S79 XRD patterns of **1**$_{70\%}$ at (a) 25 °C, (b) 50 °C, (c) 80 °C, (d) 50 °C, (e) 20 °C, (f) 5 °C, (g) 50 °C, and (h) 80 °C upon (a–c) 1st heating, (d–f) 1st cooling, and (g,h) 2nd heating.

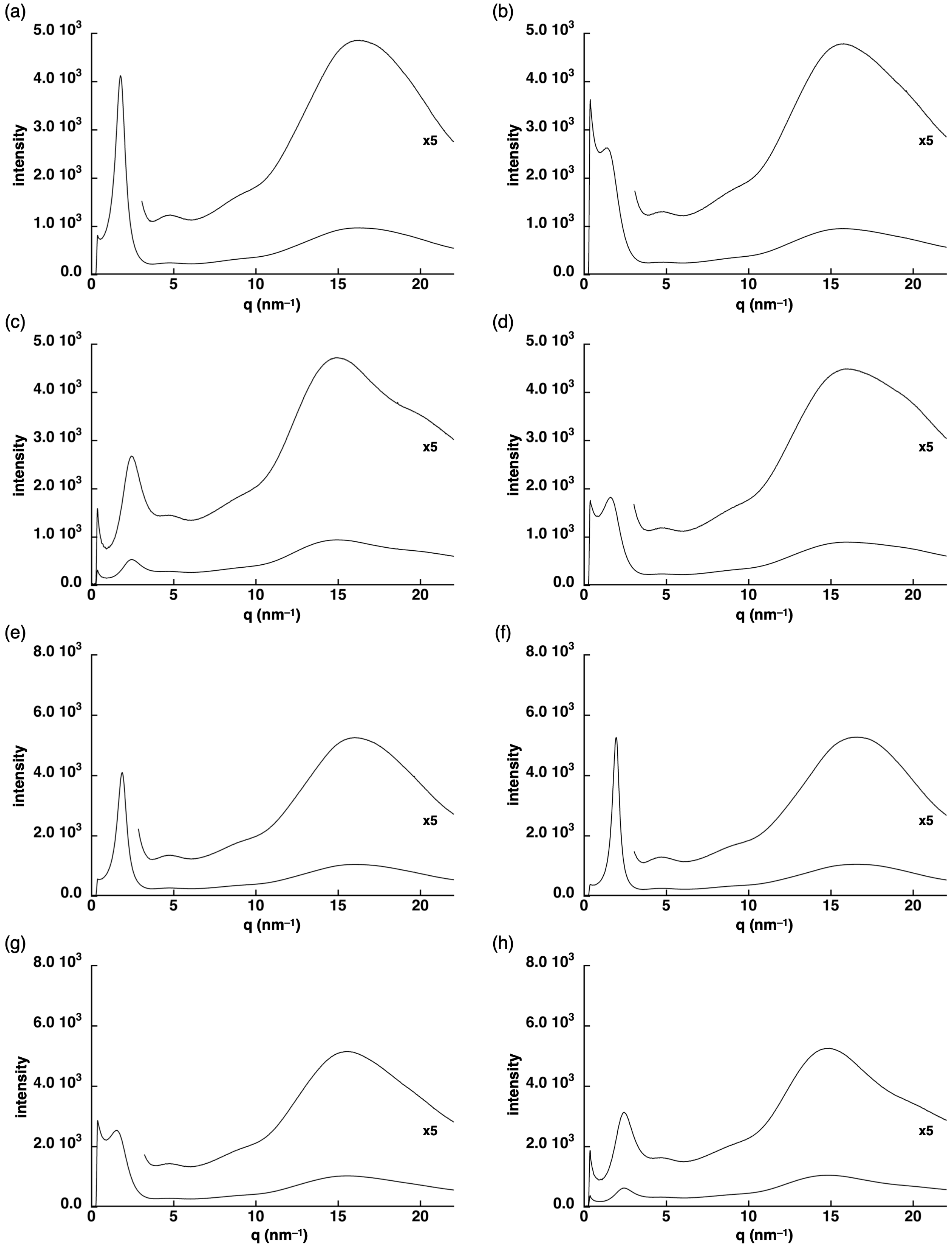

Figure S80 XRD patterns of **1**$_{50\%}$ at (a) 25 °C, (b) 50 °C, (c) 80 °C, (d) 50 °C, (e) 20 °C, (f) 5 °C, (g) 50 °C, and (h) 80 °C upon (a–c) 1st heating, (d–f) 1st cooling, and (g,h) 2nd heating.

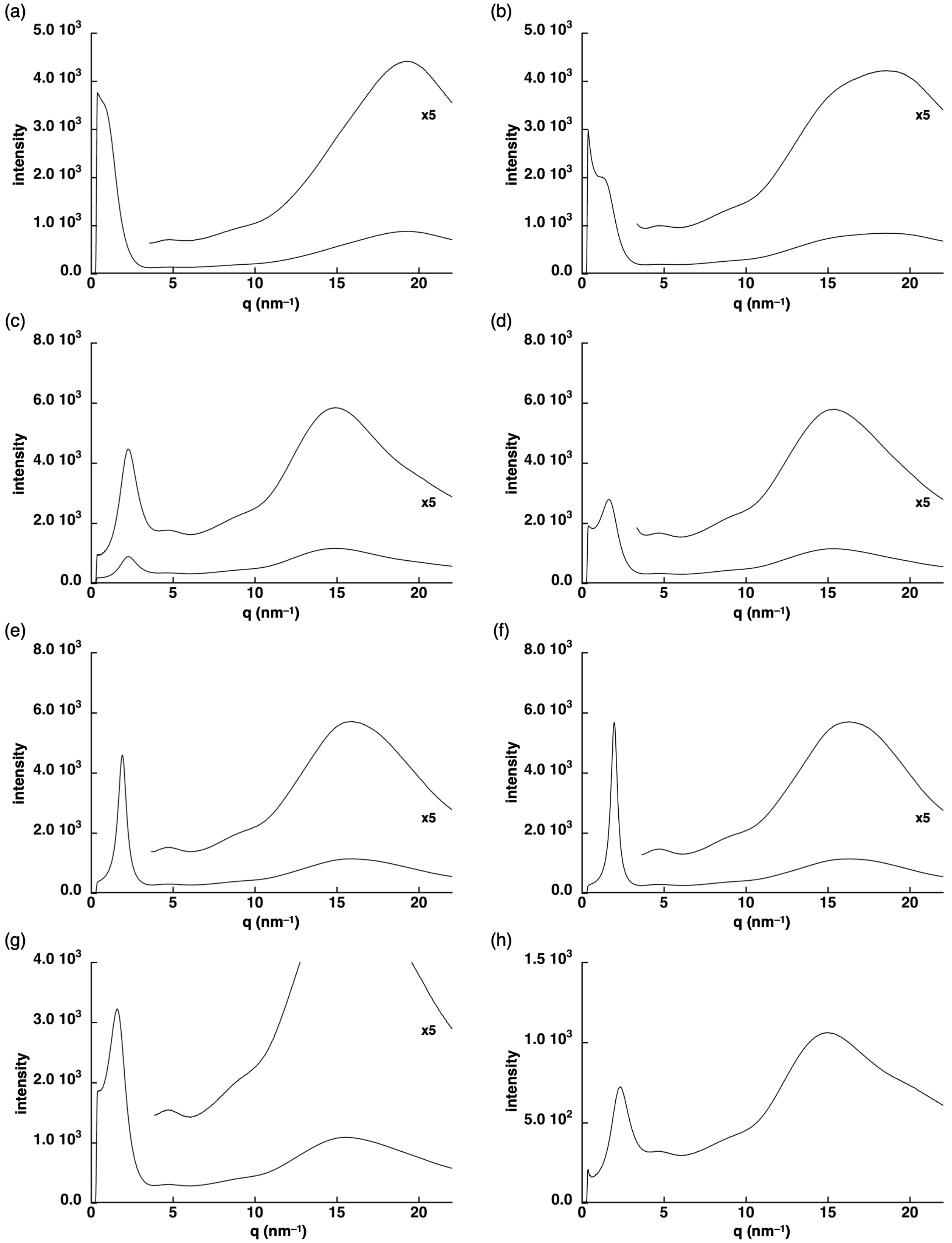

Figure S81 XRD patterns of $\mathbf{1}_{20\%}$ at (a) 25 °C, (b) 50 °C, (c) 80 °C, (d) 50 °C, (e) 20 °C, (f) 5 °C, (g) 50 °C, and (h) 80 °C upon (a–c) 1st heating, (d–f) 1st cooling, and (g,h) 2nd heating.

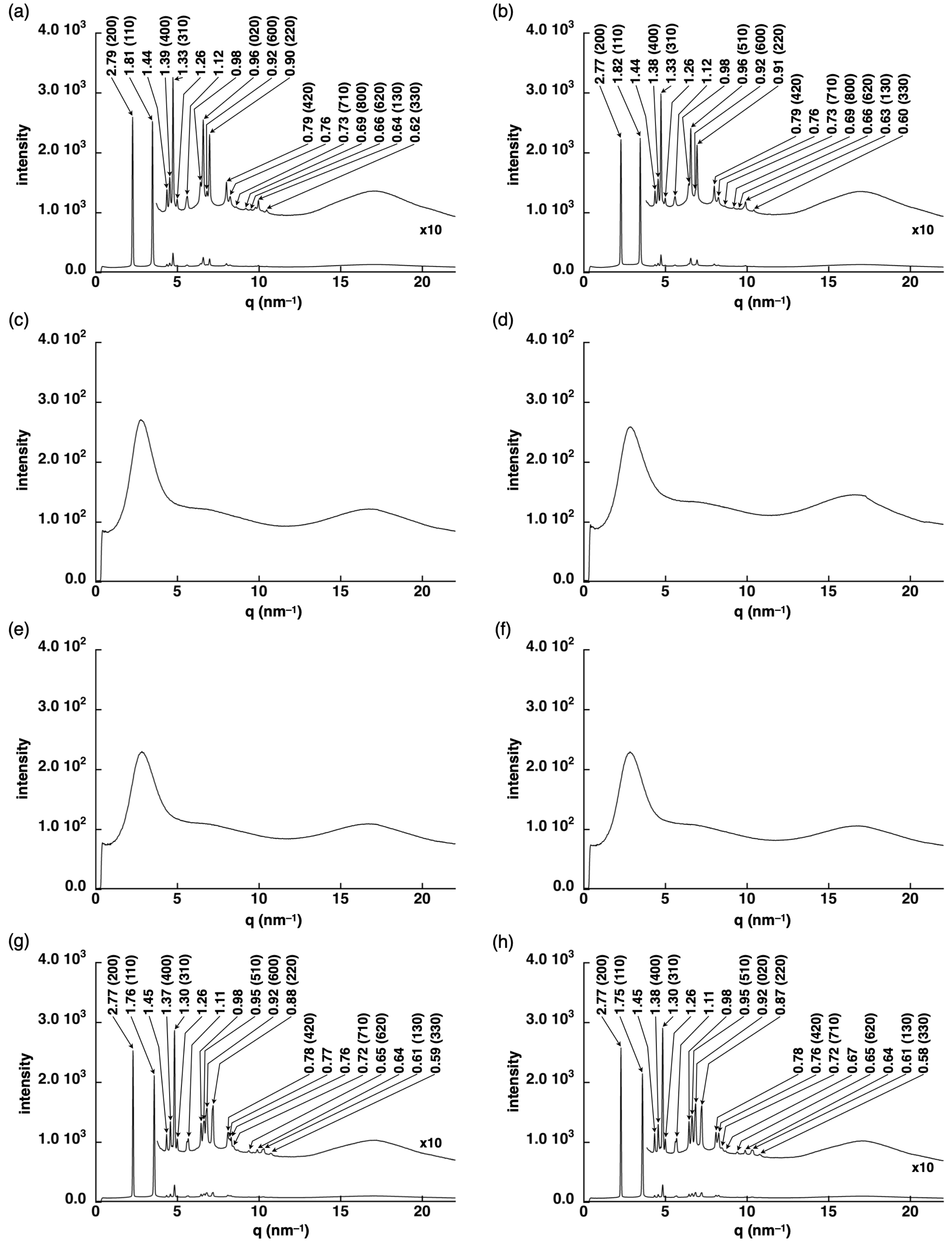

Figure S82 XRD patterns of **1au**$^+$-Cl$^-_{75\%}$ at (a) 25 °C, (b) 35 °C, (c) 60 °C, (d) 80 °C, (e) 60 °C, (f) 50 °C, (g) 30 °C, (h) 20 °C, (i) 5 °C, (j) 20 °C, (k) 35 °C, (l) 60 °C, and (m) 80 °C upon (a–d) 1st heating, (e–i) 1st cooling, and (j–m) 2nd heating.

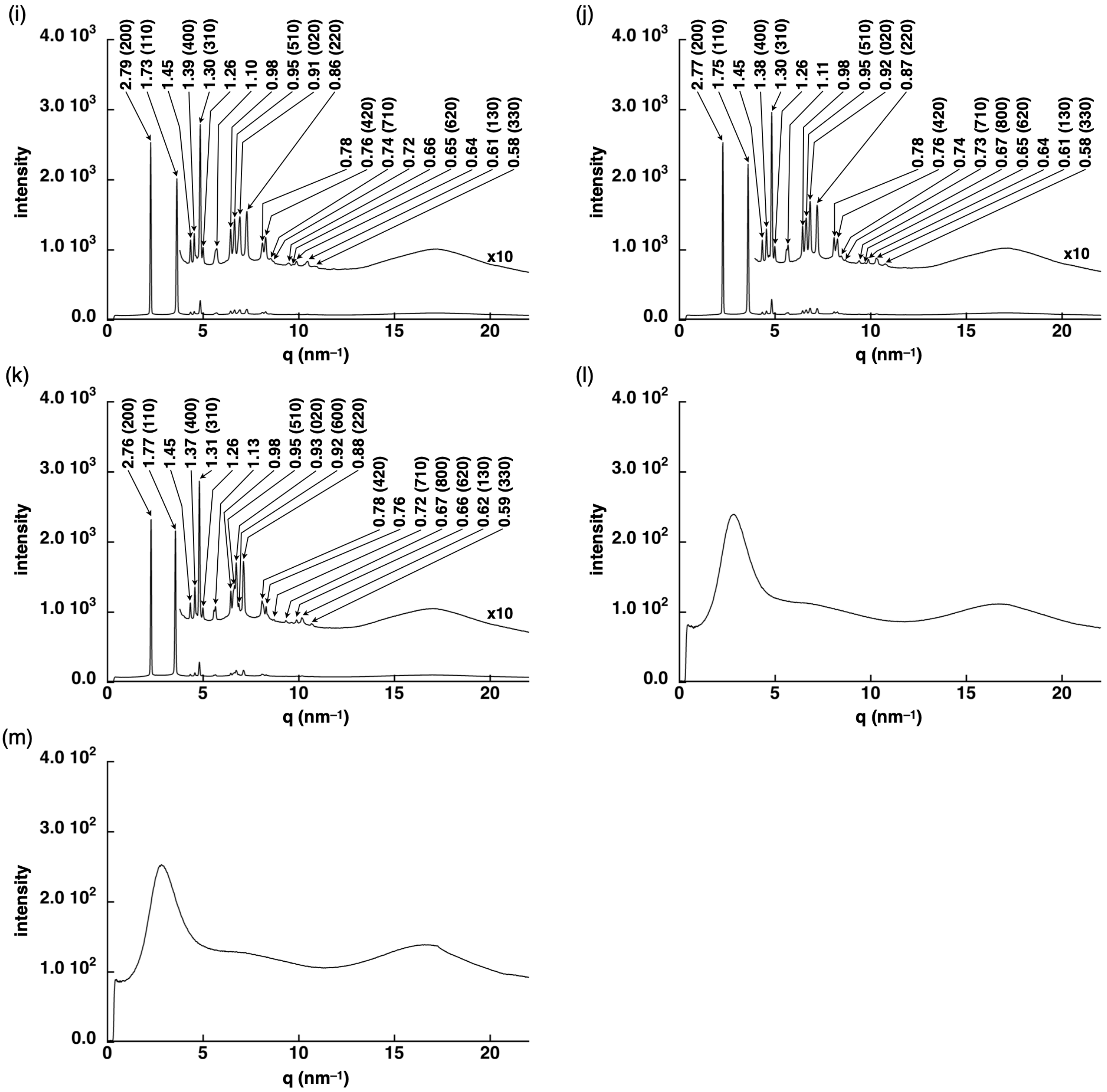

Figure S82 (Continued)

Table S6 Summary of XRD data of **1au**$^{+}$-Cl$^{-}_{75\%}$. The peaks which can be indexed are represented.

	q (nm^{-1})	*d*-spacing (nm)	ratio	ratio (calc.)	*hkl*
	2.26	2.79	1.000	1.0000	200
	3.47	1.81	0.651	0.6507	110
	4.52	1.39	0.499	0.5000	400
	4.72	1.33	0.477	0.4788	310
	6.58	0.96	0.343	0.3441	020
(a) 25 °C (1st heating)	6.80	0.92	0.332	0.3333	600
Col$_r$	6.96	0.90	0.324	0.3253	220
a = 5.57 nm, *b* = 1.92 nm	8.00	0.79	0.282	0.2834	420
	8.59	0.73	0.262	0.2639	710
	9.17	0.69	0.246	0.2500	800
	9.48	0.66	0.238	0.2394	620
	9.78	0.64	0.231	0.2279	130
	10.2	0.62	0.221	0.2169	330
	2.27	2.77	1.000	1.0000	200
	3.45	1.82	0.657	0.6568	110
	4.55	1.38	0.499	0.5000	400
	4.72	1.33	0.480	0.4812	310
	6.55	0.96	0.346	0.3467	510
(b) 35 °C (1st heating)	6.84	0.92	0.332	0.3333	600
Col$_r$	6.93	0.91	0.327	0.3284	220
a = 5.54 nm, *b* = 1.93 nm	7.98	0.79	0.284	0.2854	420
	8.63	0.73	0.263	0.2643	710
	9.17	0.69	0.247	0.2500	800
	9.48	0.66	0.239	0.2406	620
	9.90	0.63	0.229	0.2302	130
	10.4	0.60	0.218	0.2189	330
	2.27	2.77	1.000	1.0000	200
	3.57	1.76	0.636	0.6359	110
	4.57	1.37	0.496	0.5000	400
	4.82	1.30	0.470	0.4728	310
(g) 30 °C (1st cooling)	6.63	0.95	0.342	0.3435	510
Col$_r$	6.80	0.92	0.333	0.3333	600
a = 5.54 nm, *b* = 1.86 nm	7.18	0.88	0.316	0.3179	220
	8.08	0.78	0.281	0.2785	420
	8.69	0.72	0.261	0.2629	710
	9.63	0.65	0.236	0.2364	620
	10.3	0.61	0.220	0.2222	130
	10.7	0.59	0.212	0.2120	330
	2.27	2.77	1.000	1.0000	200
	3.59	1.75	0.631	0.6314	110
	4.56	1.38	0.497	0.5000	400
	4.82	1.30	0.470	0.4710	310
(h) 20 °C (1st cooling)	6.63	0.95	0.342	0.3428	510
Col$_r$	6.84	0.92	0.332	0.3327	020
a = 5.54 nm, *b* = 1.84 nm	7.23	0.87	0.314	0.3157	220
	8.26	0.76	0.275	0.2770	420
	8.68	0.72	0.261	0.2625	710
	9.68	0.65	0.234	0.2355	620
	10.3	0.61	0.221	0.2205	130
	10.8	0.58	0.211	0.2105	330

Table S6 (Continued)

	q (nm^{-1})	*d*-spacing (nm)	ratio	ratio (calc.)	*hkl*
(i) 5 °C (1st cooling) Col$_r$ a = 5.57 nm, b = 1.82 nm	2.26	2.79	1.000	1.0000	200
	3.63	1.73	0.621	0.6214	110
	4.54	1.39	0.497	0.5000	400
	4.85	1.30	0.465	0.4668	310
	6.64	0.95	0.340	0.3412	510
	6.91	0.91	0.326	0.3269	020
	7.29	0.86	0.309	0.3107	220
	8.26	0.76	0.273	0.2736	420
	8.53	0.74	0.264	0.2618	710
	9.68	0.65	0.233	0.2334	620
	10.3	0.61	0.220	0.2166	130
	10.8	0.58	0.208	0.2071	330
(j) 20 °C (2nd heating) Col$_r$ a = 5.54 nm, b = 1.84 nm	2.27	2.77	1.000	1.0000	200
	3.59	1.75	0.631	0.6314	110
	4.55	1.38	0.499	0.5000	400
	4.82	1.30	0.470	0.4710	310
	6.63	0.95	0.342	0.3428	510
	6.83	0.92	0.332	0.3327	020
	7.19	0.87	0.315	0.3157	220
	8.26	0.76	0.275	0.2770	420
	8.67	0.73	0.262	0.2625	710
	9.38	0.67	0.242	0.2500	800
	9.67	0.65	0.235	0.2355	620
	10.3	0.61	0.221	0.2205	130
	10.8	0.58	0.211	0.2105	330
(k) 35 °C (2nd heating) Col$_r$ a = 5.51 nm, b = 1.87 nm	2.28	2.76	1.000	1.0000	200
	3.55	1.77	0.642	0.6417	110
	4.57	1.37	0.499	0.5000	400
	4.80	1.31	0.475	0.4752	310
	6.64	0.95	0.343	0.3444	510
	6.73	0.93	0.339	0.3387	020
	6.86	0.92	0.332	0.3333	600
	7.10	0.88	0.321	0.3208	220
	8.07	0.78	0.283	0.2804	420
	8.70	0.72	0.262	0.2633	710
	9.32	0.67	0.245	0.2500	800
	9.59	0.66	0.238	0.2376	620
	10.2	0.62	0.225	0.2244	130
	10.7	0.59	0.214	0.2139	330

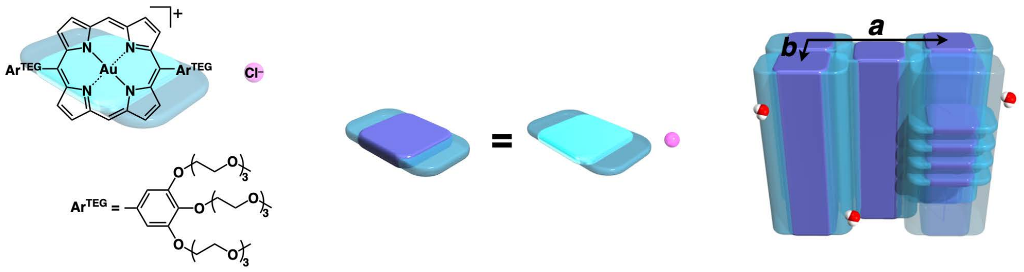

Figure S83 Possible packing model of **1au**$^+$-Cl$^-_{75\%}$ as a Col$_r$ structure.

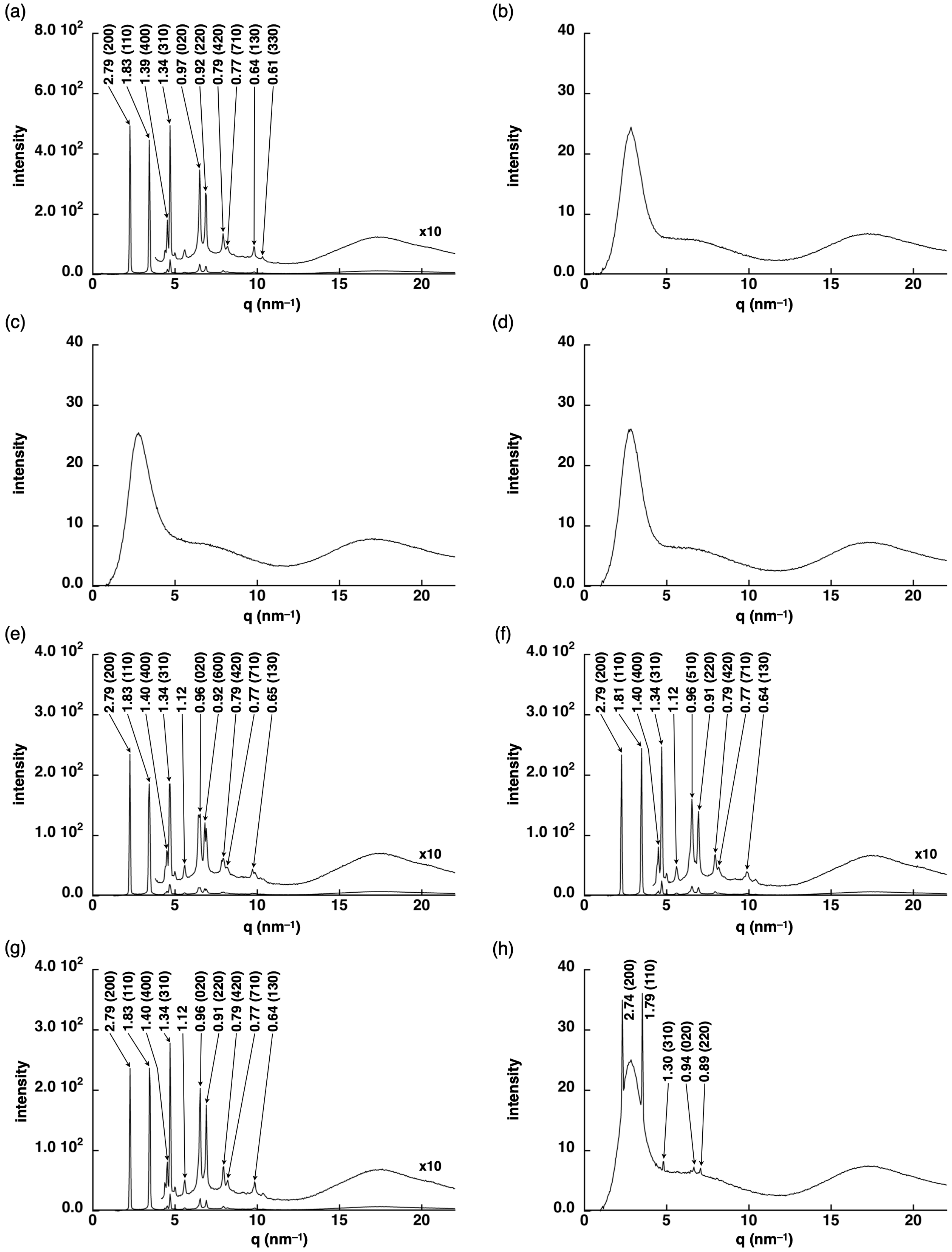

Figure S84 XRD patterns of **1au**$^+$-Cl$^-_{70\%}$ at (a) 25 °C, (b) 40 °C, (c) 80 °C, (d) 40 °C, (e) 20 °C, (f) 5 °C, (g) 20 °C, (h) 40 °C, (i) 60 °C, and (j) 80 °C upon (a–c) 1st heating, (d–f) 1st cooling, and (g–j) 2nd heating.

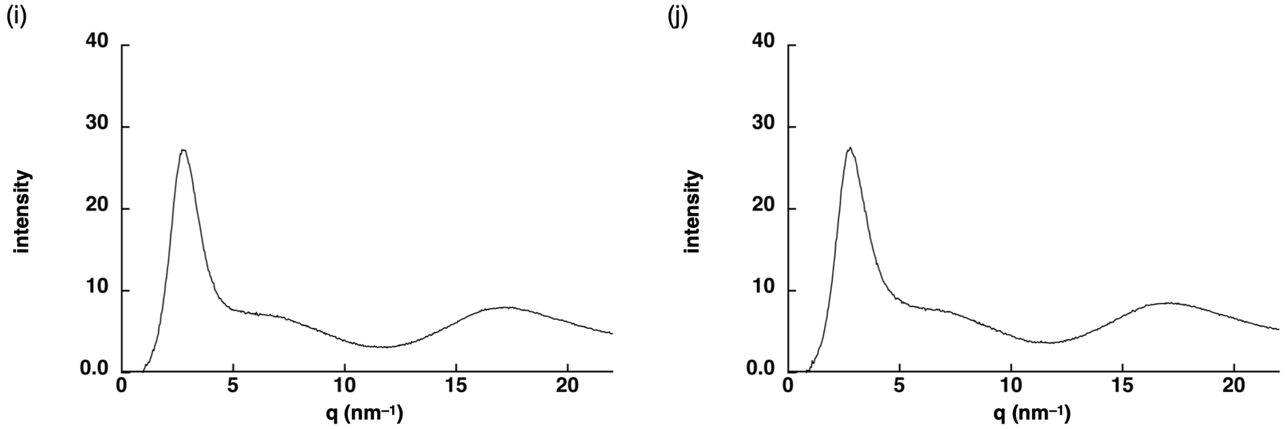

Figure S84 (Continued)

Table S7 Summary of XRD data of **1au**$^{+}$-Cl^{-}$_{70\%}$. The peaks which can be indexed are represented.

	q (nm^{-1})	*d*-spacing (nm)	ratio	ratio (calc.)	*hkl*
(a) 25 °C (1st heating) Col$_r$ *a* = 5.58 nm, *b* = 1.94 nm	2.25	2.79	1.000	1.0000	200
	3.44	1.83	0.656	0.6562	110
	4.53	1.39	0.497	0.5000	400
	4.70	1.34	0.480	0.4810	310
	6.49	0.97	0.347	0.3473	020
	6.86	0.92	0.329	0.3281	220
	7.92	0.79	0.285	0.2852	420
	8.20	0.77	0.275	0.2642	710
	9.79	0.64	0.230	0.2300	130
	10.3	0.61	0.218	0.2187	330
(e) 20 °C (1st cooling) Col$_r$ *a* = 5.58 nm, *b* = 1.94 nm	2.25	2.79	1.000	1.0000	200
	3.44	1.83	0.656	0.6562	110
	4.49	1.40	0.502	0.5000	400
	4.70	1.34	0.480	0.4810	310
	6.53	0.96	0.345	0.3473	020
	6.82	0.92	0.331	0.3333	600
	7.92	0.79	0.285	0.2852	420
	8.16	0.77	0.276	0.2642	710
	9.71	0.65	0.232	0.2300	130
(f) 5 °C (1st cooling) Col$_r$ *a* = 5.58 nm, *b* = 1.91 nm	2.25	2.79	1.000	1.0000	200
	3.48	1.81	0.648	0.6484	110
	4.49	1.40	0.502	0.5000	400
	4.70	1.34	0.480	0.4779	310
	6.53	0.96	0.345	0.3455	510
	6.94	0.91	0.325	0.3242	220
	7.96	0.79	0.283	0.2827	420
	8.16	0.77	0.276	0.2637	710
	9.87	0.64	0.228	0.2270	130
(g) 20 °C (2nd heating) Col$_r$ *a* = 5.58 nm, *b* = 1.94 nm	2.25	2.79	1.000	1.0000	200
	3.44	1.83	0.656	0.6562	110
	4.49	1.40	0.502	0.5000	400
	4.70	1.34	0.480	0.4810	310
	6.53	0.96	0.345	0.3473	020
	6.90	0.91	0.327	0.3281	220
	7.96	0.79	0.283	0.2852	420
	8.20	0.77	0.275	0.2642	710
	9.83	0.64	0.229	0.2300	130

Table S7 (Continued)

	q (nm^{-1})	*d*-spacing (nm)	ratio	ratio (calc.)	*hkl*
	2.29	2.74	1.000	1.0000	200
(h) 40 °C (2nd heating)	3.52	1.79	0.653	0.6525	110
Col_r	4.82	1.30	0.476	0.4795	310
a = 5.48 nm, b = 1.89 nm	6.65	0.94	0.345	0.3451	020
	7.06	0.89	0.325	0.3263	220

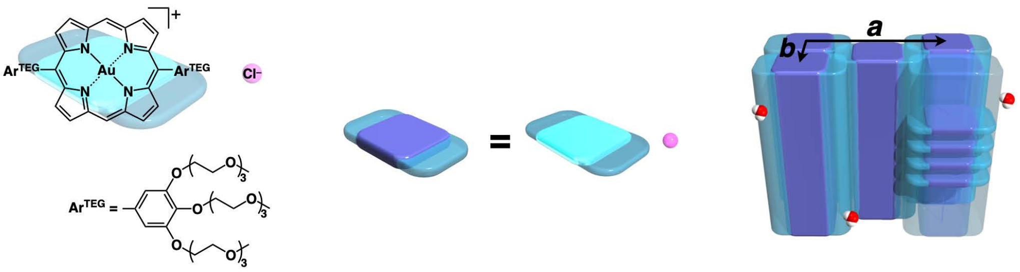

Figure S85 Possible packing model of **1au**$^+$-Cl$^-_{70\%}$ as a Col_r structure.

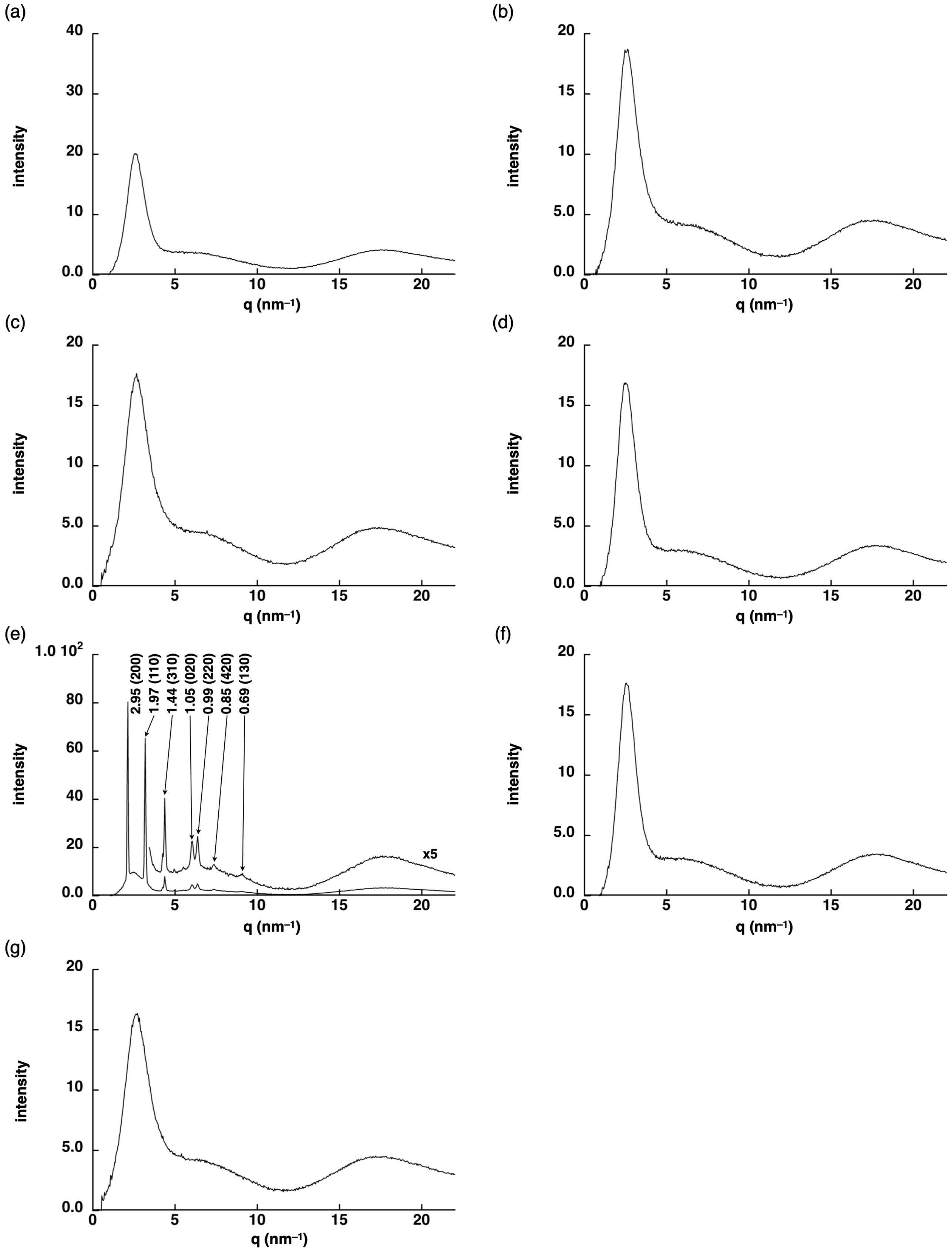

Figure S86 XRD patterns of **1au**$^{+}$-Cl$^{-}_{60\%}$ at (a) 25 °C, (b) 60 °C, (c) 80 °C, (d) 20 °C, (e) 5 °C, (f) 20 °C, and (g) 80 °C upon (a–c) 1st heating, (d,e) 1st cooling, and (f,g) 2nd heating.

Table S8 Summary of XRD data of **1au**$^{+}$-Cl^{-}$_{60\%}$. The peaks which can be indexed are represented.

	q (nm^{-1})	*d*-spacing (nm)	ratio	ratio (calc.)	*hkl*
	2.13	2.95	1.000	1.0000	200
	3.19	1.97	0.668	0.6681	110
(e) 5 °C (1st cooling)	4.37	1.44	0.488	0.4856	310
Col$_{r}$	6.00	1.05	0.355	0.3544	020
a = 5.89 nm, *b* = 2.09 nm	6.37	0.99	0.335	0.3340	220
	7.39	0.85	0.289	0.2891	420
	9.10	0.69	0.234	0.2346	130

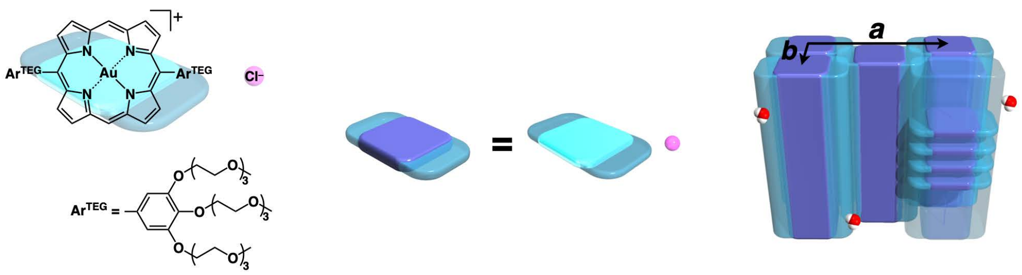

Figure S87 Possible packing model of **1au**$^{+}$-Cl^{-}$_{60\%}$ as a Col$_{r}$ structure.

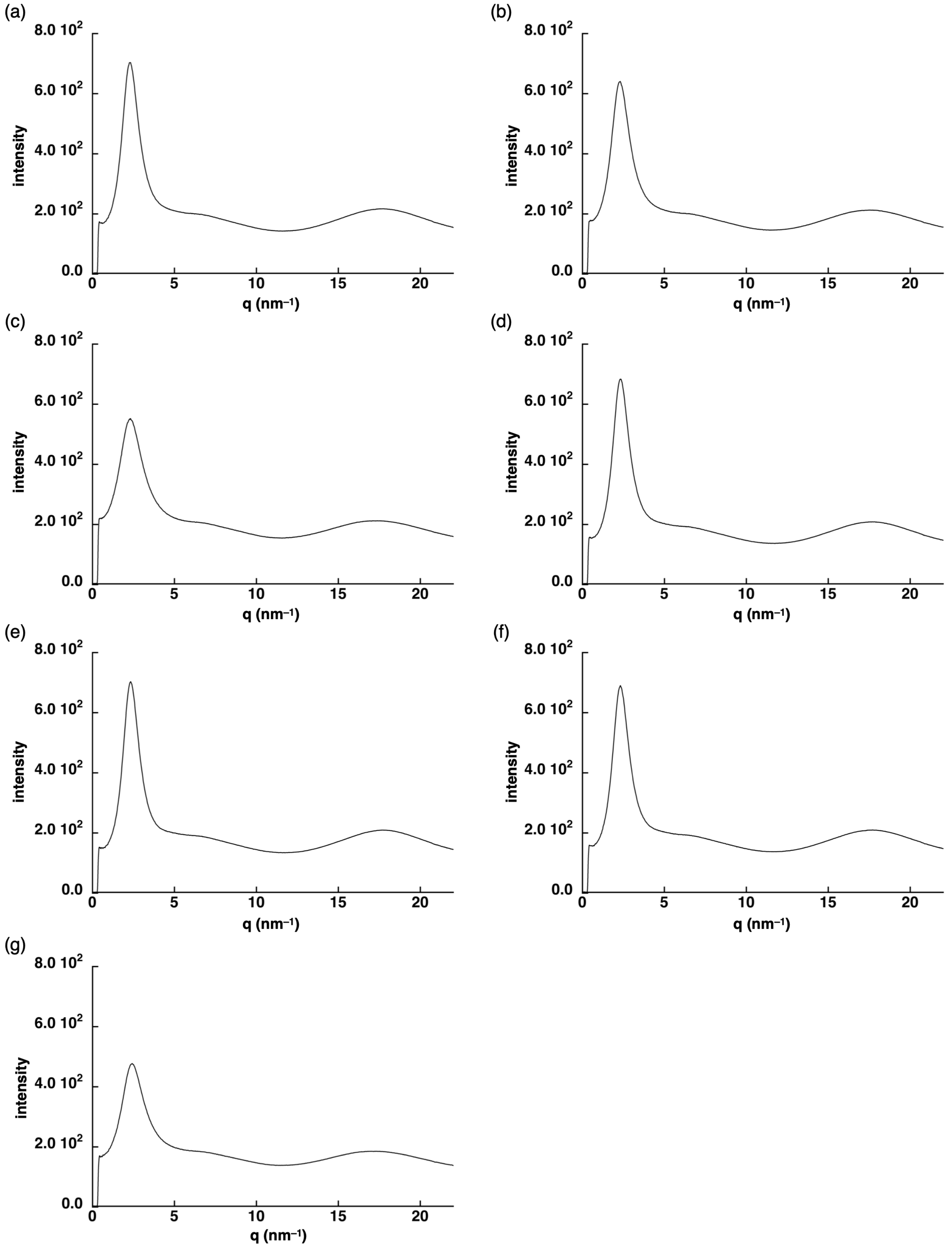

Figure S88 XRD patterns of **1au**$^{+}$-Cl$^{-}_{50\%}$ at (a) 25 °C, (b) 50 °C, (c) 80 °C, (d) 20 °C, (e) 5 °C, (f) 20 °C, and (g) 80 °C upon (a–c) 1st heating, (d,e) 1st cooling, and (f,g) 2nd heating.

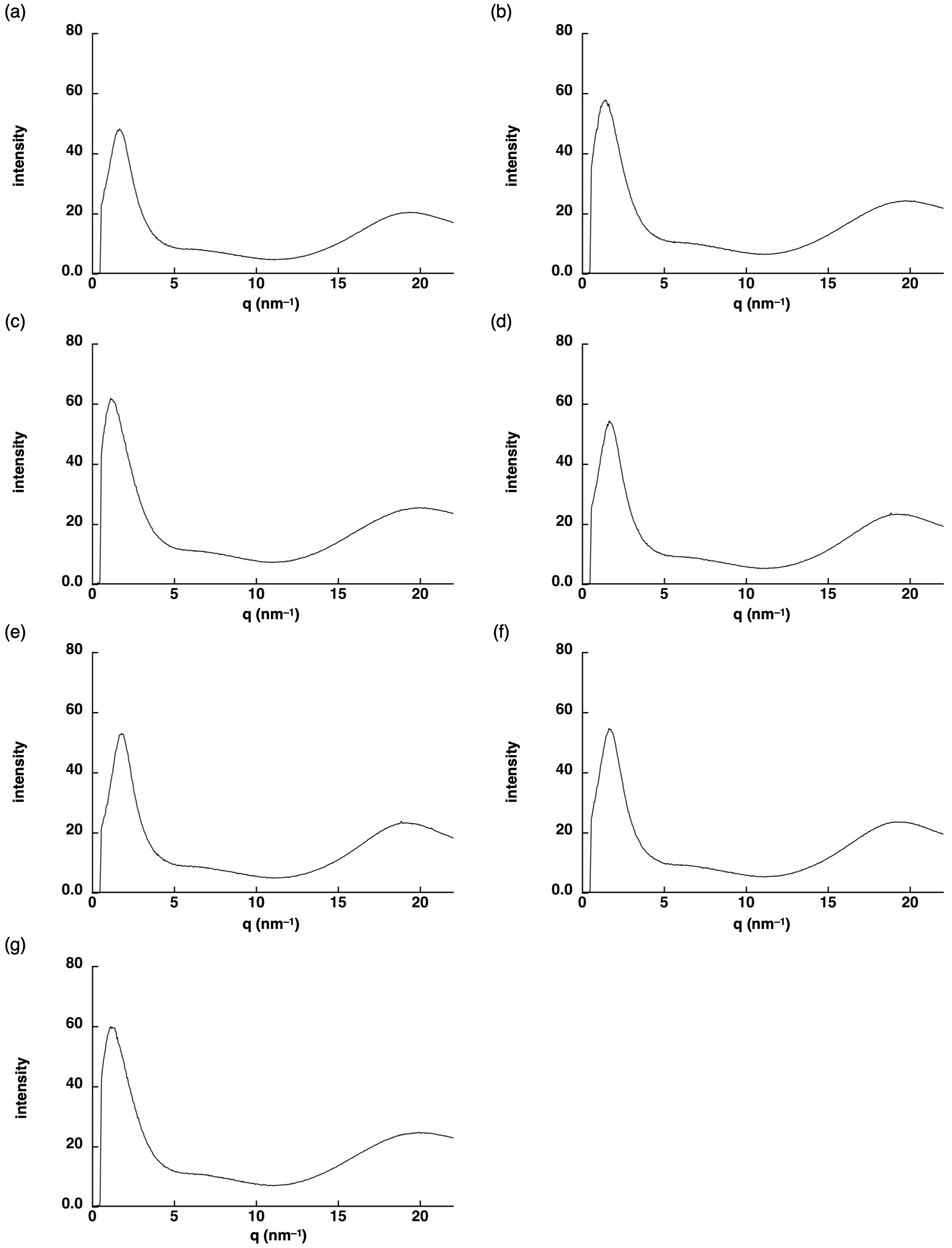

Figure S89 XRD patterns of **1au**$^{+}$-Cl^{-}$_{20\%}$ at (a) 25 °C, (b) 60 °C, (c) 80 °C, (d) 20 °C, (e) 5 °C, (f) 20 °C, and (g) 80 °C upon (a–c) 1st heating, (d,e) 1st cooling, and (f,g) 2nd heating.

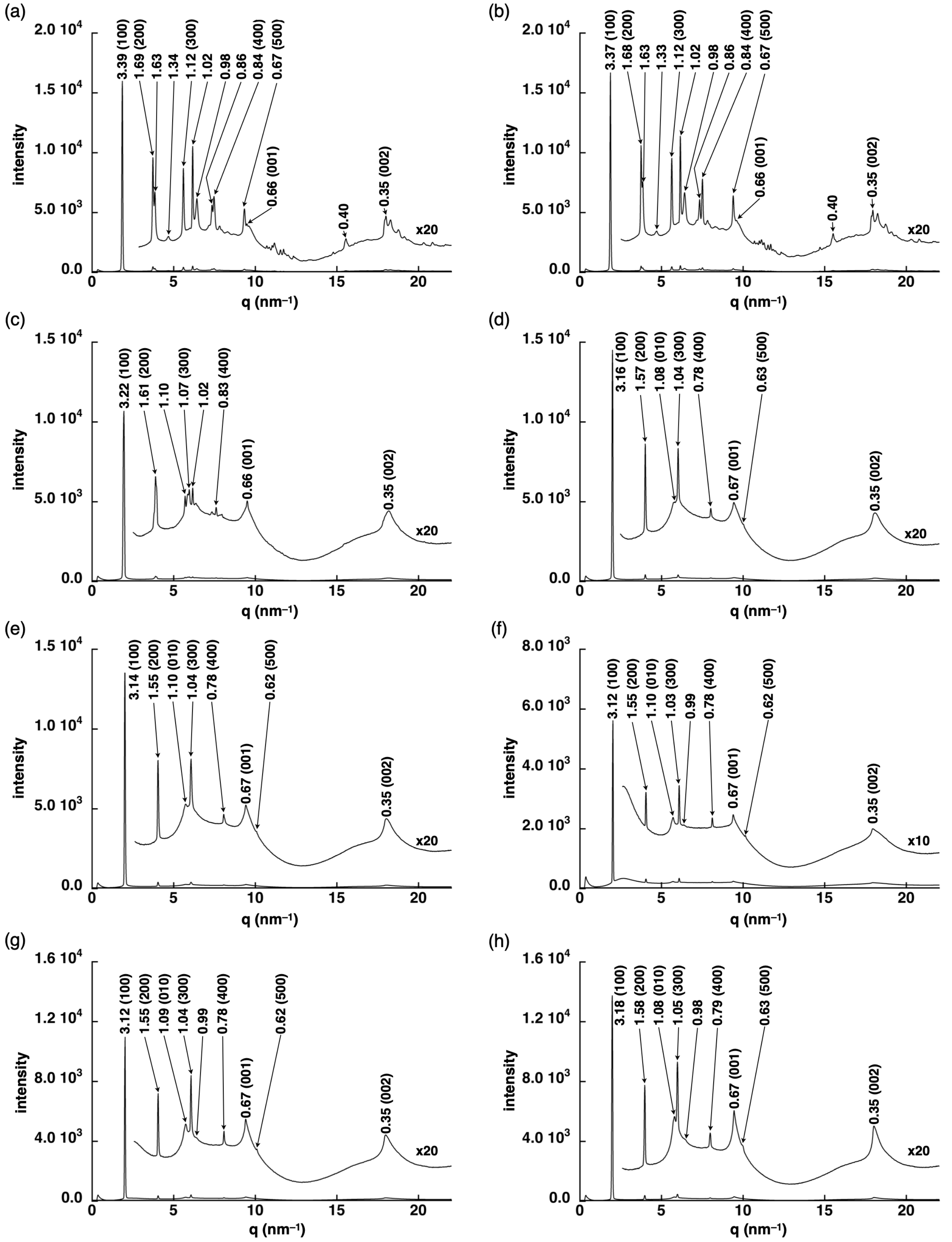

Figure S90 XRD patterns of **1au**$^+$-PCCp$^-_{70\%}$ at (a) 25 °C, (b) 50 °C, (c) 60 °C, (d) 70 °C, (e) 80 °C, (f) 90 °C, (g) 80 °C, (h) 60 °C, (i) 40 °C, (j) 20 °C, (k) 5 °C, (l) 20 °C, (m) 40 °C, (n) 60 °C, and (o) 80 °C upon (a–f) 1st heating, (g–k) 1st cooling, and (l–o) 2nd heating.

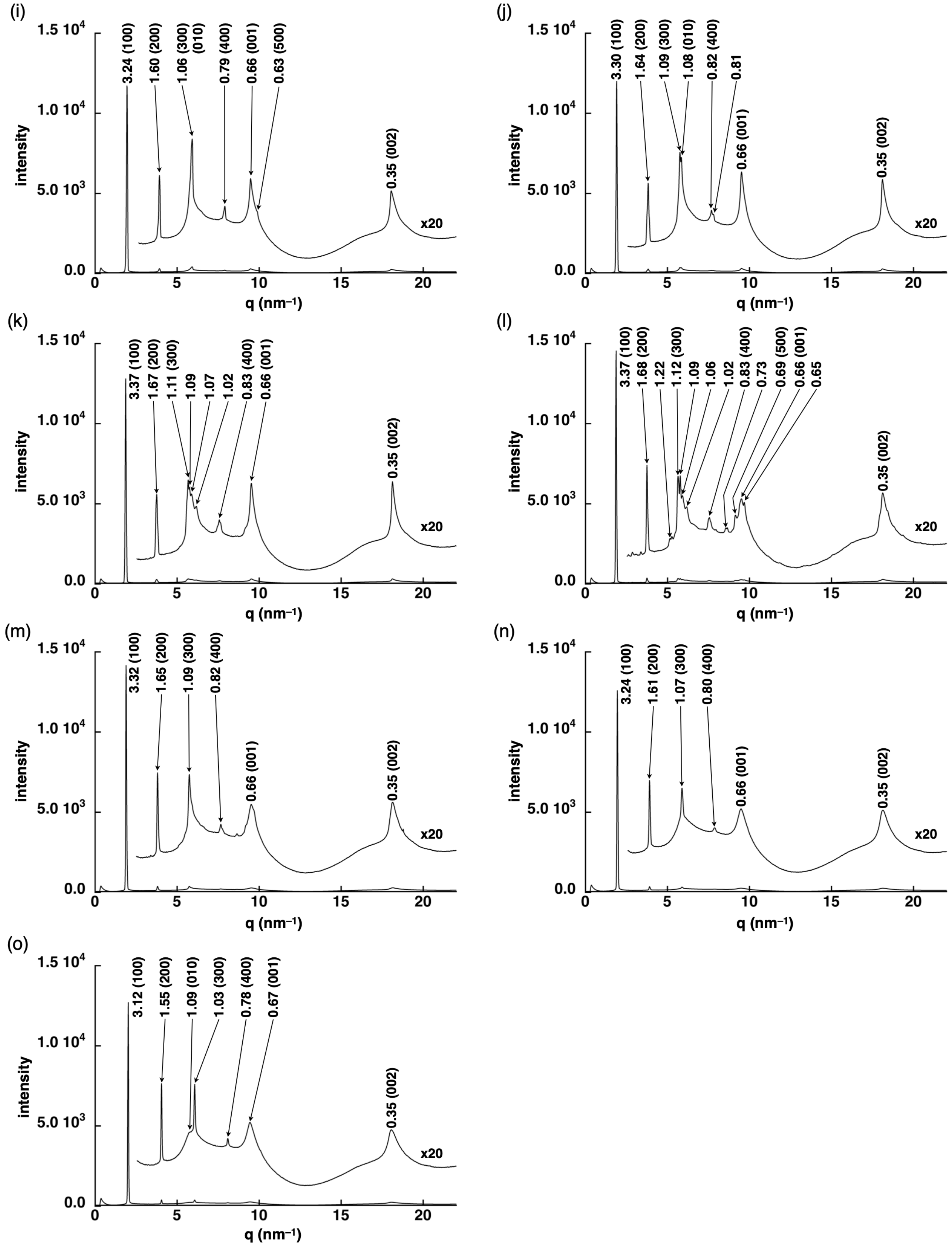

Figure S90 (Continued)

Table S9 Summary of XRD data of **1au**$^+$-PCCp$^-_{70\%}$. The peaks which can be indexed are represented.

	q (nm^{-1})	*d*-spacing (nm)	ratio	ratio (calc.)	*hkl*
(a) 25 °C (1st heating) Lam$_{col}$ a = 3.39 nm, c = 0.66 nm	1.85	3.39	1.000	1.0000	100
	3.72	1.69	0.498	0.5000	200
	5.60	1.12	0.331	0.3333	300
	7.47	0.84	0.248	0.2500	400
	9.33	0.67	0.199	0.2000	500
	9.48	0.66	–	–	001
	18.0	0.35	–	–	002
(b) 50 °C (1st heating) Lam$_{col}$ a = 3.37 nm, c = 0.66 nm	1.87	3.37	1.000	1.0000	100
	3.75	1.68	0.498	0.5000	200
	5.63	1.12	0.331	0.3333	300
	7.51	0.84	0.249	0.2500	400
	9.39	0.67	0.199	0.2000	500
	9.54	0.66	–	–	001
	17.9	0.35	–	–	002
(c) 60 °C (1st heating) Lam$_{col}$ a = 3.22 nm, c = 0.66 nm	1.95	3.22	1.000	1.0000	100
	3.90	1.61	0.501	0.5000	200
	5.85	1.07	0.334	0.3333	300
	7.61	0.83	0.257	0.2500	400
	9.50	0.66	–	–	001
	18.2	0.35	–	–	002
(d) 70 °C (1st heating) Lam$_{col}$ a = 3.16 nm, b = 1.08 nm, c = 0.67 nm	1.99	3.16	1.000	1.0000	100
	3.99	1.57	0.498	0.5000	200
	5.79	1.08	–	–	010
	6.01	1.04	0.331	0.3333	300
	8.02	0.78	0.248	0.2500	400
	9.44	0.67	–	–	001
	10.0	0.63	0.199	0.2000	500
	18.1	0.35	–	–	002
(e) 80 °C (1st heating) Lam$_{col}$ a = 3.14 nm, b = 1.10 nm, c = 0.67 nm	2.00	3.14	1.000	1.0000	100
	4.04	1.55	0.495	0.5000	200
	5.73	1.10	–	–	010
	6.06	1.04	0.330	0.3333	300
	8.06	0.78	0.249	0.2500	400
	9.42	0.67	–	–	001
	10.1	0.62	0.199	0.2000	500
	18.0	0.35	–	–	002
(f) 90 °C (1st heating) Lam$_{col}$ a = 3.12 nm, b = 1.10 nm, c = 0.67 nm	2.01	3.12	1.000	1.0000	100
	4.04	1.55	0.498	0.5000	200
	5.71	1.10	–	–	010
	6.09	1.03	0.331	0.3333	300
	8.10	0.78	0.249	0.2500	400
	9.39	0.67	–	–	001
	10.1	0.62	0.199	0.2000	500
	18.0	0.35	–	–	002
(g) 80 °C (1st cooling) Lam$_{col}$ a = 3.12 nm, b = 1.09 nm, c = 0.67 nm	2.01	3.12	1.000	1.0000	100
	4.04	1.55	0.498	0.5000	200
	5.74	1.09	–	–	010
	6.06	1.04	0.332	0.3333	300
	8.08	0.78	0.249	0.2500	400
	9.40	0.67	–	–	001
	10.1	0.62	0.200	0.2000	500
	18.0	0.35	–	–	002

Table S9 (Continued)

	q (nm^{-1})	*d*-spacing (nm)	ratio	ratio (calc.)	*hkl*
(h) 60 °C (1st cooling)<br>Lam$_{col}$<br>*a* = 3.18 nm, *b* = 1.08 nm, *c* = 0.67 nm	1.98	3.18	1.000	1.0000	100
	3.97	1.58	0.498	0.5000	200
	5.79	1.08	–	–	010
	5.98	1.05	0.331	0.3333	300
	7.98	0.79	0.248	0.2500	400
	9.44	0.67	–	–	001
	9.93	0.63	0.199	0.2000	500
	18.0	0.35	–	–	002
(i) 40 °C (1st cooling)<br>Lam$_{col}$<br>*a* = 3.24 nm, *b* = 1.06 nm, *c* = 0.66 nm	1.94	3.24	1.000	1.0000	100
	3.92	1.60	0.495	0.5000	200
	5.92	1.06	0.328	0.3333	300, 010
	7.91	0.79	0.245	0.2500	400
	9.48	0.66	–	–	001
	9.90	0.63	0.196	0.2000	500
	18.0	0.35	–	–	002
(j) 20 °C (1st cooling)<br>Lam$_{col}$<br>*a* = 3.30 nm, *b* = 1.08 nm, *c* = 0.66 nm	1.90	3.30	1.000	1.0000	100
	3.82	1.64	0.498	0.5000	200
	5.77	1.09	0.330	0.3333	300
	5.84	1.08	–	–	010
	7.68	0.82	0.248	0.2500	400
	9.51	0.66	–	–	001
	18.1	0.35	–	–	002
(k) 5 °C (1st cooling)<br>Lam$_{col}$<br>*a* = 3.37 nm, *c* = 0.66 nm	1.87	3.37	1.000	1.0000	100
	3.76	1.67	0.496	0.5000	200
	5.67	1.11	0.329	0.3333	300
	7.57	0.83	0.247	0.2500	400
	9.52	0.66	–	–	001
	18.1	0.35	–	–	002
(l) 20 °C (2nd heating)<br>Lam$_{col}$<br>*a* = 3.37 nm, *c* = 0.66 nm	1.87	3.37	1.000	1.0000	100
	3.75	1.68	0.498	0.5000	200
	5.63	1.12	0.331	0.3333	300
	7.56	0.83	0.247	0.2500	400
	9.14	0.69	0.204	0.2000	500
	9.50	0.66	–	–	001
	18.1	0.35	–	–	002
(m) 40 °C (2nd heating)<br>Lam$_{col}$<br>*a* = 3.32 nm, *c* = 0.66 nm	1.89	3.32	1.000	1.0000	100
	3.81	1.65	0.496	0.5000	200
	5.74	1.09	0.329	0.3333	300
	7.67	0.82	0.247	0.2500	400
	9.51	0.66	–	–	001
	18.1	0.35	–	–	002
(n) 60 °C (2nd heating)<br>Lam$_{col}$<br>*a* = 3.24 nm, *c* = 0.66 nm	1.94	3.24	1.000	1.0000	100
	3.91	1.61	0.497	0.5000	200
	5.89	1.07	0.329	0.3333	300
	7.85	0.80	0.247	0.2500	400
	9.49	0.66	–	–	001
	18.1	0.35	–	–	002
(o) 80 °C (2nd heating)<br>Lam$_{col}$<br>*a* = 3.12 nm, *b* = 1.09 nm, *c* = 0.67 nm	2.01	3.12	1.000	1.0000	100
	4.04	1.55	0.498	0.5000	200
	5.76	1.09	–	–	010
	6.08	1.03	0.332	0.3333	300
	8.08	0.78	0.249	0.2500	400
	9.44	0.67	–	–	001
	18.1	0.35	–	–	002

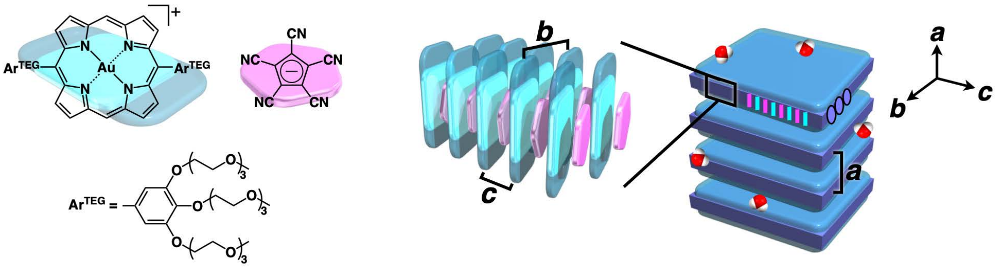

Figure S91 Possible packing model of **1au**$^{+}$-PCCp$^{-}{}_{70\%}$ as a Lam$_{col}$ structure.

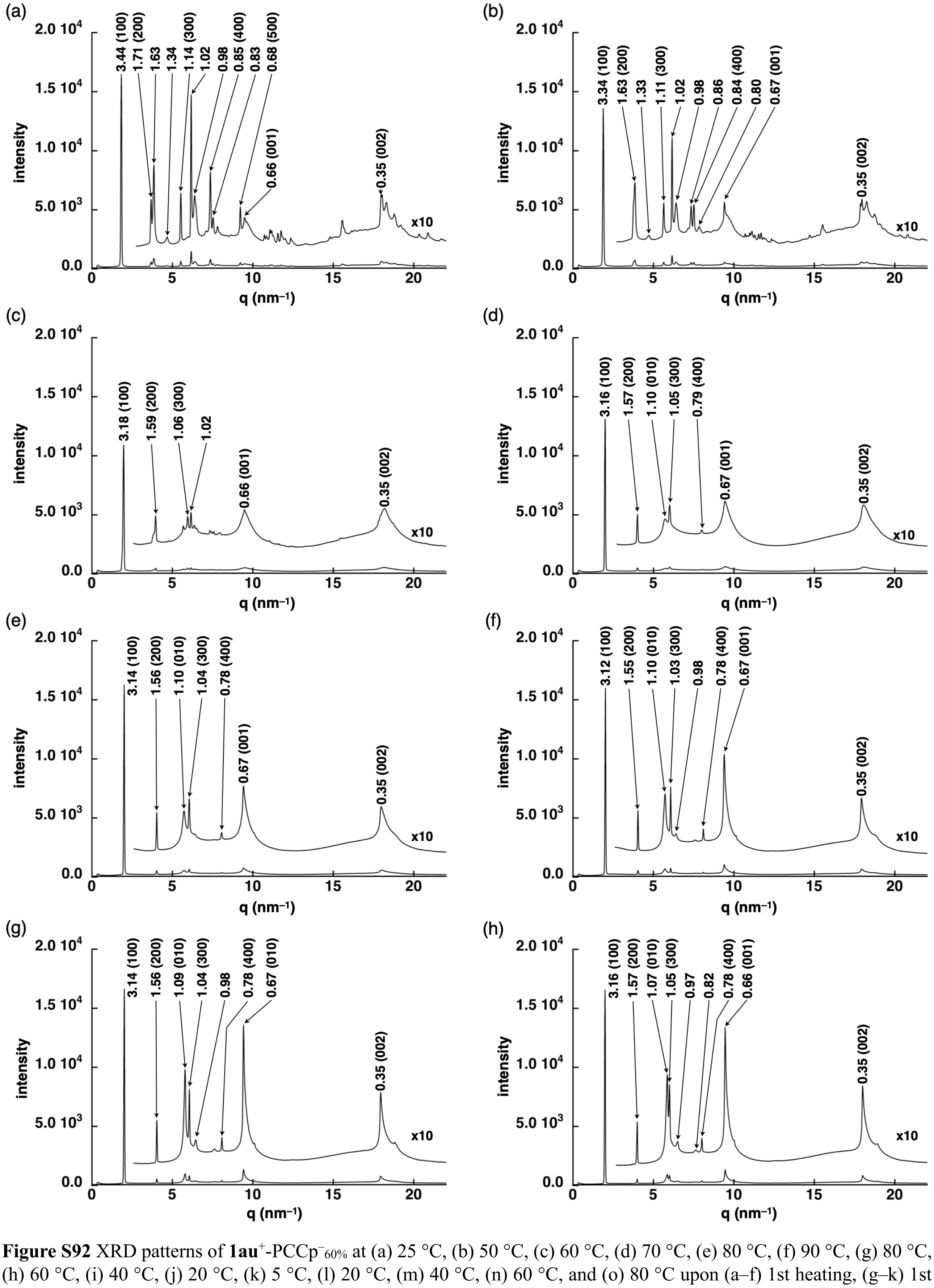

Figure S92 XRD patterns of **1au**$^{+}$-PCCp$^{-}_{60\%}$ at (a) 25 °C, (b) 50 °C, (c) 60 °C, (d) 70 °C, (e) 80 °C, (f) 90 °C, (g) 80 °C, (h) 60 °C, (i) 40 °C, (j) 20 °C, (k) 5 °C, (l) 20 °C, (m) 40 °C, (n) 60 °C, and (o) 80 °C upon (a–f) 1st heating, (g–k) 1st cooling, and (l–o) 2nd heating.

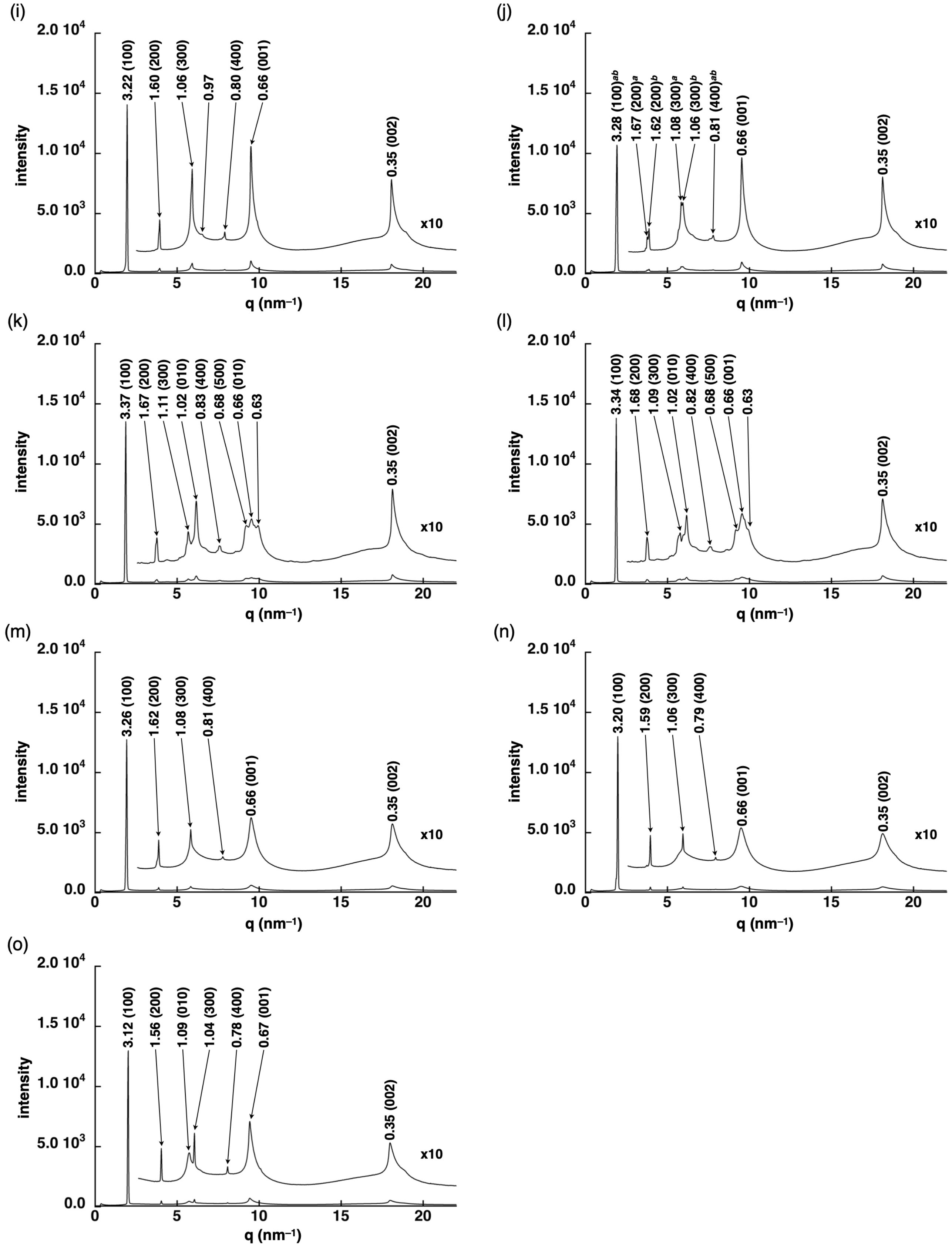

Figure S92 (Continued)

Table S10 Summary of XRD data of **1au**$^{+}$-PCCp$^{-}_{60\%}$. The peaks which can be indexed are represented.

	q (nm^{-1})	*d*-spacing (nm)	ratio	ratio (calc.)	*hkl*
	1.83	3.44	1.000	1.0000	100
	3.67	1.71	0.498	0.5000	200
(a) 25 °C (1st heating)	5.52	1.14	0.331	0.3333	300
Lam_{col}	7.36	0.85	0.248	0.2500	400
a = 3.44 nm, c = 0.66 nm	9.22	0.68	0.198	0.2000	500
	9.48	0.66	–	–	001
	18.0	0.35	–	–	002
	1.88	3.34	1.000	1.0000	100
	3.85	1.63	0.488	0.5000	200
(b) 50 °C (1st heating)	5.65	1.11	0.333	0.3333	300
Lam_{col}	7.52	0.84	0.250	0.2500	400
a = 3.34 nm, c = 0.67 nm	9.42	0.67	–	–	001
	17.9	0.35	–	–	002
	1.98	3.18	1.000	1.0000	100
(c) 60 °C (1st heating)	3.96	1.59	0.500	0.5000	200
Lam_{col}	5.95	1.06	0.332	0.3333	300
a = 3.18 nm, c = 0.66 nm	9.48	0.66	–	–	001
	18.2	0.35	–	–	002
	1.99	3.16	1.000	1.0000	100
	4.01	1.57	0.497	0.5000	200
(d) 70 °C (1st heating)	5.72	1.10	–	–	010
Lam_{col}	6.00	1.05	0.332	0.3333	300
a = 3.16 nm, b = 1.10 nm, c = 0.67 nm	8.00	0.79	0.249	0.2500	400
	9.44	0.67	–	–	001
	18.1	0.35	–	–	002
	2.00	3.14	1.000	1.0000	100
	4.03	1.56	0.497	0.5000	200
(e) 80 °C (1st heating)	5.72	1.10	–	–	010
Lam_{col}	6.05	1.04	0.331	0.3333	300
a = 3.14 nm, b = 1.10 nm, c = 0.67 nm	8.06	0.78	0.249	0.2500	400
	9.42	0.67	–	–	001
	18.0	0.35	–	–	002
	2.01	3.12	1.000	1.0000	100
	4.04	1.55	0.498	0.5000	200
(f) 90 °C (1st heating)	5.72	1.10	–	–	010
Lam_{col}	6.08	1.03	0.332	0.3333	300
a = 3.12 nm, b = 1.10 nm, c = 0.67 nm	8.10	0.78	0.249	0.2500	400
	9.40	0.67	–	–	001
	17.9	0.35	–	–	002
	2.00	3.14	1.000	1.0000	100
	4.03	1.56	0.497	0.5000	200
(g) 80 °C (1st cooling)	5.78	1.09	–	–	010
Lam_{col}	6.05	1.04	0.331	0.3333	300
a = 3.14 nm, b = 1.09 nm, c = 0.67 nm	8.08	0.78	0.248	0.2500	400
	9.42	0.67	–	–	001
	17.9	0.35	–	–	002
	1.99	3.16	1.000	1.0000	100
	3.99	1.57	0.498	0.5000	200
(h) 60 °C (1st cooling)	5.87	1.07	–	–	010
Lam_{col}	6.00	1.05	0.332	0.3333	300
a = 3.16 nm, b = 1.07 nm, c = 0.66 nm	8.01	0.78	0.249	0.2500	400
	9.45	0.66	–	–	001
	18.0	0.35	–	–	002

Table S10 (Continued)

	q (nm^{-1})	*d*-spacing (nm)	ratio	ratio (calc.)	*hkl*
(i) 40 °C (1st cooling)<br>Lam_{col}<br>*a* = 3.22 nm, *c* = 0.66 nm	1.95	3.22	1.000	1.0000	100
	3.93	1.60	0.497	0.5000	200
	5.92	1.06	0.330	0.3333	300
	7.90	0.80	0.247	0.2500	400
	9.50	0.66	–	–	001
	18.1	0.35	–	–	002
(j) 20 °C (1st cooling)<br>$^{a}Lam_{col}$<br>*a* = 3.28 nm, *c* = 0.66 nm<br>$^{b}Lam_{col}$<br>*a* = 3.28 nm, *c* = 0.66 nm	1.92[ab]	3.28	1.000	1.0000	100
	3.77[a]	1.67	0.508	0.5000	200
	3.88[b]	1.62	0.493	0.5000	200
	5.84[a]	1.08	0.328	0.3333	300
	5.94[b]	1.06	0.322	0.3333	300
	7.79[ab]	0.81	0.246	0.2500	400
	9.52	0.66	–	–	001
	18.1	0.35	–	–	002
(k) 5 °C (1st cooling)<br>Lam_{col}<br>*a* = 3.37 nm, *b* = 1.02 nm, *c* = 0.66 nm	1.87	3.37	1.000	1.0000	100
	3.77	1.67	0.495	0.5000	200
	5.67	1.11	0.329	0.3333	300
	6.16	1.02	–	–	010
	7.58	0.83	0.246	0.2500	400
	9.22	0.68	0.202	0.2000	500
	9.54	0.66	–	–	001
	18.1	0.35	–	–	002
(l) 20 °C (2nd heating)<br>Lam_{col}<br>*a* = 3.34 nm, *b* = 1.02 nm, *c* = 0.66 nm	1.88	3.34	1.000	1.0000	100
	3.75	1.68	0.501	0.5000	200
	5.77	1.09	0.326	0.3333	300
	6.16	1.02	–	–	010
	7.62	0.82	0.247	0.2500	400
	9.17	0.68	0.205	0.2000	500
	9.55	0.66	–	–	001
	18.1	0.35	–	–	002
(m) 40 °C (2nd heating)<br>Lam_{col}<br>*a* = 3.26 nm, *c* = 0.66 nm	1.93	3.26	1.000	1.0000	100
	3.87	1.62	0.498	0.5000	200
	5.83	1.08	0.331	0.3333	300
	7.79	0.81	0.248	0.2500	400
	9.50	0.66	–	–	001
	18.1	0.35	–	–	002
(n) 60 °C (2nd heating)<br>Lam_{col}<br>*a* = 3.20 nm, *c* = 0.66 nm	1.97	3.20	1.000	1.0000	100
	3.96	1.59	0.497	0.5000	200
	5.94	1.06	0.331	0.3333	300
	7.93	0.79	0.248	0.2500	400
	9.49	0.66	–	–	001
	18.1	0.35	–	–	002
(o) 80 °C (2nd heating)<br>Lam_{col}<br>*a* = 3.12 nm, *b* = 1.09 nm, *c* = 0.67 nm	2.01	3.12	1.000	1.0000	100
	4.03	1.56	0.500	0.5000	200
	5.74	1.09	–	–	010
	6.05	1.04	0.333	0.3333	300
	8.07	0.78	0.250	0.2500	400
	9.43	0.67	–	–	001
	18.0	0.35	–	–	002

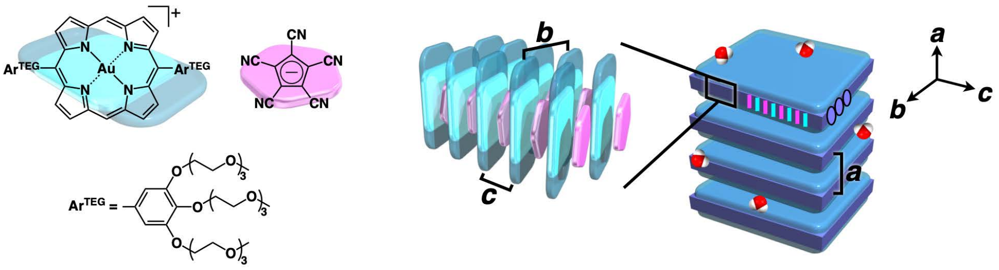

Figure S93 Possible packing model of **1au**$^{+}$-PCCp^{-}$_{60\%}$ as a Lam$_{col}$ structure.

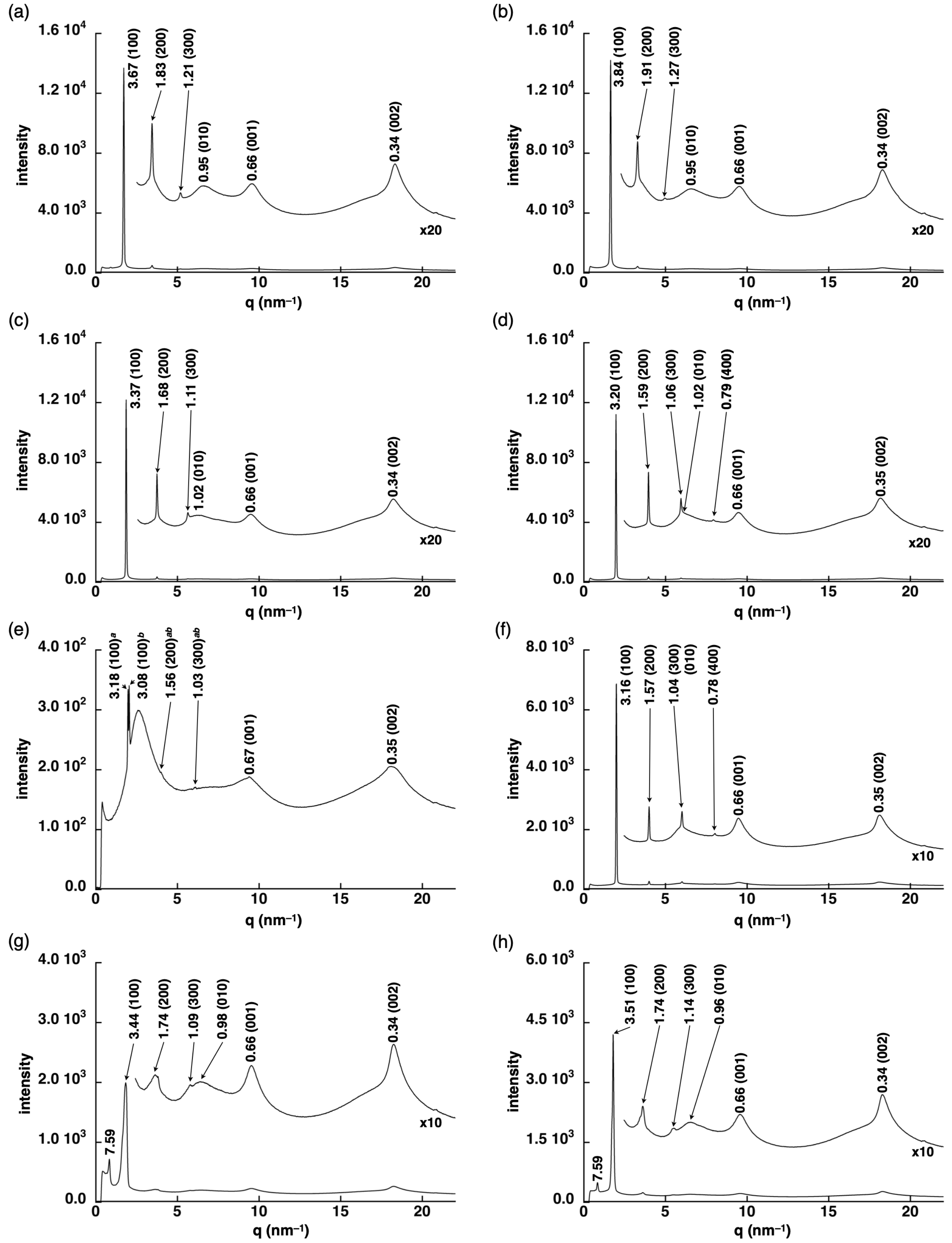

Figure S94 XRD patterns of **1au**$^+$-PCCp$^-{}_{50\%}$ at (a) 25 °C, (b) 40 °C, (c) 60 °C, (d) 70 °C, (e) 90 °C, (f) 70 °C, (g) 40 °C, (h) 20 °C, (i) 5 °C, (j) 20 °C, (k) 40 °C, (l) 60 °C, (m) 70 °C, and (n) 90 °C upon (a–e) 1st heating, (f–i) 1st cooling, and (j–n) 2nd heating.

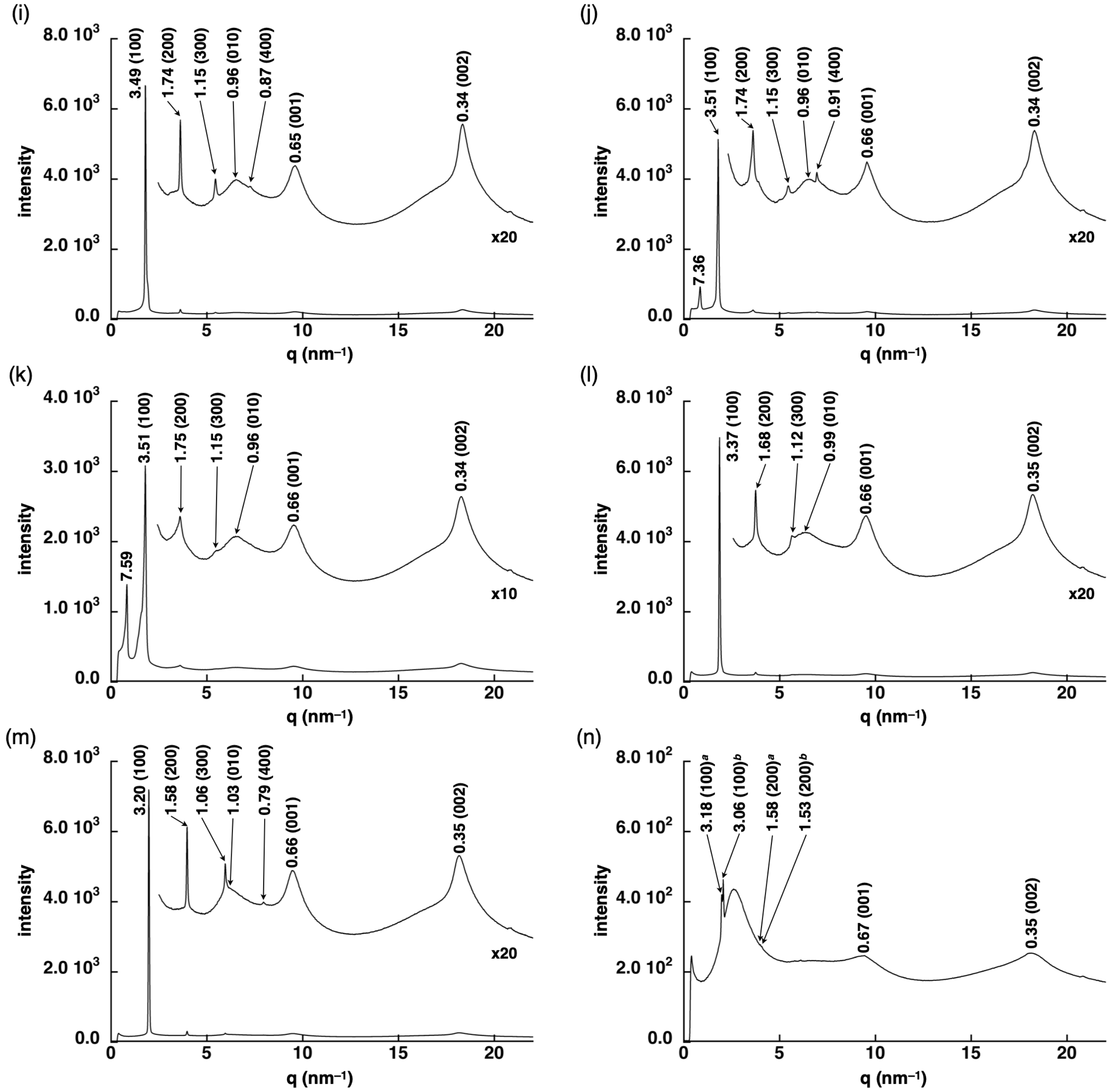

Figure S94 (Continued)

Table S11 Summary of XRD data of **1au**$^+$-PCCp$^-_{50\%}$. The peaks which can be indexed are represented.

	q (nm^{-1})	*d*-spacing (nm)	ratio	ratio (calc.)	*hkl*
(a) 25 °C (1st heating)<br>Lam$_{col}$<br>a = 3.67 nm, b = 0.95 nm, c = 0.66 nm	1.71	3.67	1.000	1.0000	100
	3.44	1.83	0.498	0.5000	200
	5.19	1.21	0.330	0.3333	300
	6.63	0.95	–	–	010
	9.59	0.66	–	–	001
	18.3	0.34	–	–	002
(b) 40 °C (1st heating)<br>Lam$_{col}$<br>a = 3.84 nm, b = 0.95 nm, c = 0.66 nm	1.64	3.84	1.000	1.0000	100
	3.29	1.91	0.498	0.5000	200
	4.96	1.27	0.330	0.3333	300
	6.60	0.95	–	–	010
	9.52	0.66	–	–	001
	18.3	0.34	–	–	002
(c) 60 °C (1st heating)<br>Lam$_{col}$´<br>a = 3.37 nm, b = 1.02 nm, c = 0.66 nm	1.86	3.37	1.000	1.0000	100
	3.74	1.68	0.498	0.5000	200
	5.64	1.11	0.331	0.3333	300
	6.18	1.02	–	–	010
	9.49	0.66	–	–	001
	18.2	0.34	–	–	002
(d) 70 °C (1st heating)<br>Lam$_{col}$´<br>a = 3.20 nm, b = 1.02 nm, c = 0.66 nm	1.96	3.20	1.000	1.0000	100
	3.96	1.59	0.497	0.5000	200
	5.94	1.06	0.331	0.3333	300
	6.14	1.02	–	–	010
	7.93	0.79	0.248	0.2500	400
	9.49	0.66	–	–	001
	18.1	0.35	–	–	002
(e) 90 °C (1st heating)<br>[a]Lam$_{col}$´<br>a = 3.18 nm, c = 0.67 nm<br>[b]Lam$_{col}$´<br>a = 3.08 nm, c = 0.67 nm	1.98[a]	3.18	1.000	1.0000	100
	2.04[a]	3.08	1.000	1.0000	100
	4.02[ab]	1.56	0.492a, 0.508b	0.5000	200
	6.10[ab]	1.03	0.324a, 0.334b	0.3333	300
	9.39	0.67	–	–	001
	18.1	0.35	–	–	002
(f) 70 °C (1st cooling)<br>Lam$_{col}$<br>a = 3.16 nm, b = 1.04 nm, c = 0.66 nm	1.99	3.16	1.000	1.0000	100
	3.99	1.57	0.498	0.5000	200
	6.01	1.04	0.331	0.3333	300, 010
	8.03	0.78	0.248	0.2500	400
	9.46	0.66	–	–	001
	18.1	0.35	–	–	002
(g) 40 °C (1st cooling)<br>Lam$_{col}$<br>a = 3.44 nm, b = 0.98 nm, c = 0.66 nm	1.83	3.44	1.000	1.0000	100
	3.62	1.74	0.505	0.5000	200
	5.79	1.09	0.315	0.3333	300
	6.44	0.98	–	–	010
	9.54	0.66	–	–	001
	18.3	0.34	–	–	002
(h) 20 °C (1st cooling)<br>Lam$_{col}$<br>a = 3.51 nm, b = 0.96 nm, c = 0.66 nm	1.79	3.51	1.000	1.0000	100
	3.62	1.74	0.494	0.5000	200
	5.51	1.14	0.324	0.3333	300
	6.56	0.96	–	–	010
	9.57	0.66	–	–	001
	18.3	0.34	–	–	002

Table S11 (Continued)

	q (nm^{-1})	*d*-spacing (nm)	ratio	ratio (calc.)	*hkl*
(i) 5 °C (1st cooling) Lam$_{col}$ *a* = 3.49 nm, *b* = 0.96 nm, *c* = 0.65 nm	1.80	3.49	1.000	1.0000	100
	3.62	1.74	0.498	0.5000	200
	5.45	1.15	0.330	0.3333	300
	6.53	0.96	–	–	010
	7.24	0.87	0.249	0.2500	400
	9.60	0.65	–	–	001
	18.3	0.34	–	–	002
(j) 20 °C (2nd heating) Lam$_{col}$ *a* = 3.51 nm, *b* = 0.96 nm, *c* = 0.66 nm	1.79	3.51	1.000	1.0000	100
	3.62	1.74	0.494	0.5000	200
	5.45	1.15	0.328	0.3333	300
	6.56	0.96	–	–	010
	6.94	0.91	0.258	0.2500	400
	9.55	0.66	–	–	001
	18.3	0.34	–	–	002
(k) 40 °C (2nd heating) Lam$_{col}$ *a* = 3.51 nm, *b* = 0.96 nm, *c* = 0.66 nm	1.79	3.51	1.000	1.0000	100
	3.59	1.75	0.498	0.5000	200
	5.48	1.15	0.326	0.3333	300
	6.55	0.96	–	–	010
	9.53	0.66	–	–	001
	18.3	0.34	–	–	002
(l) 60 °C (2nd heating) Lam$_{col}$ *a* = 3.37 nm, *b* = 0.99 nm, *c* = 0.66 nm	1.86	3.37	1.000	1.0000	100
	3.74	1.68	0.498	0.5000	200
	5.63	1.12	0.331	0.3333	300
	6.35	0.99	–	–	010
	9.51	0.66	–	–	001
	18.2	0.35	–	–	002
(m) 70 °C (2nd heating) Lam$_{col}$ *a* = 3.20 nm, *b* = 1.03 nm, *c* = 0.66 nm	1.96	3.20	1.000	1.0000	100
	3.97	1.58	0.495	0.5000	200
	5.95	1.06	0.330	0.3333	300
	6.09	1.03	–	–	010
	7.95	0.79	0.247	0.2500	400
	9.48	0.66	–	–	001
	18.2	0.35	–	–	002
(n) 90 °C (2nd heating) ^{a}Lam$_{col}$ *a* = 3.18 nm, *c* = 0.67 nm ^{b}Lam$_{col}$ *a* = 3.06 nm, *c* = 0.67 nm	1.98^{a}	3.18	1.000	1.0000	100
	2.05^{b}	3.06	1.000	1.0000	100
	3.98^{a}	1.58	0.497	0.5000	200
	4.11^{b}	1.53	0.500	0.5000	200
	9.43	0.67	–	–	001
	18.1	0.35	–	–	002

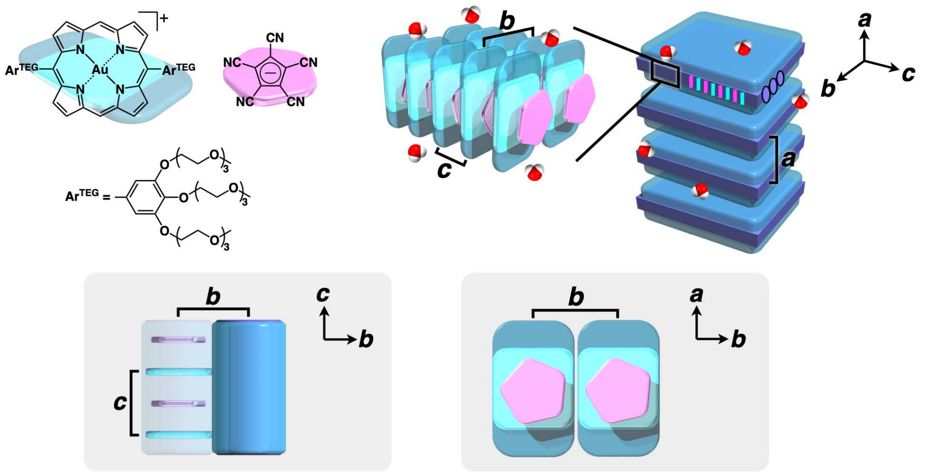

Figure S95 Possible packing model of **1au**$^{+}$-PCCp^{-}$_{50\%}$ as a Lam$_{col}$ structure.

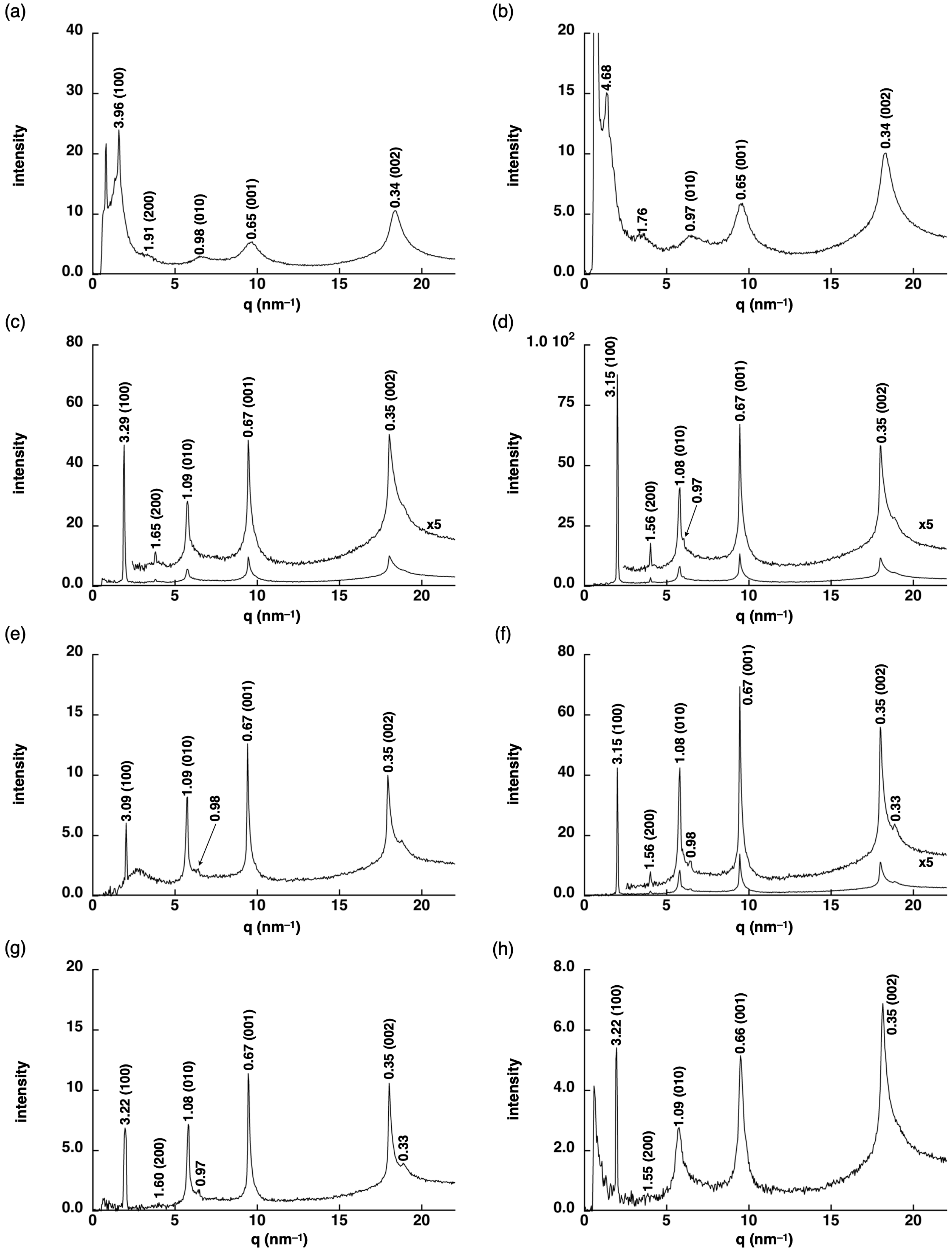

Figure S96 XRD patterns of **1au**$^{+}$-PCCp^{-}$_{40\%}$ at (a) 25 °C, (b) 40 °C, (c) 60 °C, (d) 70 °C, (e) 90 °C, (f) 70 °C, (g) 60 °C, (h) 40 °C, (i) 20 °C, (j) 5 °C, (k) 20 °C, (l) 40 °C, (m) 60 °C, (n) 70 °C, and (o) 90 °C upon (a–e) 1st heating, (f–j) 1st cooling, and (k–o) 2nd heating.

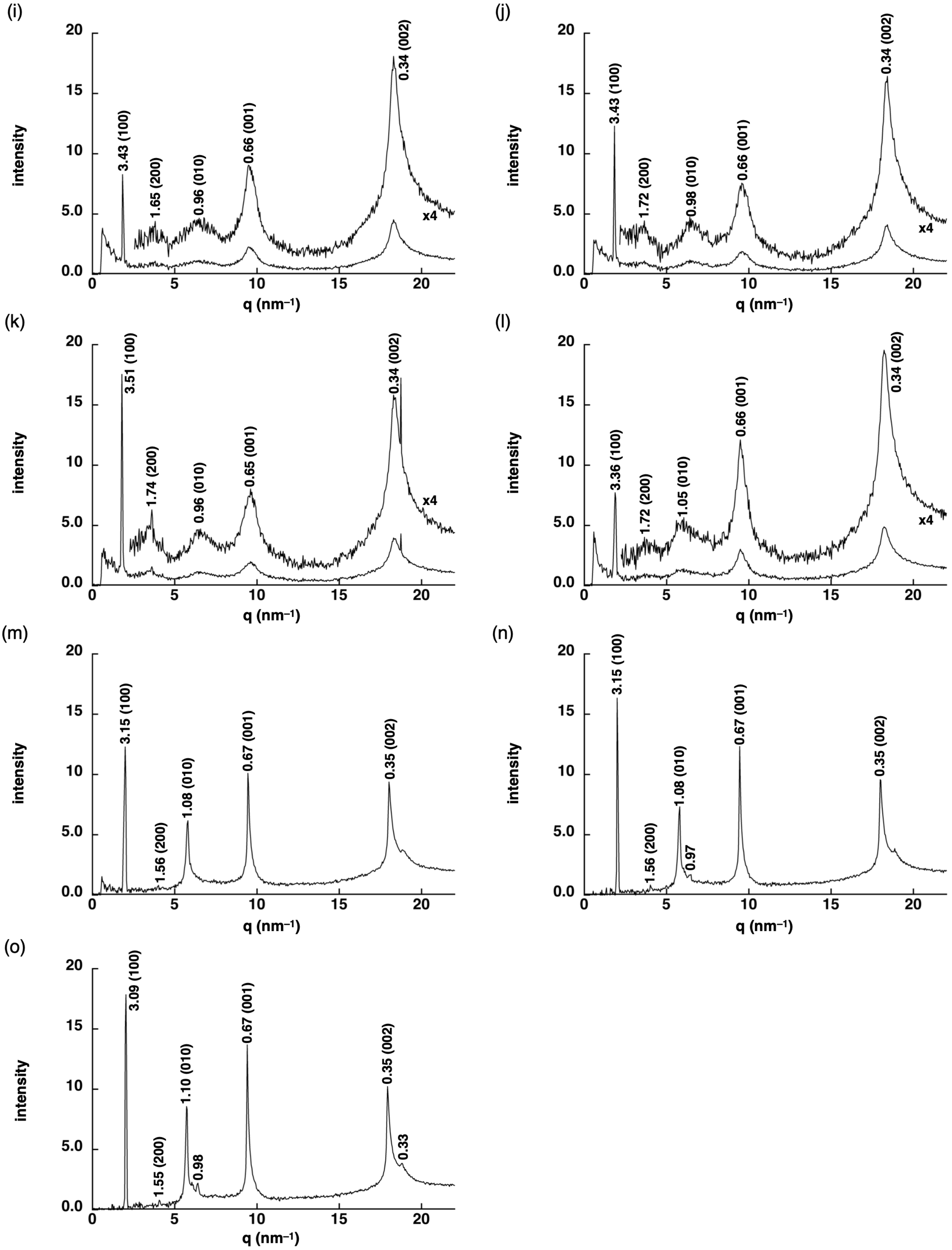

Figure S96 (Continued)

Table S12 Summary of XRD data of **1au**$^+$-PCCp$^-$$_{40\%}$. The peaks which can be indexed are represented.

	q (nm^{-1})	*d*-spacing (nm)	ratio	ratio (calc.)	*hkl*
	1.59	3.96	1.000	1.0000	100
(a) 25 °C (1st heating)	3.29	1.91	0.483	0.5000	200
Lam$_{col}$	6.41	0.98	–	–	010
a = 3.96 nm, *b* = 0.98 nm, *c* = 0.65 nm	9.68	0.65	–	–	001
	18.4	0.34	–	–	002
	1.91	3.29	1.000	1.0000	100
(c) 60 °C (1st heating)	3.81	1.65	0.501	0.5000	200
Lam$_{col}$´	5.76	1.09	–	–	010
a = 3.29 nm, *b* = 1.09 nm, *c* = 0.67 nm	9.44	0.67	–	–	001
	18.0	0.35	–	–	002
	1.99	3.15	1.000	1.0000	100
(d) 70 °C (1st heating)	4.02	1.56	0.496	0.5000	200
Lam$_{col}$´	5.80	1.08	–	–	010
a = 3.15 nm, *b* = 1.08 nm, *c* = 0.67 nm	9.44	0.67	–	–	001
	18.0	0.35	–	–	002
(e) 90 °C (1st heating)	2.03	3.09	1.000	1.0000	100
Lam$_{col}$´	5.76	1.09	–	–	010
a = 3.09 nm, *b* = 1.09 nm, *c* = 0.67 nm	9.40	0.67	–	–	001
	17.9	0.35	–	–	002
	1.99	3.15	1.000	1.0000	100
(f) 70 °C (1st cooling)	4.02	1.56	0.496	0.5000	200
Lam$_{col}$	5.80	1.08	–	–	010
a = 3.15 nm, *b* = 1.08 nm, *c* = 0.67 nm	9.44	0.67	–	–	001
	18.0	0.35	–	–	002
	1.95	3.22	1.000	1.0000	100
(g) 60 °C (1st cooling)	3.94	1.60	0.496	0.5000	200
Lam$_{col}$	5.80	1.08	–	–	010
a = 3.22 nm, *b* = 1.08 nm, *c* = 0.67 nm	9.44	0.67	–	–	001
	18.0	0.35	–	–	002
	1.95	3.22	1.000	1.0000	100
(h) 40 °C (1st cooling)	4.06	1.55	0.481	0.5000	200
Lam$_{col}$	5.76	1.09	–	–	010
a = 3.22 nm, *b* = 1.09 nm, *c* = 0.66 nm	9.48	0.66	–	–	001
	18.1	0.35	–	–	002
	1.83	3.43	1.000	1.0000	100
(i) 20 °C (1st cooling)	3.81	1.65	0.480	0.5000	200
Lam$_{col}$	6.53	0.96	–	–	010
a = 3.43 nm, *b* = 0.96 nm, *c* = 0.66 nm	9.48	0.66	–	–	001
	18.3	0.34	–	–	002
	1.83	3.43	1.000	1.0000	100
(j) 5 °C (1st cooling)	3.65	1.72	0.501	0.5000	200
Lam$_{col}$	6.41	0.98	–	–	010
a = 3.43 nm, *b* = 0.98 nm, *c* = 0.66 nm	9.56	0.66	–	–	001
	18.4	0.34	–	–	002
	1.79	3.51	1.000	1.0000	100
(k) 20 °C (2nd heating)	3.61	1.74	0.495	0.5000	200
Lam$_{col}$	6.57	0.96	–	–	010
a = 3.51 nm, *b* = 0.96 nm, *c* = 0.65 nm	9.60	0.65	–	–	001
	18.3	0.34	–	–	002
	1.87	3.36	1.000	1.0000	100
(l) 40 °C (2nd heating)	3.65	1.72	0.512	0.5000	200
Lam$_{col}$	5.96	1.05	–	–	010
a = 3.36 nm, *b* = 1.05 nm, *c* = 0.66 nm	9.48	0.66	–	–	001
	18.2	0.34	–	–	002

Table S12 (Continued)

	q (nm^{-1})	*d*-spacing (nm)	ratio	ratio (calc.)	*hkl*
	1.99	3.15	1.000	1.0000	100
(m) 60 °C (2nd heating)	4.02	1.56	0.496	0.5000	200
Lam$_{col}$	5.80	1.08	–	–	010
a = 3.15 nm, b = 1.08 nm, c = 0.67 nm	9.44	0.67	–	–	001
	18.0	0.35	–	–	002
	1.99	3.15	1.000	1.0000	100
(n) 70 °C (2nd heating)	4.02	1.56	0.496	0.5000	200
Lam$_{col}$	5.80	1.08	–	–	010
a = 3.15 nm, b = 1.08 nm, c = 0.67 nm	9.44	0.67	–	–	001
	18.0	0.35	–	–	002
	2.03	3.09	1.000	1.0000	100
(o) 90 °C (2nd heating)	4.06	1.55	0.501	0.5000	200
Lam$_{col}$	5.72	1.10	–	–	010
a = 3.09 nm, b = 1.10 nm, c = 0.67 nm	9.40	0.67	–	–	001
	17.9	0.35	–	–	002

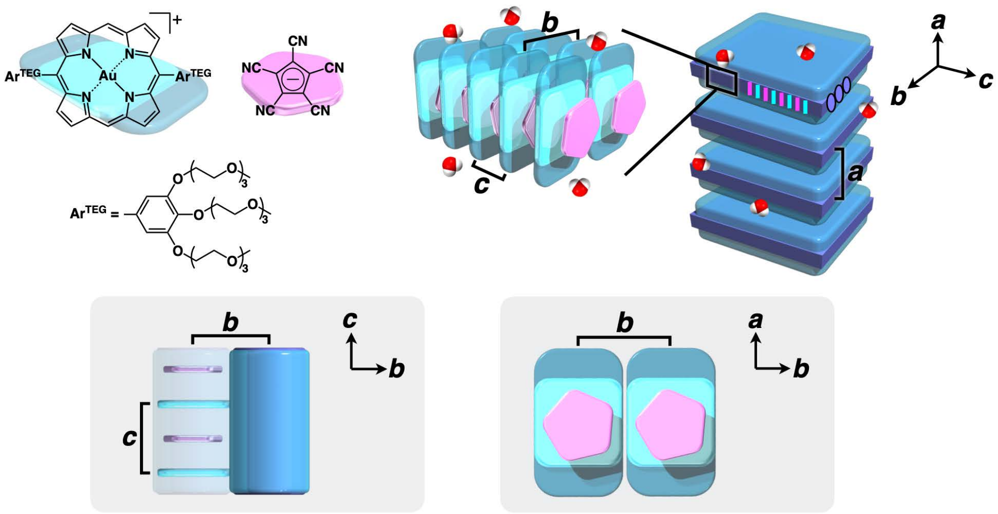

Figure S97 Possible packing model of **1au**$^{+}$-PCCp$^{-}_{40\%}$ as a Lam$_{col}$ structure.

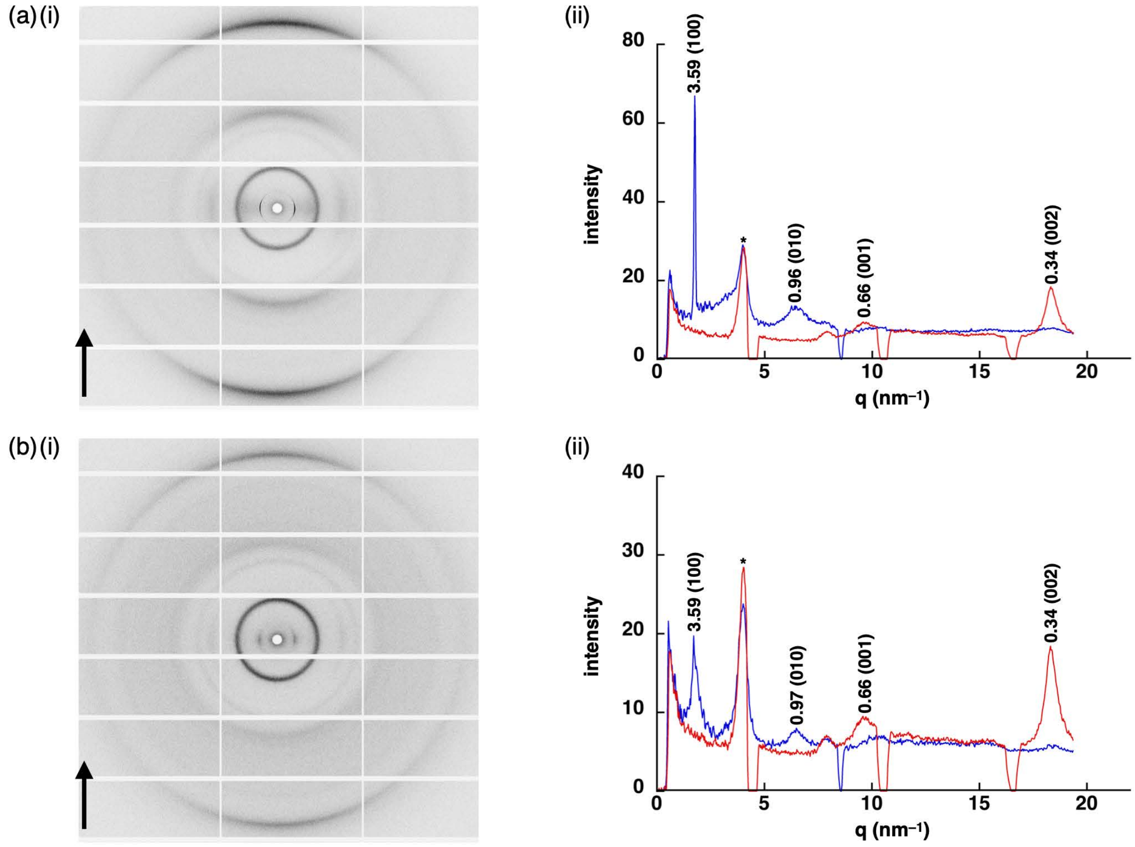

Figure S98 XRD patterns of (a) **1au**$^+$-PCCp$^-_{50\%}$ and (b) **1au**$^+$-PCCp$^-_{40\%}$ sheared between Kapton (polyimide) film at r.t.: (i) 2D XRD diffraction patterns with an arrow indicating shearing (meridional) direction and (ii) corresponding 1D patterns of shearing direction (90° ± 10°, red) and equatorial direction (0° ± 10°, blue). Diffractions of asterisk indicate those from the polyimide film. The grid-like artifacts from the detector provided no severe problems in the examinations of shearing-induced alignment.

Table S13 Summary of XRD data of (a) **1au**$^+$-PCCp$^-_{50\%}$ and (b) **1au**$^+$-PCCp$^-_{40\%}$. The peaks which can be indexed are represented.

	q (nm^{-1})	*d*-spacing (nm)	ratio	ratio (calc.)	*hkl*
	1.75	3.59	1.000	1.0000	100
(a) 25 °C (heating)	3.41	1.84	0.513	0.5000	200
$\mathrm{Lam_{col}}$	6.53	0.96	–	–	010
a = 3.59 nm, b = 0.96 nm, c = 0.66 nm	9.48	0.66	–	–	001
	18.3	0.34	–	–	002
	1.75	3.59	1.000	1.0000	100
(b) 25 °C (heating)	3.37	1.86	0.519	0.5000	200
$\mathrm{Lam_{col}}$	6.49	0.97	–	–	010
a = 3.59 nm, b = 0.97 nm, c = 0.66 nm	9.56	0.66	–	–	001
	18.3	0.34	–	–	002

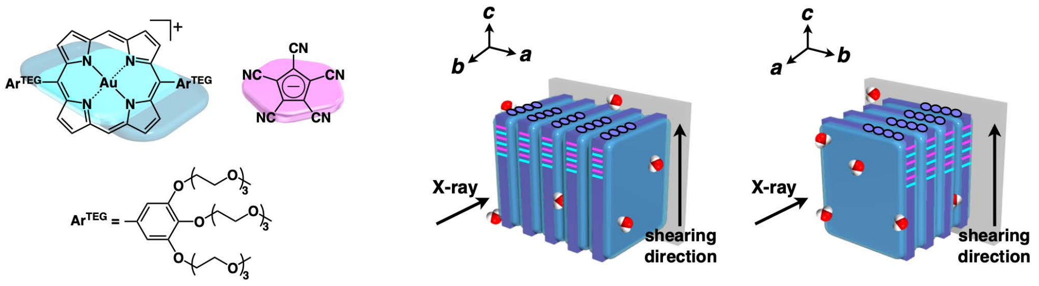

Figure S99 Possible shearing-induced alignment models of **1au**$^+$-PCCp$^-_{50\%/40\%}$ as $\mathrm{Lam_{col}}$ structures.

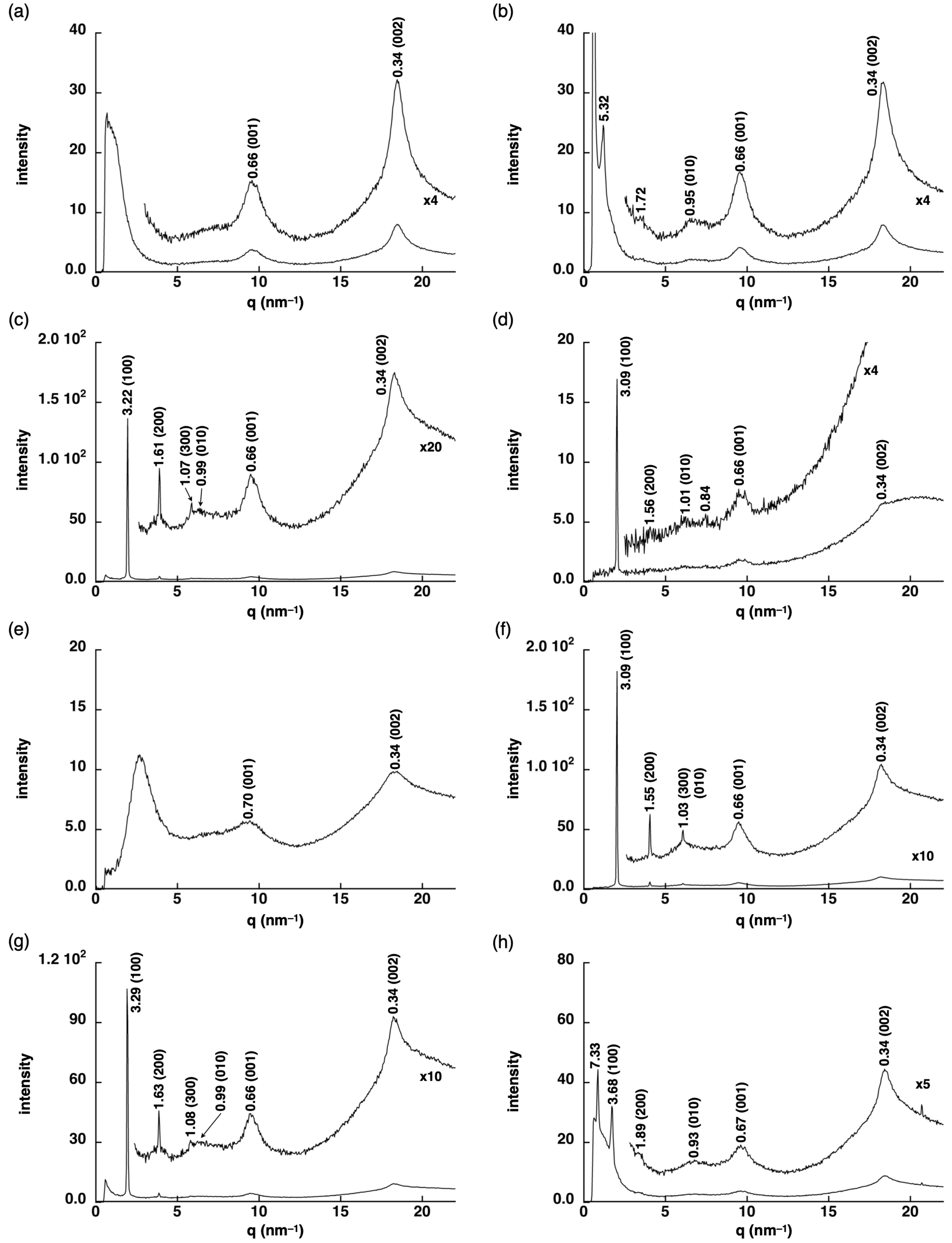

Figure S100 XRD patterns of **1au**$^{+}$-PCCp$^{-}_{30\%}$ at (a) 25 °C, (b) 40 °C, (c) 60 °C, (d) 70 °C, (e) 90 °C, (f) 70 °C, (g) 60 °C, (h) 25 °C, (i) 20 °C, (j) 5 °C, (k) 20 °C, (l) 40 °C, (m) 60 °C, (n) 70 °C, and (o) 90 °C upon (a–e) 1st heating, (f–j) 1st cooling, and (k–o) 2nd heating.

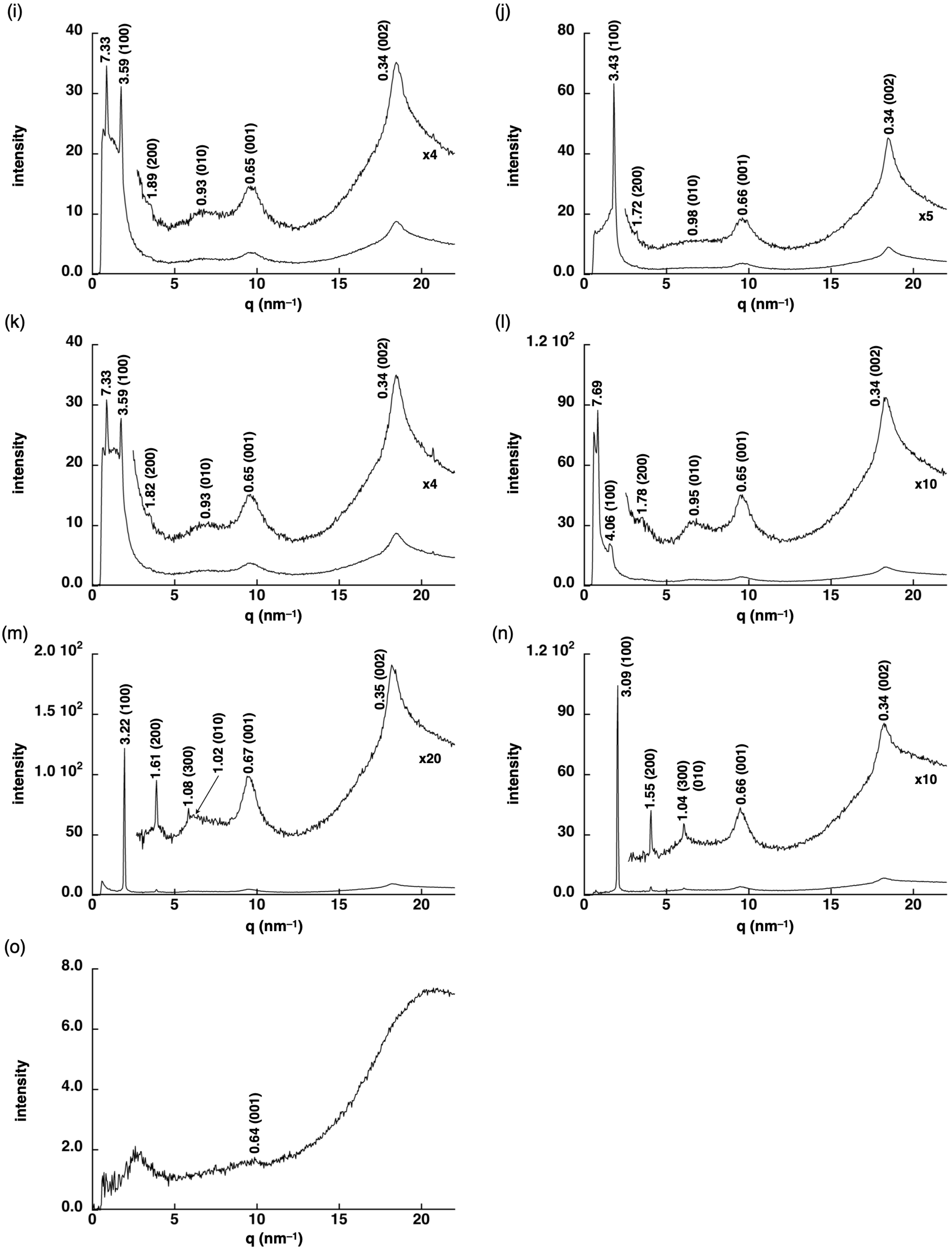

Figure S100 (Continued)

Table S14 Summary of XRD data of **1au**$^+$-PCCp$^-$$_{30\%}$. The peaks which can be indexed are represented.

	q (nm^{-1})	*d*-spacing (nm)	ratio	ratio (calc.)	*hkl*
(c) 60 °C (1st heating) Lam$_{col}$′ *a* = 3.22 nm, *b* = 0.99 nm, *c* = 0.66 nm	1.95	3.22	1.000	1.0000	100
	3.90	1.61	0.501	0.5000	200
	5.88	1.07	0.332	0.3333	300
	6.32	0.99	–	–	010
	9.48	0.66	–	–	001
	18.3	0.34	–	–	002
(d) 70 °C (1st heating) Lam$_{col}$′ *a* = 3.09 nm, *b* = 1.01 nm, *c* = 0.66 nm	2.03	3.09	1.000	1.0000	100
	4.02	1.56	0.506	0.5000	200
	6.24	1.01	–	–	010
	9.48	0.66	–	–	001
	18.5	0.34	–	–	002
(f) 70 °C (1st cooling) Lam$_{col}$ *a* = 3.09 nm, *b* = 1.03 nm, *c* = 0.66 nm	2.03	3.09	1.000	1.0000	100
	4.06	1.55	0.501	0.5000	200
	6.08	1.03	0.334	0.3333	300, 010
	9.48	0.66			001
	18.2	0.34	–	–	002
(g) 60 °C (1st cooling) Lam$_{col}$ *a* = 3.29 nm, *b* = 0.99 nm, *c* = 0.66 nm	1.91	3.29	1.000	1.0000	100
	3.85	1.63	0.496	0.5000	200
	5.80	1.08	0.329	0.3333	300
	6.37	0.99	–	–	010
	9.48	0.66	–	–	001
	18.2	0.34	–	–	002
(h) 25 °C (1st cooling) Lam$_{col}$ *a* = 3.68 nm, *b* = 0.93 nm, *c* = 0.67 nm	1.71	3.68	1.000	1.0000	100
	3.33	1.89	0.513	0.5000	200
	6.77	0.93	–	–	010
	9.44	0.67	–	–	001
	18.4	0.34	–	–	002
(i) 20 °C (1st cooling) Lam$_{col}$ *a* = 3.59 nm, *b* = 0.93 nm, *c* = 0.65 nm	1.75	3.59	1.000	1.0000	100
	3.33	1.89	0.525	0.5000	200
	6.77	0.93	–	–	010
	9.60	0.65	–	–	001
	18.5	0.34	–	–	002
(j) 5 °C (1st cooling) Lam$_{col}$ *a* = 3.43 nm, *b* = 0.98 nm, *c* = 0.66 nm	1.83	3.43	1.000	1.0000	100
	3.65	1.72	0.501	0.5000	200
	6.41	0.98	–	–	010
	9.56	0.66	–	–	001
	18.4	0.34	–	–	002
(k) 20 °C (2nd heating) Lam$_{col}$ *a* = 3.59 nm, *b* = 0.93 nm, *c* = 0.65 nm	1.75	3.59	1.000	1.0000	100
	3.45	1.82	0.507	0.5000	200
	6.77	0.93	–	–	010
	9.60	0.65	–	–	001
	18.5	0.34	–	–	002
(l) 40 °C (2nd heating) Lam$_{col}$ *a* = 4.06 nm, *b* = 0.95 nm, *c* = 0.65 nm	1.55	4.06	1.000	1.0000	100
	3.53	1.78	0.438	0.5000	200
	6.61	0.95	–	–	010
	9.60	0.65	–	–	001
	18.2	0.34	–	–	002
(m) 60 °C (2nd heating) Lam$_{col}$ *a* = 3.22 nm, *b* = 1.02 nm, *c* = 0.67 nm	1.95	3.22	1.000	1.0000	100
	3.90	1.61	0.501	0.5000	200
	5.84	1.08	0.334	0.3333	300
	6.16	1.02	–	–	010
	9.40	0.67	–	–	001
	18.2	0.35	–	–	002

Table S14 (Continued)

	q (nm^{-1})	*d*-spacing (nm)	ratio	ratio (calc.)	*hkl*
	2.03	3.09	1.000	1.0000	100
(n) 70 °C (2nd heating)	4.06	1.55	0.501	0.5000	200
Lam_{col}	6.04	1.04	0.336	0.3333	300, 010
a = 3.09 nm, b = 1.04 nm, c = 0.66 nm	9.48	0.66	–	–	001
	18.2	0.34	–	–	002

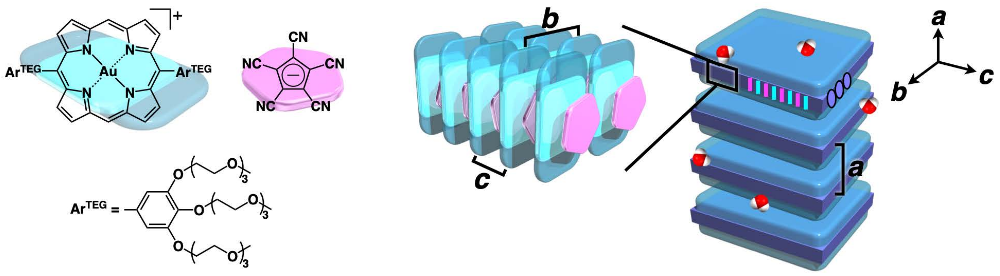

Figure S101 Possible packing model of **1au**$^+$-PCCp$^-_{30\%}$ as a Lam_{col} structure.

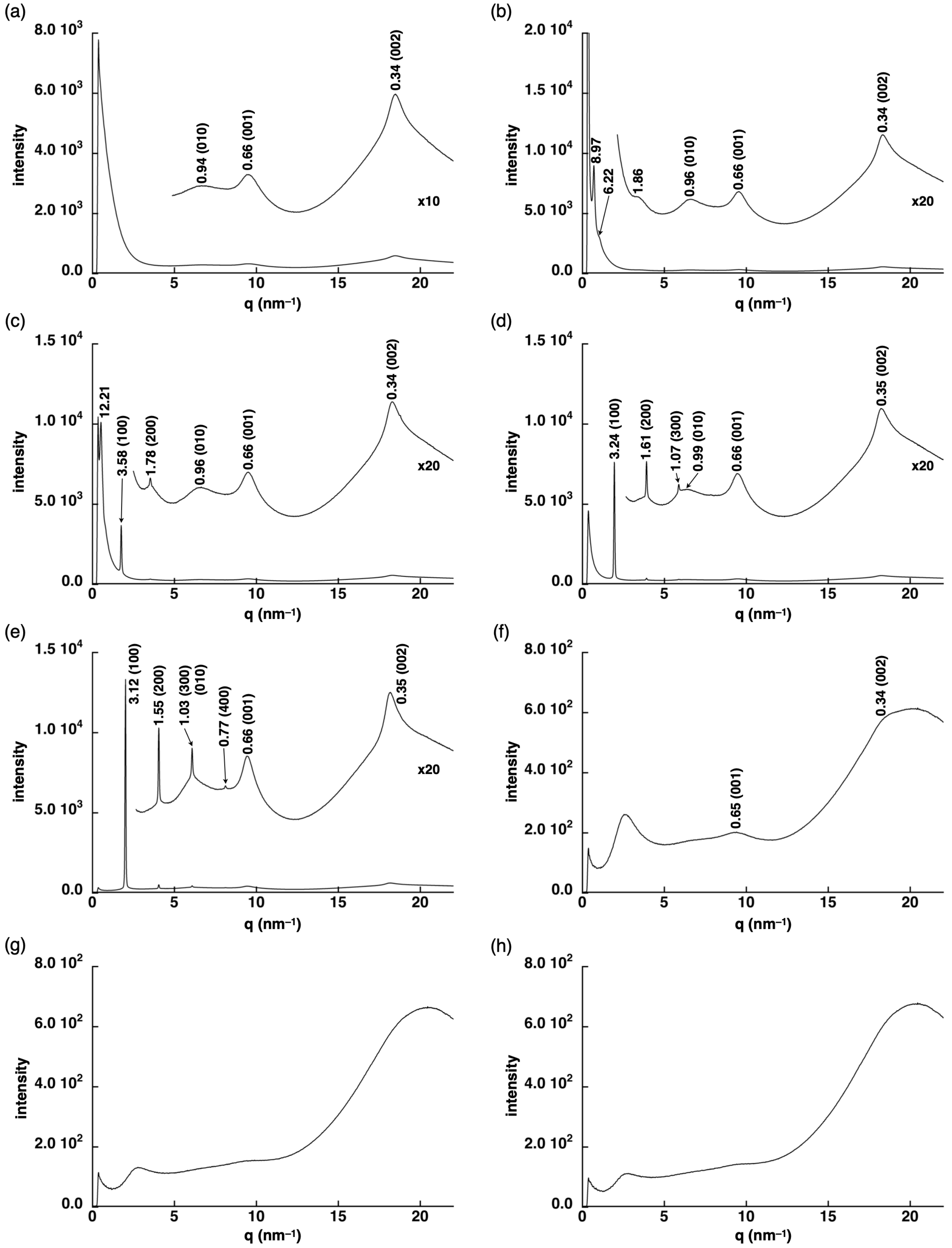

Figure S102 XRD patterns of **1au**$^{+}$-PCCp$^{-}_{20\%}$ at (a) 25 °C, (b) 40 °C, (c) 50 °C, (d) 60 °C, (e) 70 °C, (f) 80 °C, (g) 90 °C, (h) 80 °C, (i) 70 °C, (j) 60 °C, (k) 50 °C, (l) 40 °C, (m) 20 °C, (n) 5 °C, (o) 20 °C, (p) 40 °C, (q) 50 °C, (r) 60 °C, (s) 70 °C, (t) 80 °C, and (o) 90 °C upon (a–g) 1st heating, (h–n) 1st cooling, and (o–u) 2nd heating.

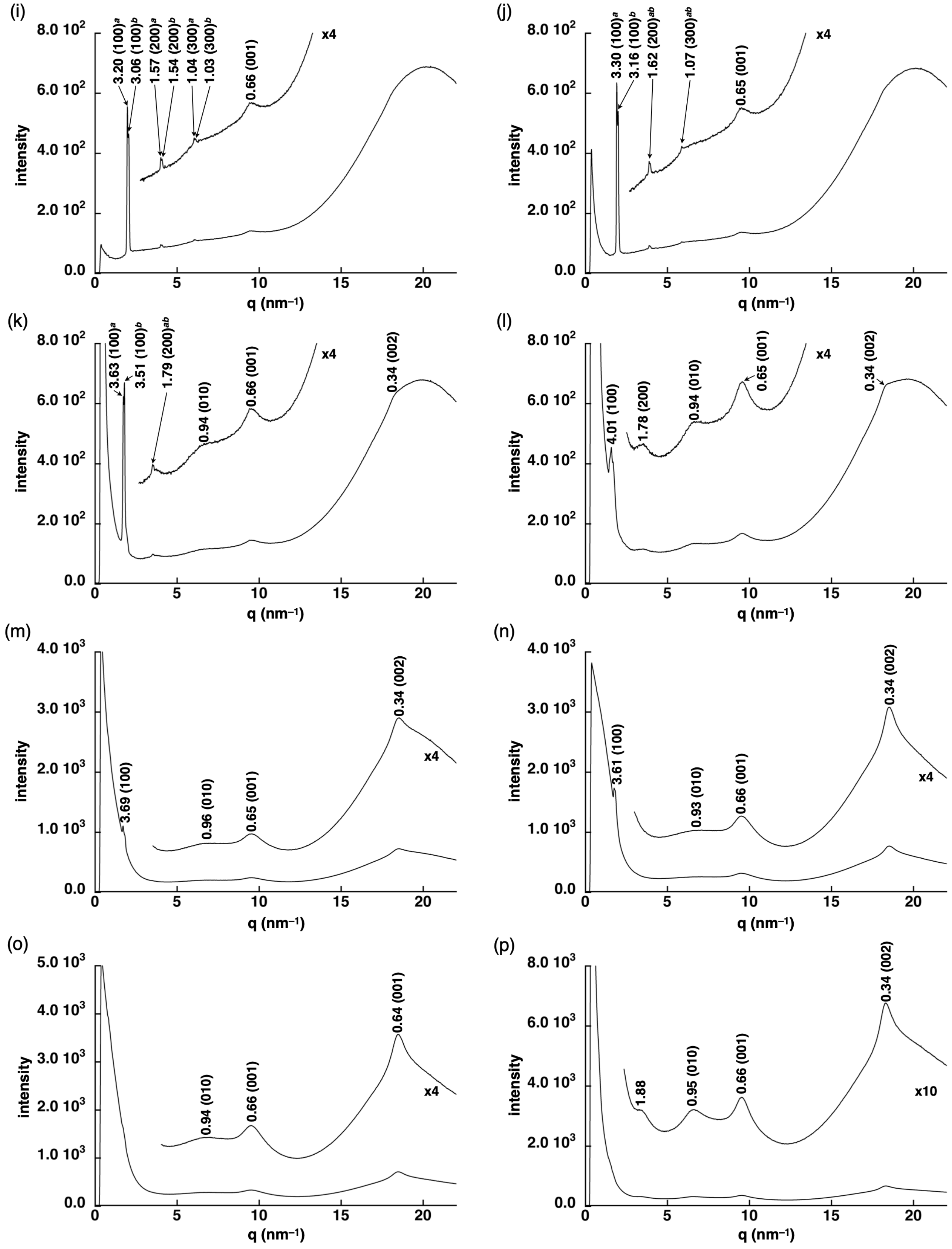

Figure S102 (Continued)

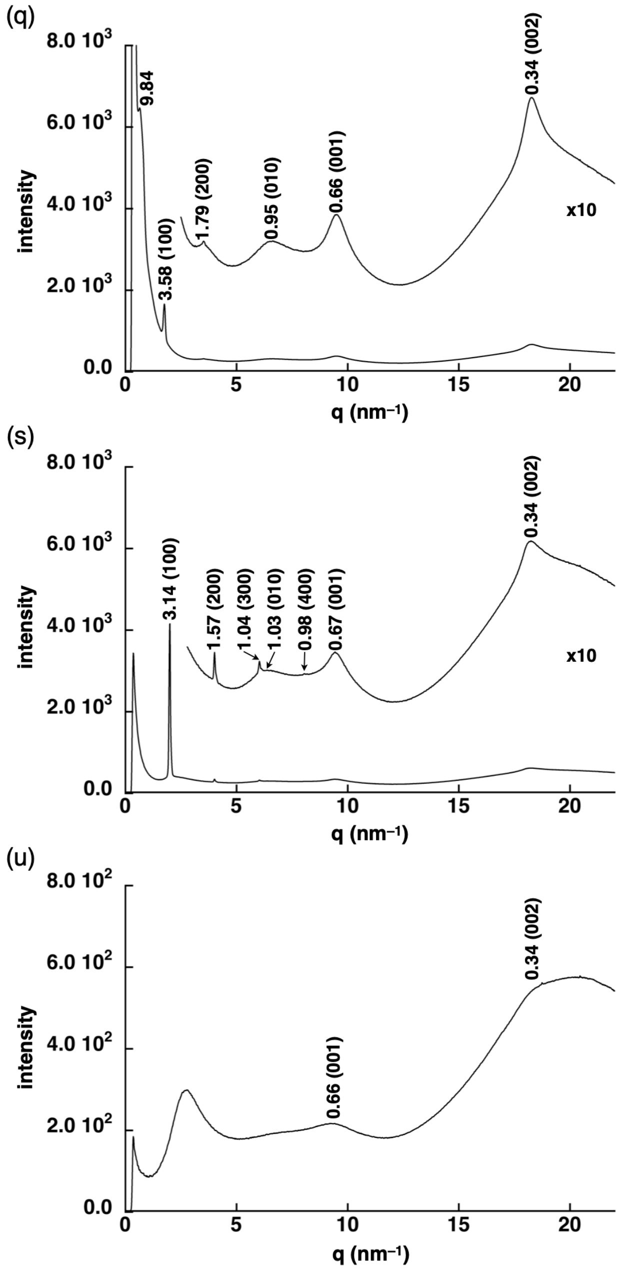

Figure S102 (Continued)

Table S15 Summary of XRD data of **1au**$^{+}$-PCCp$^{-}_{20\%}$. The peaks which can be indexed are represented.

	q (nm^{-1})	*d*-spacing (nm)	ratio	ratio (calc.)	*hkl*
	1.75	3.58	1.000	1.0000	100
(c) 50 °C (1st heating)	3.52	1.78	0.498	0.5000	200
Lam$_{col}$′	6.52	0.96	–	–	010
a = 3.58 nm, b = 0.96 nm, c = 0.66 nm	9.50	0.66	–	–	001
	18.3	0.34	–	–	002
	1.94	3.24	1.000	1.0000	100
(d) 60 °C (1st heating)	3.91	1.61	0.497	0.5000	200
Lam$_{col}$′	5.87	1.07	0.331	0.3333	300
a = 3.24 nm, b = 0.99 nm, c = 0.66 nm	6.32	0.99	–	–	010
	9.45	0.66	–	–	001
	18.2	0.35	–	–	002
	2.01	3.12	1.000	1.0000	100
(e) 70 °C (1st heating)	4.04	1.55	0.498	0.5000	200
Lam$_{col}$′	6.08	1.03	0.332	0.3333	300, 010
a = 3.12 nm, b = 1.03 nm, c = 0.66 nm	8.12	0.77	0.248	0.2500	400
	9.45	0.66	–	–	001
	18.2	0.35	–	–	002
	1.97^{a}	3.20	1.000	1.0000	100
(i) 70 °C (1st cooling)	2.05^{b}	3.06	1.000	1.0000	100
^{a}Lam$_{col}$	4.01^{a}	1.57	0.491	0.5000	200
a = 3.20 nm, c = 0.66 nm	4.08^{b}	1.54	0.503	0.5000	200
^{b}Lam$_{col}$	6.04^{a}	1.04	0.325	0.3333	300
a = 3.06 nm, c = 0.66 nm	6.12^{b}	1.03	0.335	0.3333	300
	9.46	0.66	–	–	001
(j) 60 °C (1st cooling)	1.90^{a}	3.30	1.000	1.0000	100
^{a}Lam$_{col}$	1.99^{b}	3.16	1.000	1.0000	100
a = 3.30 nm, c = 0.65 nm	3.88^{ab}	1.62	0.490^{a}, 0.513^{b}	0.5000	200
^{b}Lam$_{col}$	5.88^{ab}	1.07	0.324^{a}, 0.339^{b}	0.3333	300
a = 3.16 nm, c = 0.65 nm	9.71	0.65	–	–	001
(k) 50 °C (1st cooling)	1.73^{a}	3.63	1.000	1.0000	100
^{a}Lam$_{col}$	1.79^{b}	3.51	1.000	1.0000	100
a = 3.63 nm, b = 0.94 nm, c = 0.66 nm	3.51^{ab}	1.79	0.492^{a}, 0.510^{b}	0.5000	200
^{b}Lam$_{col}$	6.69	0.94	–	–	010
a = 3.51 nm, b = 0.94 nm, c = 0.66 nm	9.52	0.66	–	–	001
	18.4	0.34	–	–	002
	1.57	4.01	1.000	1.0000	100
(l) 40 °C (1st cooling)	3.52	1.78	0.445	0.5000	200
Lam$_{col}$	6.65	0.94	–	–	010
a = 4.01 nm, b = 0.94 nm, c = 0.65 nm	9.62	0.65	–	–	001
	18.4	0.34	–	–	002
	1.75	3.58	1.000	1.0000	100
(q) 50 °C (2nd heating)	3.51	1.79	0.500	0.5000	200
Lam$_{col}$	6.63	0.95	–	–	010
a = 3.58 nm, b = 0.95 nm c = 0.66 nm	9.45	0.66	–	–	001
	18.2	0.34	–	–	002
	1.94	3.24	1.000	1.0000	100
	3.88	1.62	0.500	0.5000	200
(r) 60 °C (2nd heating)	5.84	1.08	0.332	0.3333	300
Lam$_{col}$	6.49	0.97	–	–	010
a = 3.24 nm, b = 0.97 nm c = 0.66 nm	9.50	0.66	–	–	001
	18.2	0.34	–	–	002

Table S15 (Continued)

	q (nm^{-1})	*d*-spacing (nm)	ratio	ratio (calc.)	*hkl*
	2.00	3.14	1.000	1.0000	100
	4.01	1.57	0.500	0.5000	200
(s) 70 °C (2nd heating)	6.03	1.04	0.332	0.3333	300
Lam$_{col}$	6.11	1.03	–	–	010
a = 3.14 nm, b = 1.03 nm c = 0.67 nm	6.38	0.98	0.314	0.2500	400
	9.43	0.67	–	–	001
	18.2	0.34	–	–	002

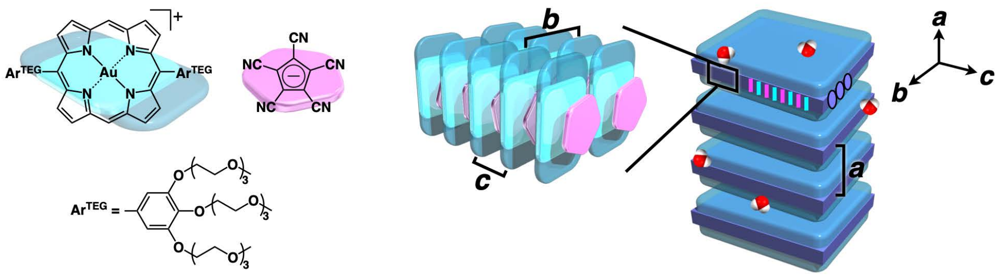

Figure S103 Possible packing model of **1au**$^{+}$-PCCp$^{-}_{20\%}$ as a Lam$_{col}$ structure.

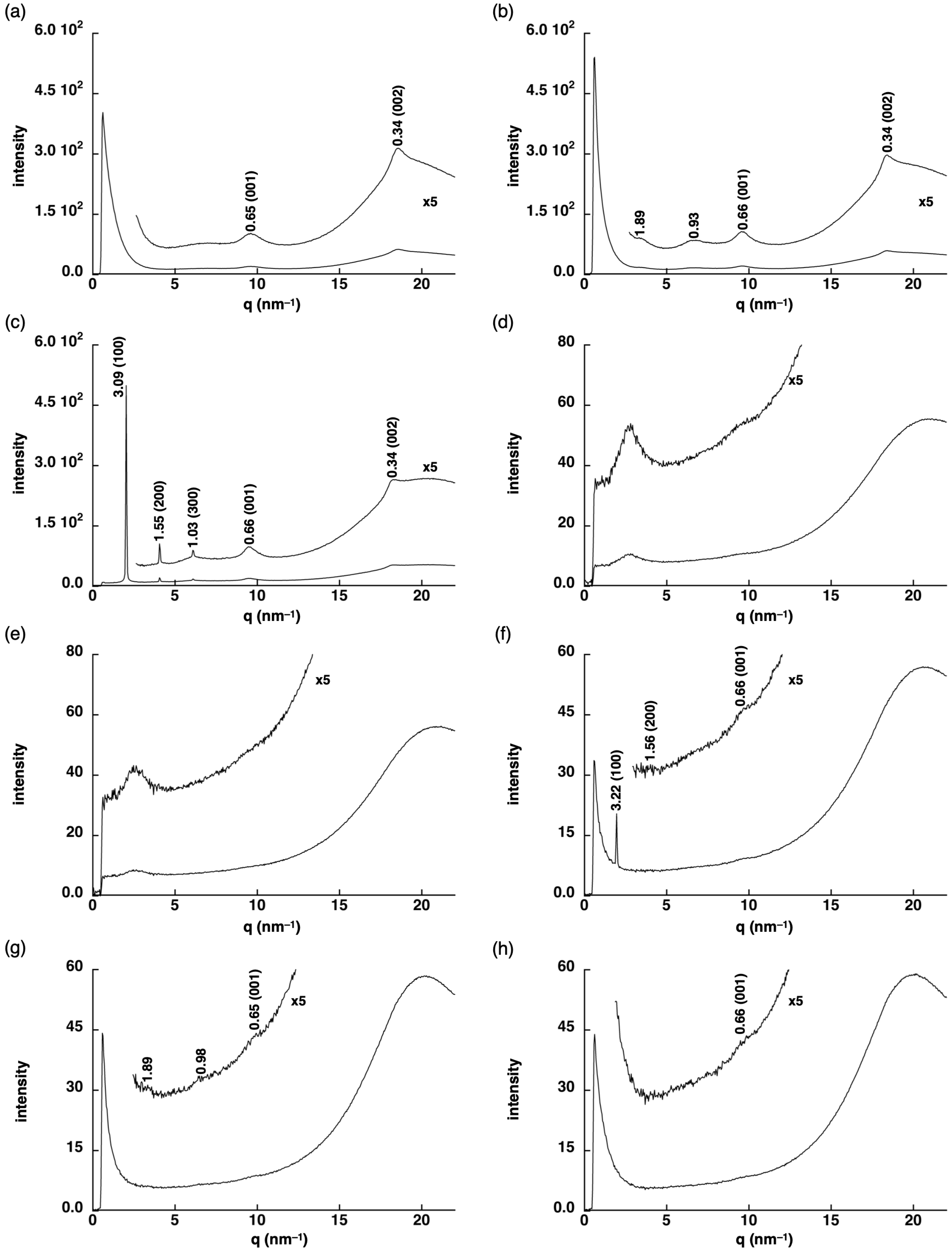

Figure S104 XRD patterns of **1au**$^+$-PCCp$^-_{10\%}$ at (a) 25 °C, (b) 40 °C, (c) 70 °C, (d) 90 °C, (e) 80 °C, (f) 60 °C, (g) 30 °C, (h) 20 °C, (i) 5 °C, (j) 20 °C, (k) 40 °C, (l) 70 °C, and (m) 90 °C upon (a–d) 1st heating, (e–i) 1st cooling, and (j–m) 2nd heating.

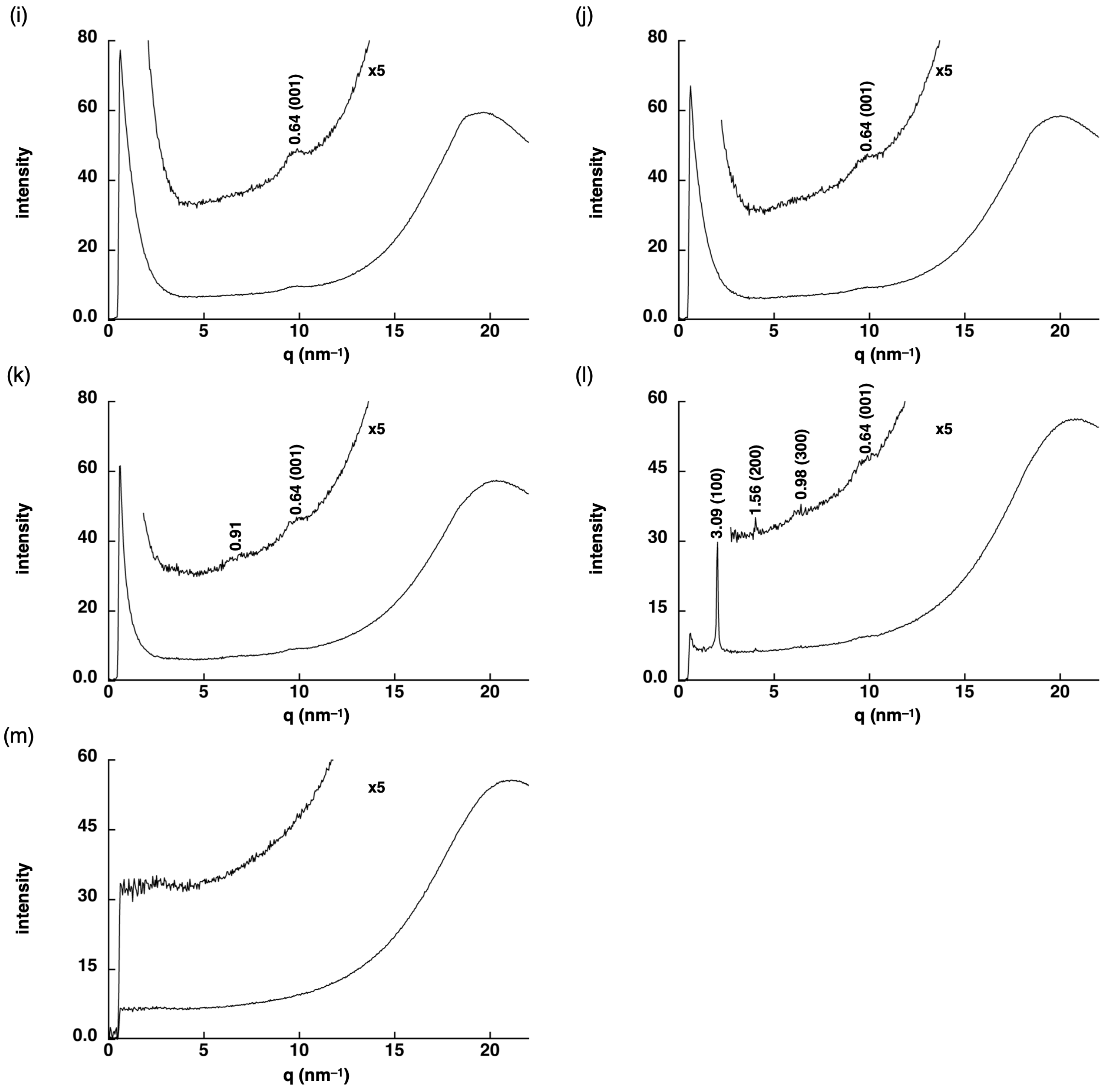

Figure S104 (Continued)

Table S16 Summary of XRD data of **1au**$^{+}$-PCCp$^{-}_{10\%}$. The peaks which can be indexed are represented.

	q (nm^{-1})	*d*-spacing (nm)	ratio	ratio (calc.)	*hkl*
	2.03	3.09	1.000	1.0000	100
(c) 70 °C (1st heating)	4.06	1.55	0.501	0.5000	200
Lam$_{col}$′	6.08	1.03	0.334	0.3333	300
a = 3.09 nm, c = 0.66 nm	9.48	0.66	–	–	001
	18.4	0.34	–	–	002
(f) 60 °C (1st cooling)	1.95	3.22	1.000	1.0000	100
Lam$_{col}$′	4.02	1.56	0.486	0.5000	200
a = 3.22 nm, c = 0.66 nm	9.52	0.66	–	–	001
(l) 70 °C (2nd heating)	2.03	3.09	1.000	1.0000	100
Lam$_{col}$′	4.02	1.56	0.506	0.5000	200
a = 3.09 nm, c = 0.64 nm	6.41	0.98	0.317	0.3333	300
	9.77	0.64	–	–	001

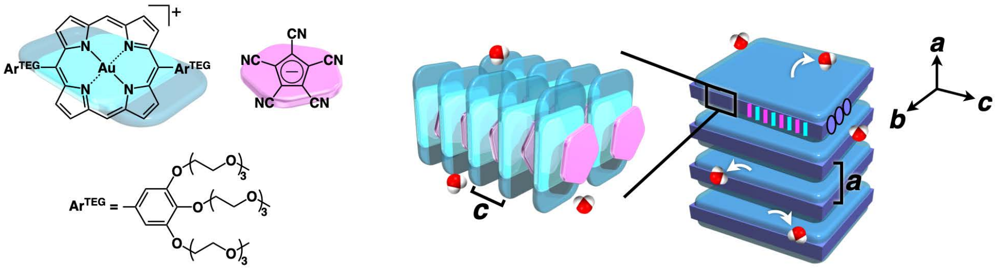

Figure S105 Possible packing model of **1au**$^{+}$-PCCp$^{-}{}_{10\%}$ as a Lam$_{col}$′ structure.

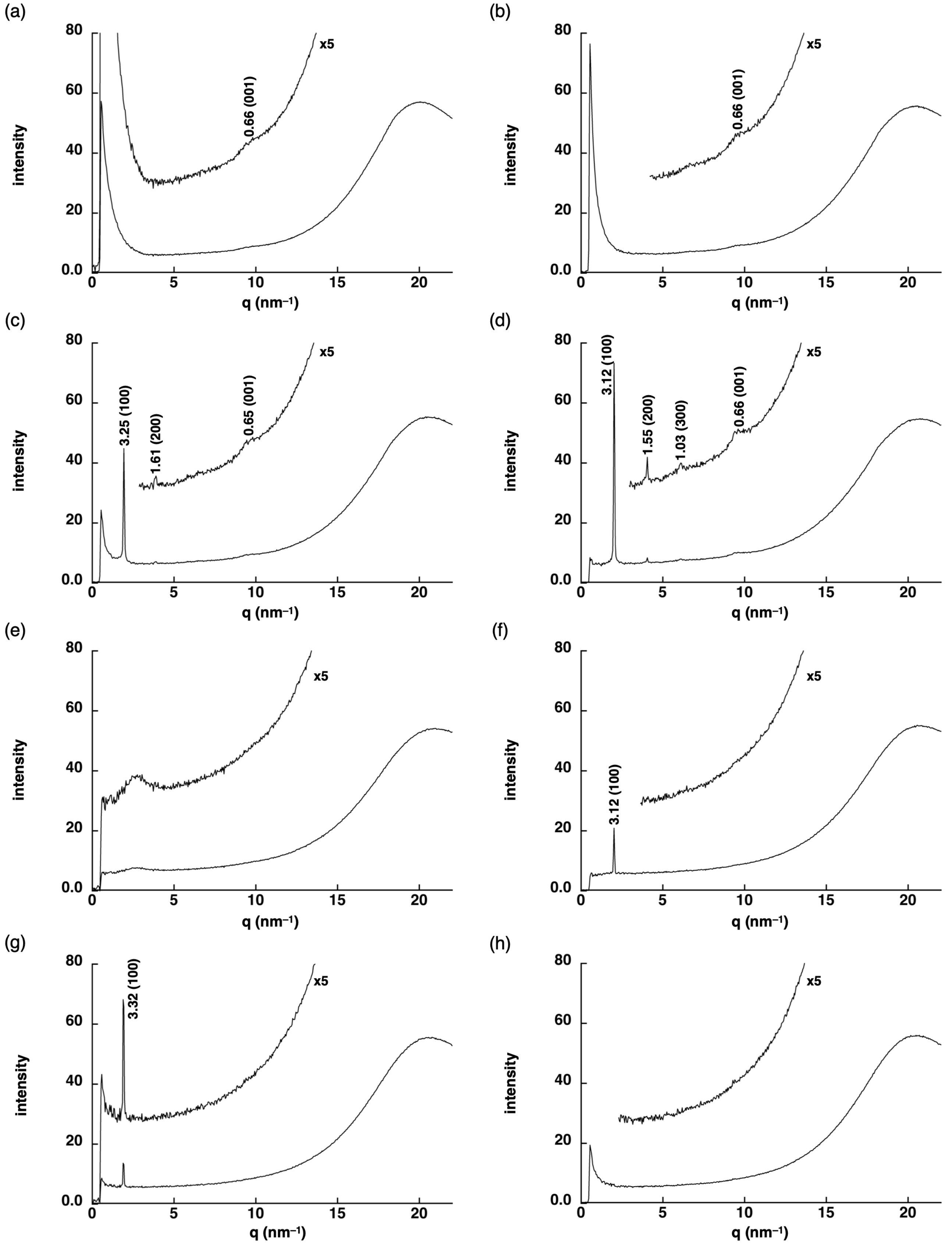

Figure S106 XRD patterns of **1au**$^+$-PCCp$^-_{1\%}$ at (a) 25 °C, (b) 50 °C, (c) 60 °C, (d) 70 °C, (e) 90 °C, (f) 70 °C, (g) 60 °C, (h) 50 °C, (i) 20 °C, (j) 5 °C, (k) 20 °C, (l) 50 °C, (m) 70 °C, and (n) 80 °C upon (a–e) 1st heating, (f–j) 1st cooling, and (k–n) 2nd heating.

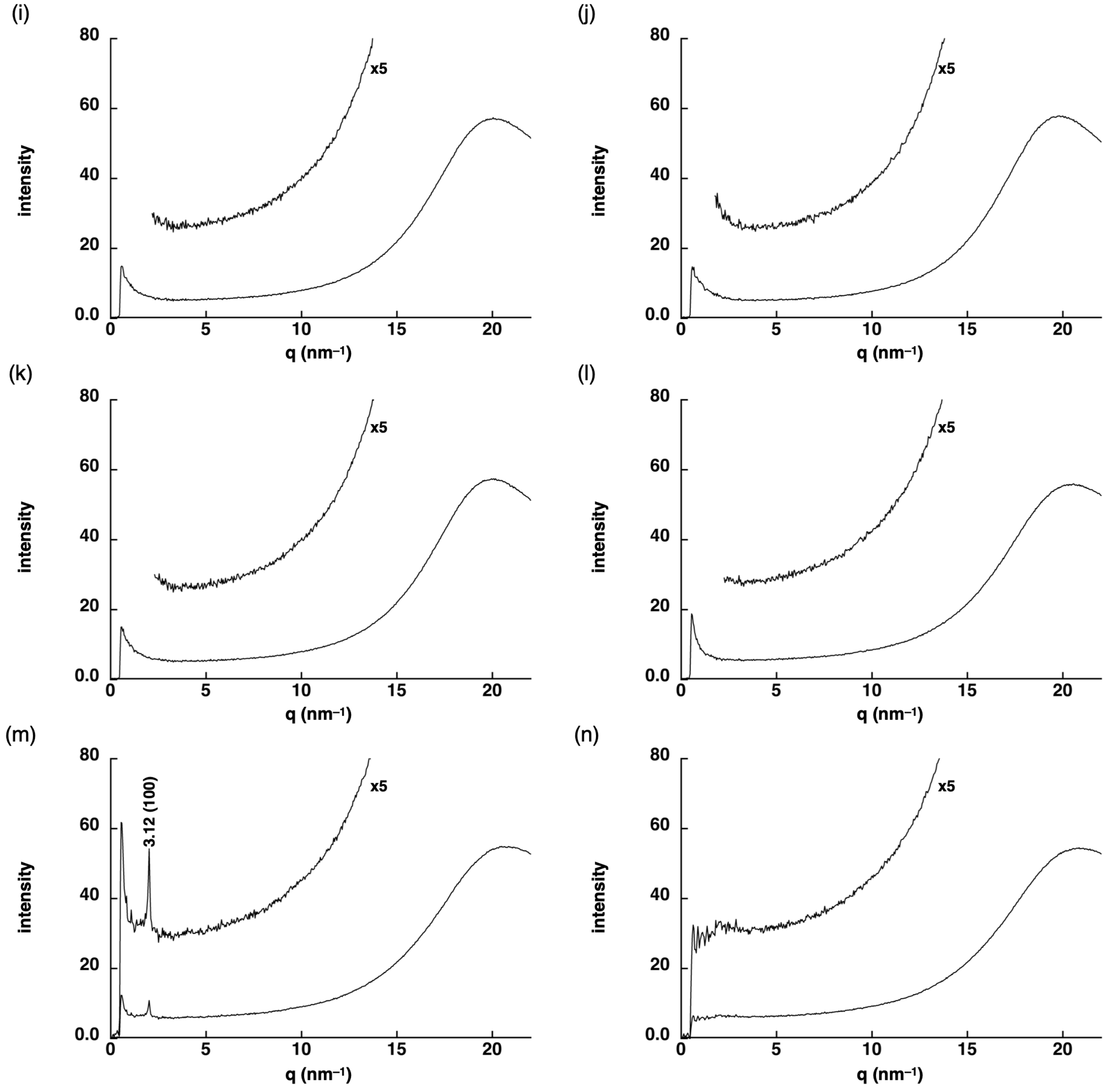

Figure S106 (Continued)

Table S17 Summary of XRD data of **1au**$^{+}$-PCCp$^{-}_{1\%}$. The peaks which can be indexed are represented.

	q (nm^{-1})	*d*-spacing (nm)	ratio	ratio (calc.)	*hkl*
(c) 60 °C (1st heating)	1.93	3.25	1.000	1.0000	100
Lam$_{col}$′	3.89	1.61	0.496	0.5000	200
a = 3.25 nm, *c* = 0.65 nm	9.73	0.65	–	–	001
(d) 70 °C (1st heating)	2.01	3.12	1.000	1.0000	100
Lam$_{col}$′	4.05	1.55	0.497	0.5000	200
a = 3.12 nm, *c* = 0.66 nm	6.09	1.03	0.330	0.3333	300
	9.52	0.66	–	–	001

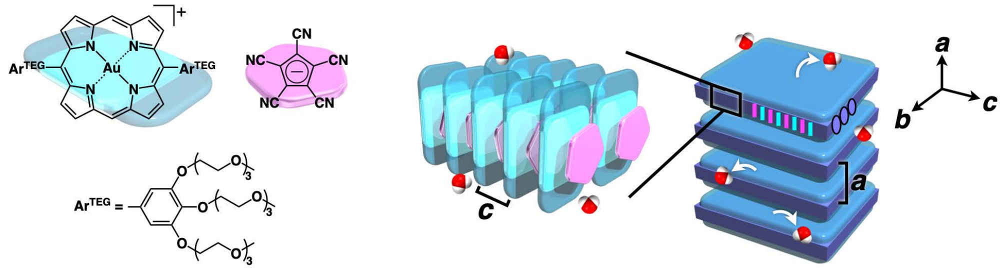

Figure S107 Possible packing model of **1au**$^{+}$-PCCp^{-}$_{1\%}$ as a Lam$_{col}$´ structure.

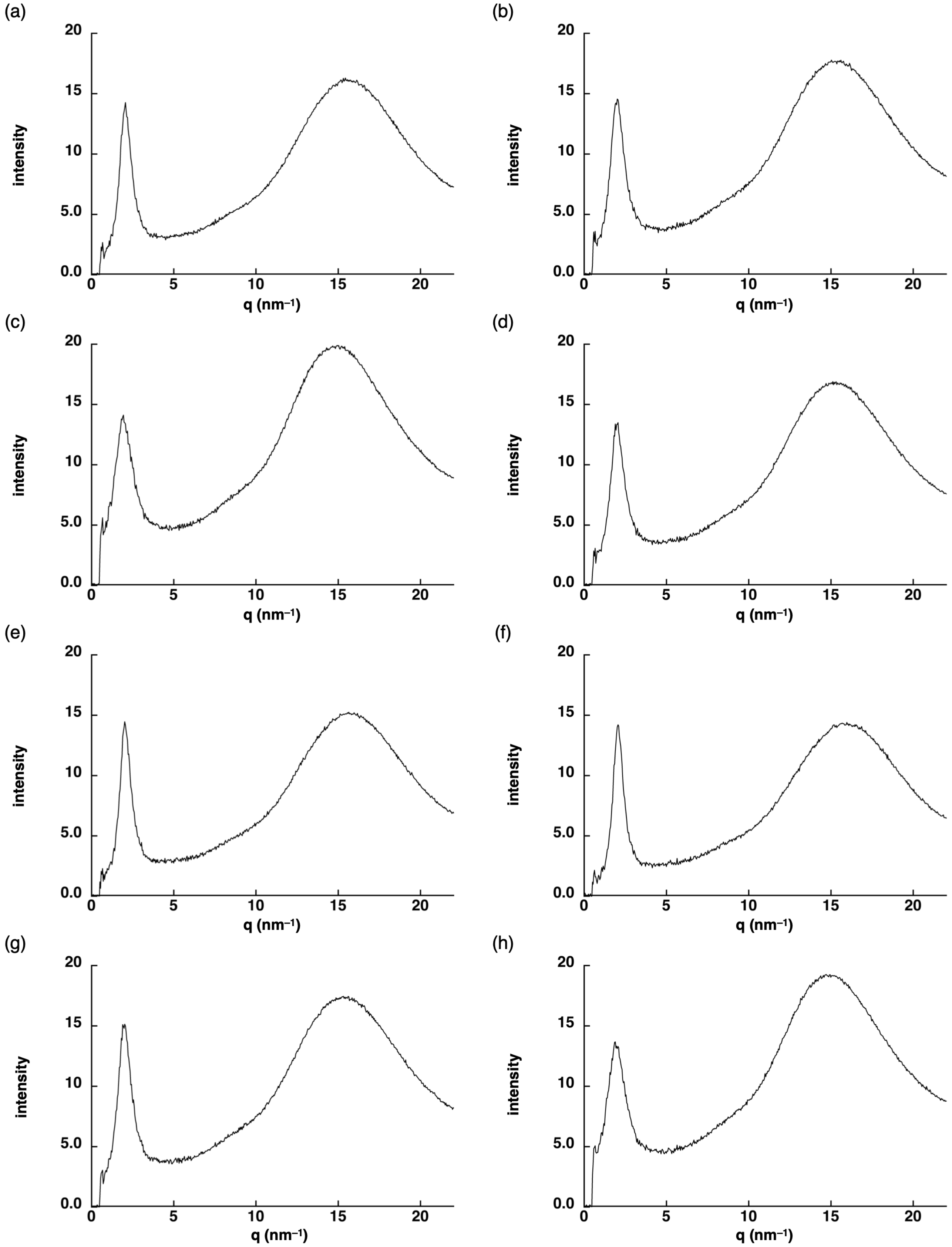

Figure S108 XRD patterns of $\mathbf{2}_{70\%}$ at (a) 25 °C, (b) 50 °C, (c) 80 °C, (d) 50 °C, (e) 20 °C, (f) 5 °C, (g) 50 °C, and (h) 80 °C upon (a–c) 1st heating, (d–f) 1st cooling, and (g,h) 2nd heating.

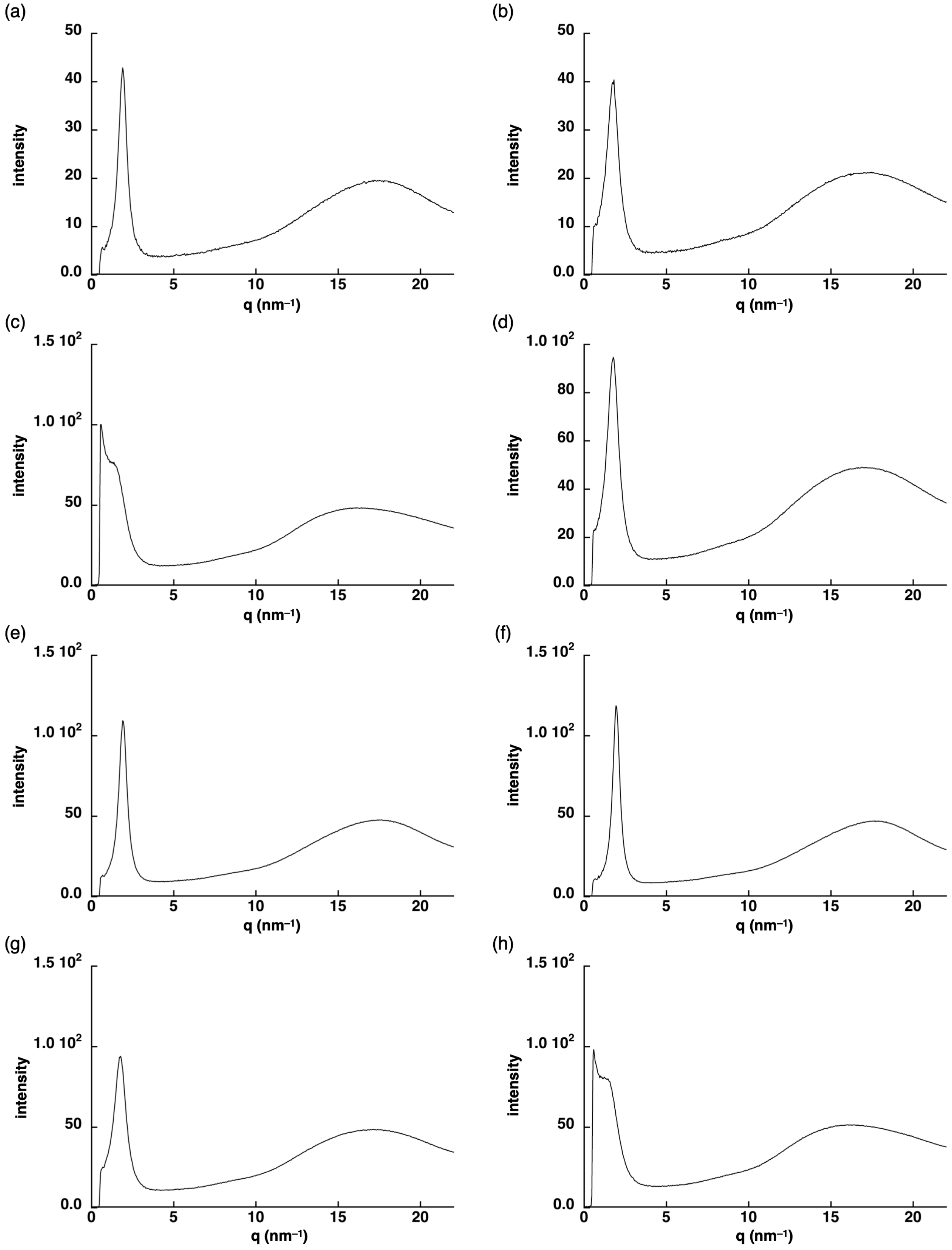

Figure S109 XRD patterns of $\mathbf{2}_{50\%}$ at (a) 25 °C, (b) 50 °C, (c) 80 °C, (d) 50 °C, (e) 20 °C, (f) 5 °C, (g) 50 °C, and (h) 80 °C upon (a–c) 1st heating, (d–f) 1st cooling, and (g,h) 2nd heating.

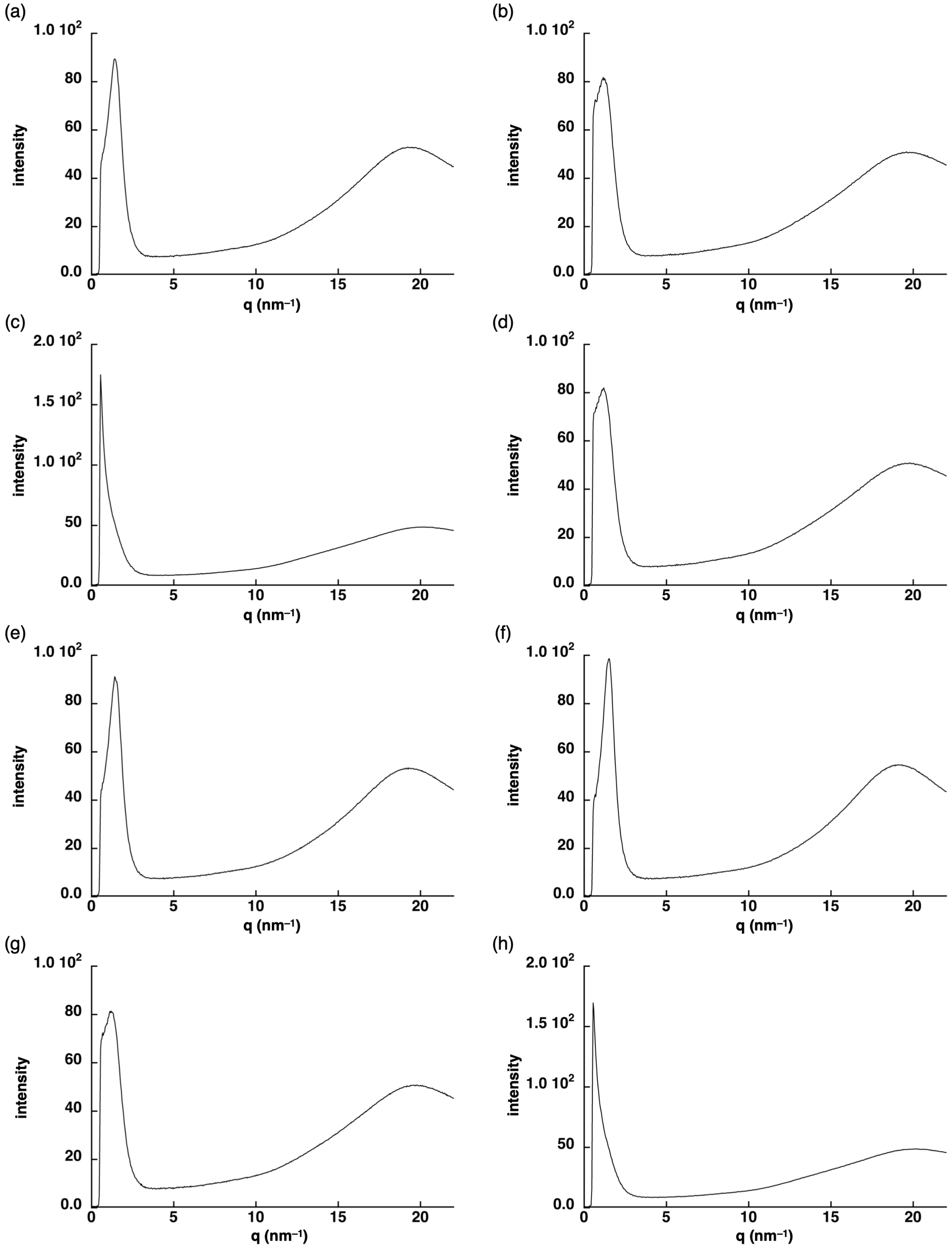

Figure S110 XRD patterns of $\mathbf{2}_{20\%}$ at (a) 25 °C, (b) 50 °C, (c) 80 °C, (d) 50 °C, (e) 20 °C, (f) 5 °C, (g) 50 °C, and (h) 80 °C upon (a–c) 1st heating, (d–f) 1st cooling, and (g,h) 2nd heating.

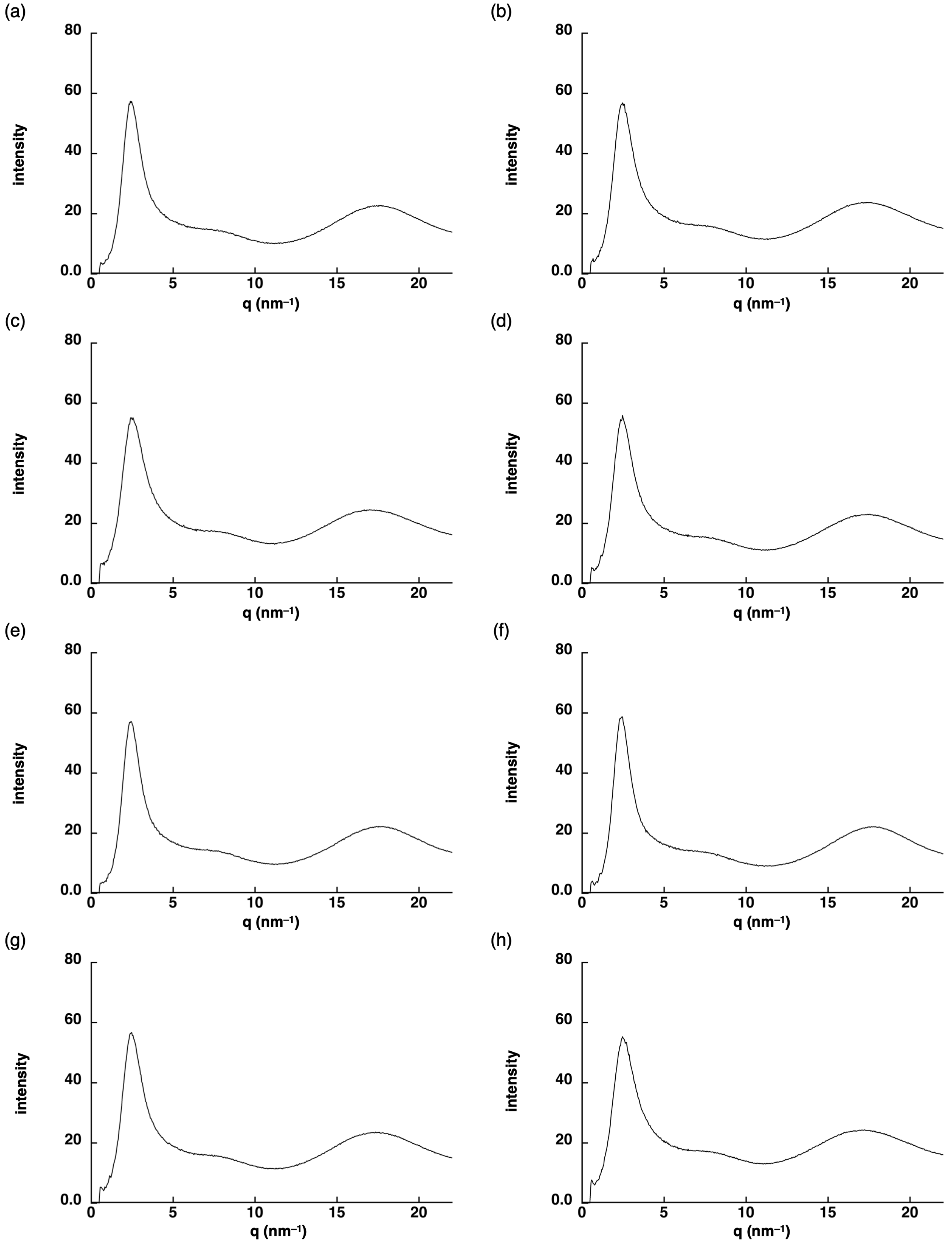

Figure S111 XRD patterns of **2au**$^+$-Cl$^-_{70\%}$ at (a) 25 °C, (b) 50 °C, (c) 80 °C, (d) 50 °C, (e) 20 °C, (f) 5 °C, (g) 50 °C, and (h) 80 °C upon (a–c) 1st heating, (d–f) 1st cooling, and (g,h) 2nd heating.

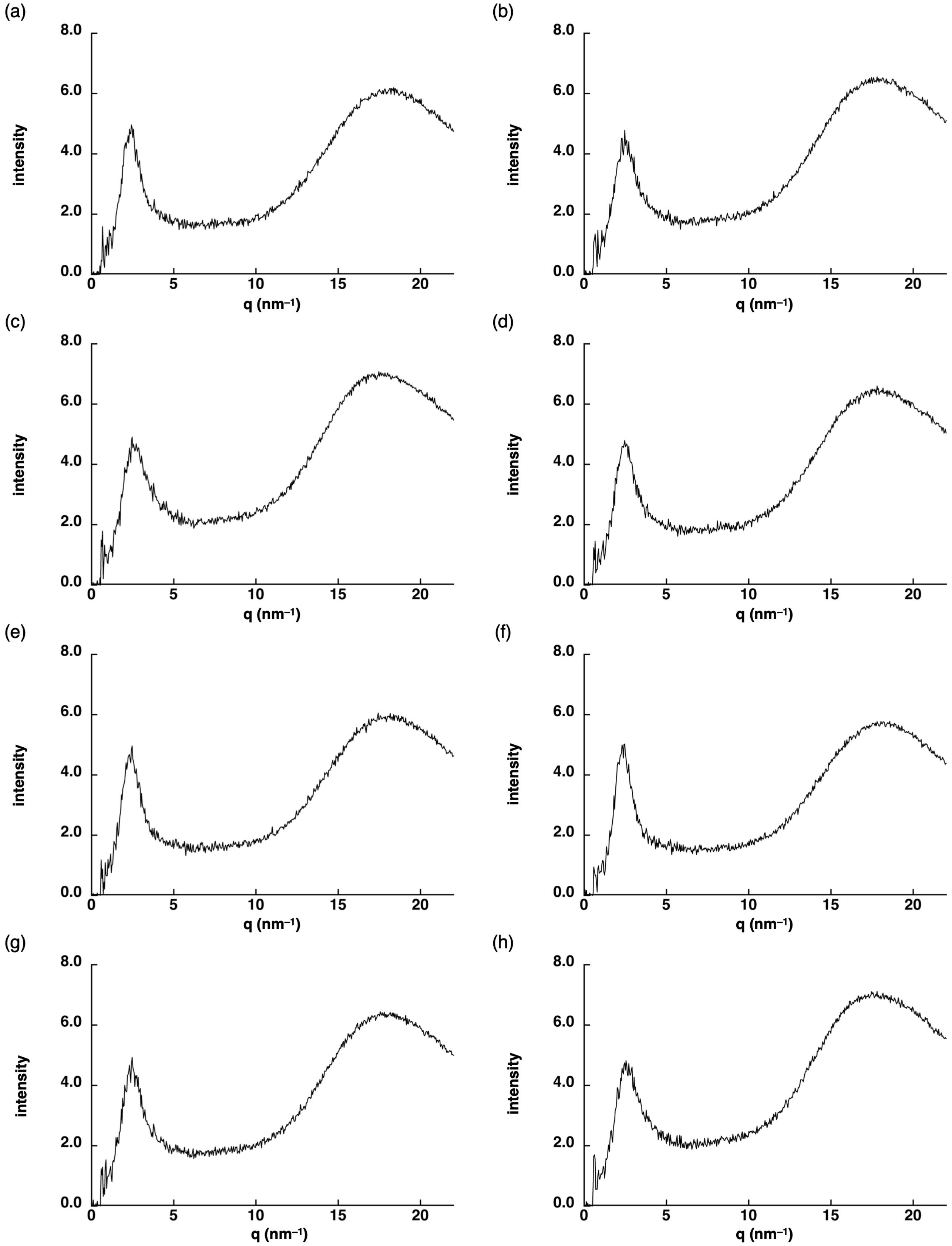

Figure S112 XRD patterns of **2au**$^+$-Cl$^-_{50\%}$ at (a) 25 °C, (b) 50 °C, (c) 80 °C, (d) 50 °C, (e) 20 °C, (f) 5 °C, (g) 50 °C, and (h) 80 °C upon (a–c) 1st heating, (d–f) 1st cooling, and (g,h) 2nd heating.

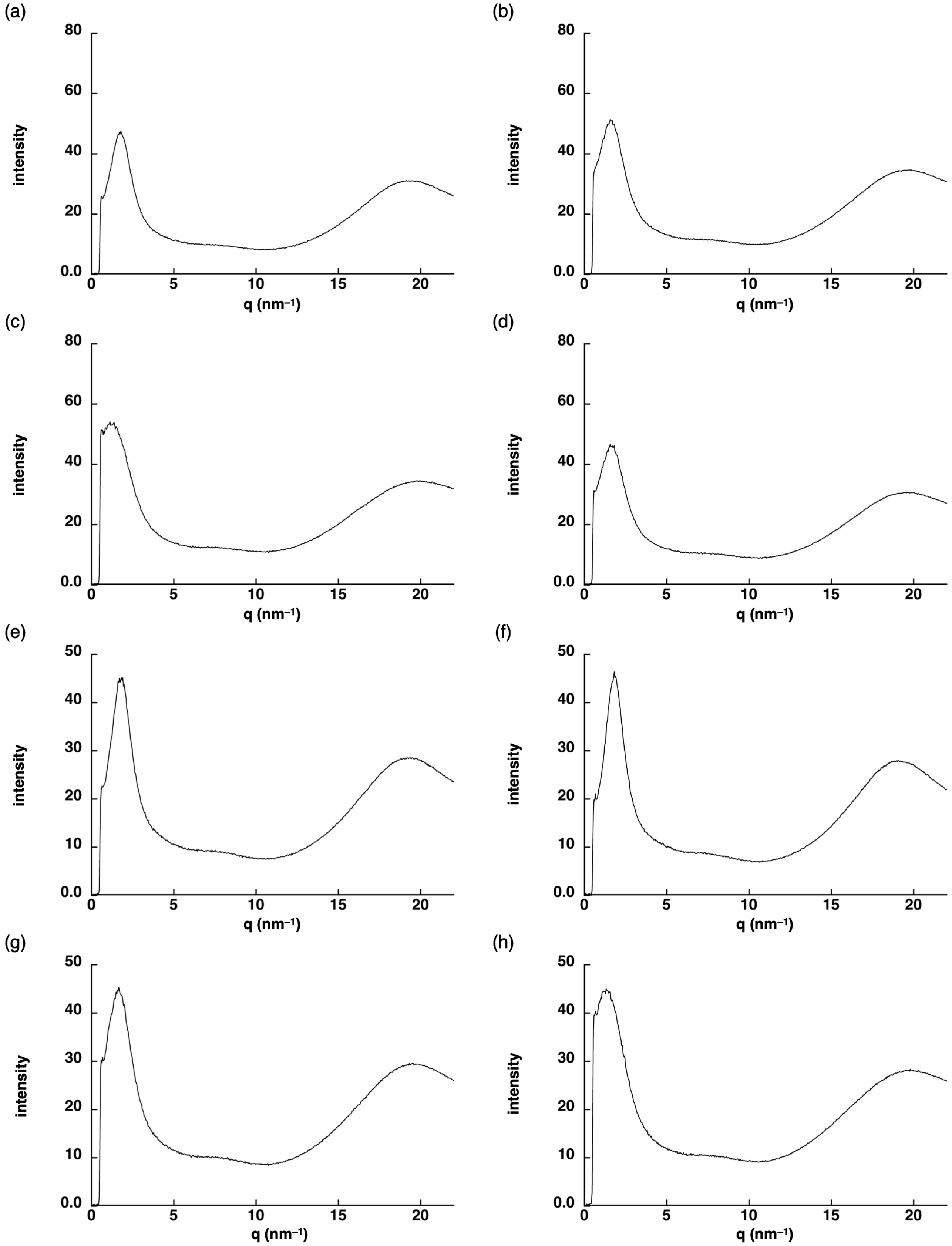

Figure S113 XRD patterns of **2au**$^+$-Cl$^-_{20\%}$ at (a) 25 °C, (b) 50 °C, (c) 80 °C, (d) 50 °C, (e) 20 °C, (f) 5 °C, (g) 50 °C, and (h) 80 °C upon (a–c) 1st heating, (d–f) 1st cooling, and (g,h) 2nd heating.

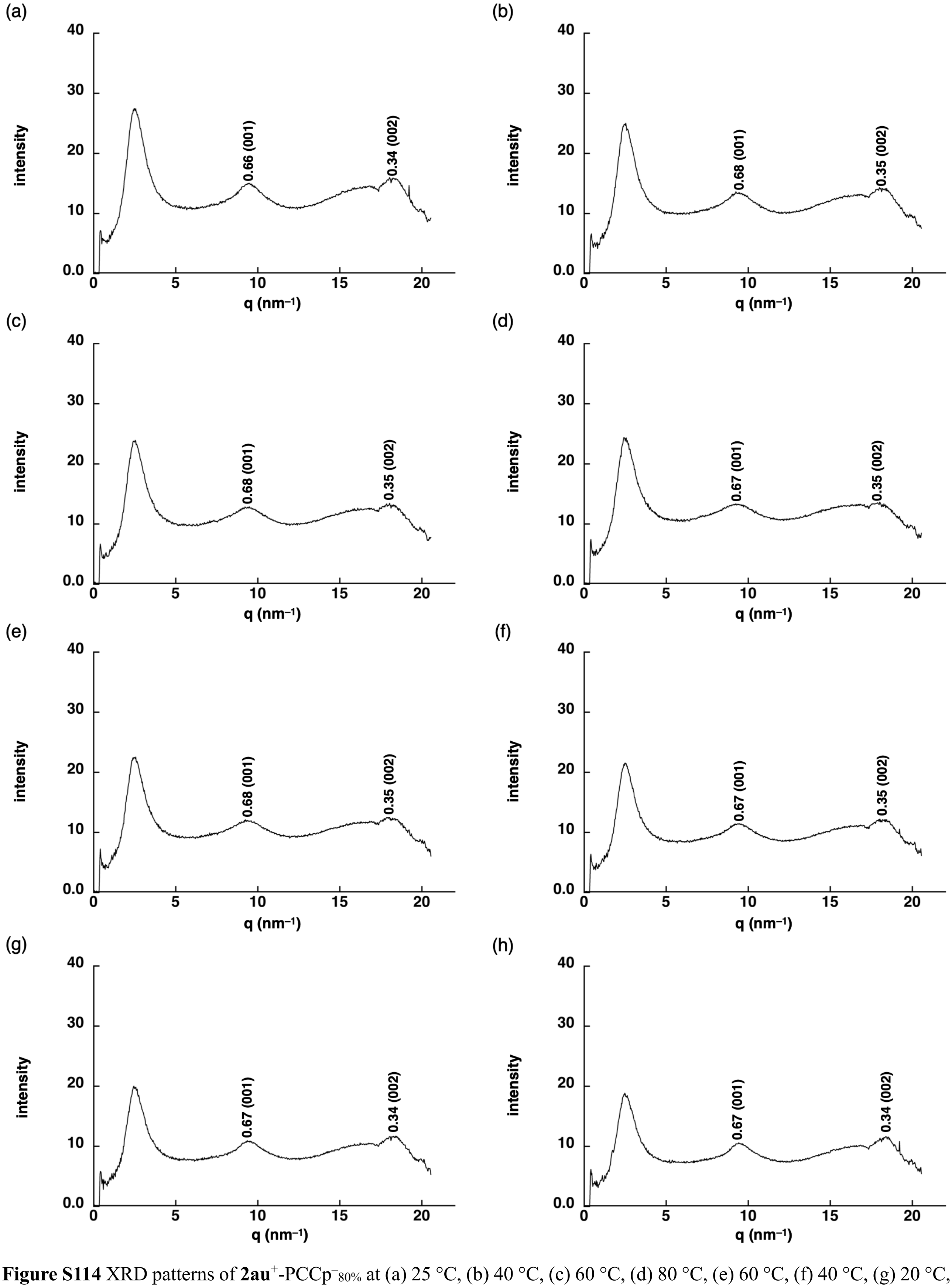

Figure S114 XRD patterns of **2au**$^{+}$-PCCp$^{-}_{80\%}$ at (a) 25 °C, (b) 40 °C, (c) 60 °C, (d) 80 °C, (e) 60 °C, (f) 40 °C, (g) 20 °C, (h) 5 °C, (i) 20 °C, (j) 40 °C, (k) 60 °C, and (l) 80 °C upon (a–d) 1st heating, (e–h) 1st cooling, and (i–l) 2nd heating.

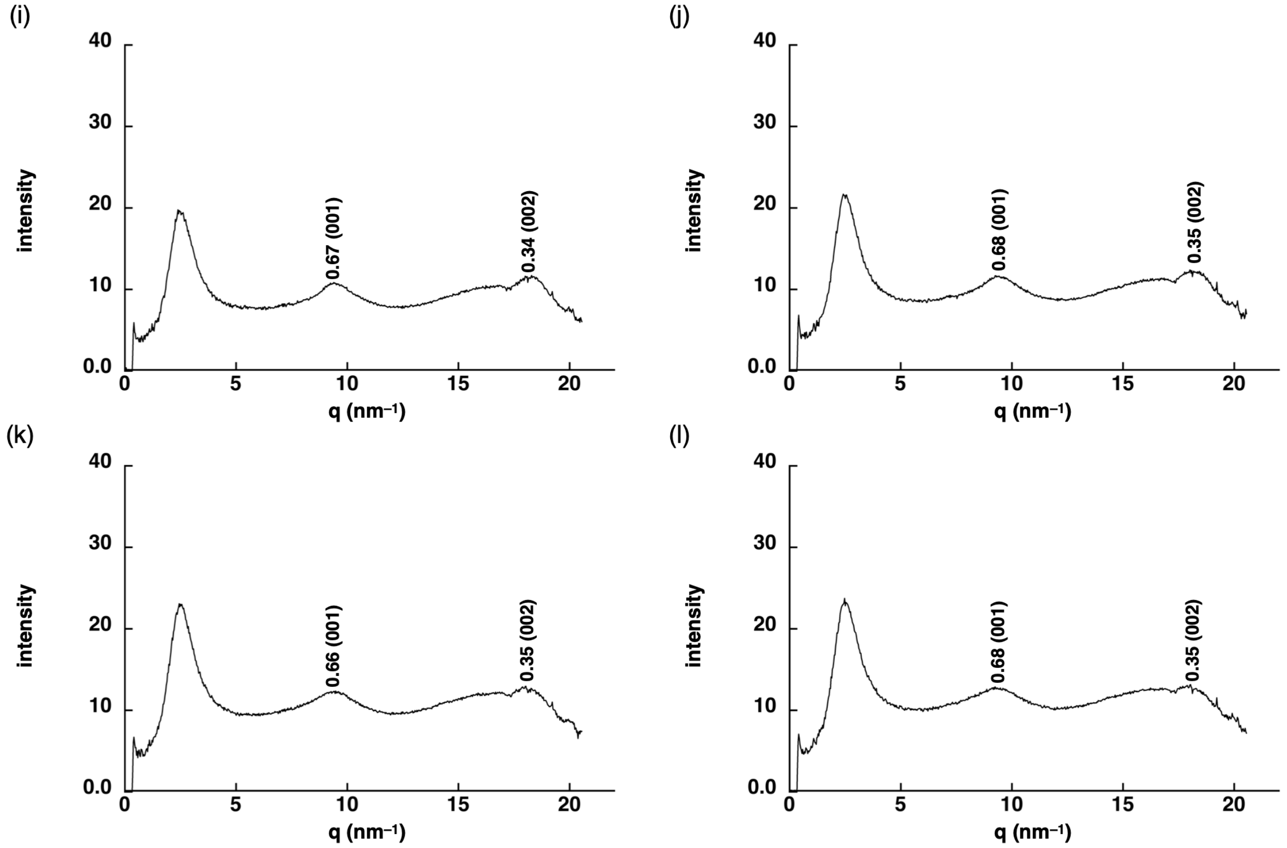

Figure S114 (Continued)

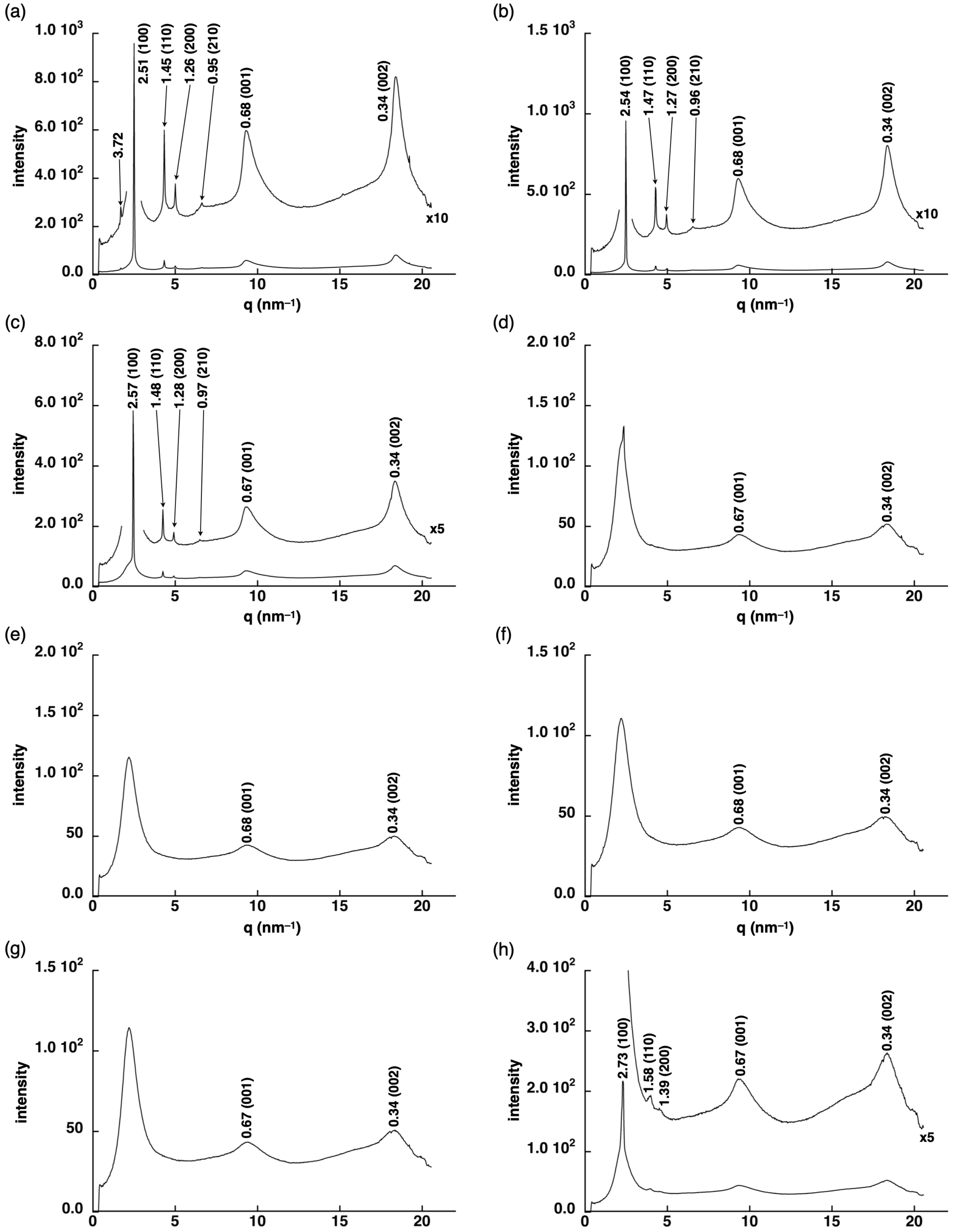

Figure S115 XRD patterns of **2au**$^+$-PCCp$^-_{70\%}$ at (a) 25 °C, (b) 40 °C, (c) 50 °C, (d) 60 °C, (e) 70 °C, (f) 80 °C, (g) 70 °C, (h) 60 °C, (i) 50 °C, (j) 20 °C, (k) 5 °C, (l) 20 °C, (m) 50 °C, (n) 60 °C, (o) 70 °C, and (p) 80 °C upon (a–f) 1st heating, (g–k) 1st cooling, and (l–p) 2nd heating.

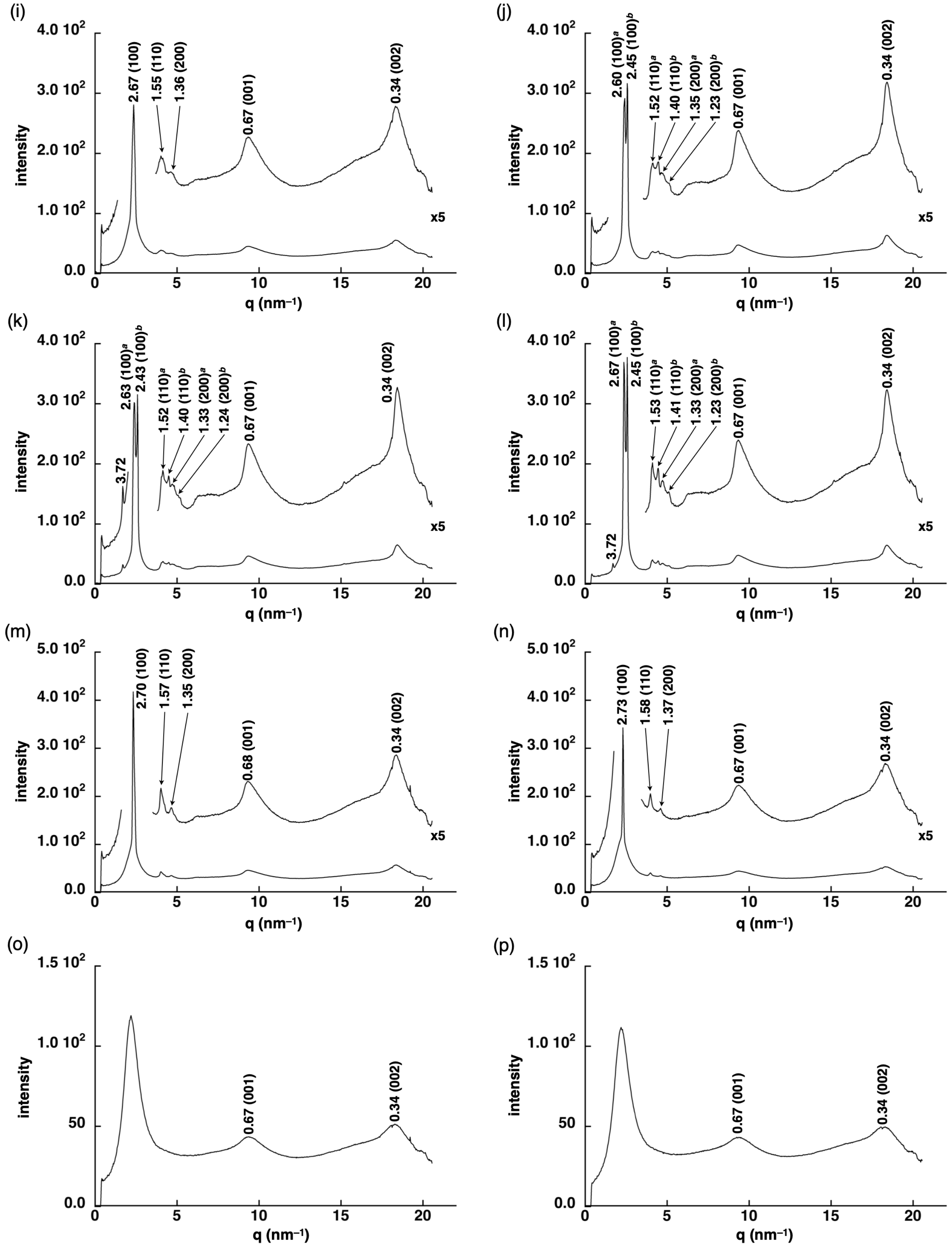

Figure S115 (Continued)

Table S18 Summary of XRD data of **2au**$^+$-PCCp$^-$$_{70\%}$. The peaks which can be indexed are represented.

	q (nm^{-1})	*d*-spacing (nm)	ratio	ratio (calc.)	*hkl*
	2.50	2.51	1.000	1.0000	100
(a) 25 °C (1st heating)	4.33	1.45	0.577	0.5774	110
Col$_h$	5.00	1.26	0.500	0.5000	200
a = 2.90 nm, c = 0.68 nm	6.63	0.95	0.377	0.3780	210
Z = 1 (ρ = 0.88)	9.27	0.68	–	–	001
	18.4	0.34	–	–	002
	2.47	2.54	1.000	1.0000	100
(b) 40 °C (1st heating)	4.28	1.47	0.578	0.5774	110
Col$_h$	4.94	1.27	0.500	0.5000	200
a = 2.93 nm, c = 0.68 nm	6.54	0.96	0.378	0.3780	210
Z = 1 (ρ = 0.86)	9.30	0.68	–	–	001
	18.3	0.34	–	–	002
	2.44	2.57	1.000	1.0000	100
(c) 50 °C (1st heating)	4.25	1.48	0.576	0.5774	110
Col$_h$	4.92	1.28	0.497	0.5000	200
a = 2.97 nm, c = 0.67 nm	6.48	0.97	0.377	0.3780	210
Z = 1 (ρ = 0.85)	9.36	0.67	–	–	001
	18.3	0.34	–	–	002
(h) 60 °C (1st cooling)	2.30	2.73	1.000	1.0000	100
Col$_h$	3.99	1.58	0.577	0.5774	110
a = 3.16 nm, c = 0.67 nm	4.51	1.39	0.510	0.5000	200
Z = 1 (ρ = 0.75)	9.36	0.67	–	–	001
	18.3	0.34	–	–	002
(i) 50 °C (1st cooling)	2.36	2.67	1.000	1.0000	100
Col$_h$	4.04	1.55	0.583	0.5774	110
a = 3.08 nm, c = 0.67 nm	4.62	1.36	0.510	0.5000	200
Z = 1 (ρ = 0.79)	9.33	0.67	–	–	001
	18.3	0.34	–	–	002
(j) 20 °C (1st cooling)	2.42^a	2.60	1.000	1.0000	100
^{a}Col$_h$	2.56^b	2.45	1.000	1.0000	100
a = 3.00 nm, c = 0.67 nm	4.13^a	1.52	0.585	0.5774	110
Z = 1 (ρ = 0.84)	4.48^b	1.40	0.572	0.5774	110
^{b}Col$_h$	4.65^a	1.35	0.519	0.5000	200
a = 2.83 nm, c = 0.67 nm	5.09^b	1.23	0.503	0.5000	200
Z = 1 (ρ = 0.94)	9.45	0.67	–	–	001
	18.4	0.34	–	–	002
(k) 5 °C (1st cooling)	2.39^a	2.63	1.000	1.0000	100
^{a}Col$_h$	2.59^b	2.43	1.000	1.0000	100
a = 3.04 nm, c = 0.67 nm	4.13^a	1.52	0.578	0.5774	110
Z = 1 (ρ = 0.81)	4.48^b	1.40	0.578	0.5774	110
^{b}Col$_h$	4.71^a	1.33	0.506	0.5000	200
a = 2.80 nm, c = 0.67 nm	5.06^b	1.24	0.512	0.5000	200
Z = 1 (ρ = 0.95)	9.36	0.67	–	–	001
	18.4	0.34	–	–	002
(l) 20 °C (2nd heating)	2.36^a	2.67	1.000	1.0000	100
^{a}Col$_h$	2.56^b	2.45	1.000	1.0000	100
a = 3.08 nm, c = 0.67 nm	4.10^a	1.53	0.575	0.5774	110
Z = 1 (ρ = 0.79)	4.45^b	1.41	0.575	0.5774	110
^{b}Col$_h$	4.71^a	1.33	0.500	0.5000	200
a = 2.83 nm, c = 0.67 nm	5.09^b	1.23	0.503	0.5000	200
Z = 1 (ρ = 0.93)	9.33	0.67	–	–	001
	18.4	0.34	–	–	002

Table S18 (Continued)

	q (nm^{-1})	*d*-spacing (nm)	ratio	ratio (calc.)	*hkl*
(m) 50 °C (2nd heating)	2.33	2.70	1.000	1.0000	100
Col_h	4.01	1.57	0.580	0.5774	110
a = 3.12 nm, c = 0.68 nm	4.65	1.35	0.500	0.5000	200
Z = 1 (ρ = 0.76)	9.30	0.68	–	–	001
	18.4	0.34	–	–	002
(n) 60 °C (2nd heating)	2.30	2.73	1.000	1.0000	100
Col_h	3.99	1.58	0.577	0.5774	110
a = 3.16 nm, c = 0.67 nm	4.60	1.37	0.500	0.5000	200
Z = 1 (ρ = 0.75)	9.36	0.67	–	–	001
	18.3	0.34	–	–	002

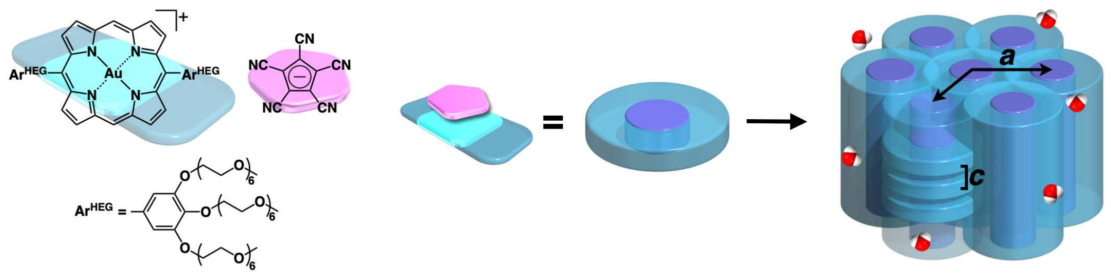

Figure S116 Possible packing model of **2au**$^{+}$-PCCp$^{-}_{70\%}$ as a Col_h structure.

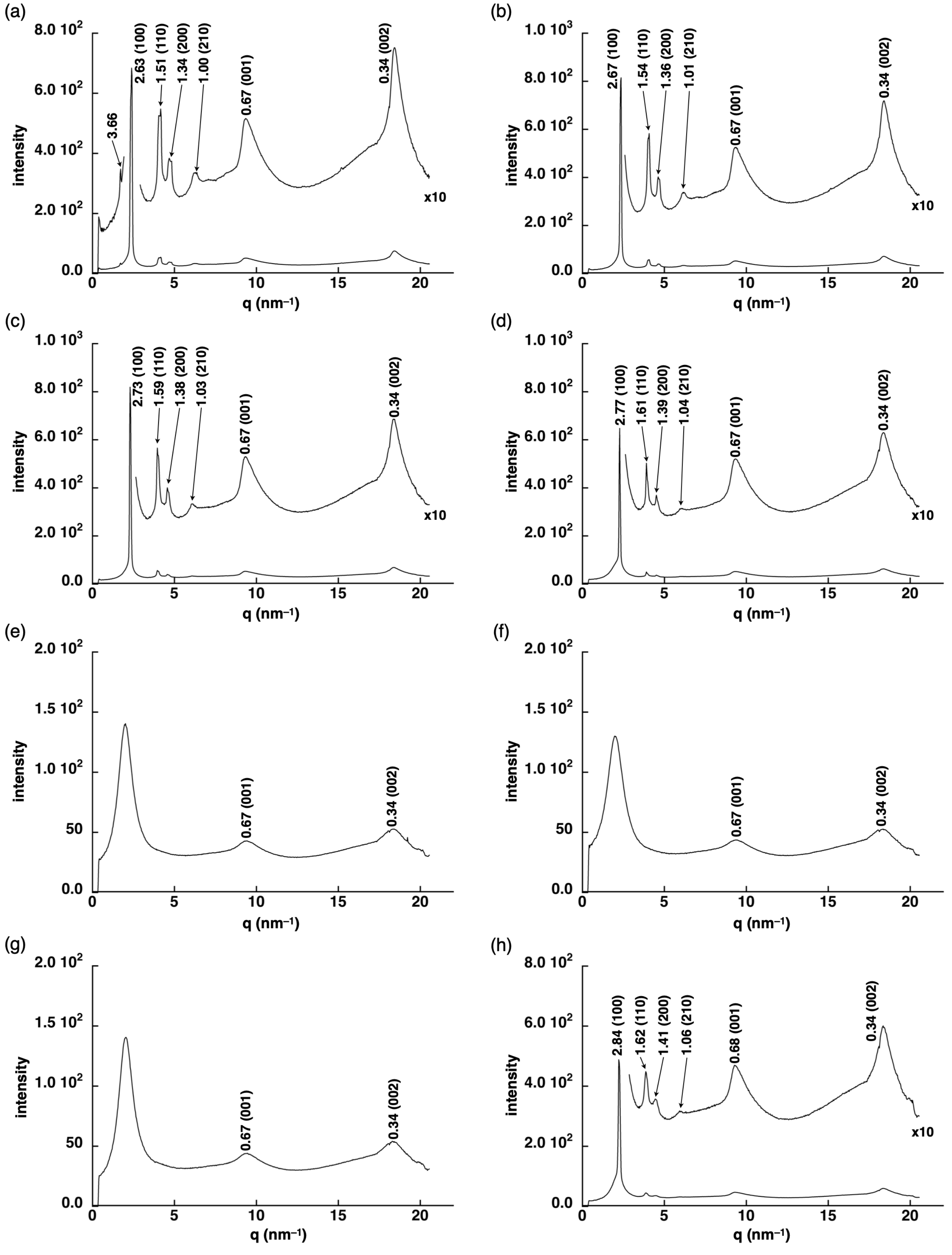

Figure S117 XRD patterns of **2au**$^{+}$-PCCp$^{-}{}_{60\%}$ at (a) 25 °C, (b) 40 °C, (c) 50 °C, (d) 60 °C, (e) 70 °C, (f) 80 °C, (g) 70 °C, (h) 60 °C, (i) 50 °C, (j) 20 °C, (k) 5 °C, (l) 20 °C, (m) 50 °C, (n) 60 °C, (o) 70 °C, and (p) 80 °C upon (a–f) 1st heating, (g–k) 1st cooling, and (l–p) 2nd heating.

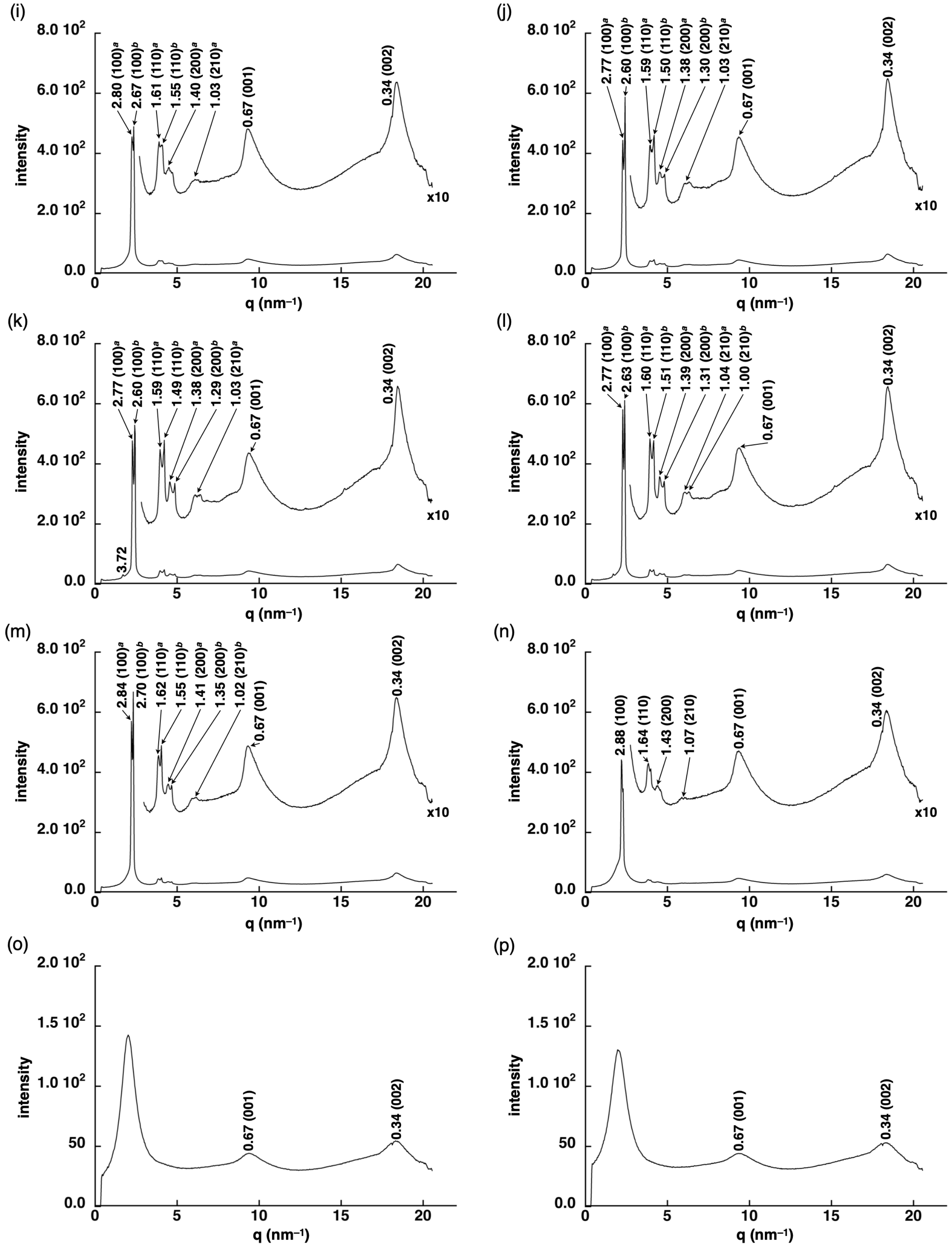

Figure S117 (Continued)

Table S19 Summary of XRD data of **2au**$^{+}$-PCCp$^{-}_{60\%}$. The peaks which can be indexed are represented.

	q (nm^{-1})	*d*-spacing (nm)	ratio	ratio (calc.)	*hkl*
	2.39	2.63	1.000	1.0000	100
(a) 25 °C (1st heating)	4.16	1.51	0.574	0.5774	110
$\mathrm{Col_h}$	4.68	1.34	0.510	0.5000	200
a = 3.04 nm, c = 0.67 nm	6.28	1.00	0.380	0.3780	210
Z = 1 (ρ = 0.81)	9.33	0.67	–	–	001
	18.4	0.34	–	–	002
	2.36	2.67	1.000	1.0000	100
(b) 40 °C (1st heating)	4.07	1.54	0.579	0.5774	110
$\mathrm{Col_h}$	4.62	1.36	0.510	0.5000	200
a = 3.08 nm, c = 0.67 nm	6.19	1.01	0.381	0.3780	210
Z = 1 (ρ = 0.79)	9.36	0.67	–	–	001
	18.4	0.34	–	–	002
	2.30	2.73	1.000	1.0000	100
(c) 50 °C (1st heating)	3.96	1.59	0.581	0.5774	110
$\mathrm{Col_h}$	4.57	1.38	0.503	0.5000	200
a = 3.16 nm, c = 0.67 nm	6.11	1.03	0.376	0.3780	210
Z = 1 (ρ = 0.75)	9.33	0.67	–	–	001
	18.3	0.34	–	–	002
	2.27	2.77	1.000	1.0000	100
(d) 60 °C (1st heating)	3.90	1.61	0.582	0.5774	110
$\mathrm{Col_h}$	4.51	1.39	0.503	0.5000	200
a = 3.20 nm, c = 0.67 nm	6.02	1.04	0.377	0.3780	210
Z = 1 (ρ = 0.73)	9.36	0.67	–	–	001
	18.3	0.34	–	–	002
	2.21	2.84	1.000	1.0000	100
(h) 60 °C (1st cooling)	3.87	1.62	0.572	0.5774	110
$\mathrm{Col_h}$	4.45	1.41	0.497	0.5000	200
a = 3.28 nm, c = 0.68 nm	5.93	1.06	0.373	0.3780	210
Z = 1 (ρ = 0.69)	9.27	0.68	–	–	001
	18.3	0.34	–	–	002
(i) 50 °C (1st cooling)	2.24[a]	2.80	1.000	1.0000	100
[a]$\mathrm{Col_h}$	2.36[b]	2.67	1.000	1.0000	100
a = 3.24 nm, c = 0.67 nm	3.90[a]	1.61	0.575	0.5774	110
Z = 1 (ρ = 0.71)	4.04[b]	1.55	0.554	0.5774	110
[b]$\mathrm{Col_h}$	4.48[a]	1.40	0.500	0.5000	200
a = 3.08 nm, c = 0.67 nm	6.08[a]	1.03	0.369	0.3780	210
Z = 1 (ρ = 0.79)	9.33	0.67	–	–	001
	18.4	0.34	–	–	002
	2.27[a]	2.77	1.000	1.0000	100
(j) 20 °C (1st cooling)	2.42[b]	2.60	1.000	1.0000	100
[a]$\mathrm{Col_h}$	3.96[a]	1.59	0.574	0.5774	110
a = 3.20 nm, c = 0.67 nm	4.19[b]	1.50	0.577	0.5774	110
Z = 1 (ρ = 0.73)	4.54[a]	1.38	0.500	0.5000	200
[b]$\mathrm{Col_h}$	4.83[b]	1.30	0.500	0.5000	200
a = 3.00 nm, c = 0.67 nm	6.11[a]	1.03	0.372	0.3780	210
Z = 1 (ρ = 0.83)	9.36	0.67	–	–	001
	18.4	0.34	–	–	002

Table S19 (Continued)

	q (nm^{-1})	*d*-spacing (nm)	ratio	ratio (calc.)	*hkl*
	2.27^{a}	2.77	1.000	1.0000	100
(k) 5 °C (1st cooling)	2.42^{b}	2.60	1.000	1.0000	100
^{a}Col$_h$	3.96^{a}	1.59	0.574	0.5774	110
a = 3.20 nm, c = 0.67 nm	4.22^{b}	1.49	0.573	0.5774	110
Z = 1 (ρ = 0.73)	4.54^{a}	1.38	0.500	0.5000	200
^{b}Col$_h$	4.86^{b}	1.29	0.497	0.5000	200
a = 3.00 nm, c = 0.67 nm	6.11^{a}	1.03	0.372	0.3780	210
Z = 1 (ρ = 0.83)	9.36	0.67	–	–	001
	18.4	0.34	–	–	002
	2.27^{a}	2.77	1.000	1.0000	100
	2.39^{b}	2.63	1.000	1.0000	100
(l) 20 °C (2nd heating)	3.93^{a}	1.60	0.578	0.5774	110
^{a}Col$_h$	4.16^{b}	1.51	0.574	0.5774	110
a = 3.20 nm, c = 0.67 nm	4.51^{a}	1.39	0.503	0.5000	200
Z = 1 (ρ = 0.73)	4.80^{b}	1.31	0.497	0.5000	200
^{b}Col$_h$	6.05^{a}	1.04	0.375	0.3780	210
a = 3.04 nm, c = 0.67 nm	6.31^{b}	1.00	0.378	0.3780	210
Z = 1 (ρ = 0.81)	9.39	0.67	–	–	001
	18.4	0.34	–	–	002
	2.21^{a}	2.84	1.000	1.0000	100
(m) 50 °C (2nd heating)	2.33^{b}	2.70	1.000	1.0000	100
^{a}Col$_h$	3.87^{a}	1.62	0.572	0.5774	110
a = 3.28 nm, c = 0.67 nm	4.04^{b}	1.55	0.576	0.5774	110
Z = 1 (ρ = 0.69)	4.45^{a}	1.41	0.497	0.5000	200
^{b}Col$_h$	4.65^{b}	1.35	0.500	0.5000	200
a = 3.12 nm, c = 0.67 nm	6.16^{b}	1.02	0.378	0.3780	210
Z = 1 (ρ = 0.77)	9.33	0.67	–	–	001
	18.4	0.34	–	–	002
	2.18	2.88	1.000	1.0000	100
(n) 60 °C (2nd heating)	3.84	1.64	0.568	0.5774	110
Col$_h$	4.39	1.43	0.497	0.5000	200
a = 3.32 nm, c = 0.67 nm	5.87	1.07	0.372	0.3780	210
Z = 1 (ρ = 0.67)	9.33	0.67	–	–	001
	18.3	0.34	–	–	002

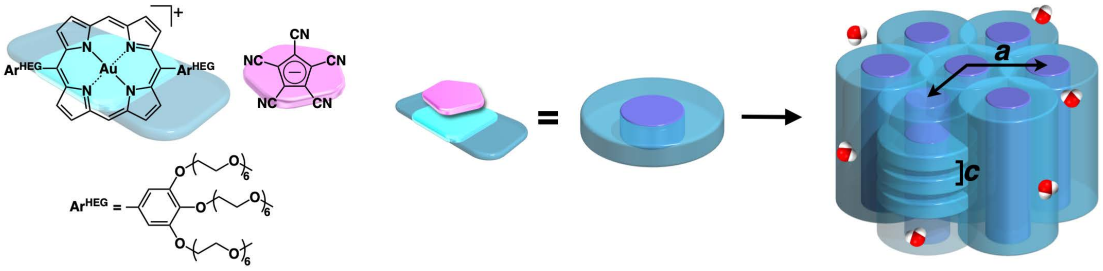

Figure S118 Possible packing model of **2au**$^{+}$-PCCp$^{-}_{60\%}$ as a Col$_h$ structure.

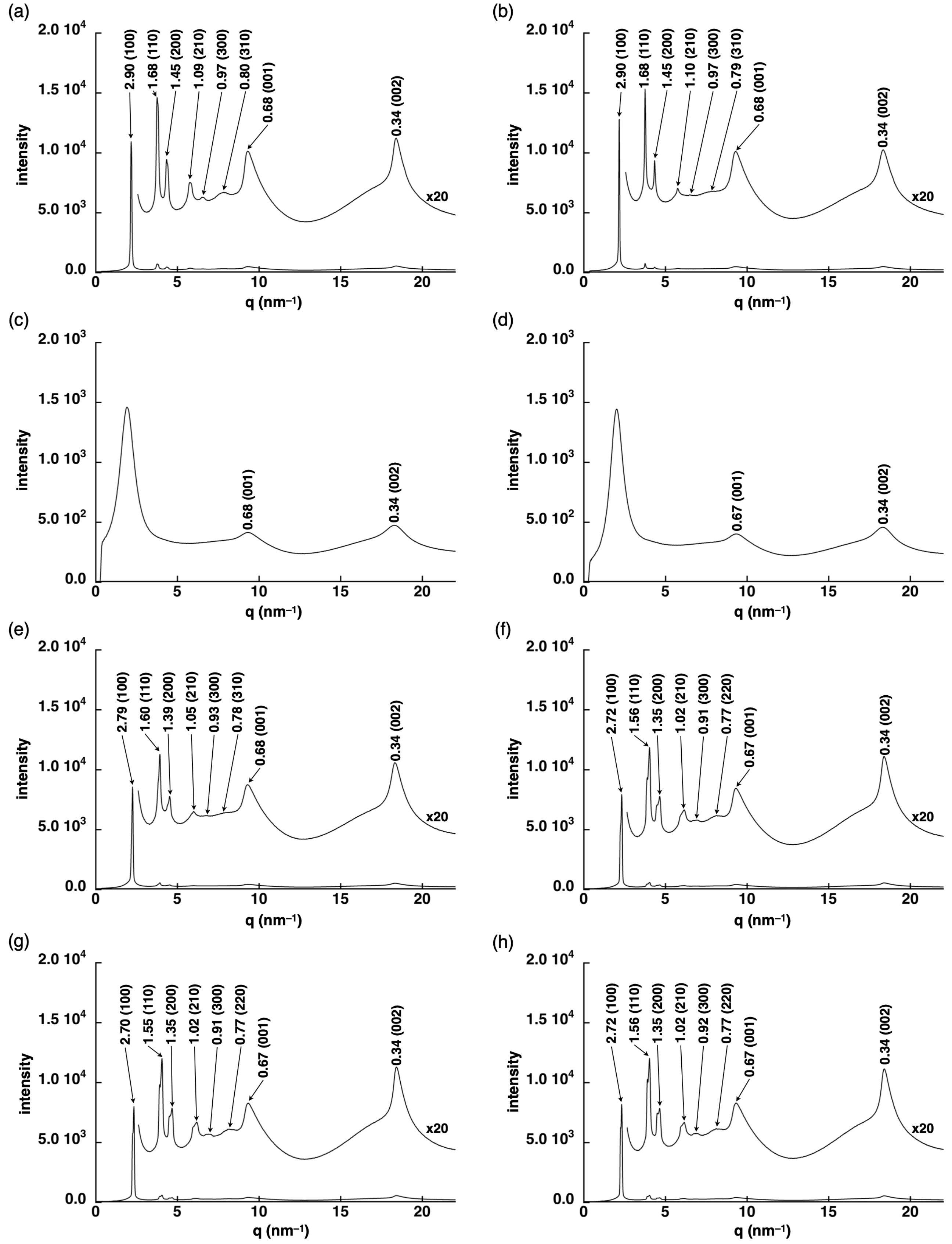

Figure S119 XRD patterns of **2au**$^+$-PCCp$^-_{50\%}$ at (a) 25 °C, (b) 60 °C, (c) 80 °C, (d) 70 °C, (e) 60 °C, (f) 20 °C, (g) 5 °C, (h) 20 °C, (i) 60 °C, and (j) 80 °C upon (a–c) 1st heating, (d–g) 1st cooling, and (h–j) 2nd heating.

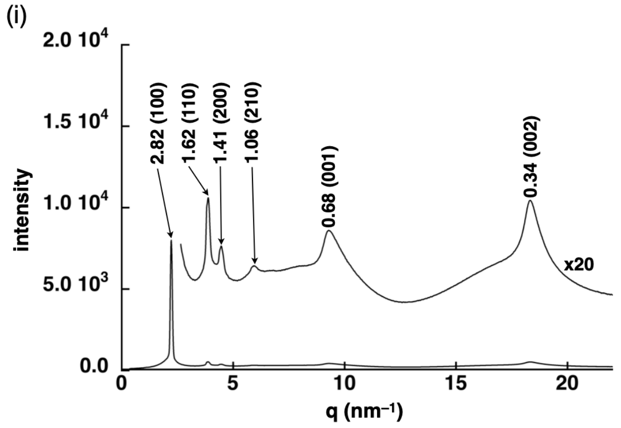

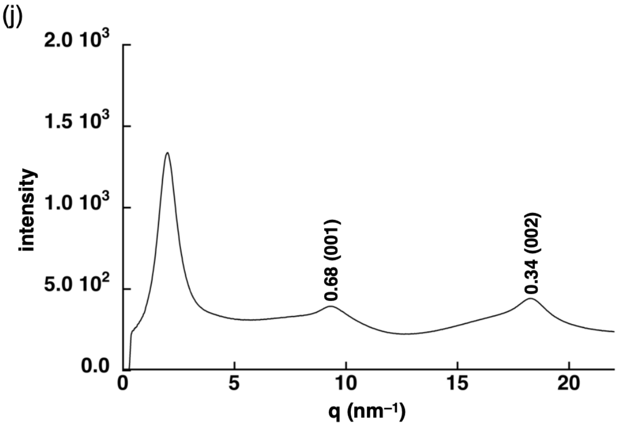

Figure S119 (Continued)

Table S20 Summary of XRD data of **2au**$^+$-PCCp$^-$$_{50\%}$. The peaks which can be indexed are represented.

	q (nm^{-1})	*d*-spacing (nm)	ratio	ratio (calc.)	*hkl*
	2.17	2.90	1.000	1.0000	100
	3.75	1.68	0.577	0.5774	110
(a) 25 °C (1st heating)	4.33	1.45	0.500	0.5000	200
Col$_h$	5.75	1.09	0.377	0.3780	210
a = 3.35 nm, c = 0.68 nm	6.50	0.97	0.333	0.3333	300
Z = 1 (ρ = 0.66)	7.83	0.80	0.277	0.2774	310
	9.29	0.68	–	–	001
	18.4	0.34	–	–	002
	2.17	2.90	1.000	1.0000	100
	3.75	1.68	0.577	0.5774	110
(b) 60 °C (1st heating)	4.33	1.45	0.500	0.5000	200
Col$_h$	5.74	1.10	0.377	0.3780	210
a = 3.35 nm, c = 0.68 nm	6.47	0.97	0.335	0.3333	300
Z = 1 (ρ = 0.66)	7.96	0.79	0.272	0.2774	310
	9.29	0.68	–	–	001
	18.3	0.34	–	–	002
	2.25	2.79	1.000	1.0000	100
	3.92	1.60	0.574	0.5774	110
(e) 60 °C (1st cooling)	4.52	1.39	0.499	0.5000	200
Col$_h$	5.99	1.05	0.376	0.3780	210
a = 3.22 nm, c = 0.68 nm	6.79	0.93	0.332	0.3333	300
Z = 1 (ρ = 0.71)	8.10	0.78	0.278	0.2774	310
	9.28	0.68	–	–	001
	18.3	0.34	–	–	002
	2.31	2.72	1.000	1.0000	100
	4.02	1.56	0.575	0.5774	110
(f) 20 °C (1st cooling)	4.65	1.35	0.497	0.5000	200
Col$_h$	6.14	1.02	0.377	0.3780	210
a = 3.14 nm, c = 0.67 nm	6.89	0.91	0.336	0.3333	300
Z = 1 (ρ = 0.76)	8.11	0.77	0.285	0.2887	220
	9.34	0.67	–	–	001
	18.4	0.34	–	–	002

Table S20 (Continued)

	q (nm^{-1})	*d*-spacing (nm)	ratio	ratio (calc.)	*hkl*
(g) 5 °C (1st cooling)<br>Col$_h$<br>a = 3.12 nm, c = 0.67 nm<br>Z = 1 (ρ = 0.77)	2.33	2.70	1.000	1.0000	100
	4.05	1.55	0.575	0.5774	110
	4.66	1.35	0.499	0.5000	200
	6.18	1.02	0.377	0.3780	210
	6.89	0.91	0.338	0.3333	300
	8.11	0.77	0.287	0.2887	220
	9.34	0.67	–	–	001
	18.4	0.34	–	–	002
(h) 20 °C (2nd heating)<br>Col$_h$<br>a = 3.14 nm, c = 0.67 nm<br>Z = 1 (ρ = 0.76)	2.31	2.72	1.000	1.0000	100
	4.02	1.56	0.575	0.5774	110
	4.64	1.35	0.499	0.5000	200
	6.14	1.02	0.377	0.3780	210
	6.85	0.92	0.338	0.3333	300
	8.14	0.77	0.284	0.2887	220
	9.34	0.67	–	–	001
	18.4	0.34	–	–	002
(i) 60 °C (2nd heating)<br>Col$_h$<br>a = 3.26 nm, c = 0.68 nm<br>Z = 1 (ρ = 0.70)	2.23	2.82	1.000	1.0000	100
	3.89	1.62	0.573	0.5774	110
	4.47	1.41	0.499	0.5000	200
	5.94	1.06	0.375	0.3780	210
	9.29	0.68	–	–	001
	18.3	0.34	–	–	002

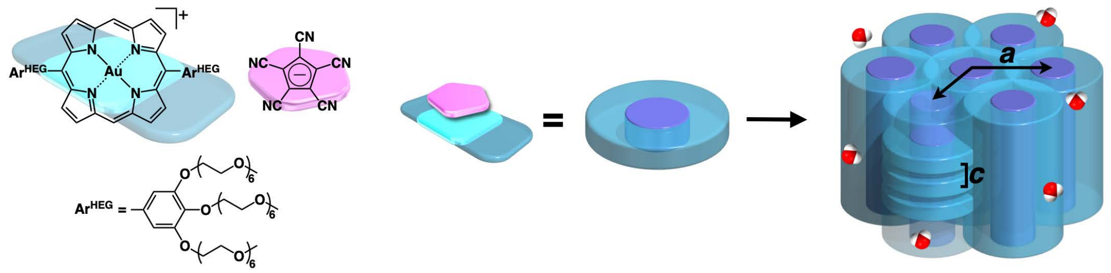

Figure S120 Possible packing model of **2au**$^{+}$-PCCp$^{-}_{50\%}$ as a Col$_h$ structure.

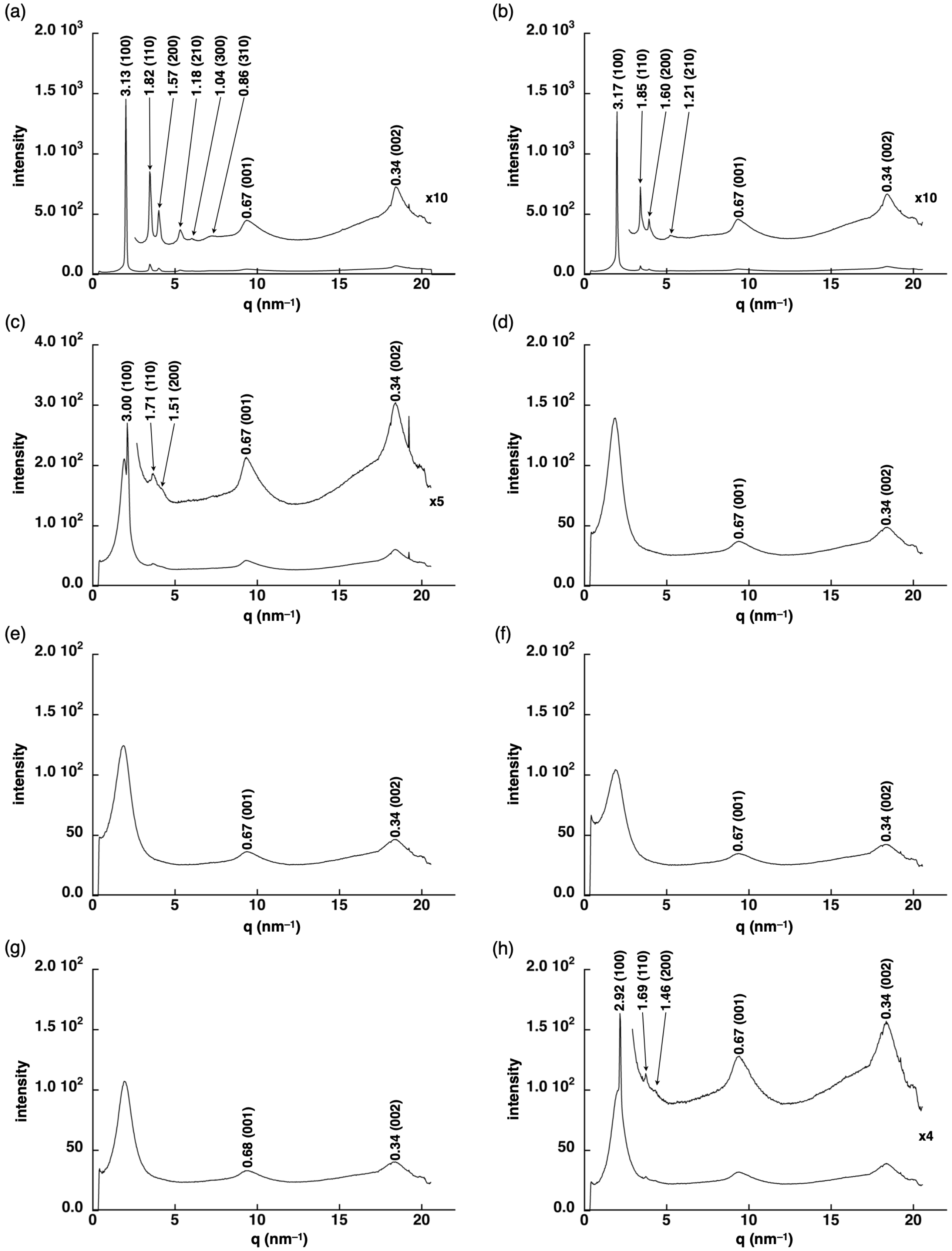

Figure S121 XRD patterns of **2au**$^+$-PCCp$^-_{40\%}$ at (a) 25 °C, (b) 50 °C, (c) 60 °C, (d) 65 °C, (e) 70 °C, (f) 80 °C, (g) 70 °C, (h) 65 °C, (i) 60 °C, (j) 55 °C, (k) 50 °C, (l) 20 °C, (m) 5 °C, (n) 20 °C, (o) 40 °C, (p) 50 °C, (q) 60 °C, (r) 70 °C, and (s) 80 °C upon (a–f) 1st heating, (g–m) 1st cooling, and (n–s) 2nd heating.

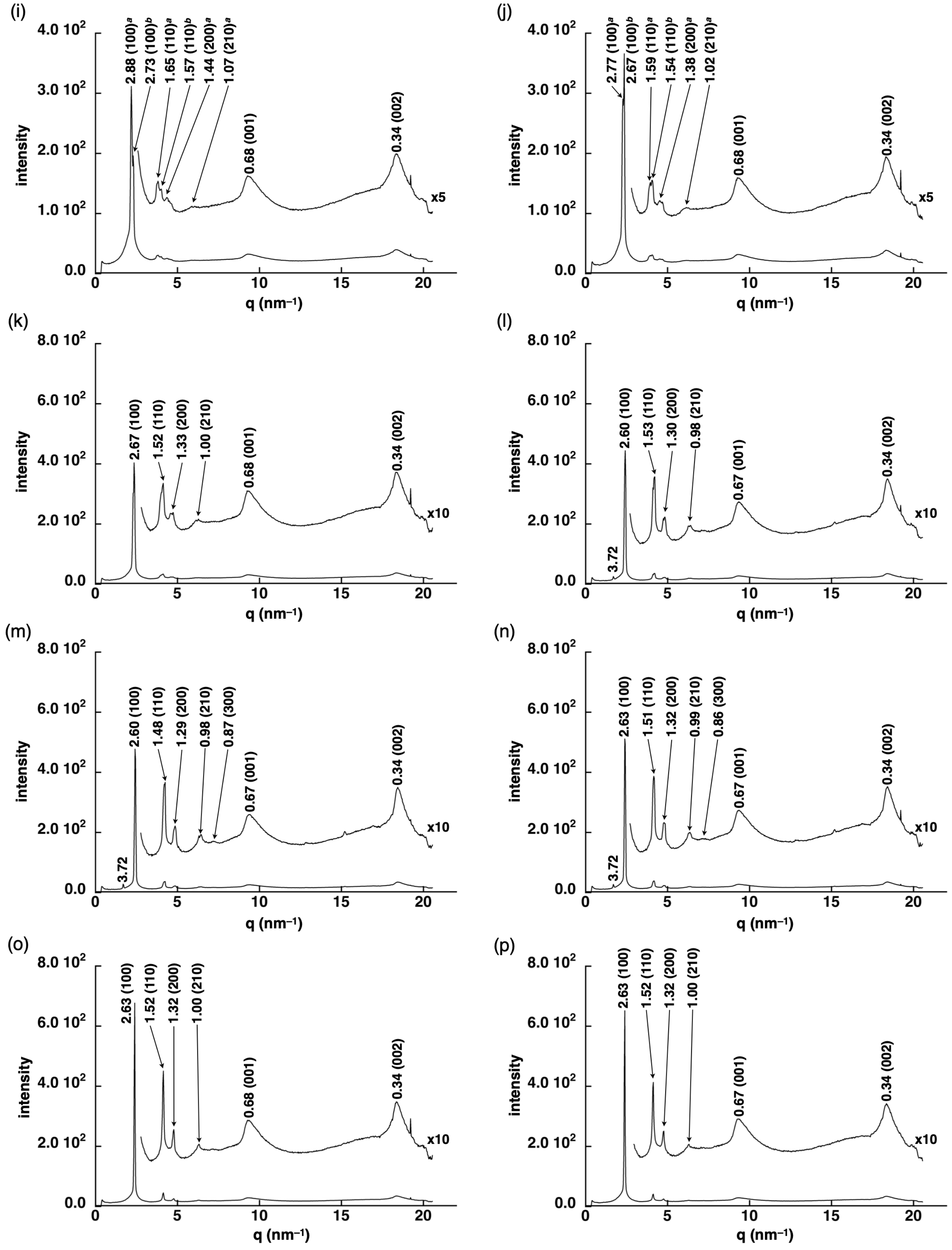

Figure S121 (Continued)

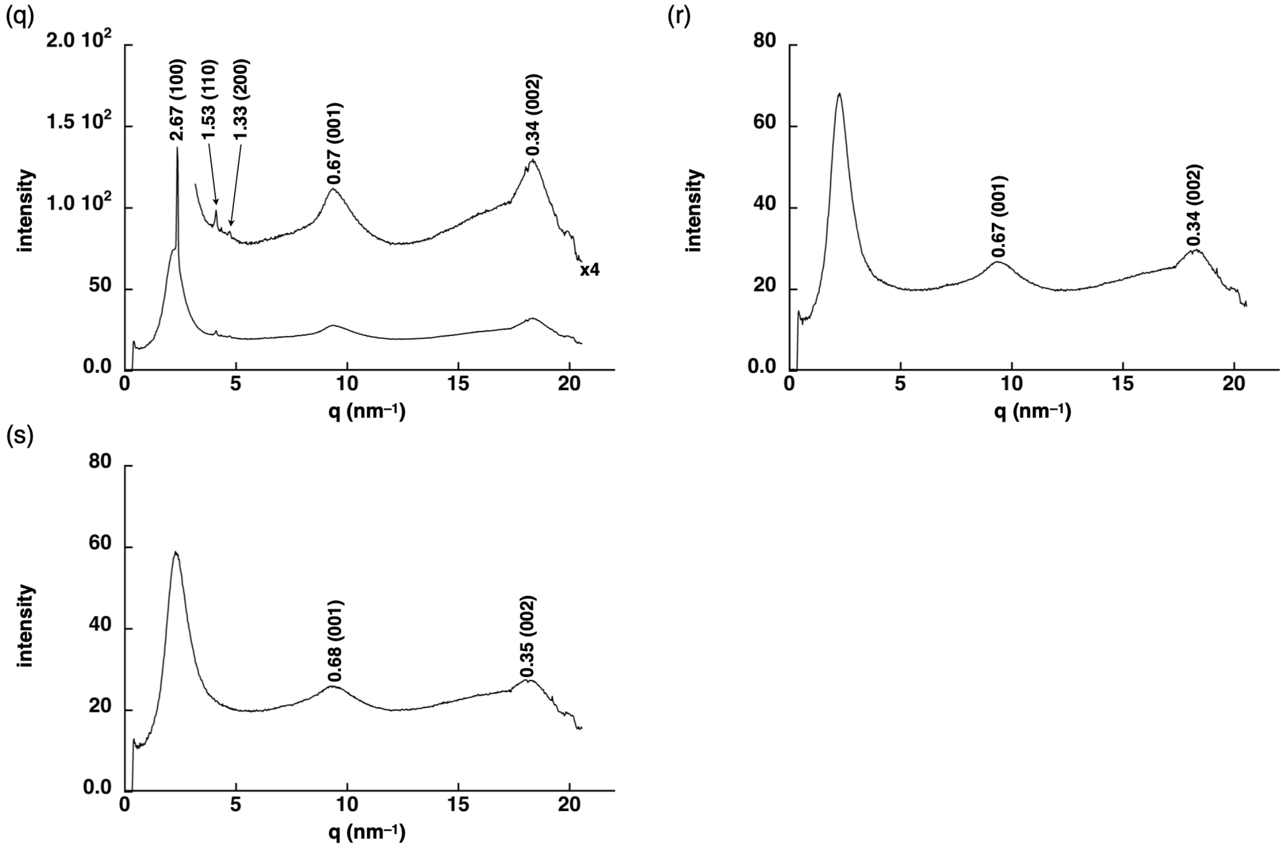

Figure S121 (Continued)

Table S21 Summary of XRD data of **2au**$^{+}$-PCCp$^{-}_{40\%}$. The peaks which can be indexed are represented.

	q (nm^{-1})	*d*-spacing (nm)	ratio	ratio (calc.)	*hkl*
	2.01	3.13	1.000	1.0000	100
	3.46	1.82	0.580	0.5774	110
(a) 25 °C (1st heating)	4.01	1.57	0.500	0.5000	200
Col$_h$	5.32	1.18	0.377	0.3780	210
a = 3.61 nm, c = 0.67 nm	6.02	1.04	0.334	0.3333	300
Z = 1 (ρ = 0.57)	7.30	0.86	0.275	0.2774	310
	9.33	0.67	–	–	001
	18.4	0.34	–	–	002
	1.98	3.17	1.000	1.0000	100
(b) 50 °C (1st heating)	3.40	1.85	0.581	0.5774	110
Col$_h$	3.93	1.60	0.504	0.5000	200
a = 3.67 nm, c = 0.67 nm	5.21	1.21	0.380	0.3780	210
Z = 1 (ρ = 0.55)	9.33	0.67	–	–	001
	18.4	0.34	–	–	002
(c) 60 °C (1st heating)	2.10	3.00	1.000	1.0000	100
Col$_h$	3.67	1.71	0.572	0.5774	110
a = 3.46 nm, c = 0.67 nm	4.16	1.51	0.504	0.5000	200
Z = 1 (ρ = 0.62)	9.33	0.67	–	–	001
	18.4	0.34	–	–	002
(h) 65 °C (1st cooling)	2.15	2.92	1.000	1.0000	100
Col$_h$	3.72	1.69	0.578	0.5774	110
a = 3.37 nm, c = 0.67 nm	4.30	1.46	0.500	0.5000	200
Z = 1 (ρ = 0.66)	9.42	0.67	–	–	001
	18.3	0.34	–	–	002

Table S21 (Continued)

	q (nm^{-1})	*d*-spacing (nm)	ratio	ratio (calc.)	*hkl*
(i) 60 °C (1st cooling)	2.18[a]	2.88	1.000	1.0000	100
[a]Col$_{h}$	2.30[b]	2.73	1.000	1.0000	100
a = 3.32 nm, c = 0.68 nm	3.81[a]	1.65	0.573	0.5774	110
Z = 1 (ρ = 0.67)	4.01[b]	1.57	0.573	0.5774	110
[b]Col$_{h}$	4.36[a]	1.44	0.500	0.5000	200
a = 3.16 nm, c = 0.68 nm	5.87[a]	1.07	0.372	0.3780	210
Z = 1 (ρ = 0.74)	9.27	0.68	–	–	001
	18.3	0.34	–	–	002
(j) 55 °C (1st cooling)	2.27[a]	2.77	1.000	1.0000	100
[a]Col$_{h}$	2.36[b]	2.67	1.000	1.0000	100
a = 3.20 nm, c = 0.68 nm	3.96[a]	1.59	0.574	0.5774	110
Z = 1 (ρ = 0.72)	4.07[b]	1.54	0.579	0.5774	110
[b]Col$_{h}$	4.54[a]	1.38	0.500	0.5000	200
a = 3.08 nm, c = 0.68 nm	6.14[a]	1.02	0.370	0.3780	210
Z = 1 (ρ = 0.78)	9.27	0.68	–	–	001
	18.3	0.34	–	–	002
	2.36	2.67	1.000	1.0000	100
(k) 50 °C (1st cooling)	4.13	1.52	0.571	0.5774	110
Col$_{h}$	4.74	1.33	0.497	0.5000	200
a = 3.08 nm, c = 0.68 nm	6.28	1.00	0.375	0.3780	210
Z = 1 (ρ = 0.78)	9.24	0.68	–	–	001
	18.3	0.34	–	–	002
	2.42	2.60	1.000	1.0000	100
(l) 20 °C (1st cooling)	4.10	1.53	0.589	0.5774	110
Col$_{h}$	4.83	1.30	0.500	0.5000	200
a = 3.00 nm, c = 0.67 nm	6.40	0.98	0.378	0.3780	210
Z = 1 (ρ = 0.83)	9.33	0.67	–	–	001
	18.4	0.34	–	–	002
	2.42	2.60	1.000	1.0000	100
(m) 5 °C (1st cooling)	4.25	1.48	0.569	0.5774	110
Col$_{h}$	4.86	1.29	0.497	0.5000	200
a = 3.00 nm, c = 0.67 nm	6.43	0.98	0.376	0.3780	210
Z = 1 (ρ = 0.83)	7.18	0.87	0.336	0.3333	300
	9.42	0.67	–	–	001
	18.4	0.34	–	–	002
	2.39	2.63	1.000	1.0000	100
(n) 20 °C (2nd heating)	4.16	1.51	0.574	0.5774	110
Col$_{h}$	4.77	1.32	0.500	0.5000	200
a = 3.04 nm, c = 0.67 nm	6.37	0.99	0.375	0.3780	210
Z = 1 (ρ = 0.81)	7.30	0.86	0.327	0.3333	300
	9.36	0.67	–	–	001
	18.4	0.34	–	–	002
	2.39	2.63	1.000	1.0000	100
(o) 40 °C (2nd heating)	4.13	1.52	0.578	0.5774	110
Col$_{h}$	4.77	1.32	0.500	0.5000	200
a = 3.04 nm, c = 0.68 nm	6.31	1.00	0.378	0.3780	210
Z = 1 (ρ = 0.80)	9.27	0.68	–	–	001
	18.3	0.34	–	–	002
	2.39	2.63	1.000	1.0000	100
(p) 50 °C (2nd heating)	4.13	1.52	0.578	0.5774	110
Col$_{h}$	4.77	1.32	0.500	0.5000	200
a = 3.04 nm, c = 0.67 nm	6.31	1.00	0.378	0.3780	210
Z = 1 (ρ = 0.81)	9.33	0.67	–	–	001
	18.3	0.34	–	–	002

Table S21 (Continued)

	q (nm^{-1})	*d*-spacing (nm)	ratio	ratio (calc.)	*hkl*
(q) 60 °C (2nd heating) Col_h a = 3.08 nm, c = 0.67 nm Z = 1 (ρ = 0.79)	2.36	2.67	1.000	1.0000	100
	4.10	1.53	0.575	0.5774	110
	4.71	1.33	0.500	0.5000	200
	9.36	0.67	–	–	001
	18.3	0.34	–	–	002

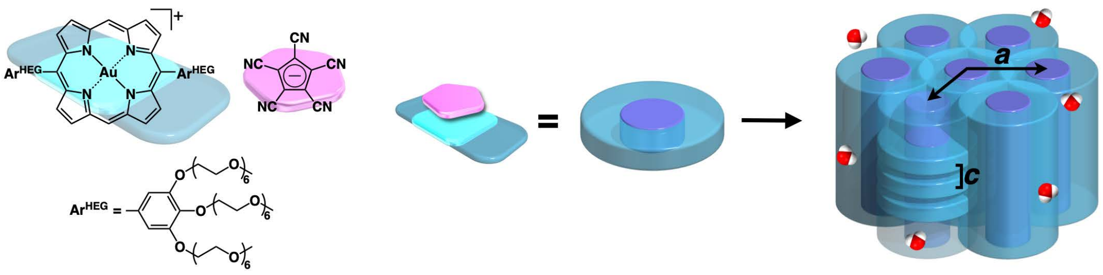

Figure S122 Possible packing model of **2au**$^{+}$-PCCp$^{-}_{40\%}$ as a Col_h structure.

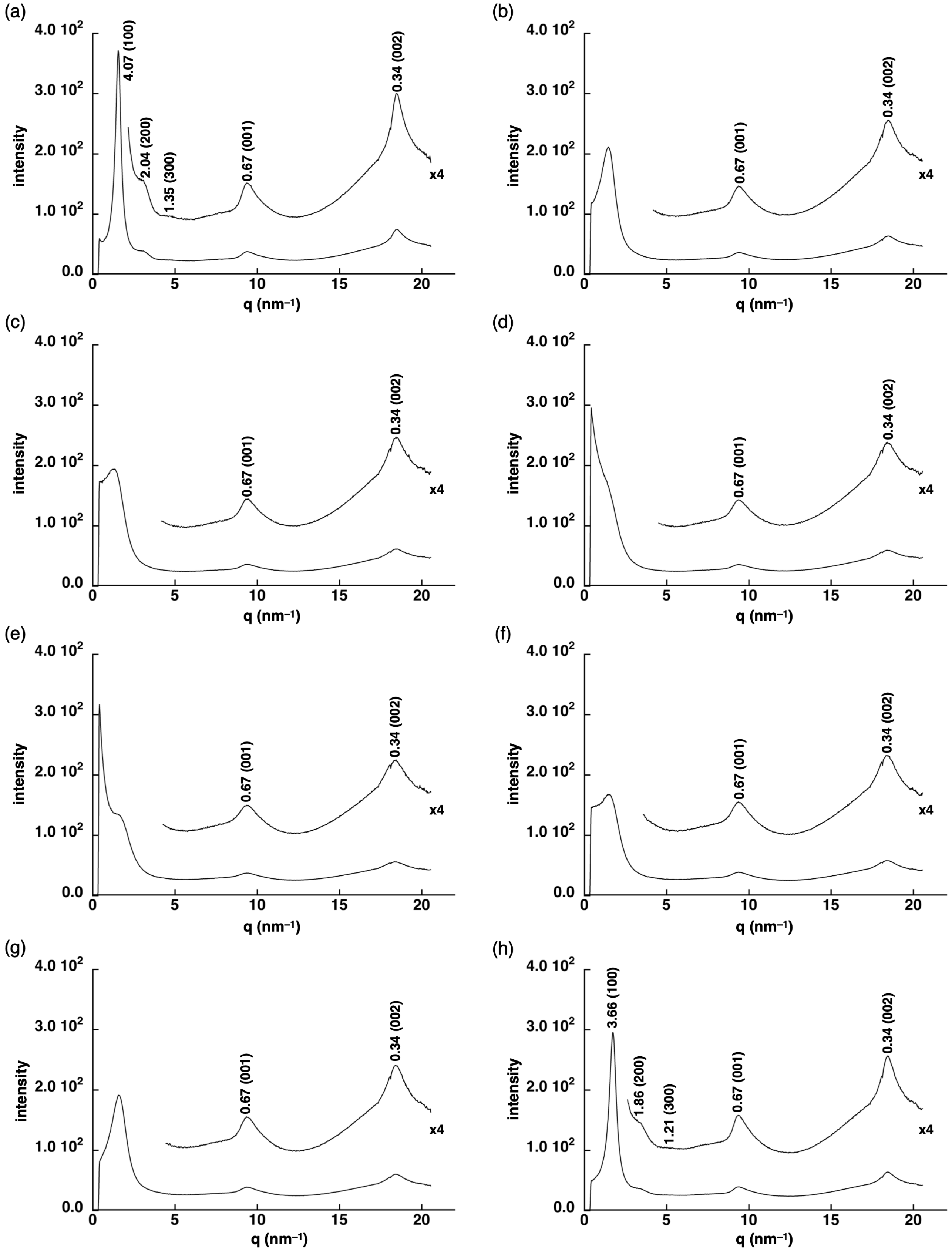

Figure S123 XRD patterns of **2au**$^+$-PCCp$^-$$_{30\%}$ at (a) 25 °C, (b) 50 °C, (c) 60 °C, (d) 70 °C, (e) 80 °C, (f) 70 °C, (g) 60 °C, (h) 50 °C, (i) 40 °C, (j) 30 °C, (k) 20 °C, (l) 5 °C, (m) 20 °C, (n) 40 °C, (o) 50 °C, (p) 60 °C, (q) 70 °C, and (r) 80 °C upon (a–e) 1st heating, (f–l) 1st cooling, and (m–r) 2nd heating.

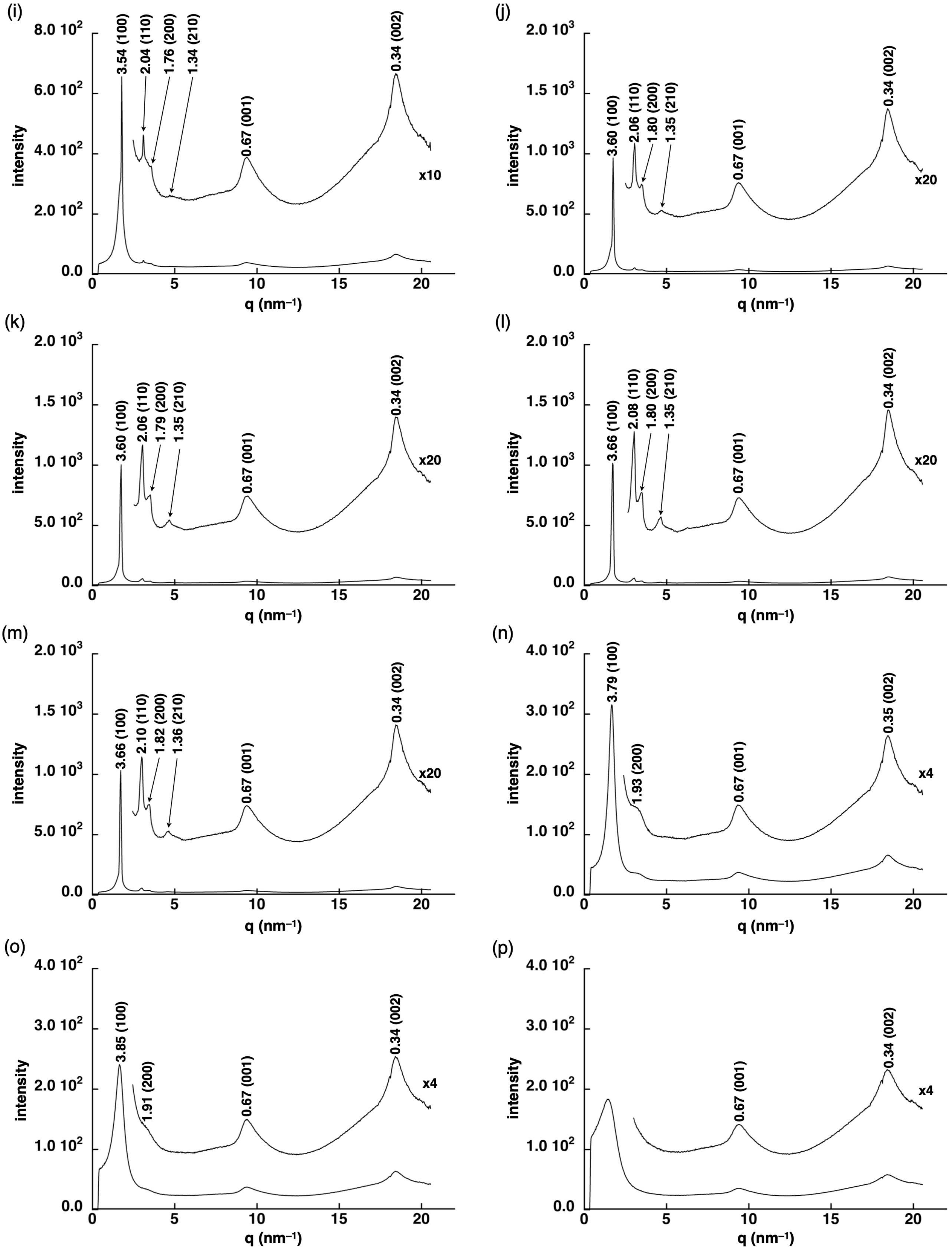

Figure S123 (Continued)

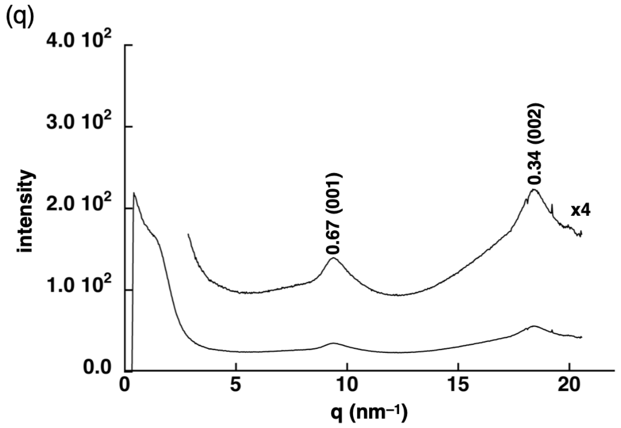

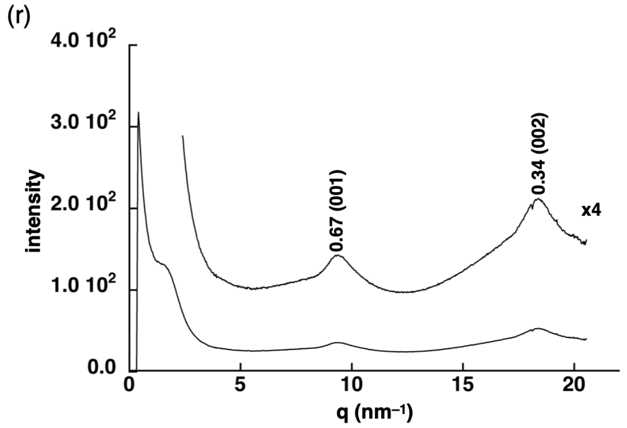

Figure S123 (Continued)

Table S22 Summary of XRD data of **2au**$^{+}$-PCCp$^{-}{}_{30\%}$. The peaks which can be indexed are represented.

	q (nm^{-1})	*d*-spacing (nm)	ratio	ratio (calc.)	*hkl*
	1.54	4.07	1.000	1.0000	100
(a) 25 °C (1st heating)	3.08	2.04	0.500	0.5000	200
Lam$_{col}$	4.65	1.35	0.332	0.3333	300
a = 4.07 nm, c = 0.67 nm	9.36	0.67	–	–	001
	18.5	0.34	–	–	002
	1.72	3.66	1.000	1.0000	100
(h) 50 °C (1st cooling)	3.37	1.86	0.509	0.5000	200
Lam$_{col}$	5.21	1.21	0.330	0.3333	300
a = 3.66 nm, c = 0.67 nm	9.33	0.67	–	–	001
	18.4	0.34	–	–	002
	1.78	3.54	1.000	1.0000	100
(i) 40 °C (1st cooling)	3.08	2.04	0.576	0.5774	110
Col$_{h}$	3.58	1.76	0.496	0.5000	200
a = 4.09 nm, c = 0.67 nm	4.68	1.34	0.379	0.3780	210
Z = 2 (ρ = 0.90)	9.39	0.67	–	–	001
	18.4	0.34	–	–	002
	1.75	3.60	1.000	1.0000	100
(j) 30 °C (1st cooling)	3.05	2.06	0.572	0.5774	110
Col$_{h}$	3.49	1.80	0.500	0.5000	200
a = 4.15 nm, c = 0.67 nm	4.65	1.35	0.375	0.3780	210
Z = 2 (ρ = 0.87)	9.39	0.67	–	–	001
	18.4	0.34	–	–	002
	1.75	3.60	1.000	1.0000	100
(k) 20 °C (1st cooling)	3.05	2.06	0.572	0.5774	110
Col$_{h}$	3.52	1.79	0.496	0.5000	200
a = 4.15 nm, c = 0.67 nm	4.65	1.35	0.375	0.3780	210
Z = 2 (ρ = 0.87)	9.42	0.67	–	–	001
	18.4	0.34	–	–	002
	1.72	3.66	1.000	1.0000	100
(l) 5 °C (1st cooling)	3.03	2.08	0.568	0.5774	110
Col$_{h}$	3.49	1.80	0.492	0.5000	200
a = 4.22 nm, c = 0.67 nm	4.65	1.35	0.369	0.3780	210
Z = 2 (ρ = 0.84)	9.42	0.67	–	–	001
	18.5	0.34	–	–	002

Table S22 (Continued)

	q (nm^{-1})	*d*-spacing (nm)	ratio	ratio (calc.)	*hkl*
	1.72	3.66	1.000	1.0000	100
(m) 20 °C (2nd heating)	3.00	2.10	0.573	0.5774	110
Col$_h$	3.46	1.82	0.496	0.5000	200
a = 4.22 nm, c = 0.67 nm	4.62	1.36	0.371	0.3780	210
Z = 2 (ρ = 0.84)	9.39	0.67	–	–	001
	18.4	0.34	–	–	002
(n) 40 °C (2nd heating)	1.66	3.79	1.000	1.0000	100
Lam$_{col}$	3.26	1.93	0.509	0.5000	200
a = 3.79 nm, c = 0.67 nm	9.33	0.67	–	–	001
	18.1	0.35	–	–	002
(o) 50 °C (2nd heating)	1.63	3.85	1.000	1.0000	100
Lam$_{col}$	3.29	1.91	0.496	0.5000	200
a = 3.85 nm, c = 0.67 nm	9.33	0.67	–	–	001
	18.4	0.34	–	–	002

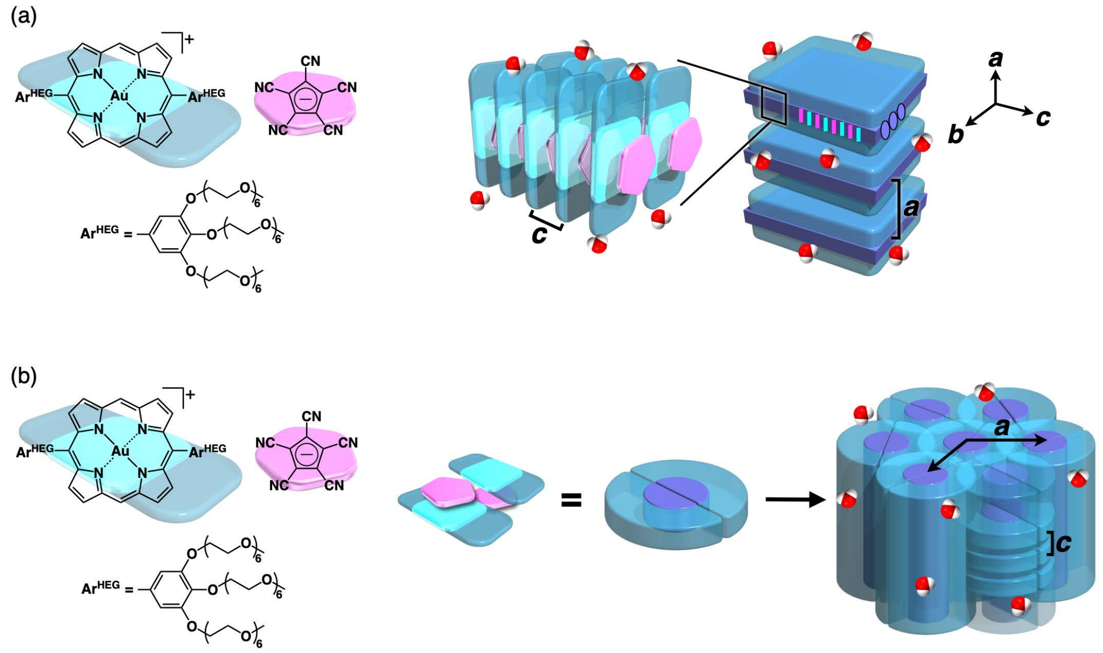

Figure S124 Possible packing models of **2au**$^+$-PCCp$^-_{30\%}$ as (a) a Lam$_{col}$ structure and (b) a Col$_h$ structure.

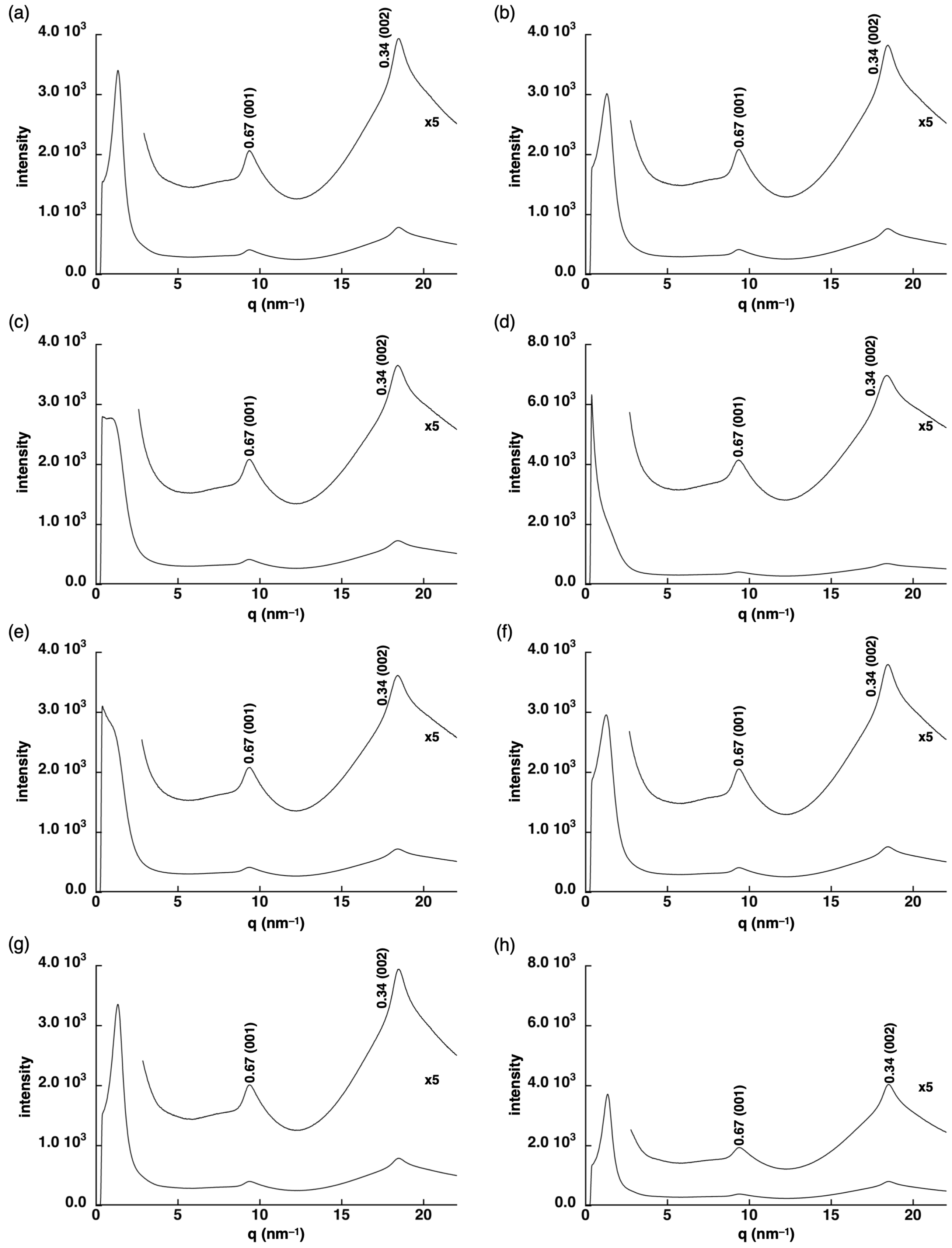

Figure S125 XRD patterns of **2au**$^+$-PCCp$^-_{20\%}$ at (a) 25 °C, (b) 40 °C, (c) 60 °C, (d) 80 °C, (e) 60 °C, (f) 40 °C, (g) 20 °C, (h) 5 °C, (i) 20 °C, (j) 40 °C, (k) 60 °C, and (l) 80 °C upon (a–d) 1st heating, (e–h) 1st cooling, and (i–l) 2nd heating.

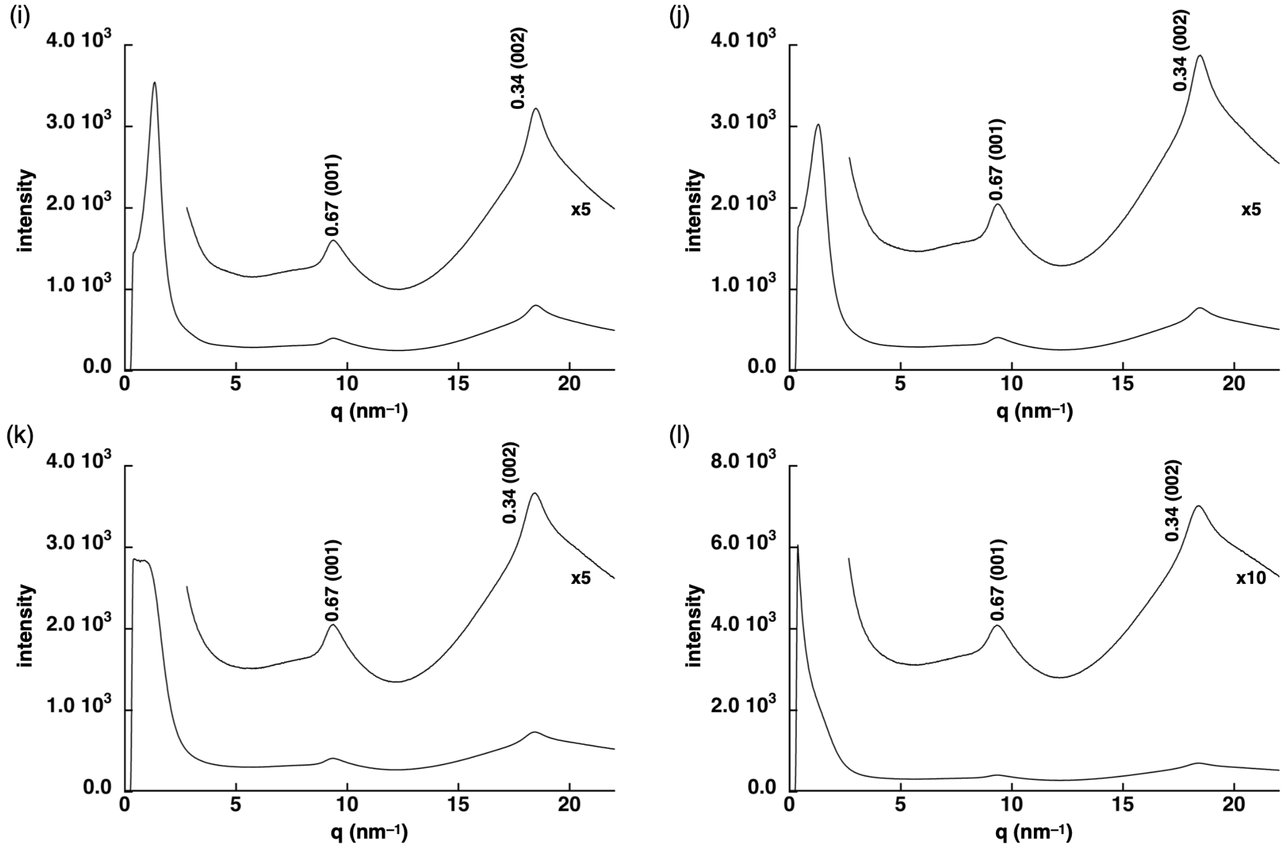

Figure S125 (Continued)

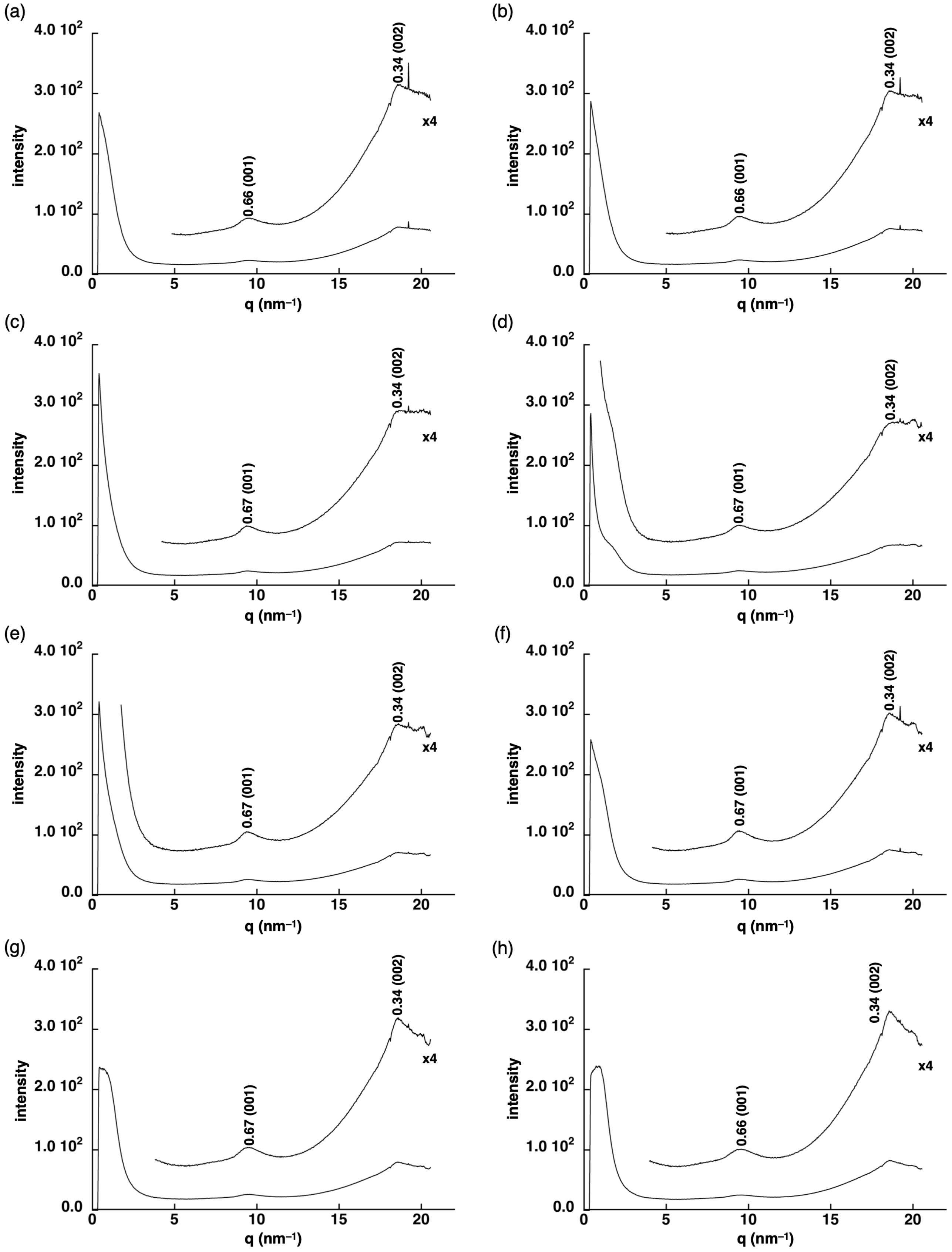

Figure S126 XRD patterns of **2au**$^+$-PCCp$^-_{10\%}$ at (a) 25 °C, (b) 40 °C, (c) 60 °C, (d) 80 °C, (e) 60 °C, (f) 40 °C, (g) 20 °C, (h) 5 °C, (i) 20 °C, (j) 40 °C, (k) 60 °C, and (l) 80 °C upon (a–d) 1st heating, (e–h) 1st cooling, and (i–l) 2nd heating.

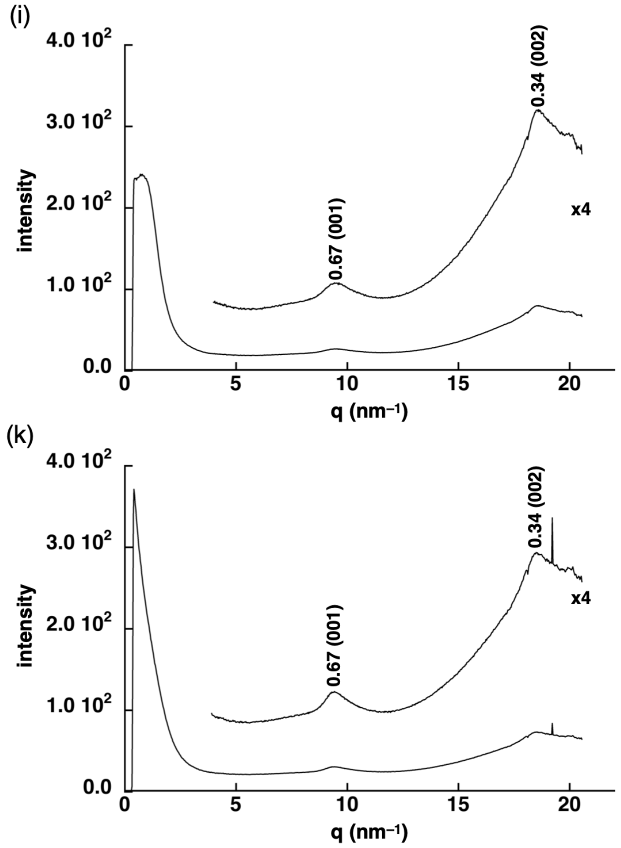

Figure S126 (Continued)

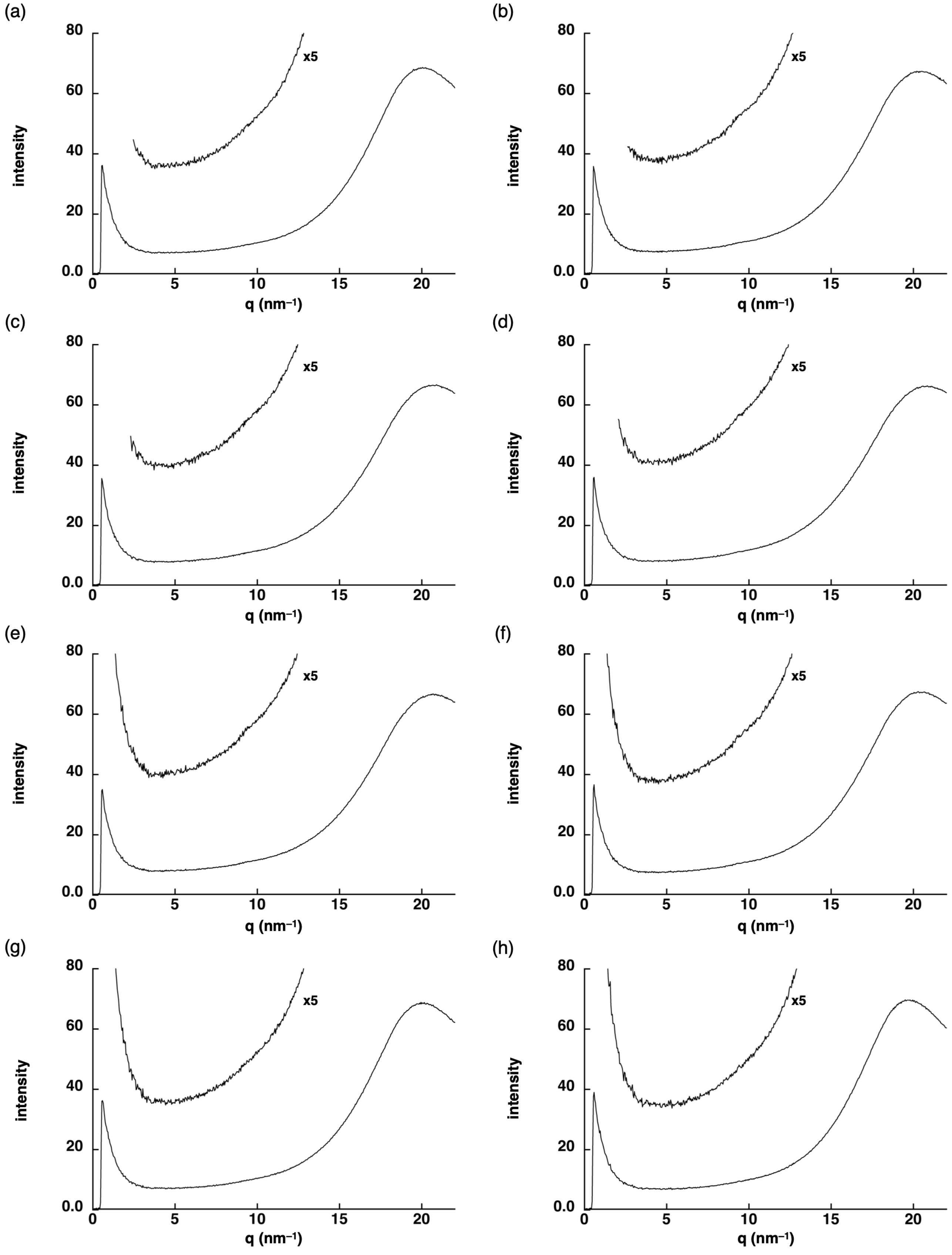

Figure S127 XRD patterns of **2au**$^+$-PCCp$^-_{1\%}$ at (a) 25 °C, (b) 50 °C, (c) 70 °C, (d) 80 °C, (e) 70 °C, (f) 50 °C, (g) 20 °C, (h) 5 °C, (i) 20 °C, (j) 50 °C, (k) 70 °C, and (l) 80 °C upon (a–d) 1st heating, (e–h) 1st cooling, and (i–l) 2nd heating.

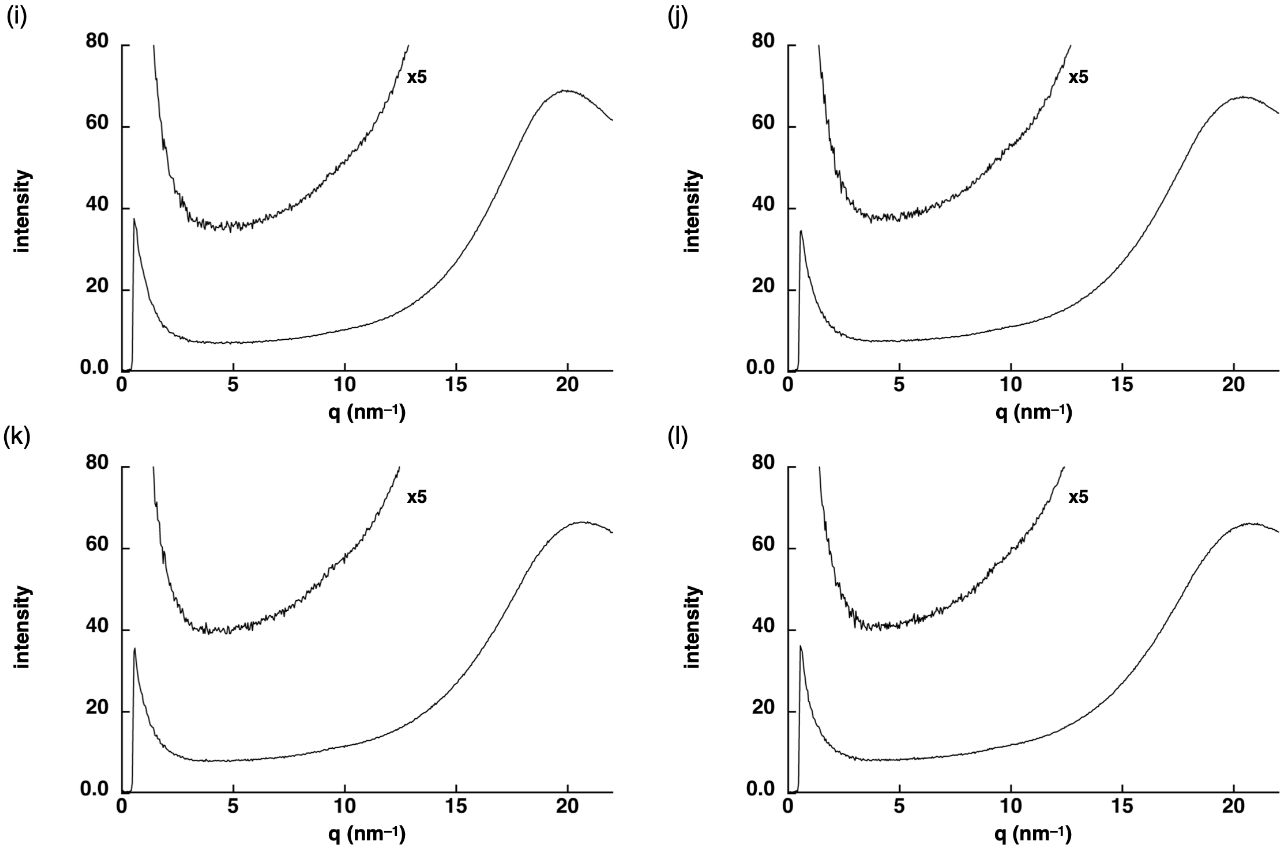

Figure S127 (Continued)

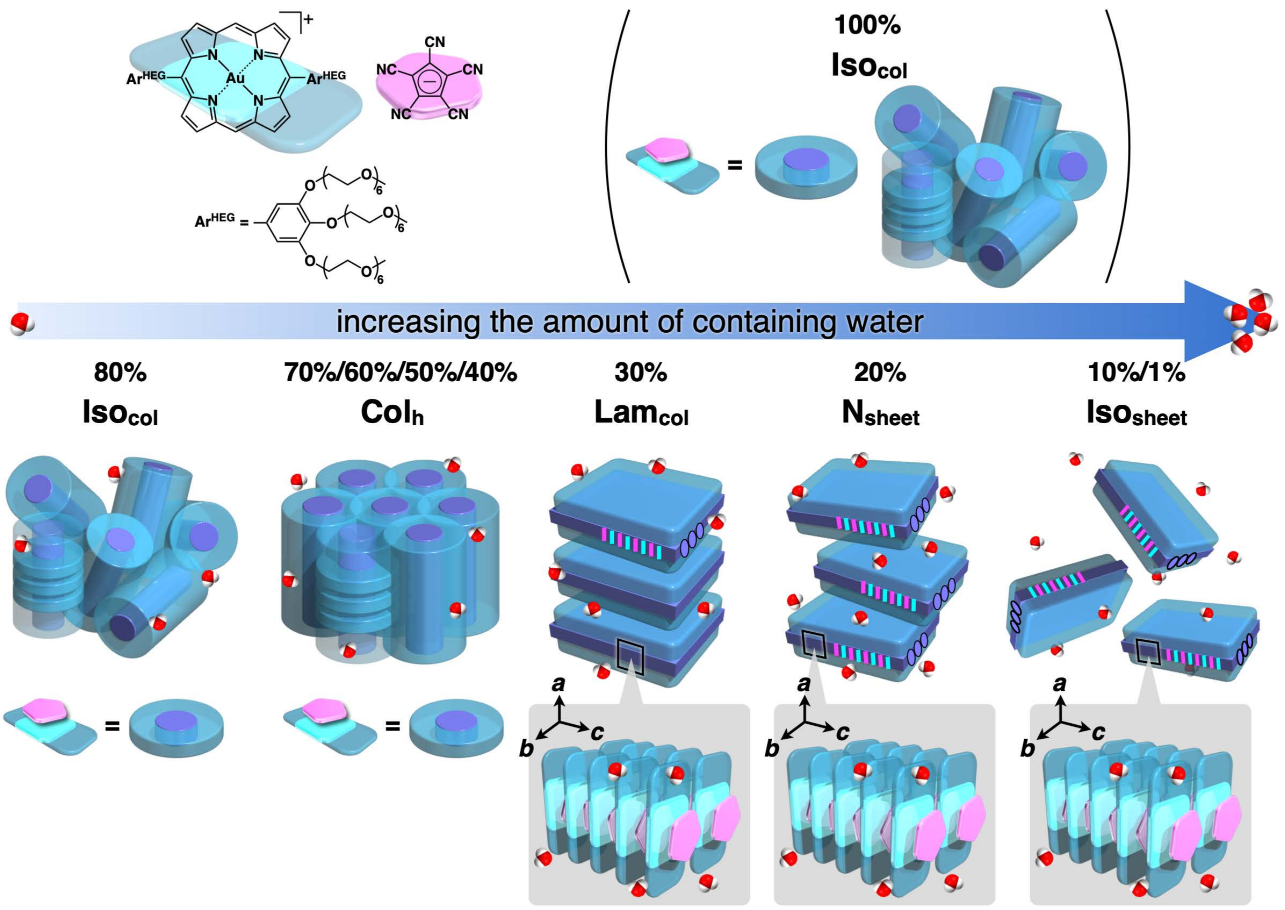

Figure S128 Phase changes of water-containing **2au**$^{+}$-PCCp^{-} according to the ion-pair content at 25 °C.

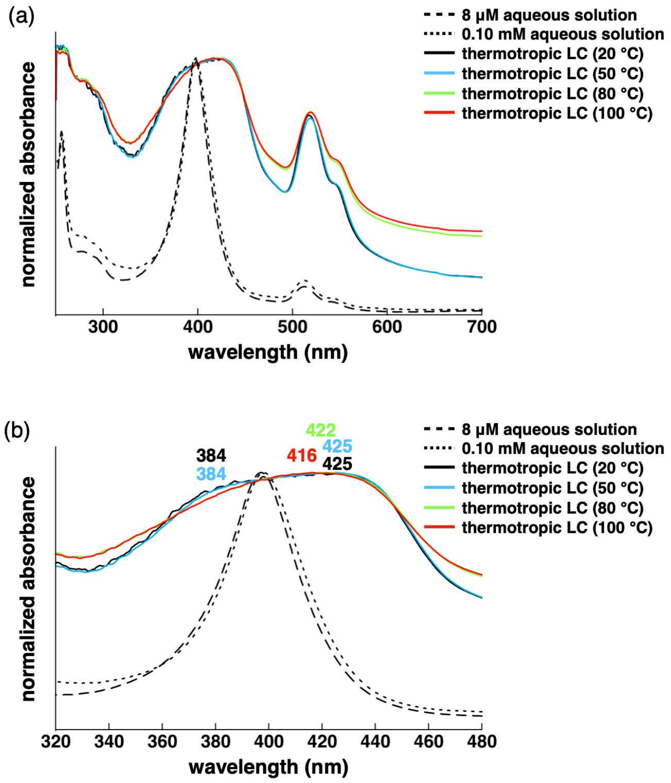

Figure S129 (a) UV/vis absorption spectra of **1au**$^{+}$-PCCp^{-} in aqueous solutions (8 μM: broken line, 0.10 mM: dotted line) and thermotropic liquid crystals (LCs) of **1au**$^{+}$-PCCp^{-} at 20 °C (black solid line), 50 °C (blue), 80 °C (green), and 100 °C (red) and (b) the corresponding enlarged spectra.

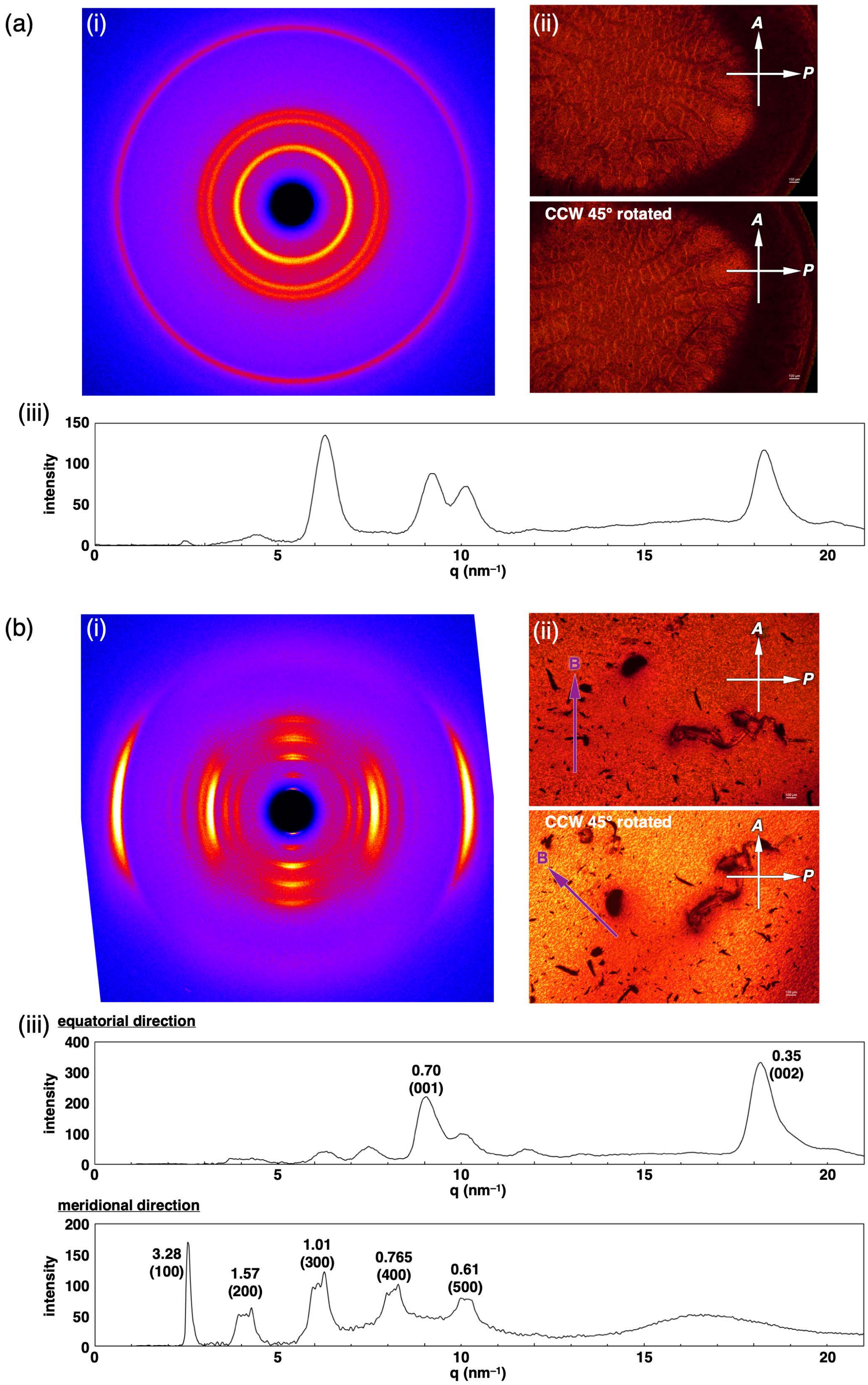

Figure S130 (i) 2D XRD, (ii) POM images, and (iii) corresponding 1D XRD of the samples of **1au**$^{+}$-PCCp^{-} cast on glass substrates, which were prepared by slow vaporization of MeOH from **1au**$^{+}$-PCCp^{-}$_{10\%}$ (a) without a magnetic field, (b) with a 10-T static magnetic field applied along the glass substrate, and (c) with a 10-T magnetic field rotating in-plane of the glass substrate. The images on the right and left were taken at the same region of the sample, whereas the left image was taken after 45° counter-clockwise rotation from the geometry of the right image.

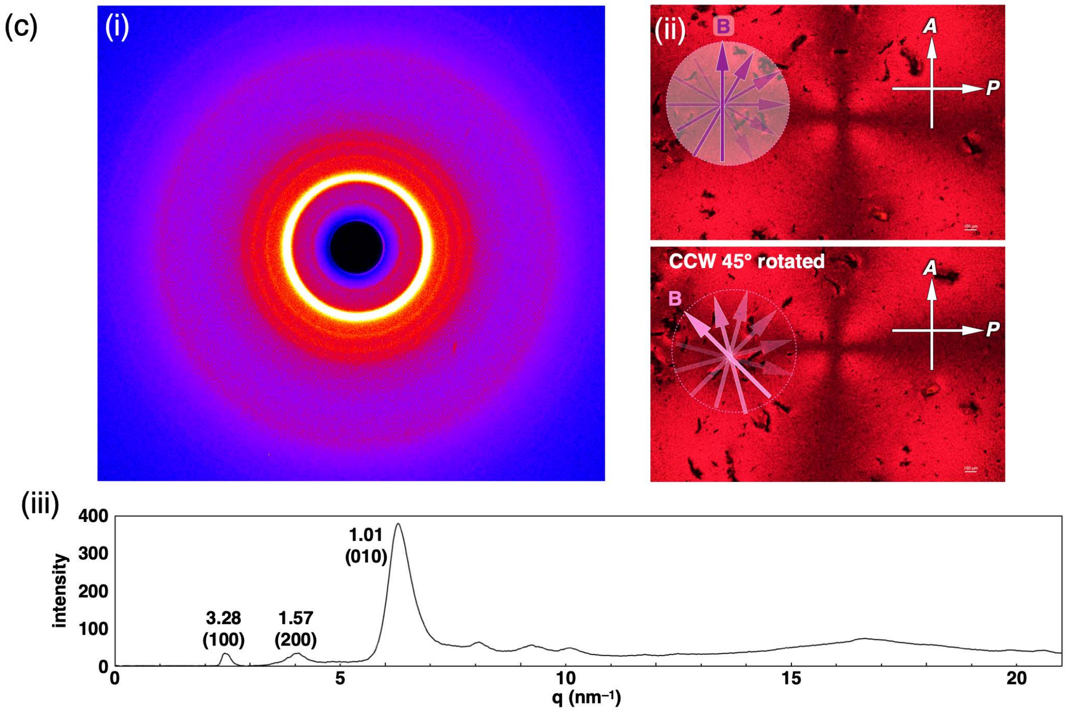

Figure S130 (Continued)

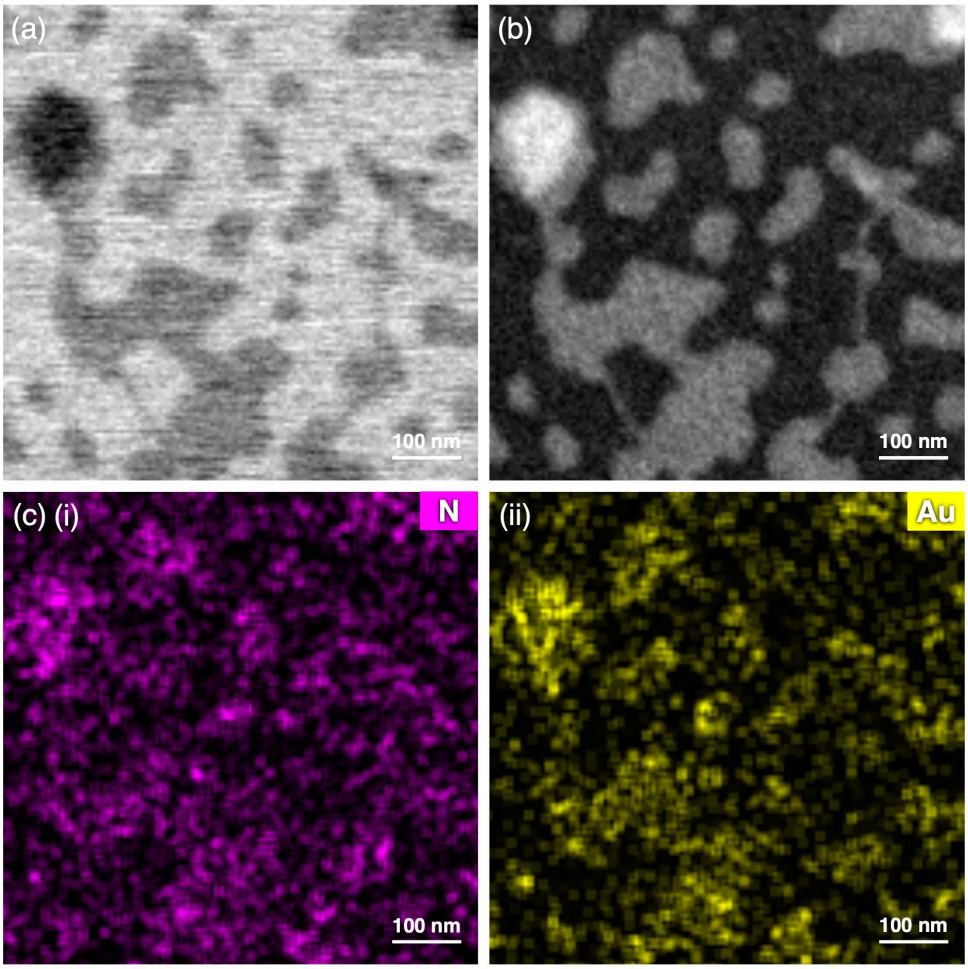

Figure S131 (a) Scanning transmission electron microscopy (STEM) image, (b) high-angle annular dark-field STEM (HAADF-STEM) image, and (c) STEM energy dispersive X-ray spectroscopy (EDS) images for (i) nitrogen (purple) and (ii) gold (yellow) elements of **1au**$^{+}$-PCCp^{-} as monolayer sheet-like structures formed in aqueous solutions (25 μM).

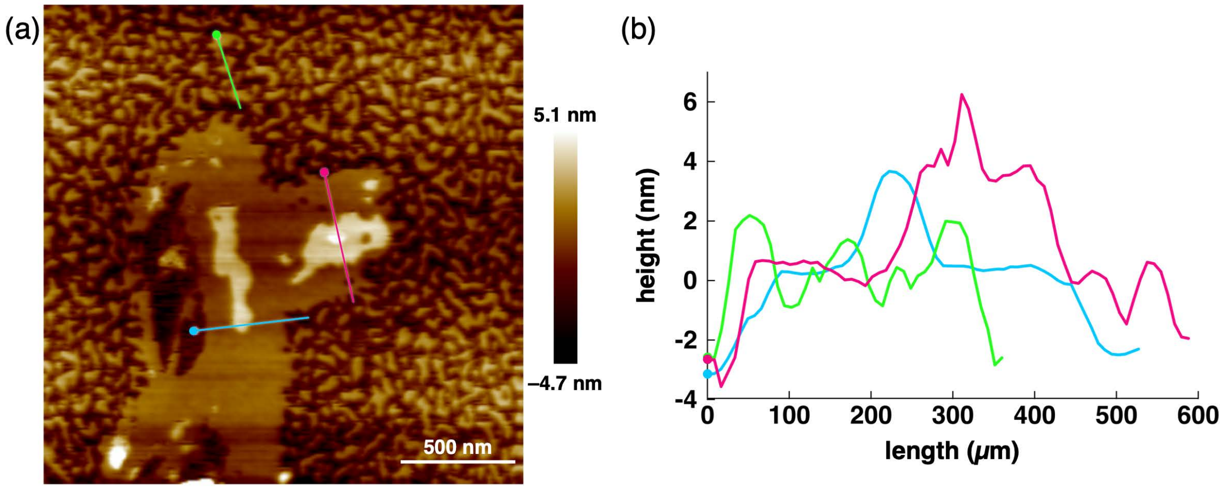

Figure S132 (a) AFM image of charge-by-charge-based sheet-like structures of **1au**$^{+}$-PCCp^{-} formed in aqueous solutions (25 μM) and (b) height profiles of sheet-like structures measured across the green, red, and blue lines in (a).
